# Spo13/MEIKIN ensures a Two‐Division meiosis by preventing the activation of APC/C^Ama1^ at meiosis I

**DOI:** 10.15252/embj.2023114288

**Published:** 2023-09-20

**Authors:** Julie Rojas, Tugce Oz, Katarzyna Jonak, Oleksii Lyzak, Vinal Massaad, Olha Biriuk, Wolfgang Zachariae

**Affiliations:** ^1^ Laboratory of Chromosome Biology Max Planck Institute of Biochemistry Martinsried Germany; ^2^ Present address: Laboratory of Genetics University of Wisconsin‐Madison Madison WI USA; ^3^ Present address: Institute of Biochemistry and Biophysics Polish Academy of Sciences Warsaw Poland

**Keywords:** cyclin phosphorylation, meiotic exit, polo‐like kinase, Spo13/MEIKIN, translational repression, Cell Cycle

## Abstract

Genome haploidization at meiosis depends on two consecutive nuclear divisions, which are controlled by an oscillatory system consisting of Cdk1‐cyclin B and the APC/C bound to the Cdc20 activator. How the oscillator generates exactly two divisions has been unclear. We have studied this question in yeast where exit from meiosis involves accumulation of the APC/C activator Ama1 at meiosis II. We show that inactivation of the meiosis I‐specific protein Spo13/MEIKIN results in a single‐division meiosis due to premature activation of APC/C^Ama1^. In the wild type, Spo13 bound to the polo‐like kinase Cdc5 prevents Ama1 synthesis at meiosis I by stabilizing the translational repressor Rim4. In addition, Cdc5‐Spo13 inhibits the activity of Ama1 by converting the B‐type cyclin Clb1 from a substrate to an inhibitor of Ama1. Cdc20‐dependent degradation of Spo13 at anaphase I unleashes a feedback loop that increases Ama1's synthesis and activity, leading to irreversible exit from meiosis at the second division. Thus, by repressing the exit machinery at meiosis I, Cdc5‐Spo13 ensures that cells undergo two divisions to produce haploid gametes.

## Graphical abstract

Polo‐like kinase Cdc5 and yeast Spo13 synergize in repressing the cell division exit machinery to prevent meiosis with only a single division.

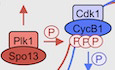

## Introduction

Meiosis consists of two consecutive nuclear divisions whereby chromosomes segregate reductionally at meiosis I and equationally at meiosis II. Reductional segregation depends on recombination, which forms bivalent chromosomes by generating crossovers between homologous, maternal, and paternal chromosomes (Petronczki *et al*, 2003). In addition, sister kinetochores fuse their microtubule‐binding interfaces so that maternal and paternal centromeres are pulled to opposite spindle poles. In yeast, this process, called sister kinetochore mono‐orientation, requires recruitment of the monopolin complex to kinetochores (Toth *et al*, 2000; Rabitsch *et al*, 2003; Petronczki *et al*, 2006). At meiosis I, dyad chromosomes segregate because the separase protease cleaves cohesin on chromosome arms (Klein *et al*, 1999; Buonomo *et al*, 2000), while centromeric cohesin is protected from cleavage by a complex consisting of shugoshin (Sgo1 in yeast) and the phosphatase PP2A (Riedel *et al*, 2006; Katis *et al*, 2010). Segregation of chromatids at meiosis II requires the removal of monopolin and Sgo1‐PP2A from centromeres, so that sister kinetochores orient towards opposite spindle poles and separase gains access to centromeric cohesin.

The meiosis I‐specific behaviour of centromeres depends on a group of weakly conserved proteins represented by Spo13 in budding yeast, Moa1 in fission yeast, and MEIKIN in mammals (Nasmyth, 2015). These proteins appear intrinsically disordered, are present only at meiosis I, and bind to polo‐like kinase (PLK1, Cdc5 in yeast). Indeed, they are thought to promote sister kinetochore mono‐orientation and centromeric cohesin protection by recruiting Cdc5/PLK1 to centromeres (Kim *et al*, 2015; Galander *et al*, 2019; Ma *et al*, 2021). However, centromere‐related functions cannot account for a striking aspect of the *spo13Δ* phenotype. While mutants lacking monopolin and the ability to protect centromeric cohesin undergo two divisions (Toth *et al*, 2000; Rabitsch *et al*, 2003), *spo13Δ* cells exit from meiosis after a single division (Klapholz & Esposito, 1980b; Shonn *et al*, 2002). Similarly, spermatocytes from *Meikin* ^−/−^ mice undergo only one division (Kim *et al*, 2015). Thus, a hitherto unexplained aspect of the *spo13Δ* and the *Meikin* ^−/−^ phenotype is premature exit from meiosis, indicating that Spo13/MEIKIN controls processes beyond the centromere. Indeed, the finding that *spo13Δ* cells undergo nuclear division in the absence of the APC/C activator Cdc20 implies a dramatic change in cell cycle regulation (Katis *et al*, 2004).

Meiosis is characterized by a period of low Cdk1 activity at prophase followed by two consecutive waves of Cdk1 activity during nuclear divisions. Unravelling how this activity pattern is generated and how it accommodates checkpoint‐dependent delays is crucial to our understanding of meiosis. Cdk1‐cyclin B and APC/C^Cdc20^ constitute an oscillatory system capable of generating successive M phases (Sha *et al*, 2003; Tyson & Novak, 2008). Cdk1 bound to B‐type cyclins (Clbs in yeast) induces spindle formation and is required for the ubiquitin‐ligase activity of APC/C^Cdc20^. Once activated, APC/C^Cdc20^ induces chromosome segregation by mediating the degradation of the separase‐inhibitor Pds1/securin and cyclin B. As cyclin B levels decline, the activity of APC/C^Cdc20^ decreases, which allows re‐accumulation of Pds1 and cyclin B, resulting in entry into meiosis II. The Cdk1‐APC/C^Cdc20^ oscillator is modulated by checkpoint controls. For instance, in the absence of tension exerted by bipolar spindle forces, kinetochores inhibit APC/C^Cdc20^ and thereby prolong metaphase through a signalling cascade, known as the spindle assembly checkpoint (SAC; Musacchio, 2015). To ensure a two‐division meiosis, SAC activity at metaphase I has to inhibit not only entry into anaphase I but also processes specific to meiosis II, although mRNAs encoding meiosis II‐specific proteins are already present at meiosis I (Brar *et al*, 2012).

In budding yeast, two meiosis‐specific proteins, the transcription factor Ndt80 and the APC/C activator Ama1, are crucial for starting and stopping the oscillator (Chu & Herskowitz, 1998; Cooper *et al*, 2000). During prophase, the recombination checkpoint represses Ndt80 and thereby the synthesis of M phase regulators (Chu & Herskowitz, 1998; Hepworth *et al*, 1998). In addition, low levels of APC/C^Ama1^ activity render M phase regulators unstable (Okaz *et al*, 2012). Once recombination is complete, Ndt80 produces Clbs and Cdc20, the key components of the oscillator (Chu *et al*, 1998). As cells enter meiosis II, Ama1 accumulates to high levels, which suppresses the Cdk1‐APC/C^Cdc20^ oscillator due to degradation of M phase regulators, including Ndt80. Similar mechanisms operate in fission yeast: The transcription factor Mei4 produces M phase regulators (Horie *et al*, 1998; Murakami‐Tonami *et al*, 2007), while high levels of the APC/C activator Fzr1/Mfr1 induce exit from meiosis (Asakawa *et al*, 2001; Blanco *et al*, 2001). Ama1 is related to the APC/C activator Cdh1, which is active during pre‐meiotic G1 but inhibited at other stages of meiosis (Oelschlaegel *et al*, 2005; Holt *et al*, 2007). Mammalian genomes encode only two APC/C activators, namely Cdc20 and Cdh1, whereby Cdh1's role resembles that of Ama1. Cdh1 prevents premature accumulation of cyclin B at prophase (Holt *et al*, 2011, 2014), and hyperactive Cdh1 causes spermatocytes to exit from meiosis after a single division (Tanno *et al*, 2020).

How exit from meiosis is confined to the second division is poorly understood. APC/C^Ama1^ is inhibited by high levels of Cdk1 activity (Oelschlaegel *et al*, 2005; Okaz *et al*, 2012). Thus, by initiating Clb degradation, APC/C^Cdc20^ might activate APC/C^Ama1^ at anaphase II and thereby induce exit from meiosis. How APC/C^Ama1^ is inhibited at meiosis I is unclear. Three properties of Ama1 might make this a challenging task: First, Ama1 accumulates at metaphase II of an unperturbed meiosis. However, it appears already at metaphase I, when the latter is prolonged due to the inhibition of Cdc20 (Oelschlaegel *et al*, 2005). Second, by destroying its inhibitor, Cdk1‐Clb, APC/C^Ama1^ increases its own activity. Once unleashed, this feedback loop triggers an irreversible exit from M phase (Okaz *et al*, 2012). Third, since Ama1 is not affected by the SAC, APC/C^Ama1^ activity threatens to curtail metaphase I. Confining APC/C^Ama1^ activity to meiosis II might therefore be crucial for the ability of the SAC to prolong metaphase I and, indeed, for performing a second division.

We show here that the Cdc5‐Spo13 kinase ensures a two‐division meiosis by preventing premature activation of APC/C^Ama1^. At metaphase I, Cdc5‐Spo13 inhibits the synthesis of Ama1 by stabilizing the translational repressor Rim4. Furthermore, by phosphorylating the cyclin Clb1, Cdc5‐Spo13 generates an inhibitor of APC/C^Ama1^. At anaphase I, Cdc20‐dependent degradation of Spo13 unleashes synthesis and activation of Ama1, leading to exit from meiosis at the second division. In the *spo13* mutant, premature accumulation and activation of Ama1 causes exit from meiosis after a single division, even in the absence of Cdc20.

## Results

### Properties of the single‐division meiosis in the *spo13Δ* mutant

**Figure 1:**
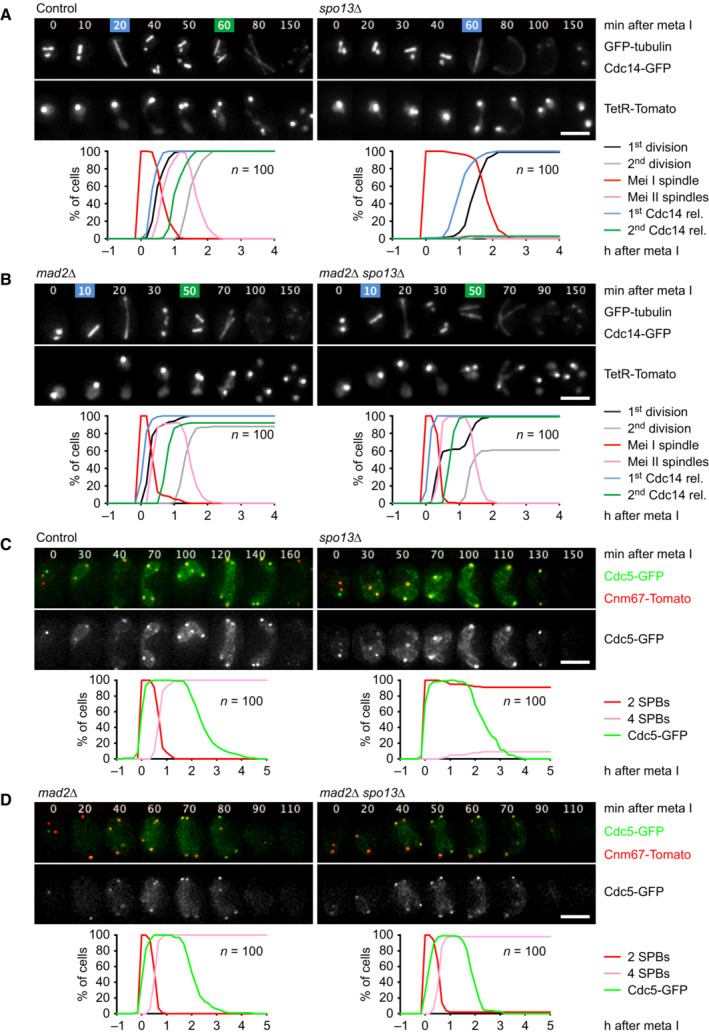
Analysis of meiotic progression in *spo13Δ* mutants A, BImaging of spindles (GFP‐tubulin), nucleolar release of Cdc14‐GFP, and TetR‐Tomato, which labels the nucleoplasm (diffuse signal) and the centromeres of one copy of chromosome V (dots). Top, time‐lapse series. First (blue) and second (green) Cdc14 release are marked. Bottom, meiotic events quantified in cells synchronized *in silico* to spindle formation at metaphase I (*t* = 0). (A) *spo13Δ* cells show a longer metaphase I (66 ± 21 min) than control cells (26 ± 9 min, *P* < 0.0001) and a single round of spindle formation and Cdc14 release. (B) *mad2Δ spo13Δ* cells show a short metaphase I (10 ± 5 min), similar to *mad2Δ* cells (10 ± 8 min, *P* = 0.68), and undergo two rounds of spindle formation and Cdc14 release. Cells in (A) and (B) were filmed together.C, DImaging of Cdc5‐GFP and SPBs (Cnm67‐Tomato). Top, time‐lapse series. Bottom, the presence of Cdc5 and SPB reduplication quantified in cells synchronized *in silico* to SPB separation at metaphase I (*t* = 0). (C) While control cells degrade Cdc5 at meiosis II (four SPBs), *spo13Δ* cells do so at meiosis I (two SPBs). (D) *mad2Δ* and *mad2Δ spo13Δ* cells degrade Cdc5 at meiosis II (four SPBs). Cells in (C) and (D) were filmed together. Data information: Data are representative of three (A) or two (B and C) independent experiments. Means were compared using Welch's *t*‐test. Scale bar, 4 μm. Source data are available online for this figure.

The single division of the *spo13Δ* mutant shows at least three unusual properties: First, these cells perform only one round of spindle formation (metaphase) and release of the Cdc14 phosphatase from the nucleolus (anaphase), which implies a single wave of Cdk1 and APC/C activity (Fig 1A). However, the division is preceded by recombination, an aspect of meiosis I, but concluded by exit from meiosis and spore formation, which are features of meiosis II (Klapholz & Esposito, 1980b). Second, *spo13Δ* mutants undergo Cdc14 release, nuclear division, and sporulation even when Cdc20 is depleted, which causes otherwise normal cells to arrest at metaphase I (Katis *et al*, 2004; Appendix Fig S1A). Thus, the *spo13Δ*‐division might be promoted by Ama1 or Cdh1, although these APC/C activators are thought to be inhibited during M phase (Zachariae *et al*, 1998; Oelschlaegel *et al*, 2005). Third, the *spo13Δ*‐division depends on the duration of metaphase I. While a normal metaphase I lasts 26 min, *spo13Δ* cells show a metaphase of 66 min, which is reduced to 10 min upon deletion of the SAC protein Mad2 (Fig 1A and B; Shonn *et al*, 2002). Remarkably, *spo13Δ mad2Δ* cells undergo two rounds of spindle formation and Cdc14 release (Fig 1B). However, 40% of these cells do not undergo nuclear division until entry into meiosis II, probably because sister kinetochores biorient in the absence of Spo13 (Katis *et al*, 2004; Lee *et al*, 2004). Indeed, the meiosis I‐division is restored by *rec8‐18D*, a mutation that causes cleavage of centromeric cohesin at anaphase I (Appendix Fig S1B; Arguello‐Miranda *et al*, 2017). Thus, *spo13Δ* cells perform a single division when metaphase I is long but two divisions when metaphase I is short. We considered the possibility that *spo13Δ* cells undergo unrestrained sporulation, thereby cutting off the second division when the first is delayed. However, blocking spore formation does not restore a second division in *spo13Δ* cells (Appendix Fig S1C). We therefore investigated the idea that Spo13 prevents Ama1 or Cdh1 from mediating exit from meiosis when Cdc20 is inhibited.

### Spo13 prevents accumulation of the APC/C activator Ama1 at metaphase I

**Figure 2:**
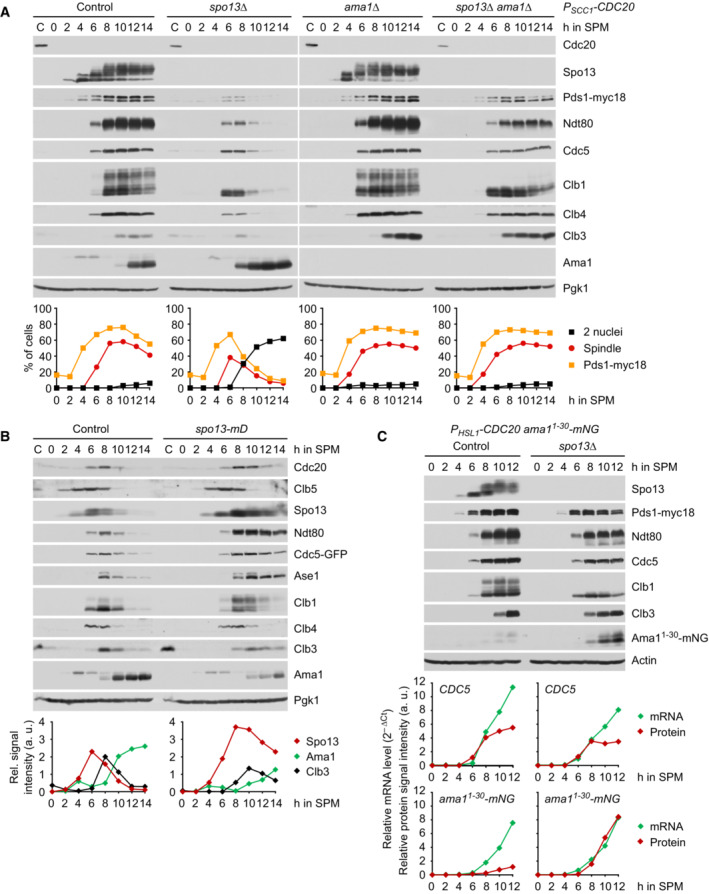
Spo13 prevents accumulation of Ama1 at metaphase I Deletion of *SPO13* causes Ama1‐dependent proteolysis and nuclear division in *P*_*SCC1*_ *‐CDC20* cells. Top, immunoblot detection of proteins. C, sample from proliferating cells. Bottom, progression of meiosis quantified in fixed cells.Spo13‐mD reduces Ama1 accumulation and Ama1‐dependent proteolysis at meiosis II. Top, immunoblot detection of proteins. Bottom, relative signal intensities of proteins.Analysis of mRNA and protein encoded by the *ama1* ^*1‐30*^ *‐mNG* locus in *P*_*HSL1*_ *‐CDC20* control and *spo13Δ* cells. Top, immunoblot detection of proteins. Bottom, mRNA levels relative to the *ACT1* transcript were quantified by RT‐qPCR and the 2^−ΔCt^ method. Signal intensities of proteins were normalized to Act1/actin. While control and *spo13Δ* cells produce *ama1* ^*1‐30*^ *‐mNG* mRNA, only *spo13Δ* cells synthesize mNG protein. Data information: (B) is representative of two independent experiments. Source data are available online for this figure.

To monitor the activity of APC/C^Ama1^, we imaged its substrate Cdc5‐GFP together with Cnm67‐tdTomato, a component of the spindle‐pole body (SPB, the yeast centrosome; Fig 1C and D). SPBs duplicate at S phase and separate at metaphase I to assemble the meiosis I‐spindle. SPBs then reduplicate at metaphase II to form a pair of meiosis II‐spindles (Moens & Rapport, 1971). As previously shown (Arguello‐Miranda *et al*, 2017), control cells degrade Cdc5 at anaphase II, after SPB reduplication. While *spo13Δ* cells prolong metaphase I and do not reduplicate SPBs, they degrade Cdc5 with similar kinetics as control cells (Fig 1C). Thus, *spo13Δ* cells degrade Cdc5 at meiosis I. By contrast, *spo13Δ mad2Δ* cells degrade Cdc5 after SPB reduplication, that is, at anaphase II (Fig 1D). One interpretation is that *spo13Δ* cells cannot restrain APC/C^Ama1^ activity when Cdc20 is inhibited by the SAC. To investigate this idea, we used the mitosis‐specific *SCC1* promoter to deplete cells of Cdc20. *P*_*SCC1*_ *‐CDC20* cells arrest at metaphase I, whereby APC/C substrates persist and nuclei do not divide. However, deletion of *SPO13* results in the degradation of APC/C substrates and nuclear division, which depends on Ama1 (Fig 2A), but not on Cdh1 (Appendix Fig S2A). Our data suggest that Spo13 prevents the activation of APC/C^Ama1^, and thereby premature exit from metaphase I, when Cdc20 is inhibited by the SAC or completely absent.

How does Spo13 restrain the activity of APC/C^Ama1^? In the wild‐type, Ama1 is present at low levels at prophase but accumulates to high levels as cells enter meiosis II at ~8 h after induction of meiosis (Fig 2B, left; Oelschlaegel *et al*, 2005). Indeed, degradation of Cdc5 and Ndt80 at anaphase II requires high levels of Ama1 and is blocked when Ama1 is expressed at prophase‐levels (Arguello‐Miranda *et al*, 2017). Depletion of Cdc20 strongly delays, but ultimately fails to suppress, the accumulation of Ama1. Thus, *P*_*SCC1*_ *‐CDC20* cells start to accumulate Ama1 at 12 h after induction of meiosis (Fig 2A). Remarkably, deletion of *SPO13* advances accumulation and binding to the APC/C of Ama1 by 4 h (Fig 2A and Appendix Fig S2B). These data suggest that Ama1 accumulation is promoted by Cdc20 and repressed by Spo13, a substrate of APC/C^Cdc20^ (Sullivan & Morgan, 2007). Deletion of *SPO13* also advances the appearance of the Clb3 cyclin (Fig 2A). This is more obvious in strains in which Clb3 is stable due to the absence of both Cdc20 and Ama1. Similar to Ama1, the accumulation of Clb3 is usually confined to meiosis II (Carlile & Amon, 2008).

To test whether Ama1 accumulation requires Cdc20‐dependent degradation of Spo13, we mutated the D‐box of Spo13 (Sullivan & Morgan, 2007). Spo13‐mD accumulates to higher levels and persists longer than wild‐type Spo13 (Fig 2B; Oz *et al*, 2022). In *spo13‐mD* cells, Ama1 levels do not exceed 50% of those in the control. This does not affect the degradation of Clb4 or Clb5, which are targeted by Cdc20 as well as Ama1. However, Spo13‐mD increases the formation of slow‐migrating species of Clb1, which persist slightly longer than the unmodified form. We shall analyse this modification further below. Importantly, Ase1, Cdc5, and Ndt80 are stabilized in *spo13‐mD* cells. These proteins are normally degraded at anaphase II in a manner dependent on high levels of Ama1 (Okaz *et al*, 2012; Arguello‐Miranda *et al*, 2017). Live‐imaging confirms that *spo13‐mD* cells fail to properly degrade Cdc5 at meiosis II (Appendix Fig S2C). Our data suggest that Cdc20‐dependent degradation of Spo13 at anaphase I is required for Ama1 accumulation and Ama1‐dependent proteolysis at meiosis II. Interestingly, Spo13 degradation is also required for normal accumulation of Clb3 (Fig 2B).

### Spo13 is required for repressing *AMA1* translation at metaphase I

**Figure EV1:**
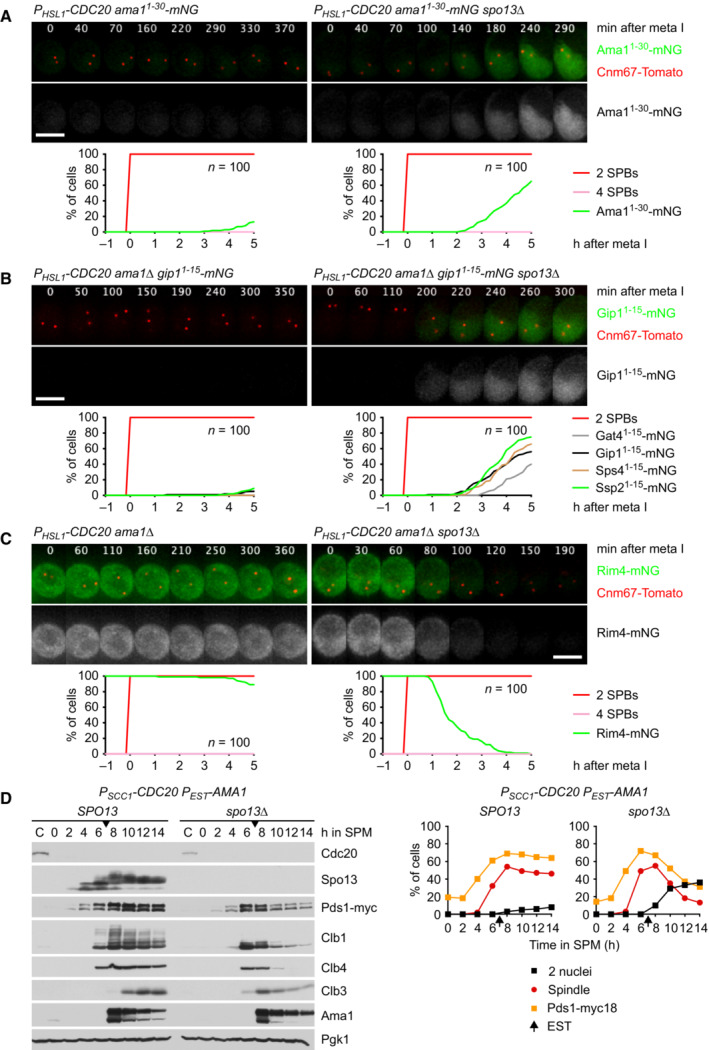
Regulation of translation by Spo13 Deletion of *SPO13* causes *AMA1* translation in Cdc20‐depleted cells. Top, time‐lapse series from the imaging of mNG and SPBs (Cnm67‐Tomato) in *P*_*HSL1*_ *‐CDC20* control and *spo13Δ* cells carrying *ama1* ^*1‐30*^ *‐mNG* in place of *AMA1*. Bottom, the presence of mNG quantified in cells synchronized *in silico* to SPB separation at metaphase I (*t* = 0).Sporulation genes showing Spo13‐dependent translational repression in metaphase I‐arrested *P*_*HSL1*_ *‐CDC20 ama1Δ* strains. Top, time‐lapse series from the imaging of mNG and SPBs (Cnm67‐Tomato) in control and *spo13Δ* cells carrying *gip1* ^*1‐15*^‐*mNG* in place of *GIP1*. Bottom, the presence of mNG quantified in cells in which *mNG* replaces coding sequences downstream of codon 15 of *GAT4*, *GIP1*, *SPS4*, or *SSP2*. Cells have been synchronized *in silico* to SPB separation at metaphase I (*t* = 0).Deletion of *SPO13* causes Rim4 degradation in metaphase I‐arrested *P*_*HSL1*_ *‐CDC20 ama1Δ* cells. Top, time‐lapse series from the imaging of Rim4‐mNG and SPBs (Cnm67‐Tomato). Bottom, the presence of Rim4‐mNG quantified in cells synchronized *in silico* to SPB separation at metaphase I (*t* = 0).Deletion of *SPO13* causes degradation of APC/C substrates and nuclear division in Cdc20‐depleted (*P*_*SCC1*_ *‐CDC20*) cells expressing *AMA1* from an estradiol‐inducible *GAL* promoter (*P*_*EST*_ *‐AMA1*) at *t* = 7 h in SPM (arrows). Left, immunoblot detection of proteins. Right, progression of meiosis quantified in fixed cells. Data information: Data are representative of three (A and C) or two (B) independent experiments. Scale bar, 4 μm. Source data are available online for this figure.

While *CLB3* transcription is induced by Ndt80 at metaphase I, *CLB3* translation is repressed until entry into meiosis II (Carlile & Amon, 2008; Berchowitz *et al*, 2013). To test whether *AMA1* expression is controlled by a similar mechanism, we used cells expressing Cdc20 from the mitosis‐specific *HSL1* promoter and replaced *AMA1* sequences downstream of codon 30 with a fragment encoding the fluorescent protein mNeonGreen (mNG; Shaner *et al*, 2013). Since these cells lack Ama1 activity, they stably arrest at metaphase I, even when *SPO13* is deleted. In control cells, *ama1* ^*1‐30*^ *‐mNG* and *CDC5* transcripts start to accumulate at 6 h after induction of meiosis (Fig 2C, green lines). While *CDC5* mRNA is promptly translated, the mNG protein is barely detectable by immunoblotting (Fig 2C, red lines) or live‐imaging (Fig EV1A). Importantly, deletion of *SPO13* has little effect on *ama1* ^*1‐30*^ *‐mNG* transcription but causes accumulation of mNG. These data confirm that *AMA1* transcription is induced at metaphase I (Chu *et al*, 1998; Primig *et al*, 2000), while translation is repressed by a Spo13‐dependent mechanism. Recent work has identified at least 20 genes involved in spore formation, which show Ndt80‐dependent transcription and delayed translation, either at entry into metaphase II (e.g., *GAT4* and *GIP1*) or at late anaphase II (e.g., *SPS4* and *SSP2*; Brar *et al*, 2012; Whinston *et al*, 2013; Jin *et al*, 2015). To monitor translation at metaphase I, we replaced coding sequences with the *mNG* fragment in *P*_*HSL1*_ *‐CDC20 ama1Δ* cells (Fig EV1B). *mNG* integrated at any one of these genes is not translated at metaphase I unless *SPO13* is deleted. This suggests that several sporulation genes are subject to Spo13‐dependent translational repression at meiosis I and explains how *spo13Δ* mutants can sporulate at meiosis I (Oz *et al*, 2022).

**Figure 3:**
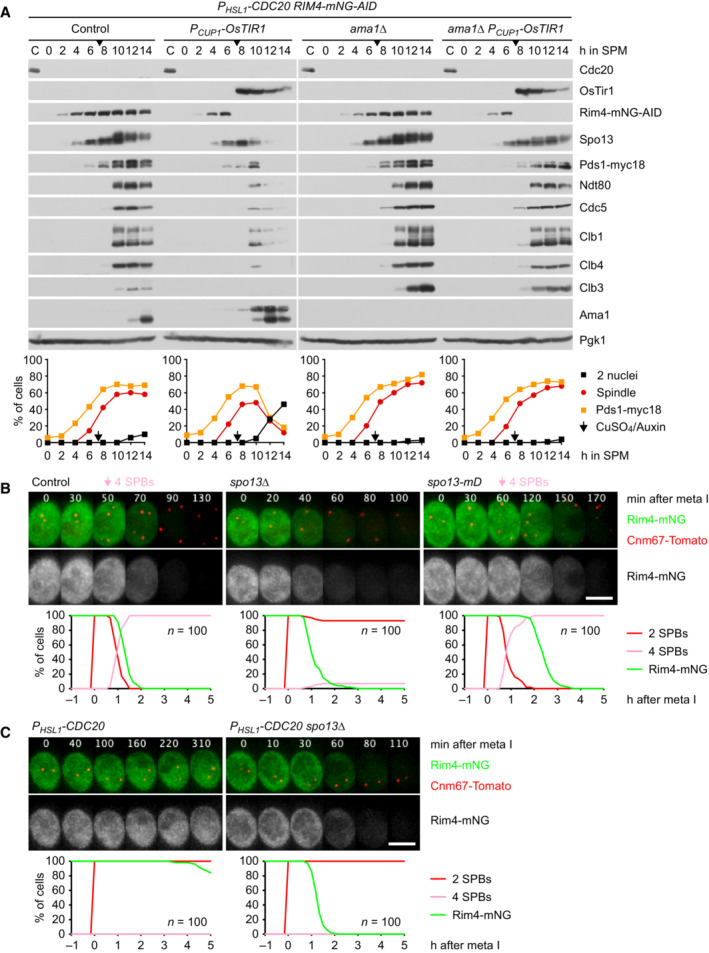
Spo13 controls Ama1 accumulation via the translational repressor Rim4 AAuxin‐inducible degradation of Rim4 causes Ama1‐dependent proteolysis in *P*_*HSL1*_ *‐CDC20* cells. At *t* = 7.5 h (arrows), CuSO_4_ and the auxin 5‐Ph‐IAA were added to induce expression and activation of OsTir1, resulting in degradation of AID‐tagged Rim4‐mNG. Top, immunoblot detection of proteins. Bottom, progression of meiosis quantified in fixed cells.B, CImaging of Rim4‐mNG and SPBs (Cnm67‐Tomato). Top, time‐lapse series. Bottom, the presence of Rim4‐mNG and SPB reduplication quantified in cells synchronized *in silico* to SPB separation at metaphase I (*t* = 0). (B) While control cells degrade Rim4 at meiosis II (four SPBs), *spo13Δ* mutants do so at meiosis I (two SPBs). Spo13‐mD delays Rim4 degradation by 64 min (95% CI, 58–69 min; *P* < 0.0001, Welch's *t*‐test). (C) Deletion of *SPO13* causes *P*_*HSL1*_ *‐CDC20* cells to degrade Rim4. Data information: Data are representative of two (B) or three (C) independent experiments. Scale bar, 4 μm. Source data are available online for this figure.

Translation of *CLB3* and *SPS4* is repressed by the RNA‐binding protein Rim4, which is degraded at entry into metaphase II. Furthermore, these mRNAs and those encoding Ama1 were found among mRNAs copurifying with Rim4 (Berchowitz *et al*, 2013; Jin *et al*, 2015; Carpenter *et al*, 2018). To test whether Rim4 represses *AMA1* translation at metaphase I, we used an auxin‐inducible degradation (AID) system (Yesbolatova *et al*, 2020) to remove Rim4 from Cdc20‐depleted cells (Fig 3A). Indeed, removal of Rim4 advances the accumulation of Ama1 by ~4 h. As a result, cells degrade APC/C substrates and undergo nuclear division in an Ama1‐dependent manner. Consistent with previous work (Berchowitz *et al*, 2013), removal of Rim4 also advances the synthesis of Clb3, which is most evident in strains lacking both Cdc20 and Ama1 (Fig 3A). We conclude that Rim4 prevents accumulation of Ama1 as well as Clb3 when metaphase I is prolonged due to the inhibition of Cdc20.

### Spo13 prevents degradation of the translational repressor Rim4 at metaphase I

The consequences of removing Rim4 from Cdc20‐depleted cells resemble those of deleting *SPO13*. Thus, Spo13 might restrain the accumulation of Ama1 by preventing the degradation of Rim4. To test this, we imaged Rim4‐mNG together with SPBs carrying Cnm67‐tdTomato. In control cells, Rim4 disappears shortly after SPBs reduplicate at entry into metaphase II (Fig 3B). While *spo13Δ* cells prolong metaphase I and do not reduplicate SPBs, they nevertheless degrade Rim4. Thus, *spo13Δ* cells degrade Rim4 at meiosis I. In *spo13‐mD* cells, on the other hand, Rim4 degradation is delayed and therefore occurs well after entry into metaphase II (Fig 3B). These data suggest that Rim4 degradation is inhibited until Spo13 is subjected to APC/C‐dependent proteolysis. Accordingly, Rim4 persists in *P*_*HSL1*_ *‐CDC20* cells in which Spo13 is stable, but is rapidly degraded in *P*_*HSL1*_ *‐CDC20 spo13Δ* cells (Fig 3C). In fact, Rim4 is degraded with similar kinetics in *spo13Δ* cells containing or lacking Cdc20 (Fig 3B and C). While *P*_*HSL1*_ *‐CDC20 spo13Δ* double mutants undergo nuclear division in an Ama1‐dependent manner, nuclear division is not required for Rim4 degradation. The *SPO13* deletion also elicits Rim4 degradation in *P*_*HSL1*_ *‐CDC20 ama1Δ* cells, which stably arrest at metaphase I (Fig EV1C). We conclude that APC/C^Cdc20^ induces Rim4 degradation by mediating the proteolysis of its canonical substrate Spo13, which in turn unleashes the mechanism directly responsible for Rim4 degradation. Does Spo13 restrain Ama1‐dependent proteolysis solely by repressing *AMA1* translation via Rim4? If so, expression of Ama1 from a foreign promoter should eliminate the effect of the *SPO13* deletion. While the *SPO13* deletion has no effect on the accumulation of Ama1 expressed from an estradiol‐inducible *GAL* promoter, it nevertheless results in degradation of APC/C substrates and nuclear division (Fig EV1D). The implication is that Spo13 not only represses Ama1 synthesis but additionally employs a post‐translational mechanism to inhibit Ama1 activity. We shall return to this issue further below.

### Spo13 prevents Ime2 from promoting the degradation of Rim4 at metaphase I

**Figure 4:**
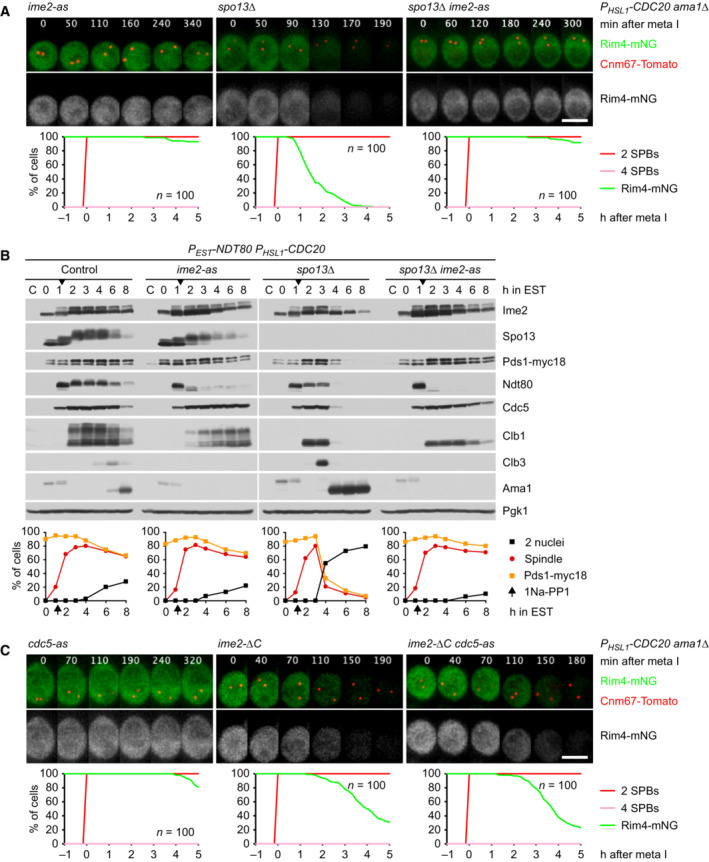
Ime2 activity is required for Rim4 degradation upon removal of Spo13 Inhibition of Ime2 activity prevents Rim4 degradation elicited by the *SPO13* deletion in metaphase I‐arrested *P*_*HSL1*_ *‐CDC20 ama1Δ* cells. Ime2‐as was inhibited with 1Na‐PP1 at 7 h in SPM. Top, time‐lapse series from the imaging of Rim4‐mNG and SPBs (Cnm67‐Tomato). Bottom, the presence of Rim4‐mNG quantified in cells synchronized *in silico* to SPB separation at metaphase I (*t* = 0).Inhibition of Ime2 activity blocks degradation of APC/C substrates and nuclear division in Cdc20‐depleted *spo13Δ* cells. *P*_*EST*_ *‐NDT80 P*_*HSL1*_ *‐CDC20* strains were released from prophase with estradiol (EST, *t* = 0), and Ime2‐as was inhibited at metaphase I (1Na‐PP1 at *t* = 75 min, arrows). Top, immunoblot detection of proteins. Bottom, progression of meiosis quantified in fixed cells.Ime2‐ΔC induces Rim4 degradation in metaphase I‐arrested *P*_*HSL1*_ *‐CDC20 ama1Δ* cells even when Cdc5 is inhibited. Cdc5‐as was inhibited with CMK at 8 h in SPM. Top, time‐lapse series from the imaging of Rim4‐mNG and SPBs (Cnm67‐Tomato). Bottom, the presence of Rim4‐mNG quantified in cells synchronized *in silico* to SPB separation at metaphase I (*t* = 0). Rim4 is degraded with similar timing in *ime2‐ΔC* cells with active or inactive Cdc5 (*P* = 0.88, Welch's *t*‐test). Data information: Data are representative of three (A) or two (C) independent experiments. Scale bar, 4 μm. Source data are available online for this figure.

Degradation of Rim4 at entry into metaphase II depends on the activity of the meiosis‐specific kinase Ime2 (Berchowitz *et al*, 2013; Carpenter *et al*, 2018). Ime2 activity is also required for premature Rim4 degradation in *spo13Δ* cells, as revealed by inhibition of analogue‐sensitive Ime2‐as (Benjamin *et al*, 2003) in the *P*_*HSL1*_ *‐CDC20 ama1Δ spo13Δ* triple mutant (Fig 4A). Next, we asked whether Ime2 activity is required for Ama1 translation in metaphase I‐arrested *spo13Δ* cells. However, Ime2 is required for the accumulation of Ndt80 and thereby for entry into metaphase I (Sopko *et al*, 2002). On the other hand, *cdc20 spo13Δ* cells start to produce Ama1 soon after entry into metaphase I. Thus, we used estradiol‐dependent expression of *NDT80* (*P*_*EST*_ *‐NDT80*; Benjamin *et al*, 2003) to arrest cells at prophase and subsequently release them into a metaphase I‐arrest imposed by depletion of Cdc20. 1Na‐PP1 was added shortly after cells had accumulated Ndt80 and entered metaphase I. While *P*_*EST*_ *‐NDT80 P*_*HSL1*_ *‐CDC20* control cells produce little Ama1, deletion of *SPO13* causes robust accumulation of Ama1, which results in degradation of APC/C substrates and nuclear division (Fig 4B). Importantly, inhibition of Ime2 prevents accumulation of Ama1, degradation of APC/C substrates, and nuclear division. Ime2 activity is also required for the transient accumulation of Clb3 elicited by the *SPO13* deletion. We conclude that Spo13 prevents Ime2 from promoting the degradation of Rim4 and thereby translation of *AMA1* as well as *CLB3* at metaphase I. Our data predict that hyperactivation of Ime2 promotes Rim4 degradation even in the presence of Spo13. To test this, we used Ime2‐ΔC, a kinase that is more stable and active due to the removal of the C‐terminal, regulatory domain (Sari *et al*, 2008). Indeed, Ime2‐ΔC induces Rim4 degradation in *P*_*HSL1*_ *‐CDC20 ama1Δ* cells, albeit with slower kinetics than the *SPO13* deletion (Fig 4C, middle). Furthermore, Ime2‐ΔC causes *P*_*HSL1*_ *‐CDC20* cells to translate *ama1* ^*1‐30*^ *‐mNG* mRNA into mNG protein (Appendix Fig S3A). Thus, Ime2‐ΔC uncouples Rim4 degradation from the inactivation of Spo13, suggesting that wild‐type Ime2 functions downstream of and is inhibited by Spo13.

### Ime2 is inhibited by Cdc5‐Spo13 and activated by free Cdc5

**Figure EV2:**
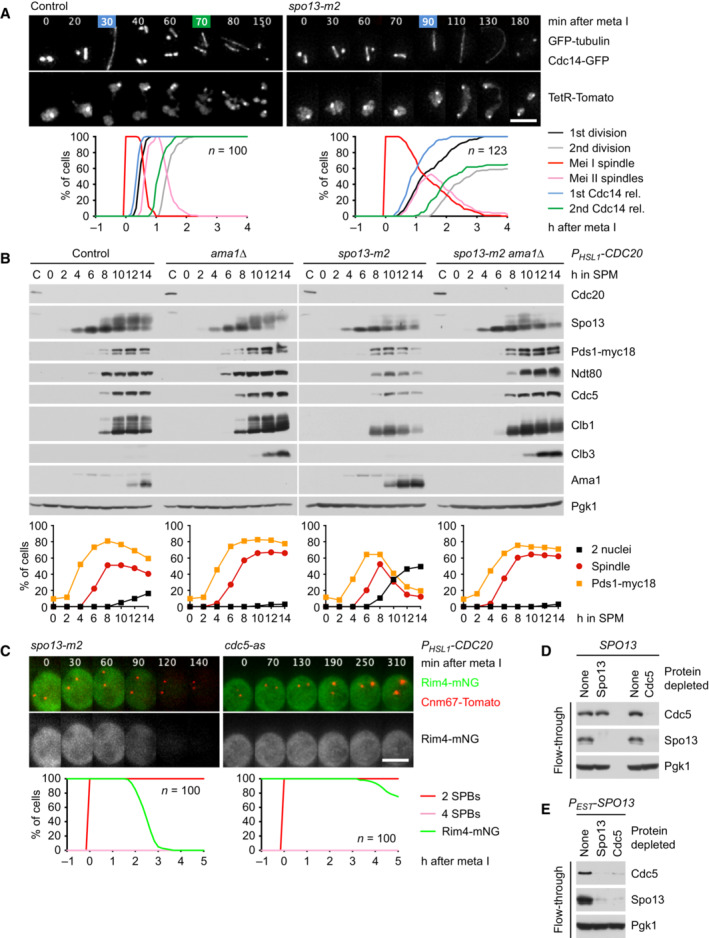
Analysis of the *spo13‐m2* mutation AMeiosis in control and *spo13‐m2* cells. Top, imaging of spindles (GFP‐tubulin), nucleolar release of Cdc14‐GFP, and TetR‐Tomato, which labels the nucleoplasm (diffuse signal) and the *URA3* locus of one copy of chromosome V (dots). First (blue) and second (green) Cdc14 release are marked. Bottom, meiotic events quantified in cells synchronized *in silico* to spindle formation at metaphase I (*t* = 0). *spo13‐m2* prolongs metaphase I to 60 ± 26 min (control, 23 ± 6 min; *P* < 0.0001, Welch's *t*‐test) and causes ~38% of cells to undergo a single round of spindle formation and Cdc14 release.B
*spo13‐m2* causes Ama1‐dependent proteolysis and nuclear division in *P*_*HSL1*_ *‐CDC20* cells. Top, immunoblot detection of proteins. Bottom, progression of meiosis quantified in fixed cells.CImaging of Rim4‐mNG and SPBs (Cnm67‐Tomato) in *P*_*HSL1*_ *‐CDC20* cells carrying *spo13‐m2* or *cdc5‐as*. Cdc5‐as was inhibited with CMK at 7.5 h in SPM. Top, time‐lapse series. Bottom, the presence of Rim4‐mNG quantified in cells synchronized *in silico* to SPB separation at metaphase I (*t* = 0).D, EExtracts from metaphase I‐arrested *P*_*HSL1*_ *‐CDC20* cells (8 h in SPM) were applied to columns carrying no antibody or antibodies to either Spo13 or Cdc5. Flow‐throughs were analyzed by immunoblotting. (D) Depletion of Spo13 from extracts of control cells (*SPO13*) removes little Cdc5 (10%). (E) Depletion of Spo13 from extracts of cells overexpressing Spo13 (*P*_*EST*_ *‐SPO13*, estradiol for 45 min) removes most of Cdc5 (97%). Data information: (B–D) are representative of two independent experiments. Scale bar, 4 μm. Source data are available online for this figure.

How does Spo13 inhibit Ime2's function in Rim4 degradation? Spo13 binds to the polo‐box domain (PBD) of Cdc5. A mutation in Spo13's PBD‐binding motif, called *spo13‐m2*, weakens this interaction and shows similar but less severe defects than the *SPO13* deletion (Matos *et al*, 2008; Oz *et al*, 2022). While the *spo13‐m2* mutation prolongs metaphase I to 60 min, it results in a lower incidence (~38%) of cells undergoing a single round of Cdc14 release than the *SPO13* deletion (> 90%; Fig EV2A). Nevertheless, the *spo13‐m2* mutation causes *P*_*HSL1*_ *‐CDC20* cells to advance the accumulation of Ama1 by 2 h (Fig EV4B). As a result, these cells degrade APC/C substrates and undergo nuclear division in an Ama1‐dependent manner. Furthermore, *P*_*HSL1*_ *‐CDC20 spo13‐m2* cells degrade Rim4, albeit with slower kinetics than the *spo13Δ* mutant (Fig EV2C). We conclude that binding to Cdc5 is required for Spo13's ability to prevent Ime2 from promoting the degradation of Rim4 at metaphase I.

**Figure 5:**
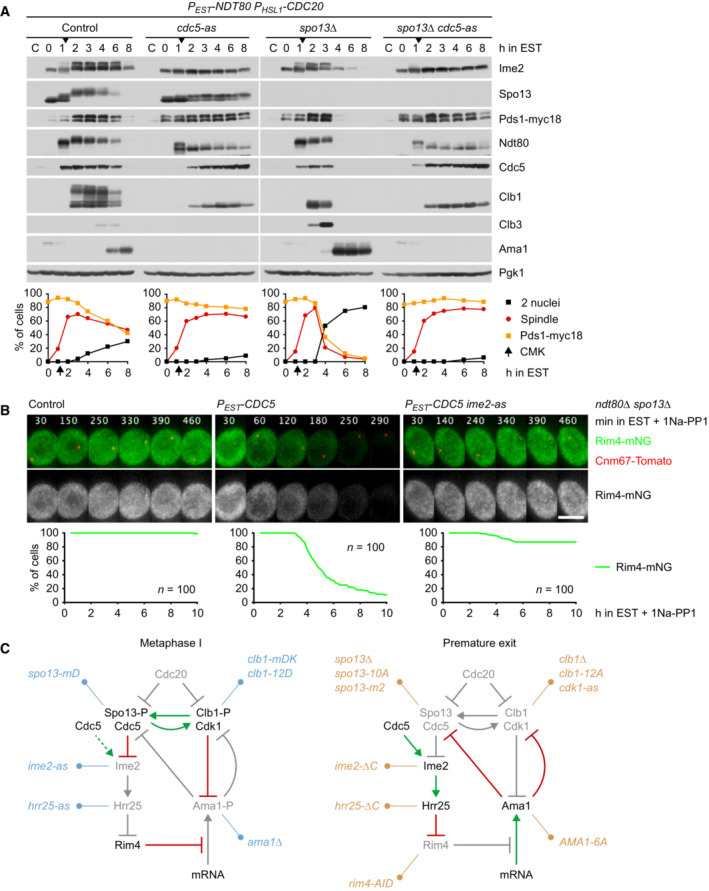
The role of Cdc5 in the control of Ama1 accumulation Inhibition of Cdc5 activity blocks degradation of APC/C substrates and nuclear division in Cdc20‐depleted *spo13Δ* cells. *P*_*EST*_ *‐NDT80 P*_*HSL1*_ *‐CDC20* strains were released from prophase with estradiol (EST, *t* = 0), and Cdc5‐as was inhibited at metaphase I (CMK at *t* = 75 min, arrows). Top, immunoblot detection of proteins. Bottom, progression of meiosis quantified in fixed cells.Cdc5 activity is required for Ime2‐dependent degradation of Rim4. *ndt80Δ spo13Δ* strains were allowed to arrest at prophase. At 4 h in SPM (*t* = 0), *P*_*EST*_ *‐CDC5* was induced with EST, and Ime2‐as was inhibited with 1Na‐PP1. Top, time‐lapse series from the imaging of Rim4‐mNG and SPBs (Cnm67‐Tomato). Bottom, quantification of the presence of Rim4‐mNG.The regulatory network controlling Ama1 synthesis and activity. Left, the low‐Ama1/high‐Cdk1 state of metaphase I. Right, the high‐Ama1/low‐Cdk1 state that induces premature exit from metaphase I. Green arrows: activation. Dashed, green arrow: latent activation overwhelmed by inhibition. Red, bar‐headed lines: inhibition. Gray items are inactive or absent. P denotes phosphorylation. Blue, mutations that stabilize metaphase I. Ochre, mutations causing exit from metaphase I. See text for details. Data information: (B) is representative of two independent experiments. Scale bar, 4 μm. Source data are available online for this figure.

By contrast, inhibition of analogue‐sensitive Cdc5‐as (Snead *et al*, 2007) in *P*_*HSL1*_ *‐CDC20* cells does not cause Rim4 degradation (Fig EV2C), Ama1 accumulation, or nuclear division (Fig 5A). Importantly, inhibition of Cdc5 blocks Ama1 accumulation in *P*_*HSL1*_ *‐CDC20 spo13Δ* cells (Fig 5A). From these data, one might presume that Spo13 functions as an inhibitor of Cdc5. However, we have recently shown that at metaphase I, the levels of Cdc5 greatly exceed those of Spo13 (Oz *et al*, 2022). Accordingly, depleting metaphase I‐extracts of Cdc5 co‐depletes most of Spo13, whereas Spo13 depletion removes only a small fraction of Cdc5 (Fig EV2D). However, Cdc5 is co‐depleted with Spo13 when Spo13 is overexpressed (Fig EV2E). These data argue against Spo13 being a stoichiometric inhibitor of Cdc5. Indeed, we show further below that Cdc5‐Spo13 is an active kinase, which coexists with free Cdc5 at metaphase I. What is the function of free Cdc5? Ime2‐ΔC restores Rim4 degradation in *P*_*HSL1*_ *‐CDC20 ama1Δ* cells even when Cdc5 is inhibited (Fig 4C), implying that wild‐type Ime2 promotes Rim4 degradation in a manner dependent on Cdc5 activity. To test this, we used *ndt80Δ spo13Δ* cells. *ndt80Δ* mutants arrest at prophase and express Ime2 but not Cdc5 (Benjamin *et al*, 2003; Clyne *et al*, 2003). *SPO13* was deleted to exclude any inhibitory effect on Ime2. Nevertheless, Rim4 accumulates in these cells, demonstrating that Ime2 is inactive towards Rim4 (Fig 5B). However, expression of Cdc5 causes degradation of Rim4, which in turn requires Ime2 activity. Our data suggest that while Cdc5‐Spo13 and free Cdc5 coexist at metaphase I, the former prevents Ime2 from promoting the degradation of Rim4. This enables Rim4 to repress the translation of *AMA1*. Proteolysis of Spo13 at anaphase I then allows Cdc5 to activate Ime2's function in Rim4 degradation (Fig 5C).

### Rim4 degradation depends on the activity of Hrr25/CK1δ

Our data raise a conundrum: In *spo13Δ* mutants, Cdc5 is required for the ability of Ime2 to promote Rim4 degradation and Ama1 accumulation (Figs 4A and 5A). However, as Ama1 accumulates, it mediates the degradation of Cdc5 (Fig 2A). This raises the question of how *AMA1* translation is sustained when Cdc5 levels decline. A related question arises at meiosis II when Ama1 accumulates despite mediating the degradation of Cdc5 (Arguello‐Miranda *et al*, 2017). To investigate this, we used *CDC20*‐meiotic‐arrest/release (*CDC20‐mAR*) cells, which are first arrested at metaphase I through the depletion of Cdc20 and then released into anaphase I by a copper‐inducible *CDC20* gene (Arguello‐Miranda *et al*, 2017). Inhibition of Cdc5 at anaphase I, shortly after the degradation of Spo13, reduces subsequent nuclear division due to the collapse of anaphase II spindles (Arguello‐Miranda *et al*, 2017) but has no effect on the accumulation of Ama1 (Appendix Fig S4). Similarly, inhibition of Ime2 at anaphase I reduces the stability of Ndt80 and hinders the meiosis II‐division but has little effect on the accumulation of Ama1 (Appendix Fig S4). Thus, while Rim4 degradation and Ama1 accumulation initially depend on Cdc5 and Ime2, they subsequently become independent of these two kinases. One possibility is that Ime2 induces an activity that promotes Rim4 degradation in a self‐sustaining manner.

**Figure EV3:**
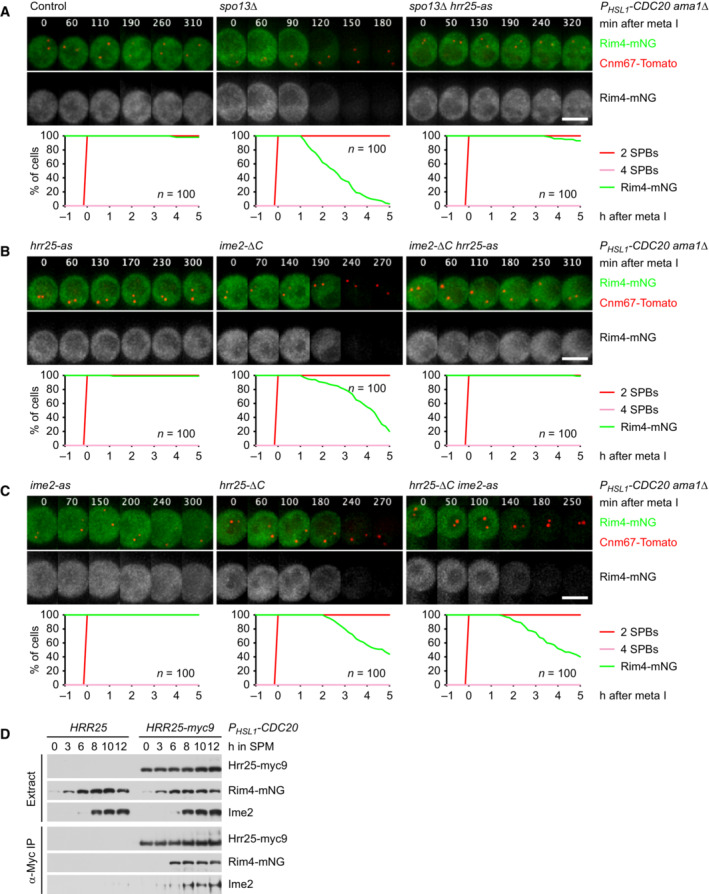
Regulation of Rim4 degradation by Hrr25 A–CImaging of Rim4‐mNG and SPBs (Cnm67‐Tomato) in metaphase I‐arrested *P*_*HSL1*_ *‐CDC20 ama1Δ* strains. Top, time‐lapse series. Bottom, the presence of mNG quantified in cells synchronized *in silico* to SPB separation at metaphase I (*t* = 0). (A) Inhibition of Hrr25 activity prevents Rim4 degradation elicited by the *SPO13* deletion. Hrr25‐as was inhibited with 1NM‐PP1 at 7 h in SPM. (B) Inhibition of Hrr25 prevents Rim4 degradation induced by hyperactive Ime2‐ΔC. Hrr25‐as was inhibited with 1NM‐PP1 at 7 h in SPM. (C) Rim4 is degraded with similar timing in *hrr25‐ΔC* cells with active or inactive Ime2 (*P* = 0.91, Welch's *t*‐test). Ime2‐as was inhibited with 1Na‐PP1 at 7 h in SPM.DBinding of Rim4‐mNG and Ime2 to Hrr25‐myc9 immunoprecipitated with α‐Myc antibodies from extracts of *P*_*HSL1*_ *‐CDC20* cells. Data information: Data are representative of two (A and B) or three (C) independent experiments. Scale bar, 4 μm. Source data are available online for this figure.

Consistent with previous work (Arguello‐Miranda *et al*, 2017), we find that accumulation of Ama1 and Clb3 at meiosis II depends on the activity of the casein kinase 1δ (CK1δ) Hrr25 (Appendix Fig S4). Accordingly, inhibition of Hrr25 blocks Rim4 degradation in metaphase I‐arrested *spo13Δ* mutants (Fig EV3A). This raises the question of where Hrr25 resides in the pathway controlling Rim4 degradation. While Ime2‐ΔC induces Rim4 degradation even when Cdc5 is inhibited (Fig 4C), it fails to do so when Hrr25 is inhibited (Fig EV3B). Thus, Hrr25 might function together with or downstream of Ime2. To test this, we generated a hyperactive Hrr25 kinase by removing the C‐terminal, autoinhibitory region common to CK1δ‐orthologues (Graves & Roach, 1995; Cegielska *et al*, 1998; Elmore *et al*, 2018). Hrr25‐ΔC has little effect on proliferation or meiotic nuclear division in otherwise normal cells (Appendix Fig S3B). On the other hand, it induces *AMA1* translation in metaphase I‐arrested cells (Appendix Fig S3C). Importantly, Hrr25‐ΔC induces Rim4 degradation even when Ime2 is inhibited (Fig EV3C), placing Hrr25 downstream of Ime2. Consistent with this, Hrr25 binds to Ime2 and Rim4 (Fig EV3D). Taken together, our data suggest that Cdc5‐Spo13 inhibits *AMA1* translation by preventing the Cdc5‐Ime2‐Hrr25 kinase cascade from inducing the degradation of Rim4 (Fig 5C). Recent work suggests that Rim4 is removed by autophagy (Wang *et al*, 2020; Herod *et al*, 2022), and that Hrr25 is required for different pathways of selective autophagy (Farre & Subramani, 2016). Interestingly, Hrr25 binds the Rab‐family GTPase Ypt1/Rab1, which activates Hrr25 and recruits it to the phagophore assembly site (PAS; Wang *et al*, 2015). Once activated, Hrr25 might sustain autophagic degradation of Rim4 independently of Ime2 and Cdc5.

**Figure 6:**
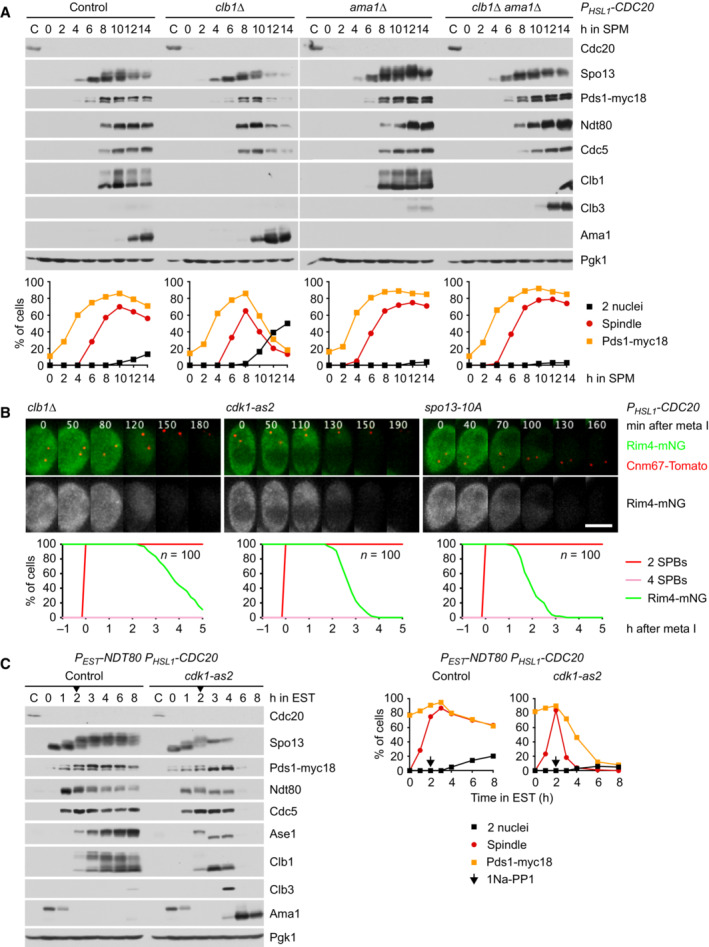
Cdk1‐Clb1 is required for preventing Ama1 accumulation at metaphase I Deletion of *CLB1* causes Ama1‐dependent proteolysis and nuclear division in *P*_*HSL1*_ *‐CDC20* cells. Top, immunoblot detection of proteins. Bottom, progression of meiosis quantified in fixed cells.Imaging of Rim4‐mNG and SPBs (Cnm67‐Tomato) in *P*_*HSL1*_ *‐CDC20* cells carrying *clb1Δ*, *cdk1‐as2*, or *spo13‐10A*. Cdk1‐as2 was inhibited with 1Na‐PP1 at 7.5 h in SPM. Top, time‐lapse series. Bottom, the presence of Rim4‐mNG quantified in cells synchronized *in silico* to SPB separation at metaphase I (*t* = 0).Inhibition of Cdk1 at metaphase I causes accumulation of Ama1 and degradation of APC/C substrates. *P*_*EST*_ *‐NDT80 P*_*HSL1*_ *‐CDC20* strains were released from prophase with estradiol (EST, *t* = 0), and Cdk1‐as2 was inhibited at metaphase I (1Na‐PP1 at *t* = 120 min, arrows). Left, immunoblot detection of proteins. Right, progression of meiosis quantified in fixed cells. Data information: (B) is representative of two independent experiments. Scale bar, 4 μm. Source data are available online for this figure.

### Cdk1‐Clb1 is required for the persistence of Rim4 at metaphase I

Cells lacking the M‐phase cyclin Clb1 resemble *spo13Δ* mutants in several aspects: First, the *CLB1* deletion prolongs metaphase I, which lasts for up to 200 min (mean, 81 min; control, 25 min), probably due to inefficient activation of APC/C^Cdc20^ (Rudner & Murray, 2000; Appendix Fig S5A). *clb1Δ* cells undergo either one (47%) or two (53%) rounds of Cdc14 release and nuclear division, whereby cells with a long metaphase I tend to undergo only one round (Appendix Fig S5B). Second, deletion of *CLB1*, but not of *CLB3* or *CLB4*, results in nuclear division in Cdc20‐depleted cells (Oz *et al*, 2022). Indeed, the *CLB1* deletion causes *P*_*HSL1*_ *‐CDC20* cells to advance the accumulation of Ama1 by 2 h, and the degradation of APC/C substrates as well as nuclear division depends on Ama1 (Fig 6A). The *CLB1* deletion also advances the appearance of Clb3, which is more obvious in *P*_*HSL1*_ *‐CDC20 ama1Δ* strains. Third, consistent with Ama1‐dependent proteolysis of Spo13, *P*_*HSL1*_ *‐CDC20 clb1Δ* cells degrade Rim4 (Fig 6B, left). We conclude that Clb1 as well as Spo13 is required for suppressing the accumulation of Ama1 and Clb3 at metaphase I.

To test whether the function of Clb1 depends on Cdk1 activity, we inhibited analogue‐sensitive Cdk1‐as2 (Bishop *et al*, 2000) shortly after *NDT80 P*_*HSL1*_ *‐CDC20* cells had entered metaphase I. This causes accumulation of Ama1 and Clb3, which is followed by the degradation of APC/C substrates, while spindle disassembly prevents nuclear division (Fig 6C). Live‐imaging revealed that inhibition of Cdk1 causes *P*_*HSL1*_ *‐CDC20* cells to degrade Rim4 (Fig 6B, middle). Thus, the consequences of inactivating Cdk1‐Clb1 resemble those of deleting *SPO13*. One possibility is that Cdk1 activity is required for an aspect of Spo13 function. We have recently shown that Spo13 requires phosphorylation by Cdk1 in order to suppress spore formation in Cdc20‐depleted cells. A non‐phosphorylatable mutant, called *spo13‐10A*, results in premature degradation of Spc72, the repressor of spore formation (Oz *et al*, 2022). Indeed, *P*_*HSL1*_ *‐CDC20 spo13‐10A* cells also degrade Rim4 (Fig 6B, right). While Rim4 degradation leads to Ama1 accumulation and Spo13 degradation, deletion of *CLB1*, inhibition of Cdk1, and the *spo13‐10A* mutation also cause Rim4 degradation in *P*_*HSL1*_ *‐CDC20 ama1Δ* cells (Appendix Fig S5C and D). This confirms that Cdk1‐Clb1 promotes not only the stability but also the activity of Spo13. We conclude that phosphorylation by Cdk1‐Clb1 is required for Spo13's ability to prevent Rim4 degradation and thereby *AMA1* translation at metaphase I (Fig 5C). Cdk1‐Clb1 might be assisted in this task by Cdk1‐Clb3 since Clb3 is translated together with Ama1. However, we found no evidence for such a mechanism: Deletion of *CLB3* does not affect Rim4 degradation, even in *clb1Δ* cells (Appendix Fig S5C).

### Cdk1‐Clb1 inhibits the function of Ama1 as an APC/C activator

In addition to repressing the translation of *AMA1*, Spo13 also inhibits the activity of the Ama1 protein (Fig EV1D). Similarly, Cdk1 can inhibit Ama1‐dependent proteolysis independently of its effect on Ama1 synthesis. For instance, expression of Clb1 in prophase‐arrested cells represses Ama1‐dependent proteolysis without affecting Ama1 levels (Okaz *et al*, 2012). This raises the question of how inhibition of Ama1 by Cdk1 is integrated with that by Spo13. Below, we first investigate the mechanism of Ama1's inhibition by Cdk1 and then turn to the role of Spo13.

**Figure 7:**
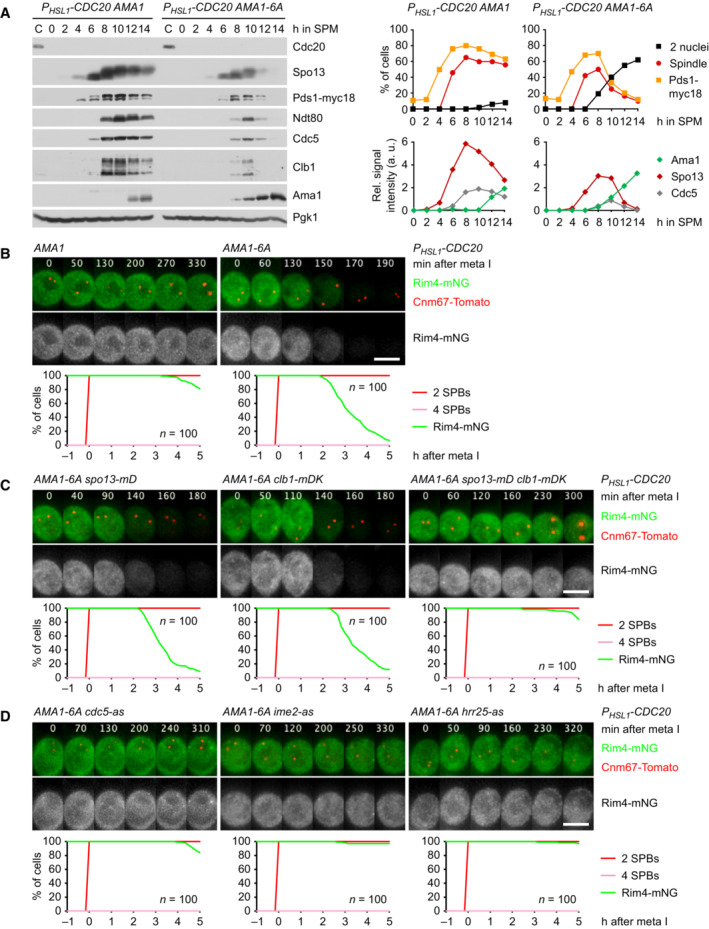
Analysis of *AMA1‐6A* mutants AAma1‐6A causes degradation of APC/C substrates and nuclear division in *P*_*HSL1*_ *‐CDC20* cells. Left, immunoblot detection of proteins. Top right, progression of meiosis quantified in fixed cells. Bottom right, relative signal intensities of proteins.B–DAnalysis of Rim4 degradation in *P*_*HSL1*_ *‐CDC20* cells carrying *AMA1‐6A*. Top, time‐lapse series from the filming of Rim4‐mNG and SPBs (Cnm67‐Tomato). Bottom, the presence of Rim4‐mNG quantified in cells synchronized *in silico* to SPB separation at metaphase I (*t* = 0). (B) Ama1‐6A causes Rim4 degradation. (C) Rim4 degradation is not affected by either *spo13‐mD* (*P* = 0.86) or *P*_*DMC1*_ *‐clb1‐mDK* (*P* = 0.20) alone, but is blocked in the presence of both alleles (*P* < 0.0001, Welch's *t*‐test). Cells in (B) and (C) were filmed together. (D) Rim4 degradation depends on the activities of Cdc5, Ime2, and Hrr25. Analogue‐sensitive kinases were inhibited at 8 h in SPM. Data information: Data are representative of four (B), two (C), or three (D) independent experiments. Scale bar, 4 μm. Source data are available online for this figure.

APC/C activators carry two motifs essential for binding to the APC/C: the C‐box in the N‐terminal region and the Ile‐Arg tail at the C‐terminus. Cdc20 and Cdh1 dissociate from the APC/C upon Cdk1‐dependent phosphorylation of multiple sites around the C‐box (Zachariae *et al*, 1998; Schwab *et al*, 2001; Labit *et al*, 2012; Chang *et al*, 2015). Thus, we mutated the six Ser/Thr‐Pro motifs in Ama1's N‐terminal region to non‐phosphorylatable Ala‐Pro. *AMA1‐6A* single mutants produce four spores (viability, 96%), implying that the mutation has little effect in otherwise normal cells. In *P*_*HSL1*_ *‐CDC20* cells, however, Ama1‐6A elicits degradation of APC/C substrates and nuclear division, while similar levels of Ama1 have little effect (Fig 7A). Thus, Ama1‐6A is a more potent activator than the wild‐type protein. Ama1‐6A also induces Rim4 degradation and therefore accumulates 2 h earlier than Ama1 (Fig 7A and B). Ama1‐6A mediates Rim4 degradation by targeting for proteolysis not only Spo13 but also Clb1; Rim4 is only stable in the presence of non‐degradable versions of both Spo13 and Clb1 (Fig 7C), which is consistent with the notion that Spo13 function requires Cdk1‐Clb1 activity. Rim4 degradation induced by Ama1‐6A depends on the activities of Cdc5, Ime2, and Hrr25 (Fig 7D), indicating that it requires signalling through the Cdc5‐Ime2‐Hrr25 kinase cascade. These data suggest that phosphorylation restrains the synthesis as well as the activity of Ama1 (Fig 5C).

**Figure EV4:**
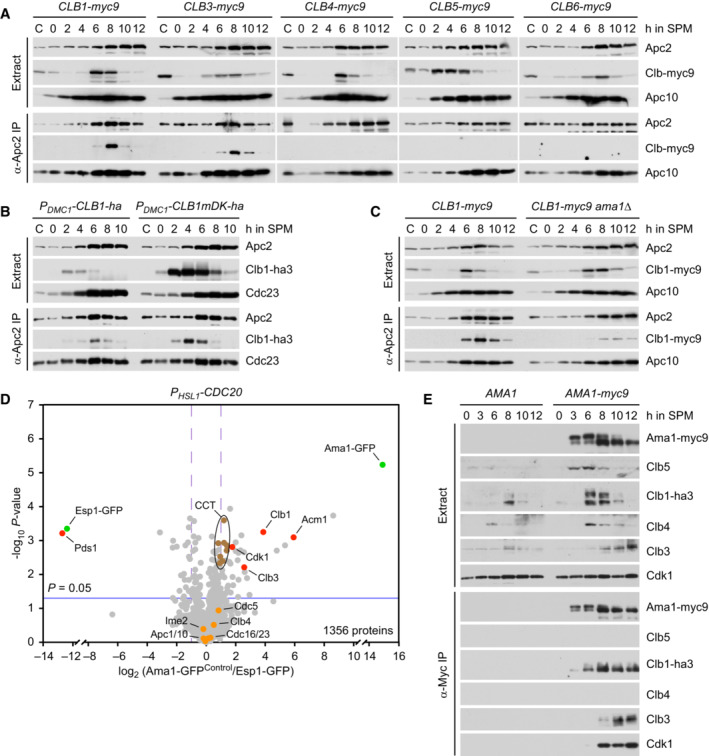
Identification of proteins copurifying with Ama1 A–CBinding of cyclins to the APC/C immunoprecipitated with α‐Apc2 antibodies. (A) Clb1 and Clb3 but not the other Clbs copurify with the APC/C. (B) The degrons of Clb1 are not required for binding to the APC/C. Ha3‐tagged Clb1 and Clb1‐mDK were expressed from the *DMC1* promoter. (C) Deletion of *AMA1* reduces Clb1's interaction with the APC/C.DProteins interacting with Ama1 at metaphase I. Ama1‐GFP and Esp1‐GFP (negative control) were purified from *P*_*HSL1*_ *‐CDC20* strains at 8 h in SPM, digested with trypsin and Lys‐C, and subjected to LC–MS/MS. ‐log_10_‐transformed *P*‐values and mean log_2_‐transformed label‐free quantifications of copurifying proteins were obtained from MaxQuant and displayed as a volcano plot. Baits (green), selected proteins with *P* < 0.05 (red) or *P* > 0.05 (orange), and the eight subunits of the CCT chaperonin (brown) are labeled. CCT is known to encapsulate and fold APC/C activators (Camasses *et al*, 2003). Only a subset of APC/C subunits is marked. Note that C‐terminally tagged Ama1 cannot bind the APC/C.EBinding of Clb1, Clb3, and Cdk1 to Ama1‐myc9 immunoprecipitated with α‐Myc antibodies. Data information: (D) is based on α‐GFP purifications from three independent cultures per strain. Source data are available online for this figure.

To investigate whether inhibition of APC/C^Ama1^ involves an interaction with CDKs, we immunoprecipitated the APC/C with antibodies to Apc2. Of the five B‐type cyclins expressed at meiosis (Clb1, 3, 4, 5, 6; Chu & Herskowitz, 1998), only Clb1 and Clb3 copurify with the APC/C (Fig EV4A). These interactions are unlikely to reflect substrate recognition by the APC/C because the degrons of Clb1 are dispensable for binding to the APC/C (Fig EV4B). On the other hand, deletion of *AMA1* reduces Clb1's association with the APC/C (Fig EV4C), suggesting that most of Clb1/3 copurifies with the APC/C via binding to Ama1. To investigate this, we purified Ama1‐GFP from extracts of Cdc20‐depleted cells. Ama1‐GFP does not bind the APC/C because C‐terminal tags prevent the Ile‐Arg tail from accessing its receptor (Vodermaier *et al*, 2003; Matyskiela & Morgan, 2009; Chang *et al*, 2015). Mass spectrometry identified Clb1, Clb3, and Cdk1 as high‐scoring interactors of Ama1‐GFP, whereas Clb4 and APC/C subunits show little or no binding (Fig EV4D). Specific binding of Cdk1‐Clb1/3 was also observed in immunoprecipitations of Ama1‐myc9 (Fig EV4E). These interactions do not depend on Spo13 (Appendix Fig S6A and B), raising the question of whether Spo13 is required for the ability of Cdk1‐Clb1 to inhibit Ama1. When expressed in prophase‐arrested *ndt80Δ* cells, Clb1 inhibits Ama1 in a Cdk1‐dependent manner, and cells produce M phase regulators under the control of the mitosis‐specific transcription factor Ndd1 (Okaz *et al*, 2012). Clb1 also inhibits Ama1 in *ndt80Δ spo13Δ* cells (Appendix Fig S6C), suggesting that Cdk1‐Clb1 can inhibit Ama1 independently of Spo13, provided that *AMA1* transcription is low (i.e., not induced by Ndt80). Despite binding to Ama1, Clb3 does not inhibit Ama1‐dependent proteolysis when expressed in *ndt80Δ* cells (Appendix Fig S6D). Accordingly, deletion of *CLB3* does not elicit nuclear division in Cdc20‐depleted cells (Oz *et al*, 2022). Ama1‐GFP also binds Acm1 (Fig EV4D), which has been shown to inhibit Cdh1 at mitosis (Dial *et al*, 2007; Enquist‐Newman *et al*, 2008). However, Acm1 does not play a role in suppressing Ama1's activity and synthesis at metaphase I since deletion of *ACM1* does not affect Rim4 stability in *P*_*HSL1*_ *‐CDC20* or *P*_*HSL1*_ *‐CDC20 AMA1‐6A* cells (Appendix Fig S6E). Taken together, our data suggest that Cdk1‐Clb1 employs two mechanisms to restrain Ama1‐dependent proteolysis at metaphase I (Fig 5C): First, by activating Spo13, it prevents the degradation of Rim4 and thereby the translation of *AMA1*. Second, by phosphorylating Ama1, it inhibits Ama1's activity. Consistent with this idea, APC/C only binds the unmodified form of Ama1 (Appendix Fig S2B). This brings back the question of how Spo13 inhibits the activity of Ama1.

### Inhibition of APC/C^Ama1^ requires phosphorylation of Clb1 by Cdc5‐Spo13

**Figure EV5:**
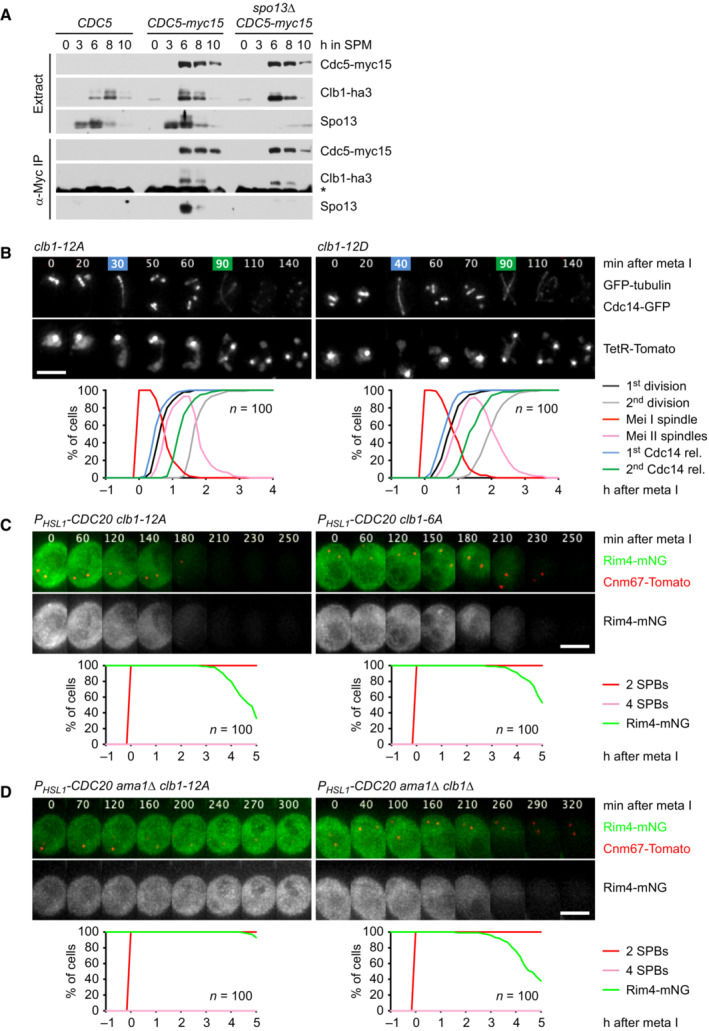
Analysis of Cdc5/Spo13‐dependent phosphorylation of Clb1 Clb1‐ha3 binds to Cdc15‐myc15 in α‐Myc immunoprecipitations from extracts of control and *spo13Δ* cells.Meiosis in *clb1‐12A* and *clb1‐12D* cells. Top, time lapse series from the imaging of spindles (GFP‐tubulin), nucleolar release of Cdc14‐GFP, and TetR‐Tomato, which labels the nucleoplasm (diffuse signal) and the centromeres of one copy of chromosome V (dots). First (blue) and second (green) Cdc14 release are marked. Bottom, meiotic events quantified in cells synchronized *in silico* to spindle formation at metaphase I (*t* = 0). The duration of metaphase I is similar in *clb1‐12A* and *clb1‐12D* cells (*P* = 0.33).
*P*_*HSL1*_ *‐CDC20 clb1‐12A* cells degrade Rim4 earlier than *P*_*HSL1*_ *‐CDC20 clb1‐6A* cells (*P* < 0.0001). Top, time‐lapse series from the imaging of Rim4‐mNG and SPBs (Cnm67‐Tomato). Bottom, the presence of Rim4‐mNG quantified in cells synchronized *in silico* to SPB separation at metaphase I (*t* = 0).Clb1 phosphorylation is not required for the activation of Spo13. Top, time‐lapse series from the imaging of Rim4‐mNG and SPBs (Cnm67‐Tomato) in *P*_*HSL1*_ *‐CDC20 ama1Δ* cells carrying *clb1‐12A* or *clb1Δ*. Bottom, the presence of Rim4‐mNG quantified in cells synchronized *in silico* to SPB separation at metaphase I (*t* = 0). Data information: Data are representative of two (B and C) or three (D) independent experiments. Means were compared using Welch's *t*‐test. Scale bar, 4 μm. Source data are available online for this figure.

While Cdk1‐Clb1 inhibits the synthesis and activity of Ama1, Clb1 is also a substrate of APC/C^Ama1^ (Okaz *et al*, 2012; Arguello‐Miranda *et al*, 2017). This constitutes a double‐negative feedback loop, which generates two, mutually exclusive states: a state of low APC/C^Ama1^ and high Cdk1 activity that is characteristic of metaphase I and a state of high APC/C^Ama1^ and low Cdk1 activity, which resembles anaphase II. The low‐Ama1/high‐Cdk1 state could be stabilized by rendering Clb1 less susceptible to Ama1‐dependent proteolysis (Fig 5C). It is noteworthy, therefore, that Clb1 shows multiple bands of reduced electrophoretic mobility at metaphase I but not at metaphase II or mitosis (Attner *et al*, 2013; Tibbles *et al*, 2013). This modification correlates with the levels of Spo13 since it is enhanced and persists longer in *spo13‐mD* cells (Fig 2B). Indeed, Clb1's modification depends on Spo13 (Fig 2A and C), the binding of Spo13 to Cdc5 (Fig EV2B), and the catalytic activity of Cdc5 (Fig 5A). It also requires the activity of Cdk1, the kinase that activates Spo13 (Fig 6C). Remarkably, Cdc5 still binds but cannot modify Clb1 in the absence of Spo13 (Fig EV5A). We conclude that the Cdc5‐Spo13 complex is an active kinase that phosphorylates Clb1.

**Figure 8:**
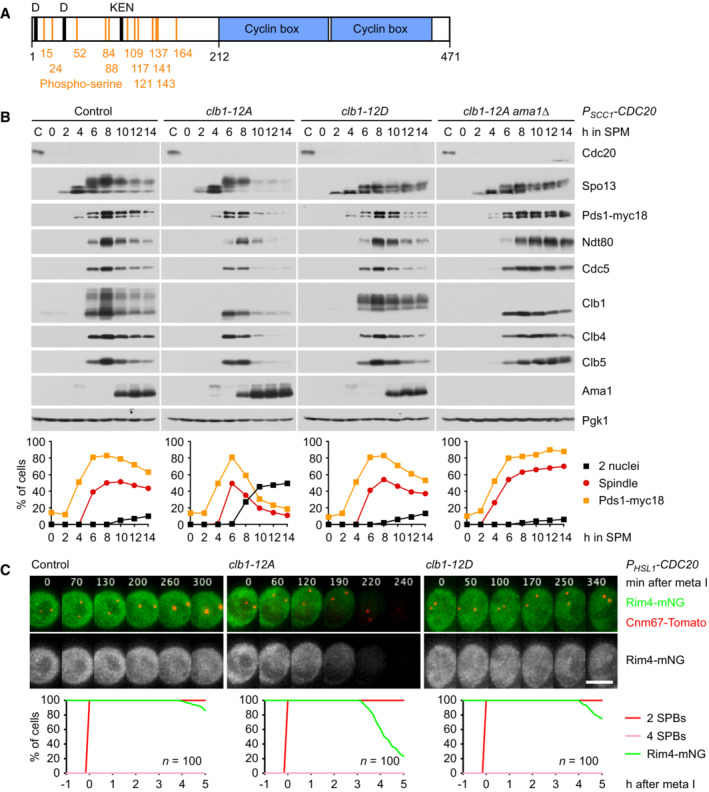
Analysis of *CLB1* phosphorylation site mutants Domain structure of Clb1 showing D‐ and KEN‐boxes (black), phospho‐serines (orange), and cyclin boxes (blue).
*clb1‐12A* but not *clb1‐12D* causes Ama1‐dependent proteolysis and nuclear division in Cdc20‐depleted cells. Top, immunoblot detection of proteins. Bottom, progression of meiosis quantified in fixed cells.Imaging of Rim4‐mNG and SPBs (Cnm67‐Tomato) in *P*_*HSL1*_ *‐CDC20* control cells and cells carrying *clb1‐12A* or *clb1‐12D*. Top, time‐lapse series. Bottom, the presence of Rim4‐mNG quantified in cells synchronized *in silico* to SPB separation at metaphase I (*t* = 0). Data information: (C) is representative of three independent experiments. Scale bar, 4 μm. Source data are available online for this figure.

To identify phosphorylation sites, we purified GFP‐tagged Clb1 from *P*_*SCC1*_ *‐CDC20 ama1Δ* strains arrested at metaphase I (Appendix Fig S7A). Mass spectrometry revealed phosphorylation of eight serines in Clb1‐GFP from control cells. These phosphorylation sites were not detected or only at low intensity in Clb1‐GFP from cells lacking Spo13 or Cdc5, although the amounts of Clb1‐GFP were similar to those obtained from control cells (Appendix Fig S7B–D). Seven of these serines and four additional serines were also found to be phosphorylated in Clb1‐GFP purified from *P*_*EST*_ *‐NDT80* cells progressing into metaphase I (Appendix Fig S8A). Phosphorylation site intensities are strongly reduced in Clb1‐GFP from *spo13Δ* cells at metaphase I and control cells at metaphase II (Appendix Fig S8B–D). The 12 phospho‐serines are located in Clb1's N‐terminal, intrinsically disordered region, but not the conserved cyclin‐box domain (Fig 8A; Varadi *et al*, 2022). The N‐terminal region contains 22 serines and 10 threonines (Appendix Fig S8E), indicating that at least in Clb1, Cdc5‐Spo13 prefers serine to threonine. We mutated the phosphorylated serines to either alanine or aspartate to generate non‐phosphorylatable Clb1‐12A and phospho‐mimicking Clb1‐12D, respectively. *clb1‐12A* and *clb1‐12D* mutants markedly differ from *clb1Δ* cells in that they undergo two divisions and produce four spores with high viability (99%; Fig EV5B). This suggests that Clb1‐12A and Clb1‐12D are capable of activating Cdk1.

Next, we introduced *clb1‐12A* and *clb1‐12D* into *P*_*HSL1*_ *‐CDC20* cells. Immunoblotting revealed that mutating these serines either eliminates (Clb1‐12A) or strongly reduces (Clb1‐12D) the modification of Clb1, confirming that phosphorylation is confined to Clb1's N‐terminal region (Fig 8B). However, Clb1‐12A and Clb1‐12D show strikingly different properties: Clb1‐12A is more sensitive to APC/C^Ama1^ activity than wild‐type Clb1, being degraded as soon as Ama1 appears. As a result, *P*_*HSL1*_ *‐CDC20 clb1‐12A* cells degrade APC/C substrates and undergo nuclear division in an Ama1‐dependent manner (Fig 8B). Furthermore, these cells degrade Rim4, which in turn advances the accumulation of Ama1 (Fig 8C). Thus, Ama1's ability to target Clb1‐12A for proteolysis unleashes feedback loops, which cause Ama1 to increase its own activity and synthesis through the degradation of Clb1 and Spo13 (Fig 5C). We also analyzed *clb1‐6A* in which only six high‐confidence phosphorylation sites are replaced by alanine (the first six sites in Appendix Fig S7C). *P*_*HSL1*_ *‐CDC20 clb1‐6A* cells degrade Rim4 more slowly than cells carrying *clb1‐12A* (Fig EV5C), indicating that most, if not all, of the 12 phospho‐serines contribute to suppressing Ama1's activity and synthesis. Indeed, phospho‐mimetic Clb1‐12D persists in the presence of Ama1, similar to wild‐type Clb1 (Fig 8B). Clb1‐12D is therefore able to inhibit Ama1‐dependent proteolysis, so that APC/C substrates persist and nuclear division is blocked. *P*_*HSL1*_ *‐CDC20 clb1‐12D* cells also maintain high levels of Rim4, which prevents premature synthesis of Ama1 (Fig 8C). These data suggest that phosphorylation of Clb1 by Cdc5‐Spo13 restrains Ama1's ability to increase its own activity and synthesis at metaphase I (Fig 5C). Phosphorylation of Clb1 might enable Cdk1‐Clb1 to inhibit Ama1's function as an APC/C activator or it might allow Cdk1‐Clb1 to activate Spo13. To test the latter possibility, we imaged Rim4‐mNG in *P*_*HSL1*_ *‐CDC20 ama1Δ* strains (Fig EV5D). Rim4 persists in cells carrying *clb1‐12A* but is degraded in the *clb1Δ* mutant, suggesting that activation of Spo13 requires Cdk1‐Clb1 activity but not the phosphorylation of Clb1. This finding indicates that phosphorylation enables Clb1 to inhibit Ama1's activity, which in turn stabilizes not only Clb1 but also Spo13.

### Phosphorylation of Clb1 reduces its Ama1‐dependent degradation

**Figure 9:**
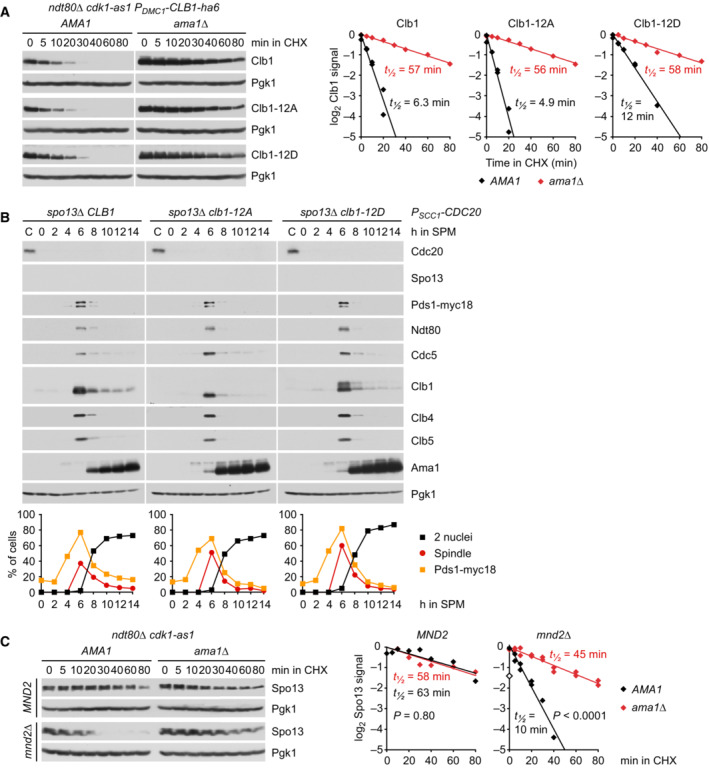
Protection of Clb1 and Spo13 from Ama1‐dependent proteolysis Stability of Clb1, Clb1‐12A, and Clb1‐12D. *CLB1* alleles were expressed from the *DMC1* promoter in *ndt80Δ cdk1‐as1* strains carrying *AMA1* or *ama1Δ*. Strains were arrested at prophase in the presence of 1NM‐PP1 to inhibit Cdk1‐as1. At 6 h in SPM, translation was inhibited with cycloheximide (CHX, *t* = 0). Left, immunoblot detection of Clb1. Right, log_2_‐transformed Clb1 signal intensities (normalized to *t* = 0) plotted over time in CHX. Half‐lives were calculated by linear regression.Analysis of APC/C substrates in *P*_*SCC1*_ *‐CDC20* cells carrying *spo13Δ* and *CLB1*, *clb1‐12A*, or *clb1‐12D*. Top, immunoblot detection of proteins. Bottom, progression of meiosis quantified in fixed cells.Spo13 stability at prophase. *ndt80Δ cdk1‐as1* strains carrying *AMA1* or *ama1Δ* were arrested at prophase in the presence of 1NM‐PP1 to inhibit Cdk1‐as1. At 6 h in SPM, translation was inhibited with CHX (*t* = 0). *mnd2Δ* cells expressing Spo13 from the *DMC1* promoter were processed in parallel. Left, immunoblot detection of Spo13. Right, log_2_‐transformed Spo13 signal intensities (normalized to *t* = 0) plotted over time in CHX. Half‐lives were calculated by linear regression. Open diamond, data point omitted from regression. Data information: (A) *AMA1* data are from two independent experiments. *R* ^2^ values of *AMA1* and *ama1Δ* regression lines: Clb1, 0.92 and 0.99; Clb1‐12A, 0.95 and 0.99; Clb1‐12D, 0.99 and 0.97. (C) *mnd2Δ* data are from two independent experiments. *R* ^2^ values of *AMA1* and *ama1Δ* regression lines: *MND2*, 0.75 and 0.84; *mnd2Δ*, 0.98 and 0.95. Slopes were compared using ANCOVA. Source data are available online for this figure.

Our data imply that phosphorylation, or the phospho‐mimicking mutations, protects Clb1 from Ama1‐dependent proteolysis. To investigate the extent of this protection, we arrested *ndt80Δ cdk1‐as1* strains at prophase and added 1NM‐PP1 to inhibit Cdk1. Under these conditions, APC/C^Ama1^ is active towards Clb1 and other M phase regulators (Okaz *et al*, 2012). We expressed Clb1 from the early meiosis‐specific *DMC1* promoter and followed its degradation upon inhibition of translation by cycloheximide (Fig 9A). Wild‐type Clb1 and Clb1‐12A are degraded with a half‐life of 5–6 min (*P* = 0.14). Thus, at prophase, when Cdc5‐Spo13 is absent, wild‐type Clb1 behaves similarly to Clb1‐12A. However, Clb1‐12D is degraded more slowly, showing a half‐life of 12 min (*P* < 0.001). Upon deletion of *AMA1*, all Clb1 variants are stabilized, displaying half‐lives of ~57 min. These data suggest that phosphorylation of Clb1 by Cdc5‐Spo13 results in a 2.5‐fold reduction in Clb1's degradation rate. While modest, the increase in Clb1's stability supports feedback loops that reinforce the stability and phosphorylation of Spo13 and thereby inhibit the synthesis as well as the activity of Ama1 (Fig 5C). Accordingly, deletion of *SPO13* advances the accumulation of Ama1 in *P*_*HSL1*_ *‐CDC20 clb1‐12D* cells, resulting in the degradation of APC/C substrates and nuclear division (Fig 9B). In fact, *spo13Δ* cells degrade Clb1‐12D as fast as Clb1‐12A, suggesting that the phospho‐mimicking mutations provide little protection from Ama1‐dependent degradation once Ama1 synthesis has been induced (Fig 9B). Taken together, our data show that mutual phosphorylation of Cdc5‐Spo13 and Cdk1‐Clb1 integrates and amplifies the pathways that repress Ama1 synthesis and activity at metaphase I (Fig 5C).

### Restoring a two‐division meiosis in *spo13Δ* mutants

Deletion of *AMA1* might be expected to restore a second division in *spo13Δ* cells, which implies, however, that Ama1 differs from Cdc20 by its regulation but not its range of substrates. This is not the case: *spo13Δ ama1Δ* cells undergo a single division (Appendix Fig S9A). Indeed, deletion of *AMA1* causes accumulation of Ndt80, which results in higher levels of M phase regulators, including Cdc5 and Cdc20 (Okaz *et al*, 2012; Appendix Fig S9C). This might increase the activity of APC/C^Cdc20^, in particular after a prolonged metaphase I. While APC/C^Cdc20^ mediates a division in *spo13Δ ama1Δ* cells, it might subsequently prevent a second one. To test this idea, we tagged Ndt80 with AID but found that the tag reduces Ndt80 levels about tenfold, even in the absence of OsTir1 (Appendix Fig S9D). While Ndt80‐AID has little effect in otherwise normal cells, it causes 70% of *spo13Δ ama1Δ* cells to undergo two divisions (Appendix Fig S9B). Ndt80‐AID also restores two divisions in 50% of *spo13Δ* cells, probably because it reduces the activity of APC/C^Cdc20^ as well as APC/C^Ama1^. Thus, a two‐division meiosis depends on the mutual regulation between APC/C^Cdc20^ and APC/C^Ama1^ via Spo13 and Ndt80.

### Mnd2 protects Spo13 from Ama1‐dependent degradation at prophase

At entry into metaphase I, Ndt80 induces the transcription of *AMA1* and *CDC5* (Fig 2C). To suppress the translation of *AMA1* mRNA, Cdc5‐Spo13 must prevent free Cdc5 from inducing the degradation of Rim4. Indeed, Spo13 accumulates at prophase, that is, before Cdc5 (Fig 2A; Matos *et al*, 2008). However, APC/C^Ama1^ is active, albeit at low levels, at this stage of meiosis (Okaz *et al*, 2012). Similar to Pds1 and Clb5, Spo13 might be protected from Ama1‐dependent proteolysis by the inhibitory APC/C subunit Mnd2 (Oelschlaegel *et al*, 2005; Penkner *et al*, 2005). To test this idea, we measured Spo13's stability in prophase‐arrested cells. In cells containing Mnd2, Spo13 shows a half‐life of ~60 min in the presence and absence of Ama1 (Fig 9C). In *mnd2Δ* cells, however, Spo13 is targeted for degradation by Ama1 (half‐life, 10 min). Accordingly, Mnd2 is required for normal levels of Spo13 at prophase (Appendix Fig S10). By protecting Spo13, but not Cdc5, from Ama1‐dependent proteolysis at prophase, Mnd2 ensures that Cdc5‐Spo13 is formed as soon as Cdc5 appears at entry into metaphase I.

## Discussion

Meiosis I and ‐II must have different outcomes in order to achieve genome haploidization and gamete differentiation. For instance, exit from meiosis must be initiated at meiosis II to generate a two‐division meiosis. However, mRNAs for cell cycle regulators and differentiation factors are produced by a transcriptional program shortly before the first division. This raises the question of how proteins involved in meiosis II‐specific events are translated or activated not at meiosis I but at meiosis II. We argue that the *spo13* mutant is key to answering this question because it fails to repress meiosis II‐specific events at meiosis I. Indeed, *spo13* cells perform only one division because they prematurely activate the meiotic exit machinery.

### The *spo13* mutant fails to repress meiosis II‐specific processes at meiosis I

The *spo13* mutant was among the first single‐division mutants to be discovered more than 40 years ago (Klapholz & Esposito, 1980a). It has attracted attention mainly because defects in sister kinetochore mono‐orientation and centromeric cohesin protection suggest that Spo13 coordinates centromere‐related processes unique to meiosis I (Katis *et al*, 2004; Lee *et al*, 2004; Matos *et al*, 2008). However, disabling these processes does not result in a single‐division meiosis (Toth *et al*, 2000; Rabitsch *et al*, 2003), suggesting that Spo13 has additional functions. A clue to these functions is the observation that despite of undergoing a single wave of Cdk1 and APC/C activity, *spo13Δ* cells show aspects of meiosis I and ‐II. For instance, the *spo13Δ*‐division is preceded by recombination, a process associated with meiosis I, but concluded by exit from meiosis and spore formation, which are meiosis II‐specific processes (Klapholz & Esposito, 1980b). We show that *spo13Δ* cells prematurely degrade the translational repressor Rim4 (Fig 3) and therefore synthesize meiosis II‐specific proteins, including Ama1 and sporulation factors (Figs 2 and EV1). *spo13Δ* cells also fail to produce phosphorylated Clb1, an inhibitor of Ama1 activity (Figs 2 and 8). Thus, activation of APC/C^Ama1^ at metaphase I causes *spo13Δ* cells to exit from meiosis after a single division. Furthermore, *spo13Δ* cells prematurely degrade Spc72, the repressor of meiotic plaque (MP) assembly, which, together with the translation of sporulation factors, leads to spore formation at meiosis I (Oz *et al*, 2022). A major defect of *spo13Δ* cells is therefore the inability to repress meiosis II‐specific events at meiosis I.

This hypothesis can also be applied to chromosome segregation. Sister kinetochore orientation is governed by two processes: Monopolin's loading onto kinetochores at prophase is required for mono‐orientation at meiosis I, while its removal at the onset of metaphase II allows bi‐orientation at meiosis II (Toth *et al*, 2000). *spo13Δ* cells fail to retain monopolin at kinetochores at metaphase I (Katis *et al*, 2004; Lee *et al*, 2004), which can be interpreted as a failure to repress the removal of monopolin. Likewise, the stepwise cleavage of cohesin depends on two processes: the protection of centromeric cohesin by Sgo1 at anaphase I and its de‐protection through APC/C‐dependent removal of Sgo1 from centromeres at anaphase II (Riedel *et al*, 2006; Mengoli *et al*, 2021). *spo13Δ* cells loose Sgo1 from centromeres at metaphase I (Katis *et al*, 2004; Lee *et al*, 2004), which can be viewed as a defect in repressing the removal of Sgo1. Therefore, most if not all phenotypes of the *spo13Δ* mutant can be explained by a single, testable hypothesis, namely premature inactivation of mechanisms that repress meiosis II‐specific events at meiosis I.

A striking feature of the *spo13Δ* phenotype is its dependence on the duration of metaphase I. *spo13Δ* cells undergo two divisions when metaphase I is short but a single division when metaphase I is prolonged due to inhibition of Cdc20. In *spo13Δ* cells, Cdc20 is inhibited by the SAC. The SAC might respond to bivalent chromosomes that cannot balance spindle forces because they carry a bioriented and a mono‐oriented pair of sister kinetochores (Nerusheva *et al*, 2014). In *clb1Δ* mutants, Spo13 is largely inactive, while activation of APC/C^Cdc20^ is compromised. Thus, the single‐division meiosis of *spo13* mutants results from two conditions: reduced Spo13 function and delayed APC/C^Cdc20^ activation. The prolonged metaphase I of *spo13Δ* cells (66 min) allows meiosis II‐specific events to unfold. By contrast, metaphase I is very short in *spo13Δ mad2Δ* cells (10 min). While ‘quick’ meiosis II‐events might still occur at meiosis I, more elaborate processes, such as Ama1 accumulation or spore formation, are only completed by the time these cells have progressed into meiosis II. Therefore, *spo13Δ mad2Δ* cells exit from meiosis and sporulate after the second division (Shonn *et al*, 2002). The time‐dependence of the *spo13* phenotype highlights a problem unique to meiotic cells: To ensure a two‐division meiosis, SAC activity at metaphase I has to halt not only entry into anaphase I but also meiosis II‐specific events. This is achieved by Spo13, a meiosis I‐specific substrate of APC/C^Cdc20^. Upon activation of the SAC at meiosis I, Spo13 is stabilized, which in turn prevents the inactivation of repressors of meiosis II‐specific events.

### Ama1: a Cdh1‐related APC/C activator tailored to the regulation of meiosis

In yeast, exit from meiosis involves the APC/C activator Ama1, which is related to the G1‐specific activator Cdh1 but shows features tailored to meiotic cell cycle control. At prophase, low levels of Ama1 suppress the expression of M phase regulators by the mitosis‐specific transcription factor Ndd1, which renders entry into metaphase I dependent on Ndt80 and the completion of recombination (Okaz *et al*, 2012). At meiosis II, Ama1 accumulates to high levels and promotes exit from meiosis through the degradation of M phase regulators, such as Cdc5, B‐type cyclins, and Ndt80. Similar to Cdh1, Ama1 is phosphorylated at Cdk1 sites around the C‐box, which prevents binding of the activator to the APC/C. While Cdh1 is inhibited by different Cdk1‐Clb kinases and Ime2 (Zachariae *et al*, 1998; Jaspersen *et al*, 1999; Bolte *et al*, 2002; Holt *et al*, 2007), Ama1 is specifically inhibited by Cdk1‐Clb1, which allows Ama1 to be active at prophase. The mutual inhibition between Cdk1‐Clb1 and Ama1, known as a double‐negative feedback loop, results in two distinct states: a state of high Cdk1‐Clb1 and low Ama1 activity and a state where the reverse is true (Okaz *et al*, 2012). Entry into metaphase I requires conditions that enable Cdk1‐Clb1 to inhibit APC/C^Ama1^, which entails induction of *CLB1* transcription by Ndt80 when Ama1 levels are low. Conversely, the transition from the high‐ to the low‐Cdk1 state is initiated by Cdc20‐dependent proteolysis of Clb1 and an increase in Ama1 synthesis. A two‐division meiosis depends on this transition occurring at meiosis II but not at meiosis I. While *AMA1* transcription is induced by Ndt80 (Chu *et al*, 1998; Primig *et al*, 2000), its translation is repressed at metaphase I by Rim4 (Fig 3A). However, translational repression becomes increasingly leaky because *AMA1* mRNA accumulates throughout metaphase I (Fig 2C). The appearance of Ama1 poses a threat to the high‐Cdk1 state of metaphase I since APC/C^Ama1^ eventually gains the ability to overwhelm its inhibitor Cdk1‐Clb1, resulting in premature exit from meiosis I. To stabilize metaphase I and allow its prolongation by the SAC, meiotic cells rely on the coordinated repression of Ama1 synthesis and activity by Spo13 (Fig 5C).

### Spo13: activator or inhibitor of Cdc5/PLK1?

We show that Spo13 prevents the Ime2 kinase from promoting Rim4 degradation and thereby *AMA1* translation at metaphase I. While Spo13's function depends on its ability to bind Cdc5, inhibition of Cdc5 activity blocks rather than elicits Rim4 degradation (Fig EV2C). At first glance, this result seems to imply that Spo13 inhibits Cdc5. We find, however, that Cdc5 exists in two forms at metaphase I, free Cdc5 and Cdc5 bound to Spo13, whereby the level of free Cdc5 greatly exceeds that of Cdc5‐Spo13 (Fig EV2D; Oz *et al*, 2022). Analysis of *spo13Δ* mutants shows that free Cdc5 is required for the ability of Ime2 to promote Rim4 degradation and Ama1 accumulation. Accordingly, Cdc5 binds and phosphorylates Ime2 independently of Spo13 (Oz *et al*, 2022; Fig 5A). The function of Cdc5‐Spo13 as an active kinase was revealed through the identification of a substrate, namely Clb1 (Appendix Figs S7 and S8). Phosphorylation of Clb1 depends on Spo13, Spo13's binding to Cdc5, and the kinase activity of Cdc5. Remarkably, Cdc5 binds, but cannot phosphorylate, Clb1 in the absence of Spo13. Thus, Clb1 is a substrate of the Cdc5‐Spo13 kinase but not of free Cdc5. Ime2 promotes another meiosis II‐specific process, namely the degradation of Spc72, which is a prerequisite for MP assembly and spore formation (Knop & Strasser, 2000; Oz *et al*, 2022). Ime2 activity requires Cdc5 and is inhibited by Spo13 in a manner dependent on its binding to Cdc5. Were it not for sub‐stoichiometric amounts of Spo13, it could be argued, again, that Spo13 inhibits Cdc5. However, MP assembly is also inhibited by a second, Spc72‐independent pathway, which requires Cdc5 activity as well as Spo13 and therefore depends on an active Cdc5‐Spo13 kinase (Oz *et al*, 2022). Thus, a simple model posits that Ime2 is inhibited by Cdc5‐Spo13 and activated by free Cdc5 (Fig 5C). At metaphase I, inhibition by Cdc5‐Spo13 dominates over activation by free Cdc5. At anaphase I, the activating function of free Cdc5 is unleashed by the degradation of Spo13. This model for the regulation of Ime2 is reminiscent of the phospho‐regulation of Cdk1‐cyclin B. Cdk1 is subject to inhibitory phosphorylation by the Wee1 kinase and activating phosphorylation by Cdk1‐activating kinase (CAK; Perry & Kornbluth, 2007). Cdk1 is inhibited until the inhibitory phosphorylation is removed upon activation of the Cdc25 phosphatase and the inactivation of Wee1.

How does Cdc5‐Spo13 recognizes its substrates? Cdc5/PLK1 binds to a phosphopeptide motif within the substrate via its C‐terminal PBD, which releases an autoinhibitory interaction between kinase domain and PBD (Elia *et al*, 2003a, 2003b; Xu *et al*, 2013). Interestingly, Spo13 itself binds to the PBD (Matos *et al*, 2008), which blocks the recognition of conventional substrates. However, recent work has identified a surface on the back of the PBD that binds substrates even when the phosphopeptide‐binding groove is occupied (Chen & Weinreich, 2010; Almawi *et al*, 2020). This interaction is inhibited rather than promoted by phosphorylation. Thus, Spo13 might both activate Cdc5 and enforce the unconventional mode of substrate recognition. Spo13 might also alter the specificity of Cdc5‐dependent phosphorylation. Cdc5‐Spo13 phosphorylates at least 12 out of 22 serines but none of the 10 threonines in Clb1's N‐terminal region (Appendix Figs S7 and S8). This suggests that at least on Clb1, Cdc5‐Spo13 prefers serine to threonine, whereas Cdc5/PLK1 phosphorylates both residues (Mok *et al*, 2010; Santamaria *et al*, 2011). However, more substrates need to be identified to establish the consensus motif for phosphorylation by the Cdc5‐Spo13 kinase. Another question concerns the phosphatases that remove Cdc5/Spo13‐dependent phosphorylation when Spo13 is degraded at anaphase I. It is conceivable that Cdc5‐Spo13 confines the activity of such phosphatases to meiosis II. We note that Cdc5‐Spo13 represses the translation of *GIP1* (Fig EV1B), which encodes a meiosis‐specific targeting subunit of the Glc7/PP1 phosphatase involved in spore formation (Tachikawa *et al*, 2001).

### Regulation of Rim4 degradation by Cdc5, Ime2, and Hrr25

Our data suggest that free Cdc5 promotes whereas Cdc5‐Spo13 inhibits Ime2's function in Rim4 degradation. Accordingly, hyperactive Ime2‐ΔC induces Rim4 degradation even when Cdc5 is inhibited. However, Ime2‐ΔC also induces Rim4 degradation in *cdc20 ama1Δ* cells, that is, in cells containing stable Spo13. We propose that both free Cdc5 and Cdc5‐Spo13 control Ime2 activity by targeting its C‐terminal regulatory domain. How does Ime2 promote Rim4 degradation? Interestingly, Rim4 inhibits the translation of mRNAs while adopting an amyloid‐like structure (Berchowitz *et al*, 2015). Furthermore, Rim4 is phosphorylated on more than 30 sites in an Ime2‐dependent manner. It has been proposed that this phosphorylation dissolves the amyloid‐like structure, thereby releasing mRNAs for translation and subjecting soluble Rim4 to ubiquitinylation and proteasomal degradation (Carpenter *et al*, 2018). However, we also show that Hrr25 functions downstream of Ime2 in promoting the degradation of Rim4. The ability of Ime2‐ΔC to induce Rim4 degradation is blocked upon inhibition of Hrr25, whereas hyperactive Hrr25‐ΔC induces Rim4 degradation even when Ime2 is inhibited (Fig EV3). Formally, our data are consistent with a linear Cdc5‐Ime2‐Hrr25 kinase cascade, whereby Hrr25, rather than Ime2, elicits the degradation of Rim4 (Fig 5C). In this scenario, Ime2 might activate Hrr25 by targeting the C‐terminal, autoinhibitory domain of Hrr25. Indeed, Ime2 and Rim4 co‐immunoprecipitate with Hrr25 (Fig EV3D). The idea that Cdc5‐Spo13 inhibits signalling through the Cdc5‐Ime2‐Hrr25 cascade at metaphase I is consistent with the observation that Ime2 activity increases as cells enter meiosis II (Berchowitz *et al*, 2013). It might also explain why Hrr25 promotes events, such as MP assembly or Sgo1's removal from centromeres, at meiosis II but not at meiosis I (Arguello‐Miranda *et al*, 2017).

Recent work suggests another, mutually not exclusive scenario whereby Rim4 degradation and exit from meiosis depend on autophagy, a proteolytic system capable of removing large aggregates and organelles from the cytosol (Farre & Subramani, 2016; Wang *et al*, 2020; Herod *et al*, 2022). Indeed, Hrr25 is required for different pathways of selective autophagy (Mochida *et al*, 2014; Pfaffenwimmer *et al*, 2014; Tanaka *et al*, 2014; Meguro *et al*, 2020). Hrr25 phosphorylates autophagy receptors to promote their binding to Atg11, the organizer of the PAS. A simple hypothesis is that upon activation by Ime2, Hrr25 induces several pathways of selective autophagy, one of which mediates the degradation of Rim4. In this scenario, mRNAs to be translated must be released from Rim4 before its enclosure by autophagosomes. This release might be elicited by Hrr25 since Hrr25‐ΔC is capable of inducing *AMA1* translation at metaphase I (Appendix Fig S3C).

Hrr25's prominent role in autophagy could explain a perplexing change in the regulation of Rim4 degradation and Ama1 accumulation: On the one hand, Rim4 degradation elicited by the *SPO13* deletion or the *AMA1‐6A* mutation depends on Cdc5 and Ime2 activity. On the other hand, Ama1 accumulation results in degradation of Cdc5 and inactivation of Ime2 (Figs 2A and 7A). While initially dependent on Cdc5 and Ime2, Rim4 degradation and Ama1 accumulation become independent of these kinases at some time after the inactivation of Spo13. Accordingly, inhibition of Cdc5 or Ime2 at late anaphase I has no effect on subsequent Ama1 accumulation at meiosis II. By contrast, Hrr25 remains essential for Rim4 degradation and Ama1 accumulation (Appendix Fig S4; Arguello‐Miranda *et al*, 2017). This is indicative of an irreversible transition whereby Hrr25‐dependent removal of Rim4 becomes self‐sustaining. An important step in the induction of selective autophagy is binding of Hrr25 to the Rab GTPase Ypt1/Rab1, which activates Hrr25 and recruits it to Atg11 (Wang *et al*, 2015). Once activated at the PAS, Hrr25 might sustain autophagic degradation of Rim4 independently of Cdc5 and Ime2.

### Regulation of Ama1 activity by Cdc5‐Spo13

Spo13 also employs a post‐translational mechanism to inhibit Ama1‐dependent proteolysis (Fig EV1D). We show that in essence, Cdc5‐Spo13 converts Cdk1‐Clb1 from a target into an inhibitor of Ama1. Cdk1‐Clb1 binds to free as well as APC/C‐bound Ama1 (Fig EV4). This interaction does not require the degrons of Clb1 but might be mediated by its hydrophobic patch, a surface involved in targeting Cdk‐cyclin complexes to their substrates (Ord *et al*, 2019). In addition, phosphorylation of Ama1's N‐terminal Thr‐Pro motif might create a docking site for Cks1, the phospho‐adaptor subunit of CDKs, thereby facilitating the phosphorylation of downstream Ser‐Pro motifs (Koivomagi *et al*, 2013; McGrath *et al*, 2013). We propose that Cdk1‐Clb1 bound to Ama1 has two alternative fates: Clb1 can be ubiquitinylated, which requires binding of Ama1 to the APC/C. Alternatively, Cdk1‐Clb1 phosphorylates Ama1, which prevents Ama1's binding to the APC/C and thereby ubiquitinylation of Clb1. By phosphorylating Clb1, Cdc5‐Spo13 shifts the balance from Clb1 ubiquitinylation towards Ama1 phosphorylation. Multisite phosphorylation of Clb1's N‐terminal region might hinder ubiquitinylation by obstructing ubiquitin‐accepting lysine residues, impeding the binding of D‐ and KEN‐boxes to their receptors, or reducing Ama1's affinity for the APC/C. Cellular polyanions, such as polyphosphate, weaken the activator‐APC/C interaction (Mizrak & Morgan, 2019). While conventional substrates can revert this effect, phosphorylated Clb1 is covalently linked to a polyanion. Phosphorylation causes a modest reduction in Clb1's degradation rate (Fig 9A), which is, however, amplified by its effects on Ama1 activity and synthesis (Fig 5C). In this way, phosphorylated Clb1 can inhibit Ama1 but still be targeted by Cdc20. Phosphorylation also inhibits degradation of the APC/C substrates Pds1, Cdc6, and KIF1C (Wang *et al*, 2001; Mailand & Diffley, 2005; Singh *et al*, 2014).

### Integration of APC/C^Ama1^ regulation with the Cdk1‐APC/C^Cdc20^ oscillator

Progression through the two M phases of meiosis might be divided into three phases: entry into and stabilization of metaphase I, transition from meiosis I to ‐II, and exit from meiosis. At entry into metaphase I, Cdc5‐Spo13 and Cdk1‐Clb1 repress the activity of APC/C^Ama1^ despite being susceptible to Ama1‐dependent proteolysis. While the synthesis of Clb1 and Cdc5 depends on Ndt80, Spo13 accumulates already at prophase. This is possible because Spo13, like Pds1, is protected from Ama1‐dependent proteolysis at prophase by the APC/C subunit Mnd2 (Fig 9C; Oelschlaegel *et al*, 2005; Penkner *et al*, 2005). The Cdc5‐Spo13 kinase therefore forms as soon as Cdc5 appears and prevents free Cdc5 from inducing Rim4 degradation and subsequent *AMA1* translation. Furthermore, by phosphorylating Clb1, Cdc5‐Spo13 generates an inhibitor of Ama1. As Cdc5‐Spo13 and Cdk1‐Clb1 accumulate, they phosphorylate and activate each other. Phosphorylation of Clb1 enables Cdk1‐Clb1 to inhibit Ama1, which in turn stabilizes Clb1 and Spo13 (Fig 8B). Phosphorylation of Spo13 activates the Cdc5‐Spo13 kinase (Fig 6B and Appendix Fig S5D; Oz *et al*, 2022), which promotes the phosphorylation of Clb1 and the repression of *AMA1* translation. Thus, Cdc5‐Spo13 and Cdk1‐Clb1 are connected by direct and indirect positive feedback loops, which stabilize the low‐Ama1/high‐Cdk1 state of metaphase I (Fig 5C). Since Clb3 is translated together with and binds to Ama1, it seems well suited to assist Clb1 in repressing Ama1‐dependent proteolysis. However, we found no evidence for Cdk1‐Clb3 inhibiting the degradation of Rim4 or the activity of Ama1 (Appendix Figs S5C and S6D).

At anaphase I, Spo13 and Clb1 are substrates of APC/C^Cdc20^. Ama1 synthesis and activity are therefore unleashed in a coordinated manner when the SAC is silenced upon biorientation of bivalent chromosomes on the meiosis I‐spindle. APC/C^Cdc20^ employs a robust mechanism, known as a coherent feedforward loop, to inactivate Spo13 and the phosphorylated form of Clb1: APC/C^Cdc20^ not only targets Spo13 for proteolysis but also destroys the Spo13‐activating kinase Cdk1‐Clb1. Similarly, APC/C^Cdc20^ targets Clb1 for degradation and additionally inactivates Cdc5‐Spo13, the kinase that phosphorylates Clb1. While Spo13 is degraded at anaphase I, APC/C^Ama1^ must only be activated at anaphase II to allow the re‐accumulation of M phase regulators required for entry into metaphase II. Indeed, Rim4 degradation is not complete until cells reach metaphase II. Furthermore, degradation of Ama1‐specific substrates at anaphase II depends on high levels of Ama1 (Arguello‐Miranda *et al*, 2017; Fig 2B). This requires a high rate of translation since Ama1 is an unstable protein (Jonak *et al*, 2017). Meanwhile, Clb1 is not completely degraded at anaphase I (Buonomo *et al*, 2003), giving Cdk1‐Clb1 a head‐start on Ama1 as both accumulate at meiosis II.

Upon silencing of the SAC at meiosis II, APC/C^Cdc20^ induces the degradation of Clb1 and the concomitant activation of high levels of Ama1. While the activity of APC/C^Cdc20^ declines as cyclins are degraded, that of APC/C^Ama1^ increases, resulting in irreversible exit from meiosis. Ama1 accumulates at meiosis II despite terminating Ndt80‐dependent transcription through the degradation of Ndt80 (Okaz *et al*, 2012). Similarly, *cdc20 spo13Δ* and *cdc20 AMA1‐6A* cells accumulate Ama1 and sporulation factors while degrading Ndt80 (Figs 2A and 7A). These data imply that mRNAs encoding meiosis II‐specific proteins are protected from degradation until translation is unleashed. How mRNA protection is related to translational repression remains to be investigated.

### A conserved role for Spo13/MEIKIN in preventing premature exit from meiosis

While budding and fission yeast express meiosis‐specific APC/C activators dedicated to exit from meiosis, mammalian genomes encode only two APC/C activators, namely Cdh1 (also known as Fzr1) and Cdc20. Nevertheless, spermatocytes of *Meikin* knock‐out mice resemble the *spo13Δ* mutant in that they undergo a single division, which corresponds to meiosis I (Kim *et al*, 2015). Remarkably, the only other mutation known to date showing a similar phenotype is a non‐phosphorylatable version of Cdh1. Whereas *Cdh1‐9A* knock‐in mice develop normally, males are infertile because spermatocytes exit from meiosis after a single division, similar to the yeast *AMA1‐6A* mutant (Tanno *et al*, 2020). Thus, phosphorylation at CDK motifs is dispensable for the inhibition of Cdh1 in somatic cells. In spermatocytes, however, it is essential for entry into meiosis II, even though Cdh1‐9A is compatible with entry into meiosis I. The implication is that Cdh1‐9A amplifies its activity during meiosis I by, for instance, increased translation or degradation of an inhibitor. We therefore speculate that PLK1‐MEIKIN counteracts the Cdh1‐activating mechanisms that cause *Cdh1‐9A* spermatocytes to prematurely exit from meiosis I. Thus, Spo13 and MEIKIN might ensure a two‐division meiosis through mechanisms conserved from yeast to male mammals.

## Materials and Methods

### Construction of *Saccharomyces cerevisiae* strains

This study was performed with diploid SK1 strains (Kane & Roth, 1974) generated by mating of the appropriate haploids. Genotypes are listed in Appendix Table S1. We used a Gal4‐estrogen receptor fusion for estradiol‐inducible expression from the *GAL* promoter (called *P*_*EST*_ herein; Benjamin *et al*, 2003), the *CUP1* promoter (450 bp) for expression induced by CuSO_4_, and the *DMC1* promoter (340 bp) for expression at early prophase. Meiotic depletion was achieved with the mitosis‐specific promoters of *HSL1* (Okaz *et al*, 2012) or *SCC1* (Clyne *et al*, 2003). *P*_*EST*_ *‐NDT80 P*_*HSL1*_ *‐CDC20* strains were constructed to release cells from prophase into an arrest at metaphase I. Arrest/release at metaphase I was performed with *CDC20‐mAR* cells containing endogenous *CDC20* controlled by a mitosis‐specific promoter and an additional *P*_*CUP1*_ *‐CDC20* gene (Arguello‐Miranda *et al*, 2017). SK1 strains carrying *ime2‐as* (M146G; Benjamin *et al*, 2003), *cdc28/cdk1‐as1* (F88G; Oelschlaegel *et al*, 2005), *cdc28/cdk1‐as2* (F88A; Oz *et al*, 2022), *cdc5‐as* (L158G; Okaz *et al*, 2012), or *hrr25‐as* (I82G; Petronczki *et al*, 2006) have been described. Hyperactive kinases were generated by replacing the C‐terminal 241 (*ime2‐ΔC*) or 189 residues (*hrr25‐ΔC*) with an Ha3 tag. The alleles *spo13‐mD* (L26A; Sullivan & Morgan, 2007), *spo13‐m2* (S132T, S134T; Matos *et al*, 2008), and *spo13‐10A* (all S/T–P to A‐P; Oz *et al*, 2022) have been described. For auxin‐inducible degradation, Rim4‐mNG was tagged with AID* (residues 71–114 of IAA17; Morawska & Ulrich, 2013) and crossed into a strain expressing OsTir1‐F74G (Yesbolatova *et al*, 2020) from the *CUP1* promoter (a gift from Joao Matos). To construct *CLB1* mutants, Ser 15, 24, 52, 84, 88, 109, 117, 121, 137, 141, 143, and 164 were mutated to Ala (*clb1‐12A*) or Asp (*clb1‐12D*). In *clb1‐6A*, Ser 15, 84, 109, 137, 141, and 143 are mutated to Ala. *CLB1* alleles (−462 to +1811, ATG = +1) were cloned into YIplac128 (Gietz & Sugino, 1988) and integrated at the *clb1Δ* locus. To measure degradation rates, *CLB1‐ha6* and *SPO13* were cloned behind the *DMC1* promoter into YIplac vectors and integrated at *leu2* or *ura3* of *ndt80Δ cdk1‐as1* cells. In *AMA1‐6A*, Ser/Thr 3, 61, 63, 69, 182, and 199 are mutated to Ala. Wild‐type *AMA1* and *AMA1‐6A* (−497 to +2004, ATG = +1) were cloned into YIplac128 and integrated at the *ama1Δ* locus. For live‐imaging, we used Cnm67‐tdTomato, Htb1‐mCherry (Matos *et al*, 2008), Cdc5‐eGFP (Okaz *et al*, 2012), and Rim4 tagged with mNeonGreen (mNG; Shaner *et al*, 2013). Strains expressing Cdc14‐eGFP, eGFP‐tubulin, and TetR‐tdTomato labelling *CENV‐tetO* and the nucleoplasm have been described (Arguello‐Miranda *et al*, 2017). To monitor translation, coding sequences downstream of codon 30 (*AMA1*) or codon 15 (*GAT4*, *GIP1*, *SPS4*, *SSP2*) were replaced with *mNG*. The *AurCMX4* cassette conferring resistance to aureobasidin A carries *S. cerevisiae AUR1‐C* (F158Y, A240C; Hashida‐Okado *et al*, 1998) synthesized with alternative codons and flanked by *A. gossypii TEF1* promoter/terminator. To assess spore viability, 36 tetrads per strain were dissected on YPD plates.

### Meiotic cultures

Meiosis was induced at 30°C (Arguello‐Miranda *et al*, 2017). Briefly, respiration‐competent cells grown on YPD medium for 24 h were evenly spread on YPD plates and allowed to form a lawn (24 h). Cells were inoculated (OD_600_ = 0.3; 5 × 10^6^ cells/ml) into liquid YPA medium (YP plus 2% K‐acetate) and grown for 12 h until entry into a transient G1 arrest. Cells were washed with sporulation medium (SPM, 2% K‐acetate), inoculated (OD_600_ = 2.4) into 100 ml of SPM in a 2.8 l‐Fernbach flask, and rotated on an orbital shaker (200 rpm). *P*_*EST*_ *‐NDT80 P*_*HSL1*_ *‐CDC20* cells were released into metaphase I with estradiol (5 μM) at 7 h in SPM, and *CDC20‐mAR* cells were released into anaphase I with CuSO_4_ (10 μM) at 8 h in SPM. Kinases were inhibited with 1NM‐PP1 (Cayman Chemical; Cdk1‐as1 and Hrr25‐as, 5 μM), 1Na‐PP1 (Cayman Chemical; Cdk1‐as2, 10 μM; Ime2‐as, 20 μM), or CMK (MedChemExpress; Cdc5‐as, 20 μM). The auxin analogue 5‐Ph‐IAA (BioAcademia, Osaka, Japan) was used at 0.1 mM and cycloheximide at 0.5 mg/ml.

### Live‐cell imaging

Cells diluted to OD_600_ = 0.4 were applied to an 8‐well glass bottom μ‐slide (ibidi, Gräfelfing, Germany) coated with Concanavalin A (Sigma‐Aldrich; 0.5 mg/ml) in 250 μl of SPM per well to give a density of 20–30 cells per field of view. Drugs and CuSO_4_ were added in 125 μl of SPM. Cells were imaged on a DeltaVision Elite system (Cytiva) consisting of an Olympus IX71 microscope with autofocus (Ultimate Focus) and solid‐state illumination (InsightISS) attenuated by an ND filter (25% T), a PlanApo 100×/1.4 NA oil objective, DeltaVision filter sets, a CoolSnap HQ2 camera (Photometrics), and an environmental chamber set to 30°C. We acquired Z‐stacks (8 × 1 μm) from 8 to 10 fields of view per strain in the GFP and the RFP channel every 10 min for 14 h. Exposure times were 100–200 ms with the adjustable ND filter set to 10% (GFP) or 32% (RFP). Z‐stacks were deconvolved and projected to a single 2D‐image with softWoRx 6.1 (standard projection). Time‐lapse series (frame width, 5 μm) were produced in Fiji (http://fiji.sc/). For quantification, time series of individual cells (*n* = 100) were aligned to a reference event (e.g., SPB separation at metaphase I) set to *t* = 0 in each cell. Percentages of other features (e.g., presence of Rim4) were calculated at 10 min‐intervals relative to the reference event using Microsoft Excel.

### Immunofluorescence microscopy

Cells (1 ml) were fixed overnight at 4°C in formaldehyde (3.7%), converted to spheroplasts with Zymolyase 100T (amsbio; 10 μg/ml), and placed on 15‐well multi‐test slides (MP Biomedicals; Salah & Nasmyth, 2000). We used monoclonal antibodies to Myc (mouse 9E10, Invitrogen; 1:100) and α‐tubulin (rat YOL1/34, Serotec; 1:300) as primary antibodies, which were detected with Alexa Fluor‐conjugated secondary antibodies from donkey (Invitrogen). DNA was stained with DAPI. Cells were observed on a Zeiss Axio Imager M2 with Colibri 7 LED light source and a Plan‐Apochromat 100×/1.4 NA oil objective. Cellular features were scored in ≥ 100 cells per timepoint.

### Analysis of mRNA

RNA was extracted with the RNeasy Mini Kit (Qiagen) from cells (2 ml) converted to spheroplasts with Zymolyase 100T (amsbio; 1.4 mg/ml). Residual DNA was removed by on‐column digestion with RNase‐free DNase I. We used a Qubit 4 fluorometer (Invitrogen) to measure RNA concentration and an Agilent 2100 Bioanalyzer for quality control. cDNA was synthesized from 1 μg of total RNA using SuperScript III First‐Strand Synthesis SuperMix (Invitrogen) and random hexamers. qPCR was performed in triplicate on a StepOnePlus real‐time PCR system (Applied Biosystems) using Takyon Rox SYBR MasterMix blue dTTP (Eurogentec) and primers at 100 nM. mRNA levels were normalized to the *ACT1* transcript by calculating the 2^−ΔCt^ value for each time point (Livak & Schmittgen, 2001). We used the following primers:

*ACT1*‐fw, 5′‐gaaatgcaaaccgctctca‐3′; *ACT1*‐rv, 5′‐taccggcagattccaaaccc‐3′;

*mNG*‐fw, 5′‐ctacgagggaagccacatca‐3′; *mNG*‐rv, 5′‐agtcttcttcgacctgcacc‐3′;

*CDC5*‐fw, 5′‐acaccatatgcggaacacct‐3′; *CDC5*‐rv, 5′‐tctcttgcttggaagggtgg‐3′.

### Analysis of proteins

For immunoprecipitations, cells (40 ml) were treated with 2 mM PMSF, washed with 2 mM PMSF in cold water, and frozen in liquid nitrogen. Cells were broken with 0.5 mm‐glass beads, and lysates were cleared by centrifugation and incubation with protein A‐agarose beads (Roche; 10% v/v). Proteins were captured from extracts (0.5 ml, 12 mg/ml) with antibodies to Apc2 (Camasses *et al*, 2003; 1:200) or Myc (mouse 9E11, Invitrogen; 1:200) and protein A‐agarose beads. For immunodepletions, cleared extracts (0.5 ml; 7 mg/ml) were passed (3×) through 0.1 ml‐protein A‐agarose columns loaded with antibodies. To analyze protein levels, cells (9 ml) were collected by centrifugation, frozen in liquid nitrogen, and broken with glass beads in 10% trichloroacetic acid (Okaz *et al*, 2012). Proteins (60 μg) were separated in SDS‐8% PAA gels and transferred to Immobilon P membranes (Millipore) by semi‐dry blotting. Membranes were horizontally cut into 2–3 slices and incubated with primary antibodies for 2 h. Antibodies had been raised in rabbits to Apc10/Doc1 (1:2,000), Cdc23 (1:2,000), Cdh1 (1:5,000), and Clb3 (1:3,000) (Schwickart *et al*, 2004), Ama1 (1:2,000) and Mnd2 (1:2,000) (Oelschlaegel *et al*, 2005), Cdc5 (1:5,000) and Spo13 (1:5,000) (Matos *et al*, 2008), Apc2 (1:2,000) and Cdc20 (1:2,000) (Camasses *et al*, 2003), Ase1 (a gift from David Pellman; Juang *et al*, 1997; 1:1,000), Cdk1/Cdc28 (Petronczki *et al*, 2006); 1:5,000), Ndt80 (a gift from Kirsten Benjamin; Benjamin *et al*, 2003; 1:5,000), and Tub2/β‐tubulin (a gift from Wolfgang Seufert; Schwab *et al*, 2001; 1:20,000). We purchased rabbit antibodies to OsTir1 (Medical & Biological Laboratories PD048; 1:1,000), Myc (Sigma c3956; 1:1,000), mNG (Cell Signaling Technology 53061; 1:1,000), and Zip1 (Santa Cruz sc‐33733; 1:3,000) and goat antibodies (Santa Cruz) to Clb1 (sc‐7647; 1:300), Clb4 (sc‐6702; 1:400), Clb5 (sc‐6704; 1:200), and Ime2 (sc‐26444; 1:300). We used monoclonal antibodies to Ha (rat 3F10, Roche; 1:2,000), Act1/actin (mouse AC‐40, Sigma‐Aldrich; 1:500), GFP (mouse 7.1 and 13.1, Roche; 1:500), Myc (mouse 9E10, Invitrogen; 1:1,000), and Pgk1 (mouse 22C5D8, Invitrogen; 1:20,000). HRP‐conjugated secondary antibodies were detected on X‐ray films using ECL start reagents (Cytiva). Signals were digitized on an Epson Perfection V750 Pro scanner and quantified in Fiji (http://fiji.sc/).

### Affinity‐enrichment of GFP‐tagged proteins

Strains were cultured in 15 l‐glass beakers placed in a 30°C‐water bath and aerated through 120 mm‐air stone discs with pressurized air, which was prewarmed and humidified with a gas‐washing bottle. Cells from 12 YPD plates were inoculated (OD_600_ = 0.3) into 8 l of YPA plus antifoam SE‐15 (Sigma‐Aldrich; 1:1,000) and grown for 12 h. Cells were washed with SPM and inoculated (OD_600_ = 3) into 4 l of SPM. *P*_*HSL1*_ *‐CDC20* and *P*_*SCC1*_ *‐CDC20* strains arresting at metaphase I were cultured for 8 h. *P*_*EST*_ *‐NDT80* strains were released from prophase at 7 h in SPM and harvested 80 min (metaphase I) or 120 min (metaphase II) thereafter. Cultures were poured through crushed ice, washed with 2 mM PMSF in cold water, and resuspended in 15 ml of buffer B70 containing protease and phosphatase inhibitors (2 mM PMSF, EDTA‐free Complete, PhosSTOP; Roche). B70 is buffer B (50 mM Hepes/KOH pH 7.4, 40 mM β‐glycerophosphate, 2.5 mM Mg‐acetate, 1 mM DTT, 0.1% Triton X100, 10% glycerol) plus 70 mM K‐acetate. Subsequent steps were performed at 4°C. Cells were broken with 0.5 mm‐glass beads in a mixer mill (Retsch MM 400, 4 × 4 min, 30 Hz), and lysates were cleared by centrifugation (27,000 *g*; 45 min). Extracts (23 ml, 23 mg/ml) were incubated with Sepharose 4B (Cytiva, 13 ml of 75% slurry) for 30 min and then with agarose beads carrying anti‐GFP nanobodies (GFP‐Trap, Chromotek; 0.5 ml of 50% slurry) for 60 min. Beads were washed with 45 ml‐aliquots of buffer B70 (2 washes), B150, B200, and W (50 mM Tris/HCl pH 7.5, 70 mM NaCl, 5% glycerol; 2 washes). Beads were resuspended in 0.5 ml of buffer E (50 mM Tris/HCl pH 7.5, 2 M urea, 1 mM DTT), divided into two samples, and subjected to on‐bead digestion with modified trypsin (Promega, sequencing grade; 5 ng/μl) or Lys‐C (Promega, mass spec grade; 5 ng/μl) for 40 min at 22°C. Digests were collected by centrifugation and the beads washed with 2 × 125 μl of buffer E plus iodoacetamide (5.5 mM). Trypsin and Lys‐C digests were combined with the corresponding washes, incubated overnight at 22°C, and quenched with trifluoroacetic acid (1% final). Peptide mixtures were desalted on C_18_‐StageTips (Rappsilber *et al*, 2003), vacuum‐dried, and stored at −20°C.

### LC–MS/MS data acquisition

Trypsin and Lys‐C digests were analyzed in separate LC–MS/MS runs. A 30 cm × 75 μm‐column packed in‐house with 1.9 μm‐silica beads (ReproSil‐Pur C_18_‐AQ; Dr Maisch GmbH) was operated at 60°C by an Easy‐nLC 1200 system (Thermo Fisher Scientific). Peptides were loaded at 250 nl/min in buffer A (0.1% formic acid) plus 2% buffer B (80% acetonitrile, 0.1% formic acid) and eluted by a gradient to 30% B (120 min), 60% B (10 min), 95% B (5 min), and 95% B (5 min). Eluting peptides were transferred via the nano‐electrospray interface into a Q Exactive HF Orbitrap instrument (Thermo Fisher Scientific). Precursor ions were selected by a data‐dependent top‐12 method for high‐energy collisional dissociation with a normalized collision energy of 28 (Scheltema *et al*, 2014). MS/MS parameters and instrument settings are given in the source data.

### MS/MS data analysis

Raw MS files of corresponding trypsin and Lys‐C digests were merged and searched in MaxQuant (version 2.0.1.0) against the *S. cerevisiae* UniProt database (UP000002311) combined with the relevant GFP‐tagged bait protein and 247 common contaminants (Tyanova *et al*, 2016a). Minimum peptide length was set to seven amino acids and maximum mass to 4,600 Da. Strict trypsin or Lys‐C specificity was required, allowing up to two missed cleavages. Carbamidomethylation of Cys was set as fixed modification and N‐terminal protein acetylation, oxidation of Met, deamination of Asn or Gln, and phosphorylation of Ser, Thr, or Tyr as variable modifications. The maximum number of modifications per peptide was set to five. Relative, label‐free quantifications (LFQ) were obtained from the MaxLFQ algorithm using default parameters and further analyzed in Perseus (version 1.6.15.0; Tyanova *et al*, 2016b). Contaminants and matches to the reversed‐sequence decoy database were removed, and LFQ intensities were log_2_‐transformed. For interaction proteomics, proteins showing at least two valid values in at least one group were retained. Missing values were imputed with values from a normal distribution at the detection limit of the mass spectrometer (width, 0.3 SD; down‐shift, 1.8 SD). For Clb1 phosphorylation analysis, phospho‐sites with a localization probability of less than 0.75 were removed. Phospho‐sites with at least two valid values in the control were retained, and phosphorylation site intensities were log_2_‐transformed. Missing values were imputed as above.

### Statistical analysis

Mean values are given ± 1 SD. Distributions of the durations of metaphase I and the times from SPB separation to Rim4 degradation tend to be symmetrical or slightly skewed. Thus, we used Welch's *t*‐test (unpaired, two‐tailed) to compare means and provide 95% confidence intervals for the difference between means. With a balanced design and a sample size of *n* = 100, Welch's *t*‐test is robust against deviations from normality. Volcano plots were generated in GraphPad Prism 9 using log_2_‐transformed LFQs and Benjamini‐Hochberg‐corrected *P*‐values calculated by Perseus (Tyanova *et al*, 2016b). We used Perseus to generate heatmaps and to perform hierarchical clustering (distance metric, Euclidian; linkage criterion, Average). To compare degradation rates, linear regression was applied to log_2_‐transformed signal intensities over time, and slopes were compared with ANCOVA in GraphPad Prism 9.

## Author contributions

**Wolfgang Zachariae:** Conceptualization; formal analysis; supervision; funding acquisition; validation; investigation; visualization; writing – original draft; project administration; writing – review and editing. **Julie Rojas:** Data curation; formal analysis; validation; investigation; visualization; methodology; writing – review and editing. **Tugce Oz:** Data curation; formal analysis; validation; investigation; visualization; methodology; writing – review and editing. **Katarzyna Jonak:** Data curation; formal analysis; validation; investigation; visualization; methodology; writing – review and editing. **Oleksii Lyzak:** Data curation; formal analysis; validation; investigation; visualization; methodology; writing – review and editing. **Vinal Massaad:** Data curation; formal analysis; investigation; visualization; writing – review and editing. **Olha Biriuk:** Data curation; formal analysis; investigation; visualization; writing – review and editing.

## Disclosure and competing interests statement

The authors declare that they have no conflict of interest.

## Supporting information



Appendix

Expanded View Figures PDF

Source Data for Expanded View and Appendix

PDF+

Source Data for Figure 1

Source Data for Figure 2

Source Data for Figure 3

Source Data for Figure 4

Source Data for Figure 5

Source Data for Figure 6

Source Data for Figure 7

Source Data for Figure 8

Source Data for Figure 9

## Data Availability

The mass spectrometry proteomics data have been deposited to the ProteomeXchange Consortium via the PRIDE partner repository with the dataset identifier PXD044597 (http://www.ebi.ac.uk/pride/archive/projects/PXD044597).
