# Supplementary material for: Spo13/MEIKIN ensures a Two‐Division meiosis by preventing the activation of APC/CAma1 at meiosis I
Source: EMBO J. 2023 Sep 20;42(20):e114288. doi: 10.15252/embj.2023114288 (PMC10577557; doi:10.15252/embj.2023114288)
Supplement: Supplementary file 1 — Appendix [file EMBJ-42-e114288-s011.pdf]

APPENDIX

Spo13/MEIKIN Ensures a Two-Division Meiosis by Preventing the Activation of APC/C^{Ama1} at Meiosis I

Julie Rojas, Tugce Oz, Katarzyna Jonak, Oleksii Lyzak, Vinal Massaad, Olha Biriuk, and Wolfgang Zachariae

TABLE OF CONTENTS

Appendix Figure S1. Analysis of nuclear division in *spo13Δ* mutants

Appendix Figure S2. Ama1 but not Cdh1 activates the APC/C at metaphase I in *spo13Δ* cells

Appendix Figure S3. Analysis of hyperactive Ime2 and Hrr25

Appendix Figure S4. Inhibition of Cdc5, Ime2, and Hrr25 activity at meiosis II

Appendix Figure S5. Analysis of cells lacking Clb1 or Cdk1 activity

Appendix Figure S6. Analysis of proteins interacting with Ama1

Appendix Figure S7. Identification of Clb1 phosphorylation sites in metaphase I-arrested cells

Appendix Figure S8. Clb1 phosphorylation sites in cells progressing through meiosis

Appendix Figure S9. Restoring a two-division meiosis in *spo13Δ* mutants

Appendix Figure S10. Mnd2 is required for normal levels of Spo13 at prophase

Appendix Table S1. *Saccharomyces cerevisiae* SK1 strains used in this study

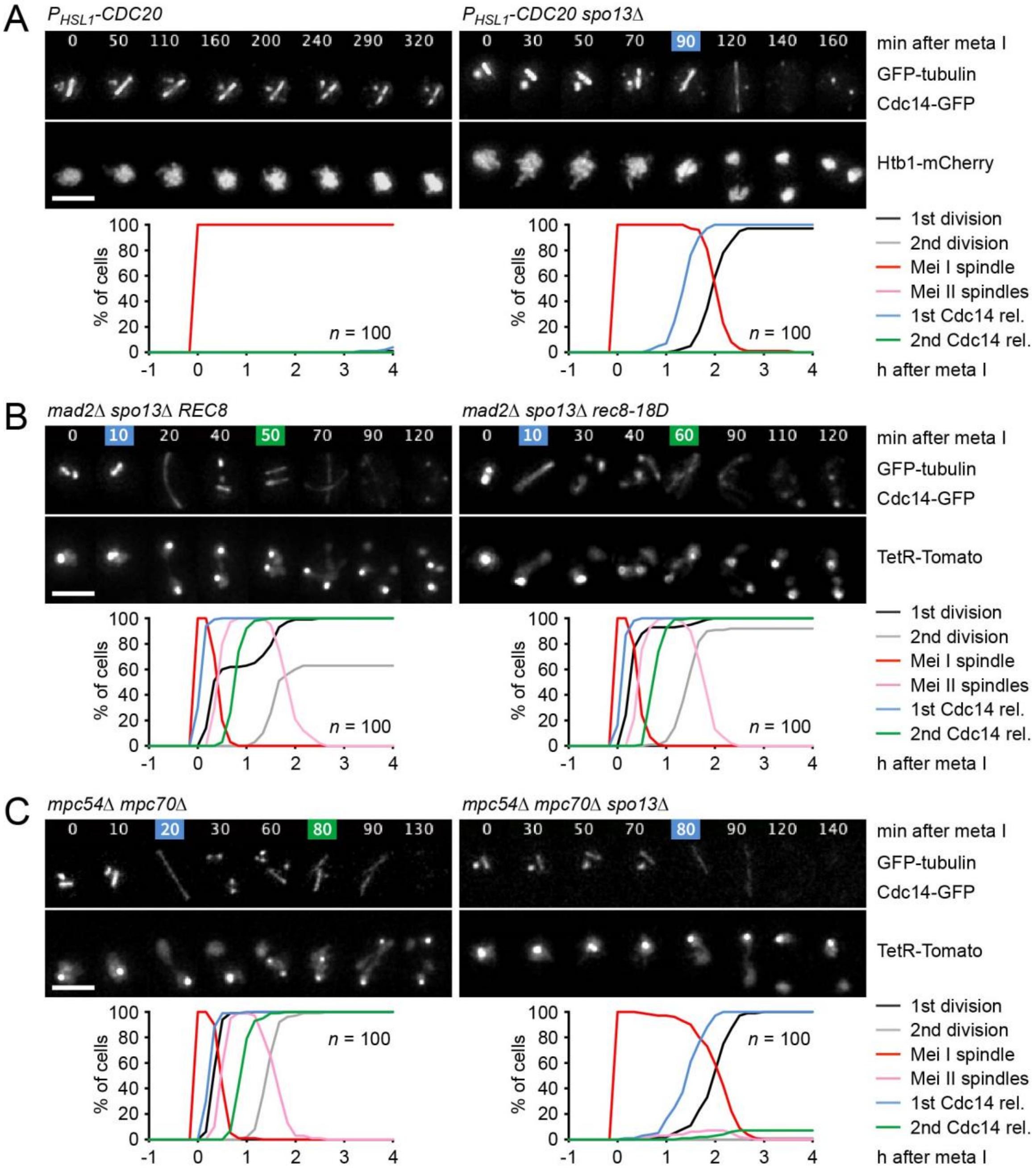

Appendix Figure S1. Analysis of nuclear division in *spo13Δ* mutants.

A-C. Imaging of spindles (GFP-tubulin), nucleolar release of Cdc14-GFP, and nuclear division (Htb1-mCherry or TetR-Tomato). TetR-Tomato labels the nucleoplasm (diffuse signal) and the centromeres of one copy of chromosome V (dots). Top, time-lapse series. First (blue) and second (green) Cdc14 release are marked. Bottom, meiotic events quantified in cells synchronized *in silico* to spindle formation at metaphase I (t = 0). (**A**) Deletion of *SPO13* causes Cdc14 release and nuclear division in Cdc20-depleted cells (P_{HSL1}-*CDC20*). (**B**) The *rec8-18D* mutation restores nuclear division at meiosis I in *mad2Δ spo13Δ* cells. (**C**) Blocking spore formation does not restore a second division in *spo13Δ* cells. Spore formation was prevented by deleting *MPC54* and *MPC70*, which encode meiotic plaque components (Knop & Strasser, 2000).

Data information: (A) is representative of two independent experiments. Scale bar, 4 μm.

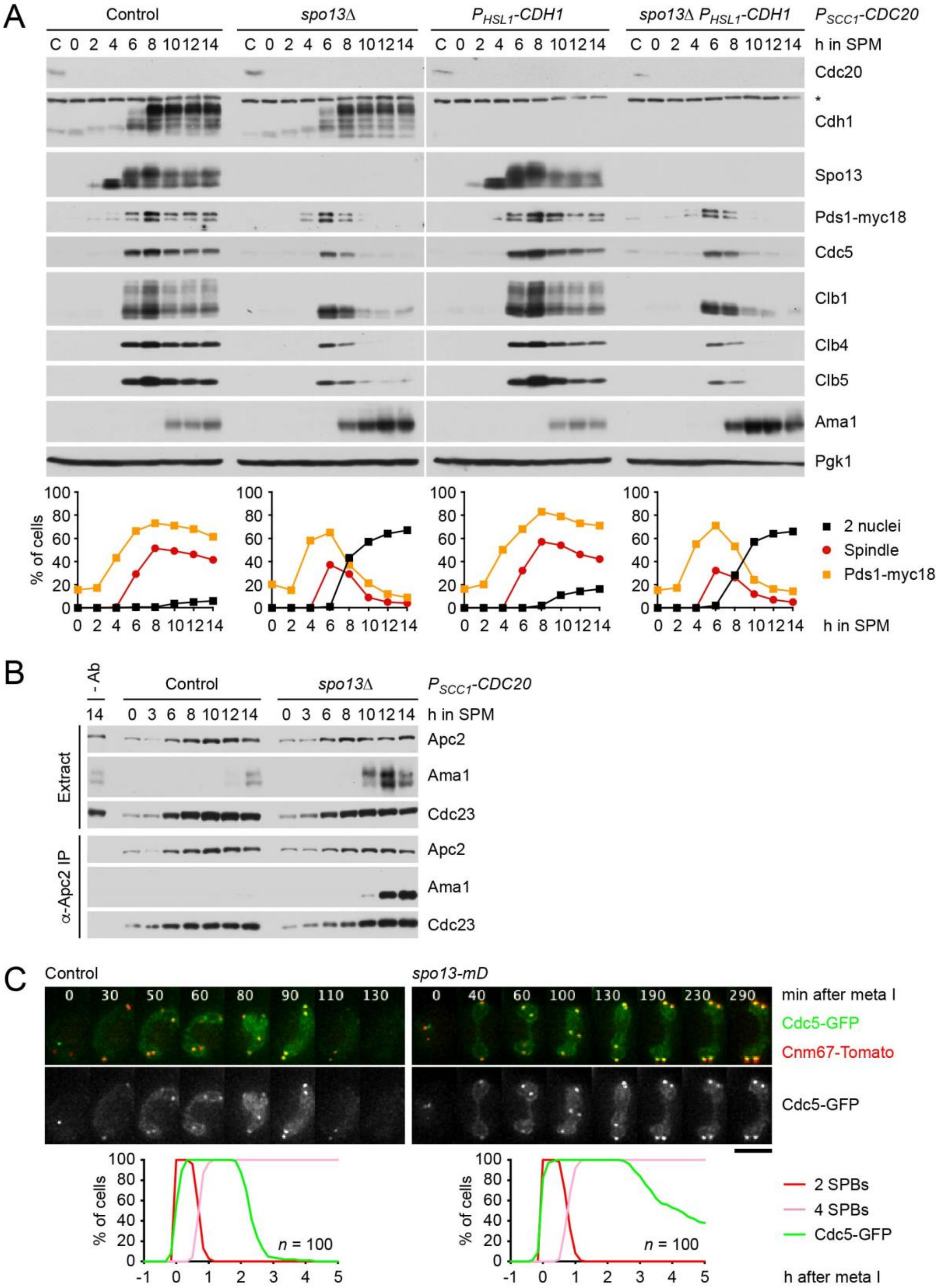

Appendix Figure S2.

◀

Appendix Figure S2. Ama1 but not Cdh1 activates the APC/C at metaphase I in *spo13Δ* cells.

A. Cdh1 is dispensable for the degradation of APC/C substrates and nuclear division in P_{SCC1}-*CDC20 spo13Δ* cells. Samples were collected from P_{SCC1}-*CDC20* cells carrying the mutations *spo13Δ* and/or P_{HSL1}-*CDH1*. Top, immunoblot detection of proteins. C, sample from proliferating cells. Asterisk, nonspecific band. Bottom, progression of meiosis quantified in fixed cells.

B. Analysis of protein extracts from P_{SCC1}-*CDC20* cells showing that deletion of *SPO13* causes accumulation and binding to the APC/C of Ama1. APC/C was immunoprecipitated with α-Apc2 antibodies. -Ab, a sample from control cells processed without α-Apc2 antibodies.

C. Imaging of Cdc5-GFP and SPBs (Cnm67-Tomato) in control and *spo13-mD* cells. Top, time-lapse series. Bottom, the presence of Cdc5 and SPB reduplication quantified in cells synchronized *in silico* to SPB separation at metaphase I (t = 0). Spo13-mD delays Cdc5 degradation by 200 min (95% CI, 163-236 min; P < 0.0001, Welch's t-test).

Data information: (C) is representative of three independent experiments. Scale bar, 4 μm.

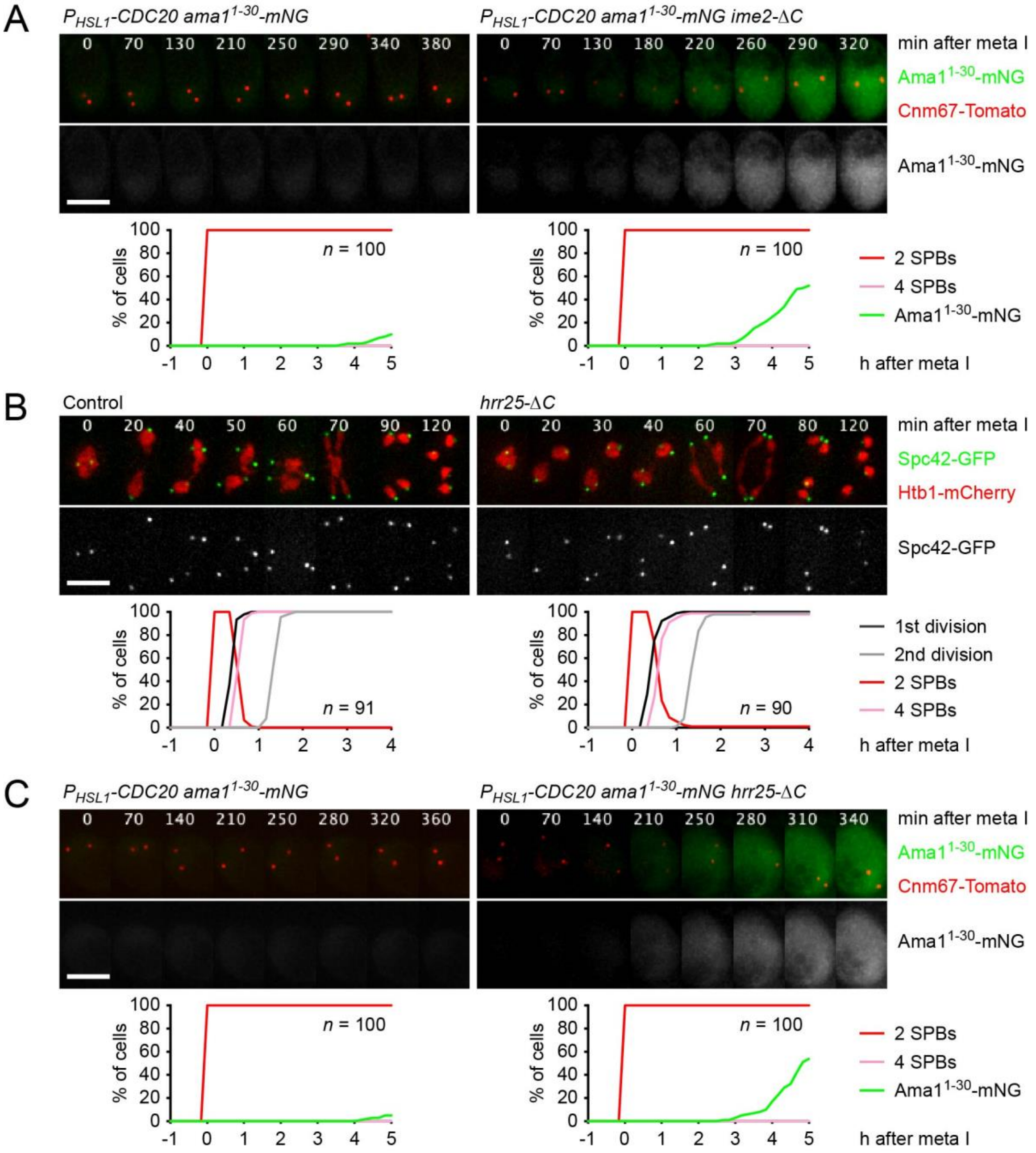

Appendix Figure S3. Analysis of hyperactive Ime2 and Hrr25.

A. Ime2-ΔC induces accumulation of mNG in P_{HSL1}-*CDC20* cells carrying *ama1*$^{1\text{-}30}$*-mNG* in place of *AMA1*. Top, time-lapse series from the imaging of mNG and SPBs (Cnm67-Tomato). Bottom, the presence of mNG quantified in cells synchronized *in silico* to SPB separation at metaphase I ($t = 0$).

B. Imaging of SPBs (Spc42-GFP) and nuclei (Htb1-mCherry) in control and *hrr25-ΔC* cells. Top, time-lapse series. Bottom, quantification of SPB reduplication and nuclear division in cells synchronized *in silico* to SPB separation at metaphase I ($t = 0$).

C. Hrr25-ΔC induces accumulation of mNG in P_{HSL1}-*CDC20* cells carrying *ama1*$^{1\text{-}30}$*-mNG* in place of *AMA1*. Top, time-lapse series from the imaging of mNG and SPBs (Cnm67-Tomato). Bottom, the presence of mNG quantified in cells synchronized *in silico* to SPB separation at metaphase I ($t = 0$).

Data information: Data are representative of two (A and B) or three (C) independent experiments. Scale bar, 4 μm.

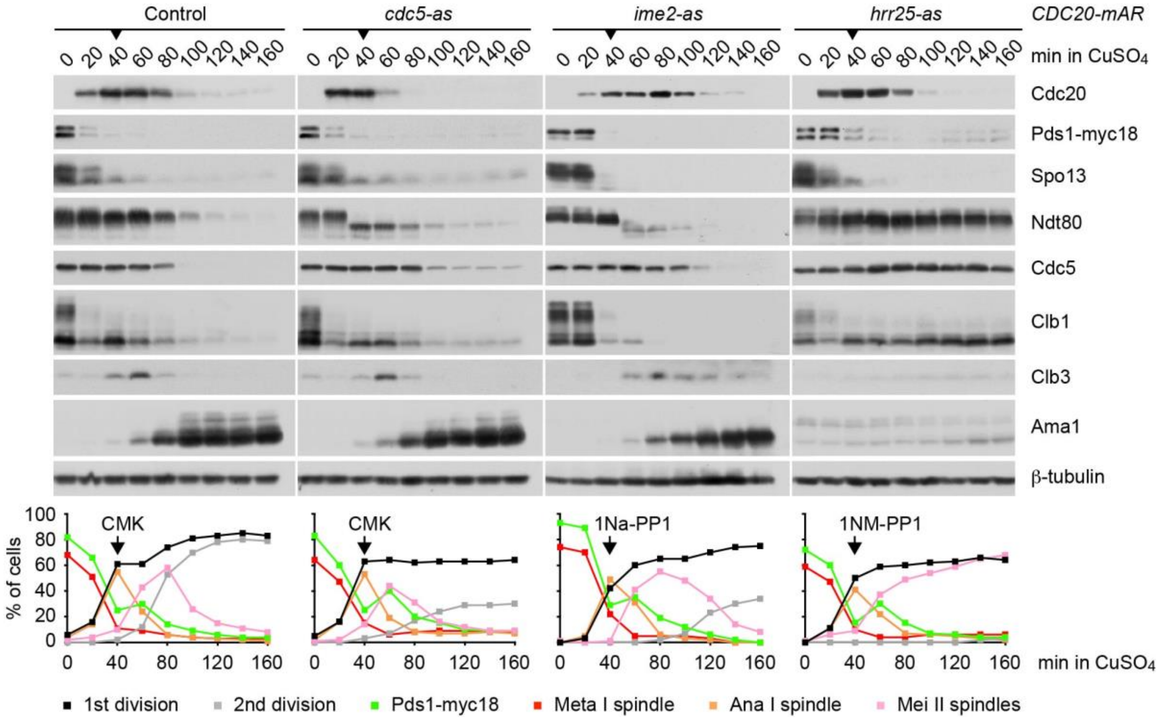

Appendix Figure S4. Inhibition of Cdc5, Ime2, and Hrr25 activity at meiosis II.
CDC20-mAR strains were released from the metaphase I-arrest with $CuSO_4$ at 8 h in SPM (t = 0). Cdc5-as, Ime2-as, or Hrr25-as was inhibited with the indicated ATP-analogue at anaphase I (t = 40 min, arrows). Top, immunoblot detection of proteins. Bottom, meiotic events quantified in fixed cells. While inhibition of Hrr25 at anaphase I blocks accumulation of Ama1 and Clb3 at meiosis II, inhibition of Cdc5 or Ime2 has no such effect.

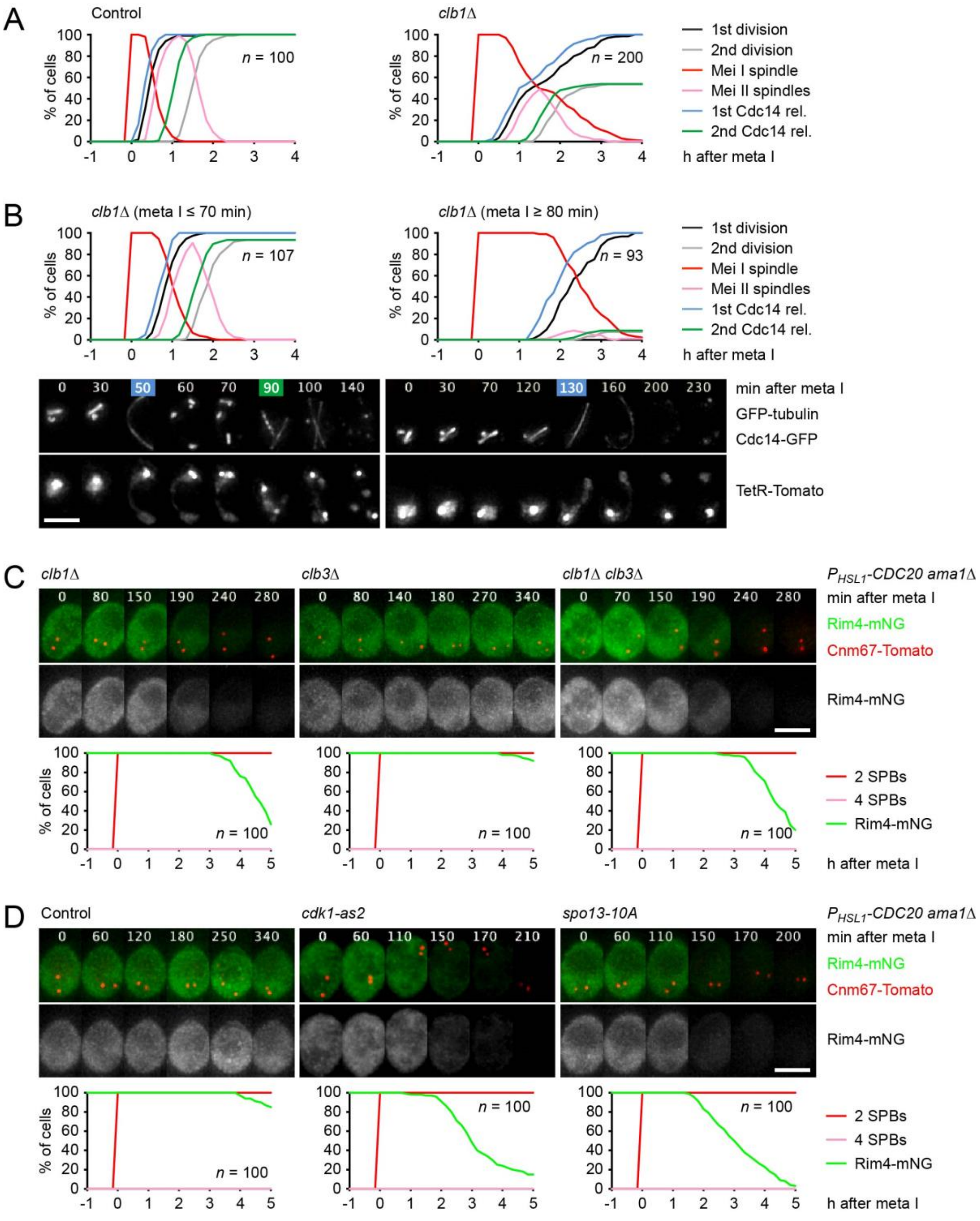

Appendix Figure S5.

◀

Appendix Figure S5. Analysis of cells lacking Clb1 or Cdk1 activity.

A, B. Analysis of meiosis in control and *clb1Δ* cells by imaging of spindles (GFP-tubulin), nucleolar release of Cdc14-GFP, and TetR-Tomato, which labels the nucleoplasm (diffuse signal) and the centromeres of one copy of chromosome V (dots). (**A**) Meiotic events quantified in control and *clb1Δ* cells synchronized *in silico* to spindle formation at metaphase I ($t = 0$). Metaphase I lasts 25 ± 10 min in control and 81 ± 44 min in *clb1Δ* cells ($P < 0.0001$, Welch's *t*-test). (**B**) Top, *clb1Δ* cells from (A) were separated into cells with a short (≤ 70 min, left) or a long metaphase I (≥ 80 min, right). Bottom, time-lapse series of *clb1Δ* cells with a short (50 min) and a long metaphase I (130 min). First (blue) and second (green) Cdc14 release are marked.

C, D. Imaging of Rim4-mNG and SPBs (Cnm67-Tomato) in metaphase I-arrested P_{HSL1}-*CDC20 ama1Δ* cells. Top, time-lapse series. Bottom, the presence of Rim4-mNG quantified in cells synchronized *in silico* to SPB separation at metaphase I ($t = 0$). (**C**) Deletion of *CLB1* causes Rim4 degradation, while deletion of *CLB3* has no effect, even in *clb1Δ* cells ($P = 0.73$, Welch's *t*-test). (**D**) Inhibition of Cdk1 and the *spo13-10A* mutation cause Rim4 degradation. Cdk1-as2 was inhibited with 1Na-PP1 at 7 h in SPM.

Data information: Data are representative of two (A-C) or three (D) independent experiments. Scale bar, 4 μm.

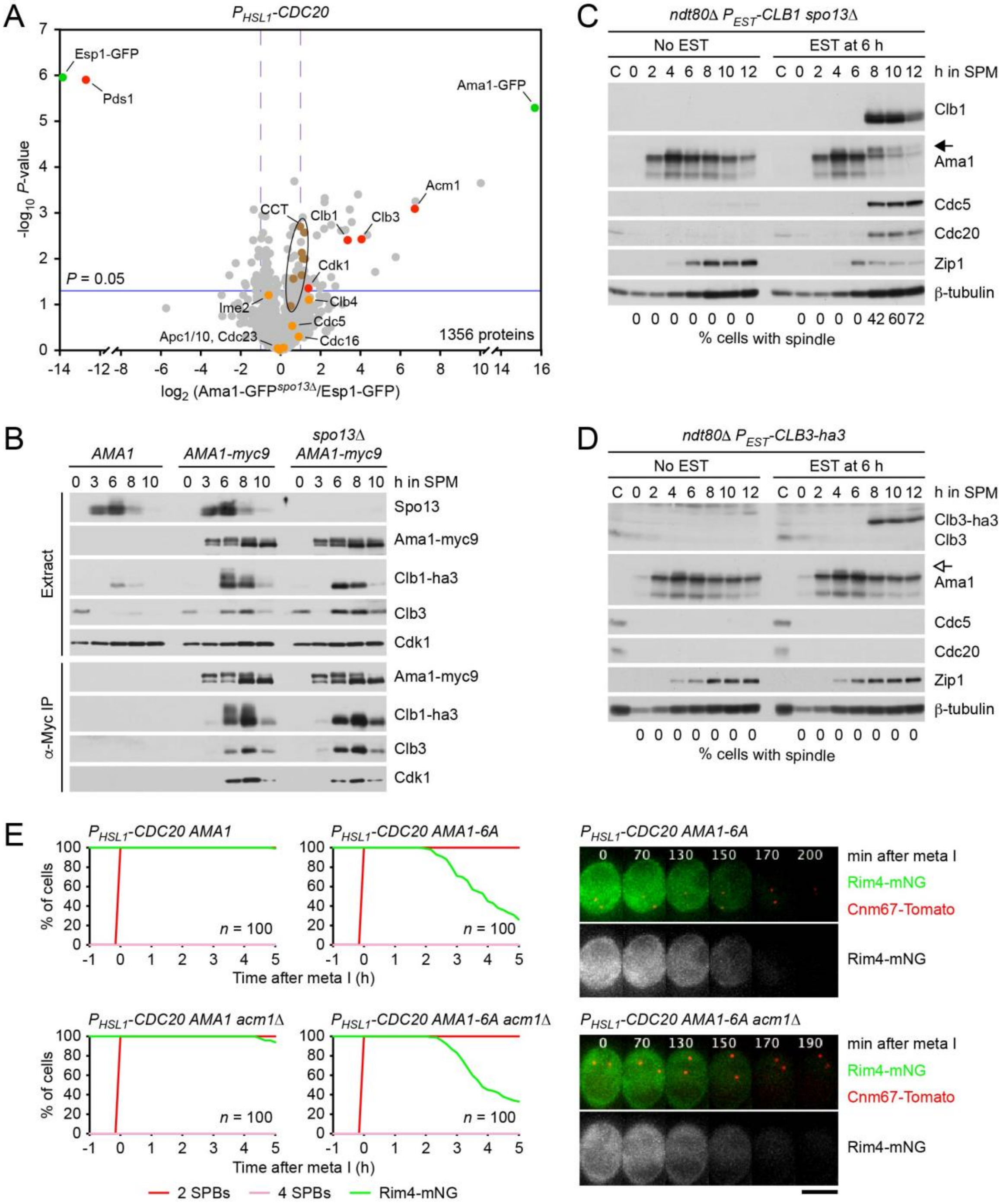

Appendix Figure S6.

◀

Appendix Figure S6. Analysis of proteins interacting with Ama1.

A. Identification of proteins copurifying with Ama1-GFP from metaphase I-arrested P_{HSL1}-*CDC20 spo13Δ* cells (8 h in SPM). Esp1-GFP from P_{HSL1}-*CDC20* cells served as negative control. Proteins digested with trypsin and Lys-C were analysed by LC-MS/MS. -$\log_{10}$-transformed *P*-values and mean $\log_2$-transformed label-free quantifications of co-purifying proteins were obtained from MaxQuant and displayed as a volcano plot. Baits (green), selected proteins with $P < 0.05$ (red) or $P > 0.05$ (orange), and the eight subunits of the CCT chaperonin (brown) are labelled. CCT is known to encapsulate and fold APC/C activators (Camasses et al., 2003). Only a subset of APC/C subunits is marked. Note that Ama1-GFP cannot bind the APC/C.

B. Clb1, Clb3, and Cdk1 bind to Ama1-myc9 immunoprecipitated with α-Myc antibodies from protein extracts of control and *spo13Δ* cells.

C, D. Immunoblot detection of proteins in *ndt80Δ* cells expressing Clb1 or Clb3-ha3 from an estradiol-inducible promoter at 6 h in SPM. The presence of a spindle was scored in fixed cells. (**C**) Clb1 inhibits Ama1-dependent proteolysis not only in *ndt80Δ* cells (Okaz et al., 2012) but also in *ndt80Δ spo13Δ* cells, resulting in the accumulation of Cdc5 and Cdc20 due to stabilization of the mitotic transcription factor Ndd1. Cdc5 activity induces degradation of Zip1. The arrow marks a Clb1- and Cdk1-dependent modification of Ama1. (**D**) Clb3 expression in *ndt80Δ* cells does not affect Ama1 modification (open arrow) or Ama1-dependent proteolysis.

E. Imaging of Rim4-mNG and SPBs (Cnm67-Tomato) in P_{HSL1}-*CDC20* cells carrying *AMA1-6A* and/or *acm1Δ*. Left, the presence of Rim4-mNG quantified in cells synchronized *in silico* to SPB separation at metaphase I ($t = 0$). P_{HSL1}-*CDC20 AMA1-6A* cells carrying *ACM1* or *acm1Δ* degrade Rim4 with similar timing ($P = 0.31$, Welch's *t*-test). Right, time-lapse series.

Data information: (A) Ama1-GFP purified from P_{HSL1}-*CDC20 spo13Δ* cells was analysed together with Ama1-GFP and Esp1-GFP from P_{HSL1}-*CDC20* cells (Fig EV4D). Data are based on α-GFP purifications from three independent cultures per strain. (E) is representative of two independent experiments. Scale bar, 4 μm.

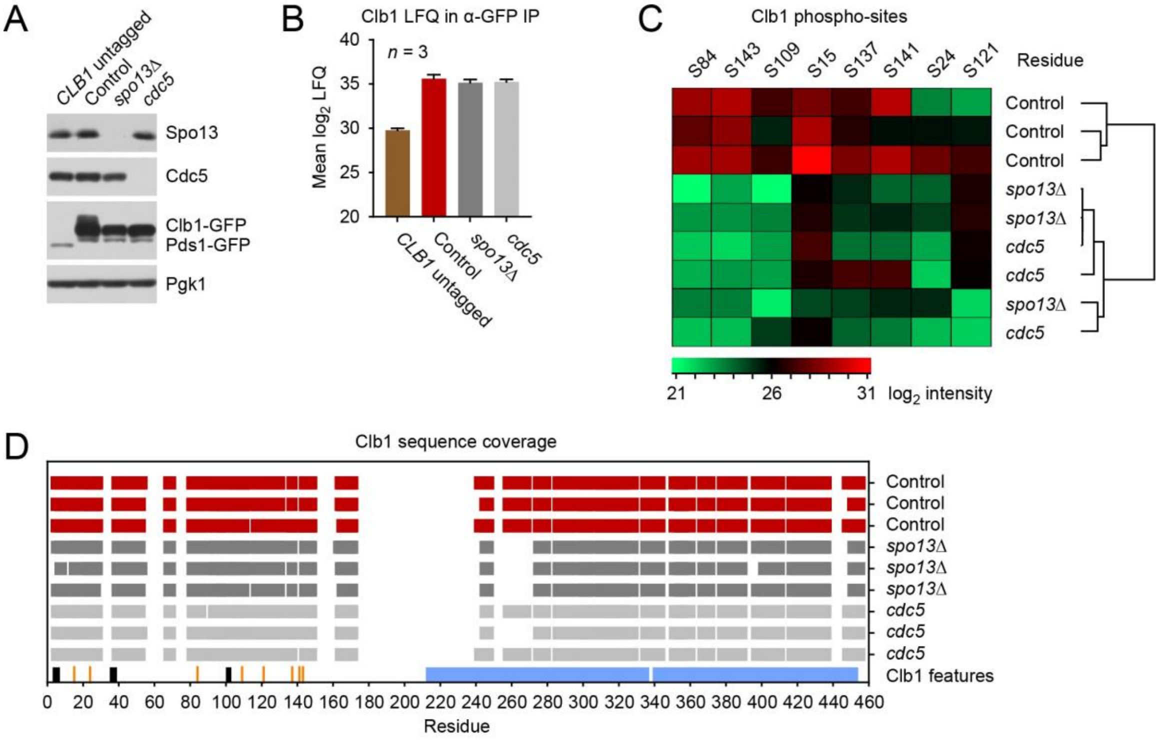

Appendix Figure S7. Identification of Clb1 phosphorylation sites in metaphase I-arrested cells.

A-D. Extracts for α-GFP immuno-purification were prepared from metaphase I-arrested *P_{SCC1}-CDC20 ama1Δ CLB1-GFP* control cells and cells carrying *spo13Δ* or *P_{SCC1}-CDC5* (*cdc5*). *P_{SCC1}-CDC20 ama1Δ PDS1-GFP* cells provided extracts containing untagged Clb1. Eluted proteins were digested with trypsin and Lys-C and subjected to LC-MS/MS. Data were analysed in MaxQuant. (**A**) Immunoblot detection of proteins in extracts from metaphase I-arrested cells (8 h in SPM). (**B**) Mean log_2-transformed label-free quantification (LFQ) of Clb1 from three independent purifications per strain. Error bars represent S.D. (**C**) Heatmap and hierarchical clustering of mean log_2-transformed intensities of Clb1 phosphorylation sites. (**D**) Sequence coverage of Clb1 from the indicated strains. D- and KEN-boxes (black), phospho-serines (orange), and cyclin boxes (blue) are labelled.

Data information: Data are based on α-GFP purifications from three independent cultures per strain.

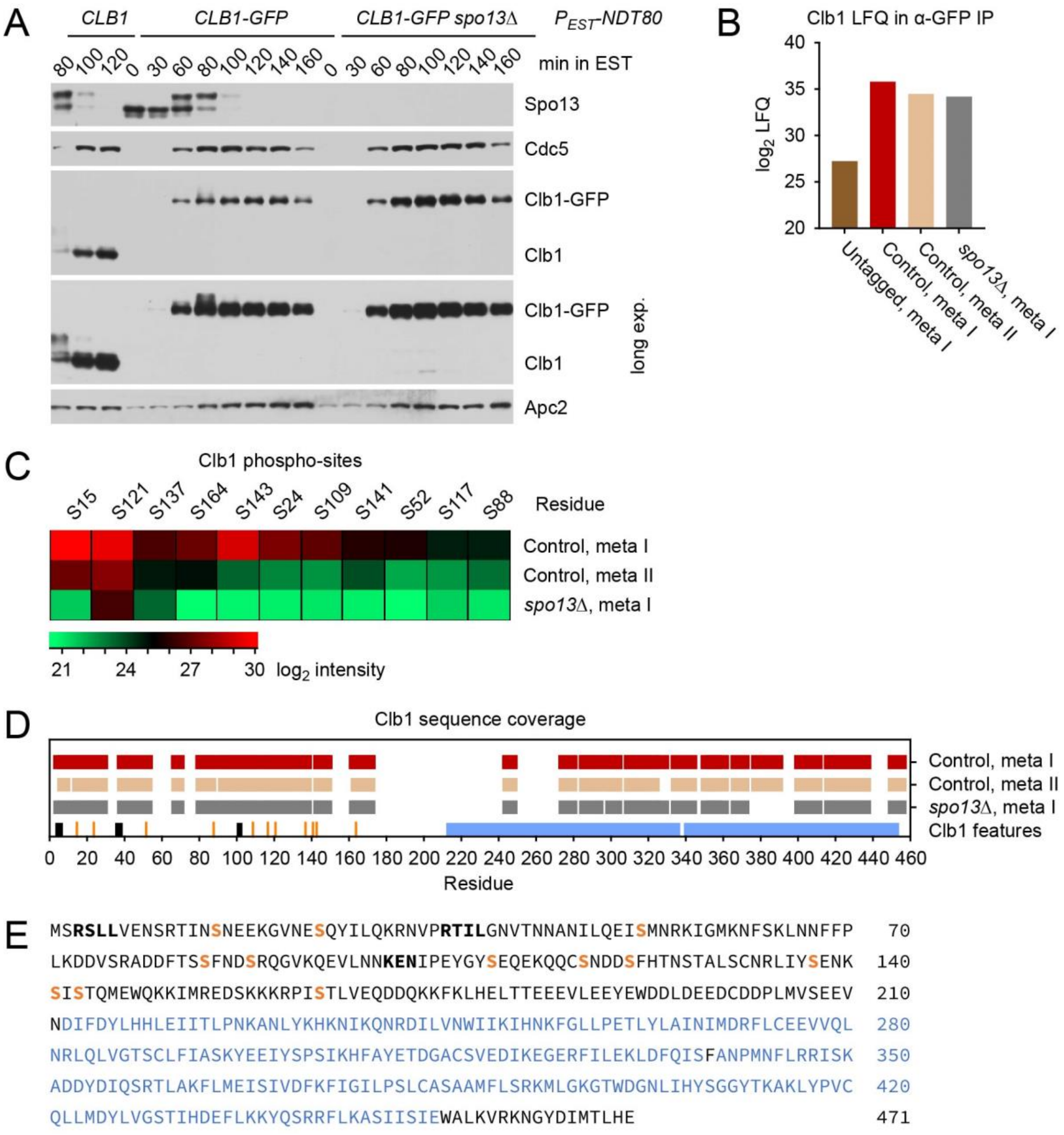

Appendix Figure S8. Clb1 phosphorylation sites in cells progression through meiosis.

A-D. Extracts for α-GFP immuno-purification were prepared from *P*$_{EST}$*-NDT80 CLB1-GFP* control cells released from the prophase arrest for 80 min (metaphase I) or 120 min (metaphase II) and from cells carrying *spo13Δ* released for 80 min (metaphase I). *P*$_{EST}$*-NDT80* cells provided extracts containing untagged Clb1. Eluted proteins were digested with trypsin and Lys-C and subjected to LC-MS/MS followed by data analysis in MaxQuant. (**A**) Immunoblot detection of proteins in cells released from the prophase arrest with estradiol (t = 0). Overexposure of Clb1 reveals a Spo13-dependent phosphorylation shift at t = 80 min. (**B**) $\log_2$-transformed LFQs of Clb1 in the indicated samples. (**C**) Heatmap of $\log_2$-transformed intensities of Clb1 phosphorylation sites. (**D**) Sequence coverage of Clb1 from the indicated samples. D- and KEN-boxes (black), phospho-serines (orange), and cyclin boxes (blue) are labelled.

E. Clb1 amino acid sequence. D- and KEN-boxes (bold), phosphorylation sites (orange), and the cyclin-box domain (blue) are labelled.

Data information: Data in (A-D) are based on α-GFP purifications from one culture per strain.

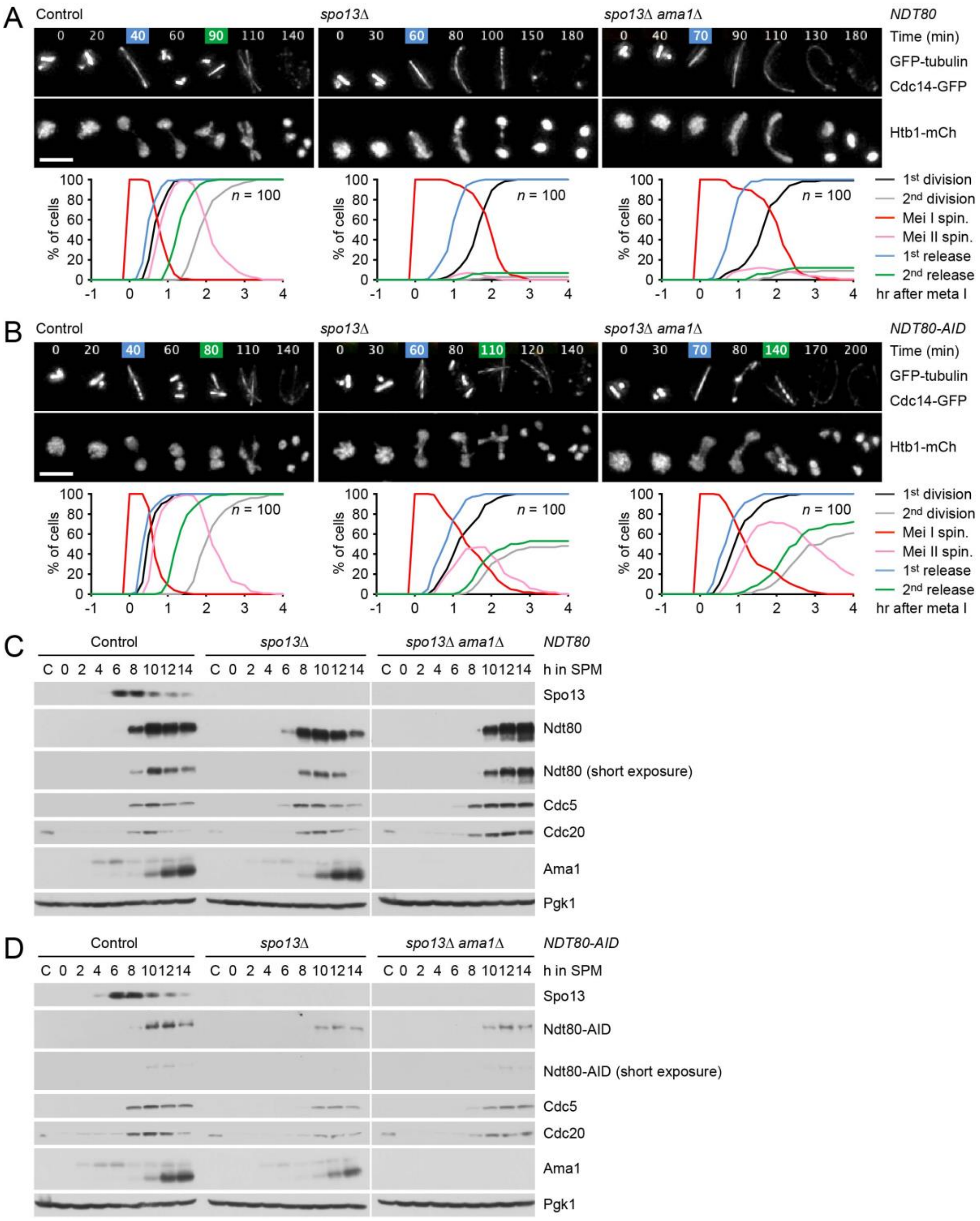

Appendix Figure S9.

◀

Appendix Figure S9. Restoring a two-division meiosis in *spo13Δ* mutants.

A, B. Meiosis in control, *spo13Δ*, and *spo13Δ ama1Δ* strains carrying wild-type *NDT80* or *NDT80-AID*. Top, time-lapse series from the imaging of spindles (GFP-tubulin), nucleolar release of Cdc14-GFP, and nuclear division (Htb1-mCherry). First (blue) and second (green) Cdc14 release are marked. Bottom, meiotic events quantified in cells synchronized *in silico* to spindle formation at metaphase I ($t = 0$). (**A**) *spo13Δ ama1Δ* cells undergo only one division, similar to *spo13Δ* single mutants ($P = 0.13$). (**B**) Ndt80-AID causes *spo13Δ* and *spo13Δ ama1Δ* cells to undergo two rounds of spindle formation, Cdc14 release, and nuclear division ($P < 0.0001$). Note that cells do not contain *OsTIR1*.

C, D. Protein extracts of *NDT80* and *NDT80-AID* strains from (A) and (B) were analysed by immunoblotting. (**C**) Deletion of *AMA1* causes accumulation of Ndt80, Cdc5, and Cdc20 in *spo13Δ* cells. (**D**) *NDT80-AID* strains produce low levels of Ndt80, Cdc5, and Cdc20.

Data information: *NDT80* and *NDT80-AID* strains were cultured and analysed together. The incidence of nuclear division was compared using Fisher's exact test. (A) is representative of two independent experiments. Scale bar, 4 µm.

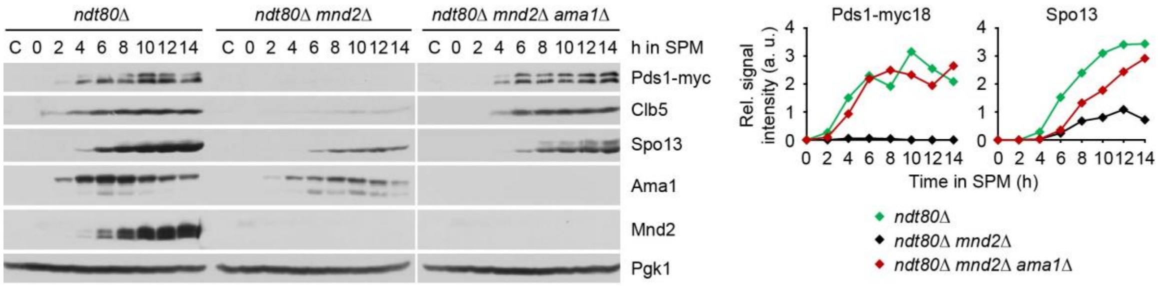

Appendix Figure S10. Mnd2 is required for normal levels of Spo13 at prophase.

Left, immunoblot analysis of protein levels at prophase in *ndt80Δ* control cells and *ndt80Δ* cells carrying *mnd2Δ* or *mnd2Δ* plus *ama1Δ*. Right, relative signal intensities of Pds1-myc18 and Spo13.

Appendix Table S1. ***Saccharomyces cerevisiae*** **SK1 Strains Used in this Study**

Figure	Strain[1]	Genotype[2]
1A	Z24864	*CDC14/CDC14-eGFP::LEU2 ura3/ura3::P$_{TUB1}$-eGFP-TUB1-URA3 CEN5/CEN5::tetO$_{224}$::HIS3 leu2/leu2::P$_{URA3}$-tetR-tdTomato::LEU2*
1A	Z24862	*CDC14/CDC14-eGFP::LEU2 ura3/ura3::P$_{TUB1}$-eGFP-TUB1-URA3 CEN5/CEN5::tetO$_{224}$::HIS3 leu2/leu2::P$_{URA3}$-tetR-tdTomato::LEU2 spo13Δ::HIS3MX6*
1B	Z24863	*CDC14/CDC14-eGFP::LEU2 ura3/ura3::P$_{TUB1}$-eGFP-TUB1-URA3 CEN5/CEN5::tetO$_{224}$::HIS3 leu2/leu2::P$_{URA3}$-tetR-tdTomato::LEU2 mad2Δ::KlURA3*
1B	Z24861	*CDC14/CDC14-eGFP::LEU2 ura3/ura3::P$_{TUB1}$-eGFP-TUB1-URA3 CEN5/CEN5::tetO$_{224}$::HIS3 leu2/leu2::P$_{URA3}$-tetR-tdTomato::LEU2 mad2Δ::KlURA3 spo13Δ::HIS3MX6*
1C	Z11674	*CDC5-eGFP::KlTRP1 CNM67-tdTomato::NatMX4*
1C	Z12066	*CDC5-eGFP::KlTRP1 CNM67-tdTomato::NatMX4 spo13Δ::HIS3MX6*
1D	Z40004	*CDC5-eGFP::KlTRP1 CNM67-tdTomato::NatMX4 mad2Δ::KlURA3*
1D	Z40005	*CDC5-eGFP::KlTRP1 CNM67-tdTomato::NatMX4 mad2Δ::KlURA3 spo13Δ::HIS3MX6*
2A	Z32563	*PDS1-myc18::KlTRP1 cdc20::P$_{SCC1}$-CDC20-KanMX6*
2A	Z32562	*PDS1-myc18::KlTRP1 cdc20::P$_{SCC1}$-CDC20-KanMX6 spo13Δ::HIS3MX6*
2A	Z32845	*PDS1-myc18::KlTRP1 cdc20::P$_{SCC1}$-CDC20-KanMX6 ama1Δ::NatMX4*
2A	Z32846	*PDS1-myc18::KlTRP1 cdc20::P$_{SCC1}$-CDC20-KanMX6 ama1Δ::NatMX4 spo13Δ::HIS3MX6*
2B	Z40205	*CDC5-eGFP::KlTRP1 CNM67-tdTomato::NatMX4*
2B	Z40206	*CDC5-eGFP::KlTRP1 CNM67-tdTomato::NatMX4 spo13Δ::HIS3MX6::spo13-mD::HphMX4*
2C	Z39219	*PDS1-myc18::KlTRP1 cdc20::P$_{HSL1}$-CDC20-HphMX4 ama1$^{1-30}$::mNG-KlTRP1*
2C	Z39220	*PDS1-myc18::KlTRP1 cdc20::P$_{HSL1}$-CDC20-HphMX4 ama1$^{1-30}$::mNG-KlTRP1 spo13Δ::HIS3MX6*
3A	Z40478	*PDS1-myc18::KlTRP1 cdc20::P$_{HSL1}$-CDC20-HphMX4 RIM4-mNG-AID*::KanMX4*
3A	Z40479	*PDS1-myc18::KlTRP1 cdc20::P$_{HSL1}$-CDC20-HphMX4 RIM4-mNG-AID*::KanMX4 his3::P$_{CUP1}$-OsTIR1-F74G::HIS3*
3A	Z40480	*PDS1-myc18::KlTRP1 cdc20::P$_{HSL1}$-CDC20-HphMX4 RIM4-mNG-AID*::KanMX4 ama1Δ::NatMX4*
3A	Z40481	*PDS1-myc18::KlTRP1 cdc20::P$_{HSL1}$-CDC20-HphMX4 RIM4-mNG-AID*::KanMX4 ama1Δ::NatMX4 his3::P$_{CUP1}$-OsTIR1-F74G::HIS3*
3B	Z35877	*RIM4-mNG::KlTRP1 CNM67-tdTomato::NatMX4*
3B	Z35876	*RIM4-mNG::KlTRP1 CNM67-tdTomato::NatMX4 spo13Δ::HIS3MX6*
3B	Z38625	*RIM4-mNG::KlTRP1 CNM67-tdTomato::NatMX4 spo13Δ::HIS3MX6::spo13-mD::HphMX4*
3C	Z39726	*cdc20::P$_{HSL1}$-CDC20-HphMX4 RIM4-mNG::KlTRP1 CNM67-tdTomato::NatMX4*
3C	Z39848	*cdc20::P$_{HSL1}$-CDC20-HphMX4 RIM4-mNG::KlTRP1 CNM67-tdTomato::NatMX4 spo13Δ::HIS3MX6*
4A	Z38375	*cdc20::P$_{HSL1}$-CDC20-HphMX4 ama1Δ::CaURA3 RIM4-mNG::KlTRP1 CNM67-tdTomato::NatMX4 ime2Δ::KanMX4::ime2-as-LEU2*
4A	Z35847	*cdc20::P$_{HSL1}$-CDC20-HphMX4 ama1Δ::CaURA3 RIM4-mNG::KlTRP1 CNM67-tdTomato::NatMX4 spo13Δ::HIS3MX6*
4A	Z38374	*cdc20::P$_{HSL1}$-CDC20-HphMX4 ama1Δ::CaURA3 RIM4-mNG::KlTRP1 CNM67-tdTomato::NatMX4 spo13Δ::HIS3MX6 ime2Δ::KanMX4::ime2-as-LEU2*

4B	Z36479	*PDS1-myc18::KlTRP1 ndt80Δ::NatMX4 leu2::*P_{GAL1}*-NDT80-LEU2 his3::*P_{GPD}*-*$GAL4^{484}$*-ER-HIS3 cdc20::*P_{HSL1}*-CDC20-HphMX4*
4B	Z36581	*PDS1-myc18::KlTRP1 ndt80Δ::NatMX4 leu2::*P_{GAL1}*-NDT80-LEU2 his3::*P_{GPD}*-*$GAL4^{484}$*-ER-HIS3 cdc20::*P_{HSL1}*-CDC20-HphMX4 ime2Δ::KanMX4::ime2-as-LEU2*
4B	Z36480	*PDS1-myc18::KlTRP1 ndt80Δ::NatMX4 leu2::*P_{GAL1}*-NDT80-LEU2 his3::*P_{GPD}*-*$GAL4^{484}$*-ER-HIS3 cdc20::*P_{HSL1}*-CDC20-HphMX4 spo13Δ::HIS3MX6*
4B	Z36582	*PDS1-myc18::KlTRP1 ndt80Δ::NatMX4 leu2::*P_{GAL1}*-NDT80-LEU2 his3::*P_{GPD}*-*$GAL4^{484}$*-ER-HIS3 cdc20::*P_{HSL1}*-CDC20-HphMX4 spo13Δ::HIS3MX6 ime2Δ::KanMX4::ime2-as-LEU2*
4C	Z38121	*cdc20::*P_{HSL1}*-CDC20-HphMX4 ama1Δ::CaURA3 RIM4-mNG::KlTRP1 CNM67-tdTomato::NatMX4 ime2-ΔC-ha3::HIS3MX6*
4C	Z38861	*cdc20::*P_{HSL1}*-CDC20-HphMX4 ama1Δ::CaURA3 RIM4-mNG::KlTRP1 CNM67-tdTomato::NatMX4 cdc5-as::HphMX4*
4C	Z38862	*cdc20::*P_{HSL1}*-CDC20-HphMX4 ama1Δ::CaURA3 RIM4-mNG::KlTRP1 CNM67-tdTomato::NatMX4 cdc5-as::HphMX4 ime2-ΔC-ha3::HIS3MX6*
5A	Z36479	*PDS1-myc18::KlTRP1 ndt80Δ::NatMX4 leu2::*P_{GAL1}*-NDT80-LEU2 his3::*P_{GPD}*-*$GAL4^{484}$*-ER-HIS3 cdc20::*P_{HSL1}*-CDC20-HphMX4*
5A	Z36595	*PDS1-myc18::KlTRP1 ndt80Δ::NatMX4 leu2::*P_{GAL1}*-NDT80-LEU2 his3::*P_{GPD}*-*$GAL4^{484}$*-ER-HIS3 cdc20::*P_{HSL1}*-CDC20-HphMX4 cdc5-as::HphMX4*
5A	Z36480	*PDS1-myc18::KlTRP1 ndt80Δ::NatMX4 leu2::*P_{GAL1}*-NDT80-LEU2 his3::*P_{GPD}*-*$GAL4^{484}$*-ER-HIS3 cdc20::*P_{HSL1}*-CDC20-HphMX4 spo13Δ::HIS3MX6*
5A	Z36596	*PDS1-myc18::KlTRP1 ndt80Δ::NatMX4 leu2::*P_{GAL1}*-NDT80-LEU2 his3::*P_{GPD}*-*$GAL4^{484}$*-ER-HIS3 cdc20::*P_{HSL1}*-CDC20-HphMX4 spo13Δ::HIS3MX6 cdc5-as::HphMX4*
5B	Z40361	*ndt80Δ::HIS3 spo13Δ::BleMX4 RIM4/RIM4-mNG::KlTRP1 CNM67-tdTomato::NatMX4 his3::*P_{GPD}*-*$GAL4^{484}$*-ER-HIS3*
5B	Z40362	*ndt80Δ::HIS3 spo13Δ::BleMX4 RIM4/RIM4-mNG::KlTRP1 CNM67-tdTomato::NatMX4 his3::*P_{GPD}*-*$GAL4^{484}$*-ER-HIS3 ura3::*P_{GAL1}*-CDC5-ha3-URA3*
5B	Z40363	*ndt80Δ::HIS3 spo13Δ::BleMX4 RIM4/RIM4-mNG::KlTRP1 CNM67-tdTomato::NatMX4 his3::*P_{GPD}*-*$GAL4^{484}$*-ER-HIS3 ura3::*P_{GAL1}*-CDC5-ha3-URA3 ime2Δ::KanMX4::ime2-as-LEU2*
6A	Z39137	*PDS1-myc18::KlTRP1 cdc20::*P_{HSL1}*-CDC20-HphMX4*
6A	Z39135	*PDS1-myc18::KlTRP1 cdc20::*P_{HSL1}*-CDC20-HphMX4 clb1Δ::NatMX4*
6A	Z39136	*PDS1-myc18::KlTRP1 cdc20::*P_{HSL1}*-CDC20-HphMX4 ama1Δ::KanMX4*
6A	Z39134	*PDS1-myc18::KlTRP1 cdc20::*P_{HSL1}*-CDC20-HphMX4 ama1Δ::KanMX4 clb1Δ::NatMX4*
6B	Z39727	*cdc20::*P_{HSL1}*-CDC20-HphMX4 RIM4/RIM4-mNG::KlTRP1 CNM67-tdTomato::NatMX4 clb1Δ::BleMX4*
6B	Z40068	*cdc20::*P_{HSL1}*-CDC20-HphMX4 RIM4/RIM4-mNG::KlTRP1 CNM67-tdTomato::NatMX4 cdc28-as2*
6B	Z39987	*cdc20::*P_{HSL1}*-CDC20-HphMX4 RIM4/RIM4-mNG::KlTRP1 CNM67-tdTomato::NatMX4 spo13Δ::HIS3MX6::spo13-10A::HphMX4*
6C	Z36479	*PDS1-myc18::KlTRP1 leu2::*P_{GAL1}*-NDT80-LEU2 his3::*P_{GPD}*-*$GAL4^{484}$*-ER-HIS3 cdc20::*P_{HSL1}*-CDC20-HphMX4*
6C	Z39319	*PDS1-myc18::KlTRP1 leu2::*P_{GAL1}*-NDT80-LEU2 his3::*P_{GPD}*-*$GAL4^{484}$*-ER-HIS3 cdc20::*P_{HSL1}*-CDC20-HphMX4 cdc28-as2*
7A	Z41535	*PDS1-myc18::KlTRP1 cdc20::*P_{HSL1}*-CDC20-HphMX4 ama1Δ::CaURA3::AMA1-LEU2*
7A	Z41536	*PDS1-myc18::KlTRP1 cdc20::*P_{HSL1}*-CDC20-HphMX4 ama1Δ::KanMX4::AMA1-6A-LEU2*

7B	Z40123	*cdc20::*P_{HSL1}*-CDC20-HphMX4 RIM4/RIM4-mNG::KlTRP1 CNM67-tdTomato::NatMX4 ama1Δ::CaURA3::AMA1-LEU2*
7B	Z40124	*cdc20::*P_{HSL1}*-CDC20-HphMX4 RIM4/RIM4-mNG::KlTRP1 CNM67-tdTomato::NatMX4 ama1Δ::KanMX4::AMA1-6A-LEU2*
7C	Z41120	*cdc20::*P_{HSL1}*-CDC20-HphMX4 RIM4/RIM4-mNG::KlTRP1 CNM67-tdTomato::NatMX4 ama1Δ::KanMX4::AMA1-6A-LEU2 spo13Δ::HIS3MX6::spo13-mD::HphMX4*
7C	Z41121	*cdc20::*P_{HSL1}*-CDC20-HphMX4 RIM4/RIM4-mNG::KlTRP1 CNM67-tdTomato::NatMX4 ama1Δ::KanMX4::AMA1-6A-LEU2 leu2::*P_{DMC1}*-clb1-mDK-LEU2*
7C	Z41122	*cdc20::*P_{HSL1}*-CDC20-HphMX4 RIM4/RIM4-mNG::KlTRP1 CNM67-tdTomato::NatMX4 ama1Δ::KanMX4::AMA1-6A-LEU2 leu2::*P_{DMC1}*-clb1-mDK-LEU2 spo13Δ::HIS3MX6::spo13-mD::HphMX4*
7D	Z41131	*cdc20::*P_{HSL1}*-CDC20-HphMX4 RIM4/RIM4-mNG::KlTRP1 CNM67-tdTomato::NatMX4 ama1Δ::KanMX4::AMA1-6A-LEU2 cdc5-as::HphMX4*
7D	Z41040	*cdc20::*P_{HSL1}*-CDC20-HphMX4 RIM4/RIM4-mNG::KlTRP1 CNM67-tdTomato::NatMX4 ama1Δ::KanMX4::AMA1-6A-LEU2 ime2Δ::KanMX4::ime2-as-LEU2*
7D	Z41041	*cdc20::*P_{HSL1}*-CDC20-HphMX4 RIM4/RIM4-mNG::KlTRP1 CNM67-tdTomato::NatMX4 ama1Δ::KanMX4::AMA1-6A-LEU2 hrr25Δ::KanMX4::hrr25-as-HIS3*
8B	Z29055	*PDS1-myc18::KlTRP1 cdc20::*P_{SCC1}*-CDC20-KanMX6*
8B	Z32508	*PDS1-myc18::KlTRP1 cdc20::*P_{SCC1}*-CDC20-KanMX6 clb1Δ::NatMX4::clb1-12A-LEU2*
8B	Z32510	*PDS1-myc18::KlTRP1 cdc20::*P_{SCC1}*-CDC20-KanMX6 clb1Δ::NatMX4::clb1-12D-LEU2*
8B	Z32954	*PDS1-myc18::KlTRP1 cdc20::*P_{SCC1}*-CDC20-KanMX6 clb1Δ::NatMX4::clb1-12A-LEU2 ama1Δ::KanMX4*
8C	Z39726	*cdc20::*P_{HSL1}*-CDC20-HphMX4 RIM4/RIM4-mNG::KlTRP1 CNM67-tdTomato::NatMX4*
8C	Z39898	*cdc20::*P_{HSL1}*-CDC20-HphMX4 RIM4/RIM4-mNG::KlTRP1 CNM67-tdTomato::NatMX4 clb1Δ::NatMX4::clb1-12A-LEU2*
8C	Z39901	*cdc20::*P_{HSL1}*-CDC20-HphMX4 RIM4/RIM4-mNG::KlTRP1 CNM67-tdTomato::NatMX4 clb1Δ::NatMX4::clb1-12D-LEU2*
9A	Z40109	*ndt80Δ::NatMX4 cdc28-as1 leu2::*P_{DMC1}*-CLB1-ha6-LEU2*
9A	Z40110	*ndt80Δ::NatMX4 cdc28-as1 leu2::*P_{DMC1}*-CLB1-ha6-LEU2 ama1Δ::KanMX4*
9A	Z40111	*ndt80Δ::NatMX4 cdc28-as1 leu2::*P_{DMC1}*-clb1-12A-ha6-LEU2*
9A	Z40112	*ndt80Δ::NatMX4 cdc28-as1 leu2::*P_{DMC1}*-clb1-12A-ha6-LEU2 ama1Δ::KanMX4*
9A	Z40113	*ndt80Δ::NatMX4 cdc28-as1 leu2::*P_{DMC1}*-clb1-12D-ha6-LEU2*
9A	Z40114	*ndt80Δ::NatMX4 cdc28-as1 leu2::*P_{DMC1}*-clb1-12D-ha6-LEU2 ama1Δ::KanMX4*
9B	Z29054	*PDS1-myc18::KlTRP1 cdc20::*P_{SCC1}*-CDC20-KanMX6 spo13Δ::HIS3MX6*
9B	Z32507	*PDS1-myc18::KlTRP1 cdc20::*P_{SCC1}*-CDC20-KanMX6 spo13Δ::HIS3MX6 clb1Δ::NatMX4::clb1-12A-LEU2*
9B	Z32509	*PDS1-myc18::KlTRP1 cdc20::*P_{SCC1}*-CDC20-KanMX6 spo13Δ::HIS3MX6 clb1Δ::NatMX4::clb1-12D-LEU2*
9C	Z40508	*ndt80Δ::NatMX4 cdc28-as1 spo13Δ::HIS3MX6::SPO13::HphMX4*
9C	Z40509	*ndt80Δ::NatMX4 cdc28-as1 spo13Δ::HIS3MX6::SPO13::HphMX4 ama1Δ::CaURA3*
9C	Z40858	*ndt80Δ::NatMX4 cdc28-as1 mnd2Δ::KanMX4 ura3::*P_{DMC1}*-SPO13-URA3*

9C	Z40859	ndt80Δ::NatMX4 cdc28-as1 mnd2Δ::KanMX4 ura3::P_{DMC1}-SPO13-URA3 ama1Δ::CaURA3
EV1A	Z39139	cdc20::P_{HSL1}-CDC20-HphMX4 ama1$^{1-30}$::mNG-KlTRP1 CNM67-tdTomato::NatMX4
EV1A	Z39138	cdc20::P_{HSL1}-CDC20-HphMX4 ama1$^{1-30}$::mNG-KlTRP1 CNM67-tdTomato::NatMX4 spo13Δ::HIS3MX6
EV1B	Z40776	cdc20::P_{HSL1}-CDC20-HphMX4 ama1Δ::CaURA3 ssp2$^{1-15}$::mNG-KlTRP1 CNM67-tdTomato::NatMX4
EV1B	Z40777	cdc20::P_{HSL1}-CDC20-HphMX4 ama1Δ::CaURA3 ssp2$^{1-15}$::mNG-KlTRP1 CNM67-tdTomato::NatMX4 spo13Δ::HIS3MX6
EV1B	Z40683	cdc20::P_{HSL1}-CDC20-HphMX4 ama1Δ::CaURA3 sps4$^{1-15}$::mNG-KlTRP1 CNM67-tdTomato::NatMX4
EV1B	Z40684	cdc20::P_{HSL1}-CDC20-HphMX4 ama1Δ::CaURA3 sps4$^{1-15}$::mNG-KlTRP1 CNM67-tdTomato::NatMX4 spo13Δ::HIS3MX6
EV1B	Z40802	cdc20::P_{HSL1}-CDC20-HphMX4 ama1Δ::CaURA3 gip1$^{1-15}$::mNG-KlTRP1 CNM67-tdTomato::NatMX4
EV1B	Z40801	cdc20::P_{HSL1}-CDC20-HphMX4 ama1Δ::CaURA3 gip1$^{1-15}$::mNG-KlTRP1 CNM67-tdTomato::NatMX4 spo13Δ::HIS3MX6
EV1B	Z40866	cdc20::P_{HSL1}-CDC20-HphMX4 ama1Δ::CaURA3 gat4$^{1-15}$::mNG-KlTRP1 CNM67-tdTomato::NatMX4
EV1B	Z40867	cdc20::P_{HSL1}-CDC20-HphMX4 ama1Δ::CaURA3 gat4$^{1-15}$::mNG-KlTRP1 CNM67-tdTomato::NatMX4 spo13Δ::HIS3MX6
EV1C	Z38116	cdc20::P_{HSL1}-CDC20-HphMX4 ama1Δ::CaURA3 RIM4/RIM4-mNG::KlTRP1 CNM67-tdTomato::NatMX4
EV1C	Z38115	cdc20::P_{HSL1}-CDC20-HphMX4 ama1Δ::CaURA3 RIM4/RIM4-mNG::KlTRP1 CNM67-tdTomato::NatMX4 spo13Δ::HIS3MX6
EV1D	Z35540	PDS1-myc18::KlTRP1 cdc20::P_{SCC1}-CDC20-KanMX4 ama1Δ::NatMX4 ura3::P_{GPD}-GAL4^{484}-ER-URA3 leu2::P_{GALL}-cAMA1-LEU2
EV1D	Z35539	PDS1-myc18::KlTRP1 cdc20::P_{SCC1}-CDC20-KanMX4 ama1Δ::NatMX4 ura3::P_{GPD}-GAL4^{484}-ER-URA3 leu2::P_{GALL}-cAMA1-LEU2 spo13Δ::HIS3MX6
EV2A	Z11761	CDC14/CDC14-eGFP::LEU2 his3/his3::P_{HIS3}-eGFP-TUB1-HIS3 ura3/ura3::tetO$_{224}$::URA3 leu2/leu2::P_{URA3}-tetR-tdTomato::LEU2 spo13Δ::HIS3MX6::SPO13::HphMX4
EV2A	Z12158	CDC14/CDC14-eGFP::LEU2 his3/his3::P_{HIS3}-eGFP-TUB1-HIS3 ura3/ura3::tetO$_{224}$::URA3 leu2/leu2::P_{URA3}-tetR-tdTomato::LEU2 spo13Δ::HIS3MX6::spo13-m2::HphMX4
EV2B	Z33285	PDS1-myc18::KlTRP1 cdc20::P_{HSL1}-CDC20-HphMX4
EV2B	Z38409	PDS1-myc18::KlTRP1 cdc20::P_{HSL1}-CDC20-HphMX4 ama1Δ::KanMX4
EV2B	Z38457	PDS1-myc18::KlTRP1 cdc20::P_{HSL1}-CDC20-HphMX4 spo13Δ::HIS3MX6::spo13-m2::HphMX4
EV2B	Z38456	PDS1-myc18::KlTRP1 cdc20::P_{HSL1}-CDC20-HphMX4 spo13Δ::HIS3MX6::spo13-m2::HphMX4 ama1Δ::KanMX4
EV2C	Z39986	cdc20::P_{HSL1}-CDC20-HphMX4 RIM4/RIM4-mNG::KlTRP1 CNM67-tdTomato::NatMX4 spo13Δ::HIS3MX6::spo13-m2::HphMX4
EV2C	Z39849	cdc20::P_{HSL1}-CDC20-HphMX4 RIM4/RIM4-mNG::KlTRP1 CNM67-tdTomato::NatMX4 cdc5-as::HphMX4
EV2D	Z41292	cdc20::P_{HSL1}-CDC20-HphMX4 his3::P_{GPD}-GAL4^{484}-ER-HIS3
EV2E	Z41293	cdc20::P_{HSL1}-CDC20-HphMX4 his3::P_{GPD}-GAL4^{484}-ER-HIS3 ura3::P_{GAL1}-SPO13-URA3
EV3A	Z38288	cdc20::P_{HSL1}-CDC20-HphMX4 ama1Δ::CaURA3 RIM4-mNG::KlTRP1 CNM67-tdTomato::NatMX4 hrr25Δ::KanMX4::HRR25-HIS3

EV3A	Z38286	*cdc20::*P_{HSL1}*-CDC20-HphMX4 ama1Δ::CaURA3 RIM4-mNG::KlTRP1 CNM67-tdTomato::NatMX4 hrr25Δ::KanMX4::HRR25-HIS3 spo13Δ::HIS3MX6*
EV3A	Z38285	*cdc20::*P_{HSL1}*-CDC20-HphMX4 ama1Δ::CaURA3 RIM4-mNG::KlTRP1 CNM67-tdTomato::NatMX4 hrr25Δ::KanMX4::hrr25-as-HIS3 spo13Δ::HIS3MX6*
EV3B	Z38287	*cdc20::*P_{HSL1}*-CDC20-HphMX4 ama1Δ::CaURA3 RIM4-mNG::KlTRP1 CNM67-tdTomato::NatMX4 hrr25Δ::KanMX4::hrr25-as-HIS3*
EV3B	Z38284	*cdc20::*P_{HSL1}*-CDC20-HphMX4 ama1Δ::CaURA3 RIM4-mNG::KlTRP1 CNM67-tdTomato::NatMX4 hrr25Δ::KanMX4::HRR25-HIS3 ime2-ΔC-ha3::HIS3MX6*
EV3B	Z38283	*cdc20::*P_{HSL1}*-CDC20-HphMX4 ama1Δ::CaURA3 RIM4-mNG::KlTRP1 CNM67-tdTomato::NatMX4 hrr25Δ::KanMX4::hrr25-as-HIS3 ime2-ΔC-ha3::HIS3MX6*
EV3C	Z40505	*cdc20::*P_{HSL1}*-CDC20-HphMX4 ama1Δ::KanMX4 RIM4-mNG::KlTRP1 CNM67-tdTomato::NatMX4 hrr25-ΔC-ha3::HIS3MX6*
EV3C	Z40606	*cdc20::*P_{HSL1}*-CDC20-HphMX4 ama1Δ::KanMX4 RIM4-mNG::KlTRP1 CNM67-tdTomato::NatMX4 HRR25-ha3::HIS3MX6 ime2Δ::KanMX4::ime2-as-LEU2*
EV3C	Z40582	*cdc20::*P_{HSL1}*-CDC20-HphMX4 ama1Δ::KanMX4 RIM4-mNG::KlTRP1 CNM67-tdTomato::NatMX4 hrr25-ΔC-ha3::HIS3MX6 ime2Δ::KanMX4::ime2-as-LEU2*
EV3D	Z41206	*cdc20::*P_{HSL1}*-CDC20-HphMX4 RIM4-mNG::KlTRP1*
EV3D	Z41207	*cdc20::*P_{HSL1}*-CDC20-HphMX4 RIM4-mNG::KlTRP1 HRR25-myc9::HIS3MX6*
EV4A	Z3539	*CLB1-myc9::KlTRP1*
EV4A	Z5155	*CLB3-myc9::HIS3MX6*
EV4A	Z4124	*CLB4-myc9::KlTRP1*
EV4A	Z3289	*CLB5-myc9-URA3*
EV4A	Z4125	*CLB6-myc9::KlTRP1*
EV4B	Z5312	*leu2::*P_{DMC1}*-CLB1-ha3-LEU2*
EV4B	Z5923	*leu2::*P_{DMC1}*-clb1-mDK-ha3-LEU2*
EV4C	Z3539	*CLB1-myc9::KlTRP1*
EV4C	Z3542	*CLB1-myc9::KlTRP1 ama1Δ::KanMX4*
EV4D	Z33959	*cdc20::*P_{HSL1}*-CDC20-HphMX4 ura3::*P_{CUP1}*-CDC20-URA3 ESP1-eGFP::HIS3MX6*
EV4D	Z33960	*cdc20::*P_{HSL1}*-CDC20-HphMX4 ura3::*P_{CUP1}*-CDC20-URA3 AMA1-eGFP::KlTRP1*
EV4E	Z32630	*CLB1-ha3::KlURA3*
EV4E	Z32629	*CLB1-ha3::KlURA3 AMA1-myc9::KlTRP1*
EV5A	Z32630	*CLB1-ha3::KlURA3*
EV5A	Z32605	*CLB1-ha3::KlURA3 CDC5-myc15-URA3*
EV5A	Z32604	*CLB1-ha3::KlURA3 CDC5-myc15-URA3 spo13Δ::HIS3MX6*
EV5B	Z32666	*CDC14/CDC14-eGFP::LEU2 ura3/ura3::*P_{TUB1}*-eGFP-TUB1-URA3 CEN5/CEN5::*$tetO_{224}$*::HIS3 leu2/leu2::*P_{URA3}*-tetR-tdTomato::LEU2 clb1Δ::NatMX4::clb1-12A::LEU2*
EV5B	Z32664	*CDC14/CDC14-eGFP::LEU2 ura3/ura3::*P_{TUB1}*-eGFP-TUB1-URA3 CEN5/CEN5::*$tetO_{224}$*::HIS3 leu2/leu2::*P_{URA3}*-tetR-tdTomato::LEU2 clb1Δ::NatMX4::clb1-12D::LEU2*
EV5C	Z39898	*cdc20::*P_{HSL1}*-CDC20-HphMX4 RIM4/RIM4-mNG::KlTRP1 CNM67-tdTomato::NatMX4 clb1Δ::NatMX4::clb1-12A::LEU2*
EV5C	Z39728	*cdc20::*P_{HSL1}*-CDC20-HphMX4 RIM4/RIM4-mNG::KlTRP1 CNM67-tdTomato::NatMX4 clb1Δ::BleMX4::clb1-6A::LEU2*
EV5D	Z40170	*cdc20::*P_{HSL1}*-CDC20-HphMX4 ama1Δ::CaURA3 RIM4/RIM4-mNG::KlTRP1 CNM67-tdTomato::NatMX4 clb1Δ::NatMX4::clb1-12A::LEU2*
EV5D	Z39213	*cdc20::*P_{HSL1}*-CDC20-HphMX4 ama1Δ::CaURA3 RIM4/RIM4-mNG::KlTRP1 CNM67-tdTomato::NatMX4 clb1Δ::BleMX4*
S1A	Z41151	*cdc20::*P_{HSL1}*-CDC20-HphMX4 CDC14/CDC14-eGFP::LEU2 ura3/ura3::*P_{TUB1}*-eGFP-TUB1-URA3 HTB1/HTB1-mCherry::HIS3MX6*

S1A	Z41152	*cdc20::*P_{HSL1}*-CDC20-HphMX4 CDC14/CDC14-eGFP::LEU2 ura3/ura3::*P_{TUB1}*-eGFP-TUB1-URA3 HTB1/HTB1-mCherry::HIS3MX6 spo13Δ::BleMX4*
S1B	Z35520	*CDC14/CDC14-eGFP::LEU2 ura3/ura3::*P_{TUB1}*-eGFP-TUB1-URA3 CEN5/CEN5::*$tetO_{224}$*::HIS3 leu2/leu2::*P_{URA3}*-tetR-tdTomato::LEU2 mad2Δ::KlTRP1 spo13Δ::HIS3MX6 rec8Δ::KanMX4::REC8-ha3::LEU2*
S1B	Z35519	*CDC14/CDC14-eGFP::LEU2 ura3/ura3::*P_{TUB1}*-eGFP-TUB1-URA3 CEN5/CEN5::*$tetO_{224}$*::HIS3 leu2/leu2::*P_{URA3}*-tetR-tdTomato::LEU2 mad2Δ::KlTRP1 spo13Δ::HIS3MX6 rec8Δ::KanMX4::rec8-18D-ha3::LEU2*
S1C	Z32651	*CDC14/CDC14-eGFP::LEU2 ura3/ura3::*P_{TUB1}*-eGFP-TUB1-URA3 CEN5/CEN5::*$tetO_{224}$*::HIS3 leu2/leu2::*P_{URA3}*-tetR-tdTomato::LEU2 mpc54Δ::AurCMX4 mpc70Δ::BleMX4*
S1C	Z32650	*CDC14/CDC14-eGFP::LEU2 ura3/ura3::*P_{TUB1}*-eGFP-TUB1-URA3 CEN5/CEN5::*$tetO_{224}$*::HIS3 leu2/leu2::*P_{URA3}*-tetR-tdTomato::LEU2 mpc54Δ::AurCMX4 mpc70Δ::BleMX4 spo13Δ::HIS3MX6*
S2A	Z32563	*PDS1-myc18::KlTRP1 cdc20::*P_{SCC1}*-CDC20-KanMX6*
S2A	Z32562	*PDS1-myc18::KlTRP1 cdc20::*P_{SCC1}*-CDC20-KanMX6 spo13Δ::HIS3MX6*
S2A	Z32561	*PDS1-myc18::KlTRP1 cdc20::*P_{SCC1}*-CDC20-KanMX6 cdh1::*P_{HSL1}*-CDH1-HphMX4*
S2A	Z32560	*PDS1-myc18::KlTRP1 cdc20::*P_{SCC1}*-CDC20-KanMX6 cdh1::*P_{HSL1}*-CDH1-HphMX4 spo13Δ::HIS3MX6*
S2B	Z29055	*PDS1-myc18::KlTRP1 cdc20::*P_{SCC1}*-CDC20-KanMX6*
S2B	Z29054	*PDS1-myc18::KlTRP1 cdc20::*P_{SCC1}*-CDC20-KanMX6 spo13Δ::HIS3MX6*
S2C	Z40205	*CDC5-eGFP::KlTRP1 CNM67-tdTomato::NatMX4*
S2C	Z40206	*CDC5-eGFP::KlTRP1 CNM67-tdTomato::NatMX4 spo13Δ::HIS3MX6::spo13-mD::HphMX4*
S3A	Z39139	*cdc20::*P_{HSL1}*-CDC20-HphMX4* $ama1^{1-30}$*::mNG-KlTRP1 CNM67-tdTomato::NatMX4*
S3A	Z39343	*cdc20::*P_{HSL1}*-CDC20-HphMX4* $ama1^{1-30}$*::mNG-KlTRP1 CNM67-tdTomato::NatMX4 ime2-ΔC-ha3::HIS3MX6*
S3B	Z21085	*SPC42-eGFP::HIS3MX6 HTB1-mCherry::HIS3MX6*
S3B	Z21084	*SPC42-eGFP::HIS3MX6 HTB1-mCherry::HIS3MX6 hrr25-ΔC::KlTRP1*
S3C	Z41057	*cdc20::*P_{HSL1}*-CDC20-HphMX4* $ama1^{1-30}$*::mNG-KlTRP1 CNM67-tdTomato::NatMX4 HRR25-ha3::HIS3MX6*
S3C	Z41059	*cdc20::*P_{HSL1}*-CDC20-HphMX4* $ama1^{1-30}$*::mNG-KlTRP1 CNM67-tdTomato::NatMX4 hrr25-ΔC-ha3::HIS3MX6*
S4	Z21260	*cdc20::*P_{CLB2}*-CDC20-KanMX6 trp1::*P_{CUP1}*-CDC20-TRP1 PDS1-myc18::HIS3MX6*
S4	Z21430	*cdc20::*P_{CLB2}*-CDC20-KanMX6 trp1::*P_{CUP1}*-CDC20-TRP1 PDS1-myc18::HIS3MX6 cdc5-as::HphMX4*
S4	Z33526	*cdc20::*P_{CLB2}*-CDC20-HphMX4 ura3::*P_{CUP1}*-CDC20-URA3 PDS1-myc18::KlTRP1 ime2Δ::KanMX4::ime2-as-LEU2*
S4	Z21582	*cdc20::*P_{CLB2}*-CDC20-KanMX6 trp1::*P_{CUP1}*-CDC20-TRP1 PDS1-myc18::HIS3MX6 hrr25Δ::KanMX4::hrr25-as-HIS3*
S5A	Z24864	*CDC14/CDC14-eGFP::LEU2 ura3/ura3::*P_{TUB1}*-eGFP-TUB1-URA3 CEN5/CEN5::*$tetO_{224}$*::HIS3 leu2/leu2::*P_{URA3}*-tetR-tdTomato::LEU2*
S5A	Z31842	*CDC14/CDC14-eGFP::LEU2 ura3/ura3::*P_{TUB1}*-eGFP-TUB1-URA3 CEN5/CEN5::*$tetO_{224}$*::HIS3 leu2/leu2::*P_{URA3}*-tetR-tdTomato::LEU2 clb1Δ::NatMX4*
S5B	Z31842	*CDC14/CDC14-eGFP::LEU2 ura3/ura3::*P_{TUB1}*-eGFP-TUB1-URA3 CEN5/CEN5::*$tetO_{224}$*::HIS3 leu2/leu2::*P_{URA3}*-tetR-tdTomato::LEU2 clb1Δ::NatMX4*
S5C	Z39213	*cdc20::*P_{HSL1}*-CDC20-HphMX4 ama1Δ::CaURA3 RIM4/RIM4-mNG::KlTRP1 CNM67-tdTomato::NatMX4 clb1Δ::BleMX4*

S5C	Z39574	*cdc20::P_{HSL1}-CDC20-HphMX4 ama1Δ::CaURA3 RIM4/RIM4-mNG::KlTRP1 CNM67-tdTomato::NatMX4 cdc28-as2*
S5C	Z39491	*cdc20::P_{HSL1}-CDC20-HphMX4 ama1Δ::CaURA3 RIM4/RIM4-mNG::KlTRP1 CNM67-tdTomato::NatMX4 spo13Δ::HIS3MX6::spo13-10A::HphMX4*
S5D	Z41403	*cdc20::P_{HSL1}-CDC20-HphMX4 ama1Δ::CaURA3 RIM4/RIM4-mNG::KlTRP1 CNM67-tdTomato::NatMX4 clb1Δ::BleMX4*
S5D	Z41404	*cdc20::P_{HSL1}-CDC20-HphMX4 ama1Δ::CaURA3 RIM4/RIM4-mNG::KlTRP1 CNM67-tdTomato::NatMX4 clb3Δ::TRP1*
S5D	Z41405	*cdc20::P_{HSL1}-CDC20-HphMX4 ama1Δ::CaURA3 RIM4/RIM4-mNG::KlTRP1 CNM67-tdTomato::NatMX4 clb1Δ::BleMX4 clb3Δ::TRP1*
S6A	Z33959	*cdc20::P_{HSL1}-CDC20-HphMX4 ura3::P_{CUP1}-CDC20-URA3 ESP1-eGFP::HIS3MX6*
S6A	Z33961	*cdc20::P_{HSL1}-CDC20-HphMX4 ura3::P_{CUP1}-CDC20-URA3 AMA1-eGFP::KlTRP1 spo13Δ::HIS3MX6*
S6B	Z32630	*CLB1-ha3::KlURA3*
S6B	Z32629	*CLB1-ha3::KlURA3 AMA1-myc9::KlTRP1*
S6B	Z32628	*CLB1-ha3::KlURA3 AMA1-myc9::KlTRP1 spo13Δ::HIS3MX6*
S6C	Z19064	*ndt80Δ::HIS3 PDS1-myc18::KlTRP1 ura3::P_{GPD}-$GAL4^{484}$-ER-URA3 leu2::P_{GAL1}-CLB1-LEU2 spo13Δ::HIS3MX6*
S6D	Z19478	*ndt80Δ::NatMX4 PDS1-myc18::KlTRP1 his3::P_{GPD}-$GAL4^{484}$-ER-HIS3 ura3::P_{GAL1}-CLB3-ha3-URA3*
S6E	Z40123	*cdc20::P_{HSL1}-CDC20-HphMX4 RIM4/RIM4-mNG::KlTRP1 CNM67-tdTomato::NatMX4 ama1Δ::CaURA3::AMA1-LEU2*
S6E	Z40124	*cdc20::P_{HSL1}-CDC20-HphMX4 RIM4/RIM4-mNG::KlTRP1 CNM67-tdTomato::NatMX4 ama1Δ::KanMX4::AMA1-6A-LEU2*
S6E	Z40209	*cdc20::P_{HSL1}-CDC20-HphMX4 RIM4/RIM4-mNG::KlTRP1 CNM67-tdTomato::NatMX4 ama1Δ::CaURA3::AMA1-LEU2 acm1Δ::KanMX4*
S6E	Z40210	*cdc20::P_{HSL1}-CDC20-HphMX4 RIM4/RIM4-mNG::KlTRP1 CNM67-tdTomato::NatMX4 ama1Δ::KanMX4::AMA1-6A-LEU2 acm1Δ::KanMX4*
S7A	Z32115	*cdc20::P_{SCC1}-CDC20-HphMX4 ama1Δ::NatMX4 PDS1-eGFP::KanMX4*
S7A	Z32114	*cdc20::P_{SCC1}-CDC20-HphMX4 ama1Δ::NatMX4 CLB1-eGFP::KlTRP1*
S7A	Z32113	*cdc20::P_{SCC1}-CDC20-HphMX4 ama1Δ::NatMX4 CLB1-eGFP::KlTRP1 spo13Δ::BleMX4*
S7A	Z32112	*cdc20::P_{SCC1}-CDC20-HphMX4 ama1Δ::NatMX4 CLB1-eGFP::KlTRP1 cdc5::P_{SCC1}-CDC5-KanMX4*
S8A	Z27121	*ndt80Δ::NatMX4 leu2::P_{GAL1}-NDT80-LEU2 his3::P_{GPD}-$GAL4^{484}$-ER-HIS3*
S8A	Z31663	*ndt80Δ::NatMX4 leu2::P_{GAL1}-NDT80-LEU2 his3::P_{GPD}-$GAL4^{484}$-ER-HIS3 CLB1-eGFP::KlTRP1*
S8A	Z31662	*ndt80Δ::NatMX4 leu2::P_{GAL1}-NDT80-LEU2 his3::P_{GPD}-$GAL4^{484}$-ER-HIS3 CLB1-eGFP::KlTRP1 spo13Δ::BleMX4*
S9A, S9C	Z41125	*CDC14/CDC14-eGFP::LEU2 ura3/ura3::P_{TUB1}-eGFP-TUB1-URA3 HTB1/HTB1-mCherry::HIS3MX6*
S9A, S9C	Z41711	*CDC14/CDC14-eGFP::LEU2 ura3/ura3::P_{TUB1}-eGFP-TUB1-URA3 HTB1/HTB1-mCherry::HIS3MX6 spo13Δ::BleMX4*
S9A, S9C	Z41714	*CDC14/CDC14-eGFP::LEU2 ura3/ura3::P_{TUB1}-eGFP-TUB1-URA3 HTB1/HTB1-mCherry::HIS3MX6 spo13Δ::BleMX4 ama1Δ::CaURA3*
S9B, S9D	Z41712	*NDT80-AID*::KanMX6 CDC14/CDC14-eGFP::LEU2 ura3/ura3::P_{TUB1}-eGFP-TUB1-URA3 HTB1/HTB1-mCherry::HIS3MX6*

S9B, S9D	Z41713	*NDT80-AID*::KanMX6 CDC14/CDC14-eGFP::LEU2 ura3/ura3::*P_{TUB1}*-eGFP-TUB1-URA3 HTB1/HTB1-mCherry::HIS3MX6 spo13Δ::BleMX4*
S9B, S9D	Z41715	*NDT80-AID*::KanMX6 CDC14/CDC14-eGFP::LEU2 ura3/ura3::*P_{TUB1}*-eGFP-TUB1-URA3 HTB1/HTB1-mCherry::HIS3MX6 spo13Δ::BleMX4 ama1Δ::CaURA3*
S10	Z3933	*ura3/ura3::*$tetO_{224}$*::URA3 leu2/leu2::*P_{URA3}*-tetR-tdTomato::LEU2 ndt80Δ::HIS3*
S10	Z3932	*ura3/ura3::*$tetO_{224}$*::URA3 leu2/leu2::*P_{URA3}*-tetR-tdTomato::LEU2 ndt80Δ::HIS3 mnd2Δ::KanMX4*
S10	Z4136	*ura3/ura3::*$tetO_{224}$*::URA3 leu2/leu2::*P_{URA3}*-tetR-tdTomato::LEU2 ndt80Δ::HIS3 mnd2Δ::KanMX4 ama1Δ::KanMX4*

[1]Strains are listed for each figure from left to right and/or top to bottom. [2]All SK1 strains are diploid with the background *MATa/MATα ho::LYS2 lys2 ade2Δ::hisG trp1Δ::hisG leu2Δ::hisG his3Δ::hisG ura3*. Mutations are homozygous unless stated otherwise.
