# Supplementary material for: Spo13/MEIKIN ensures a Two‐Division meiosis by preventing the activation of APC/CAma1 at meiosis I
Source: EMBO J. 2023 Sep 20;42(20):e114288. doi: 10.15252/embj.2023114288 (PMC10577557; doi:10.15252/embj.2023114288)
Supplement: Supplementary file 2 — Expanded View Figures PDF [file EMBJ-42-e114288-s005.pdf]

Expanded View Figures

Figure EV1. Regulation of translation by Spo13.

A Deletion of *SPO13* causes *AMA1* translation in Cdc20-depleted cells. Top, time-lapse series from the imaging of mNG and SPBs (Cnm67-Tomato) in P_{HSL1}-*CDC20* control and *spo13Δ* cells carrying *ama1*$^{1-30}$*-mNG* in place of *AMA1*. Bottom, the presence of mNG quantified in cells synchronized *in silico* to SPB separation at metaphase I (t = 0).

B Sporulation genes showing Spo13-dependent translational repression in metaphase I-arrested P_{HSL1}-*CDC20 ama1Δ* strains. Top, time-lapse series from the imaging of mNG and SPBs (Cnm67-Tomato) in control and *spo13Δ* cells carrying *gip1*$^{1-15}$*-mNG* in place of *GIP1*. Bottom, the presence of mNG quantified in cells in which *mNG* replaces coding sequences downstream of codon 15 of *GAT4*, *GIP1*, *SPS4*, or *SSP2*. Cells have been synchronized *in silico* to SPB separation at metaphase I (t = 0).

C Deletion of *SPO13* causes Rim4 degradation in metaphase I-arrested P_{HSL1}-*CDC20 ama1Δ* cells. Top, time-lapse series from the imaging of Rim4-mNG and SPBs (Cnm67-Tomato). Bottom, the presence of Rim4-mNG quantified in cells synchronized *in silico* to SPB separation at metaphase I (t = 0).

D Deletion of *SPO13* causes degradation of APC/C substrates and nuclear division in Cdc20-depleted (P_{SCC1}-*CDC20*) cells expressing *AMA1* from an estradiol-inducible *GAL* promoter (P_{EST}-*AMA1*) at t = 7 h in SPM (arrows). Left, immunoblot detection of proteins. Right, progression of meiosis quantified in fixed cells.

Data information: Data are representative of three (A and C) or two (B) independent experiments. Scale bar, 4 μm.
Source data are available online for this figure.

 © 2023 The Authors

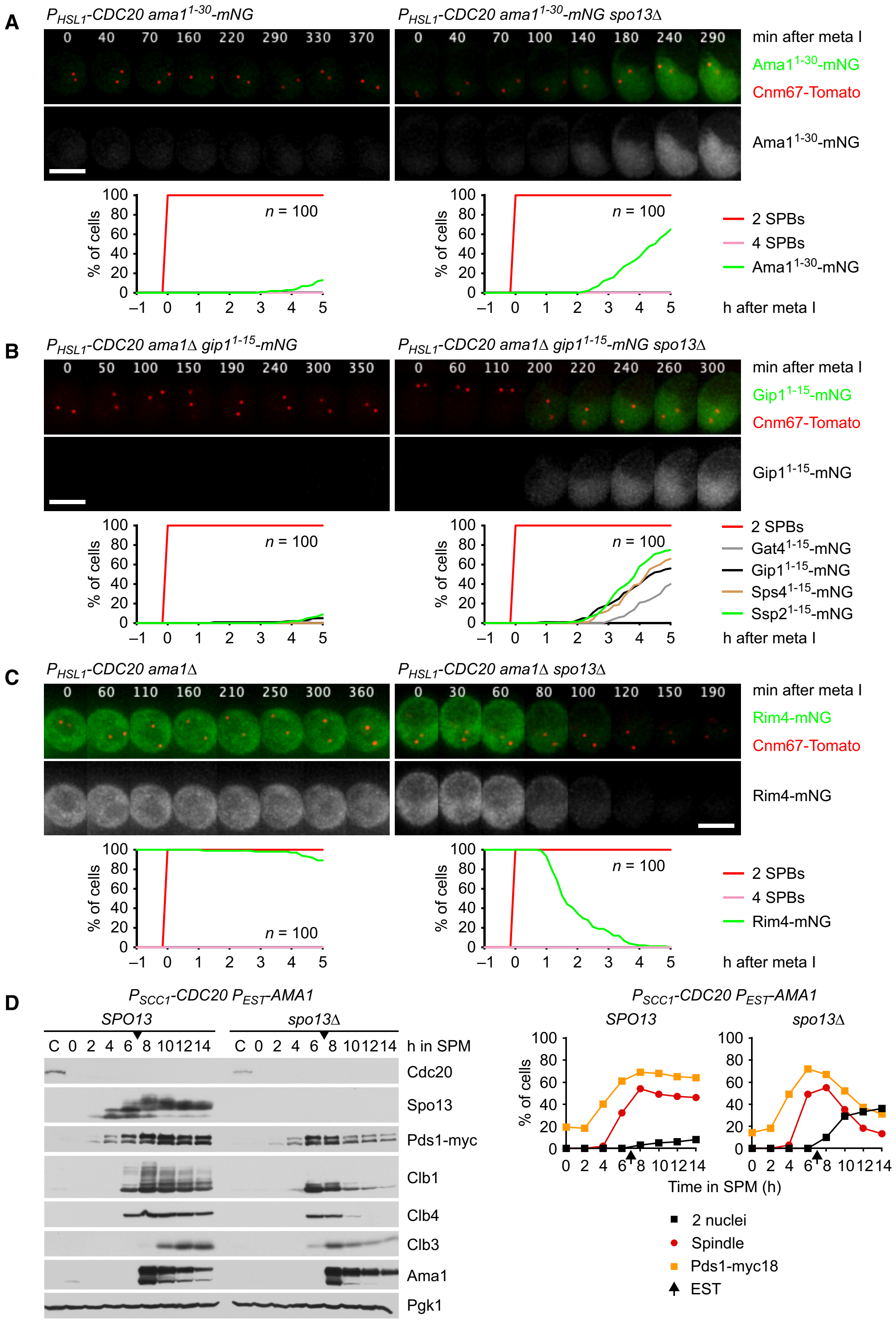
A
P_{HSL1}-CDC20 ama1$^{1-30}$-mNG
P_{HSL1}-CDC20 ama1$^{1-30}$-mNG spo13Δ
min after meta I
Ama1$^{1-30}$-mNG
Cnm67-Tomato
n = 100
2 SPBs
4 SPBs
% of cells
h after meta I
B
P_{HSL1}-CDC20 ama1Δ gip1$^{1-15}$-mNG
P_{HSL1}-CDC20 ama1Δ gip1$^{1-15}$-mNG spo13Δ
Gip1$^{1-15}$-mNG
Gat4$^{1-15}$-mNG
Sps4$^{1-15}$-mNG
Ssp2$^{1-15}$-mNG
C
P_{HSL1}-CDC20 ama1Δ
P_{HSL1}-CDC20 ama1Δ spo13Δ
Rim4-mNG
D
P_{SCC1}-CDC20 P_{EST}-AMA1
SPO13
spo13Δ
h in SPM
Cdc20
Spo13
Pds1-myc
Clb1
Clb4
Clb3
Ama1
Pgk1
Time in SPM (h)
2 nuclei
Spindle
Pds1-myc18
EST

Figure EV1.

Figure EV2. Analysis of the *spo13-m2 mutation*.

A Meiosis in control and *spo13-m2* cells. Top, imaging of spindles (GFP-tubulin), nucleolar release of Cdc14-GFP, and TetR-Tomato, which labels the nucleoplasm (diffuse signal) and the *URA3* locus of one copy of chromosome V (dots). First (blue) and second (green) Cdc14 release are marked. Bottom, meiotic events quantified in cells synchronized *in silico* to spindle formation at metaphase I ($t = 0$). *spo13-m2* prolongs metaphase I to 60 ± 26 min (control, 23 ± 6 min; $P < 0.0001$, Welch's t-test) and causes ~38% of cells to undergo a single round of spindle formation and Cdc14 release.

B *spo13-m2* causes Ama1-dependent proteolysis and nuclear division in P_{HSL1}-*CDC20* cells. Top, immunoblot detection of proteins. Bottom, progression of meiosis quantified in fixed cells.

C Imaging of Rim4-mNG and SPBs (Cnm67-Tomato) in P_{HSL1}-*CDC20* cells carrying *spo13-m2* or *cdc5-as*. Cdc5-as was inhibited with CMK at 7.5 h in SPM. Top, time-lapse series. Bottom, the presence of Rim4-mNG quantified in cells synchronized *in silico* to SPB separation at metaphase I ($t = 0$).

D, E Extracts from metaphase I-arrested P_{HSL1}-*CDC20* cells (8 h in SPM) were applied to columns carrying no antibody or antibodies to either Spo13 or Cdc5. Flow-throughs were analyzed by immunoblotting. (D) Depletion of Spo13 from extracts of control cells (*SPO13*) removes little Cdc5 (10%). (E) Depletion of Spo13 from extracts of cells overexpressing Spo13 (P_{EST}-*SPO13*, estradiol for 45 min) removes most of Cdc5 (97%).

Data information: (B–D) are representative of two independent experiments. Scale bar, 4 μm.
Source data are available online for this figure.

© 2023 The Authors

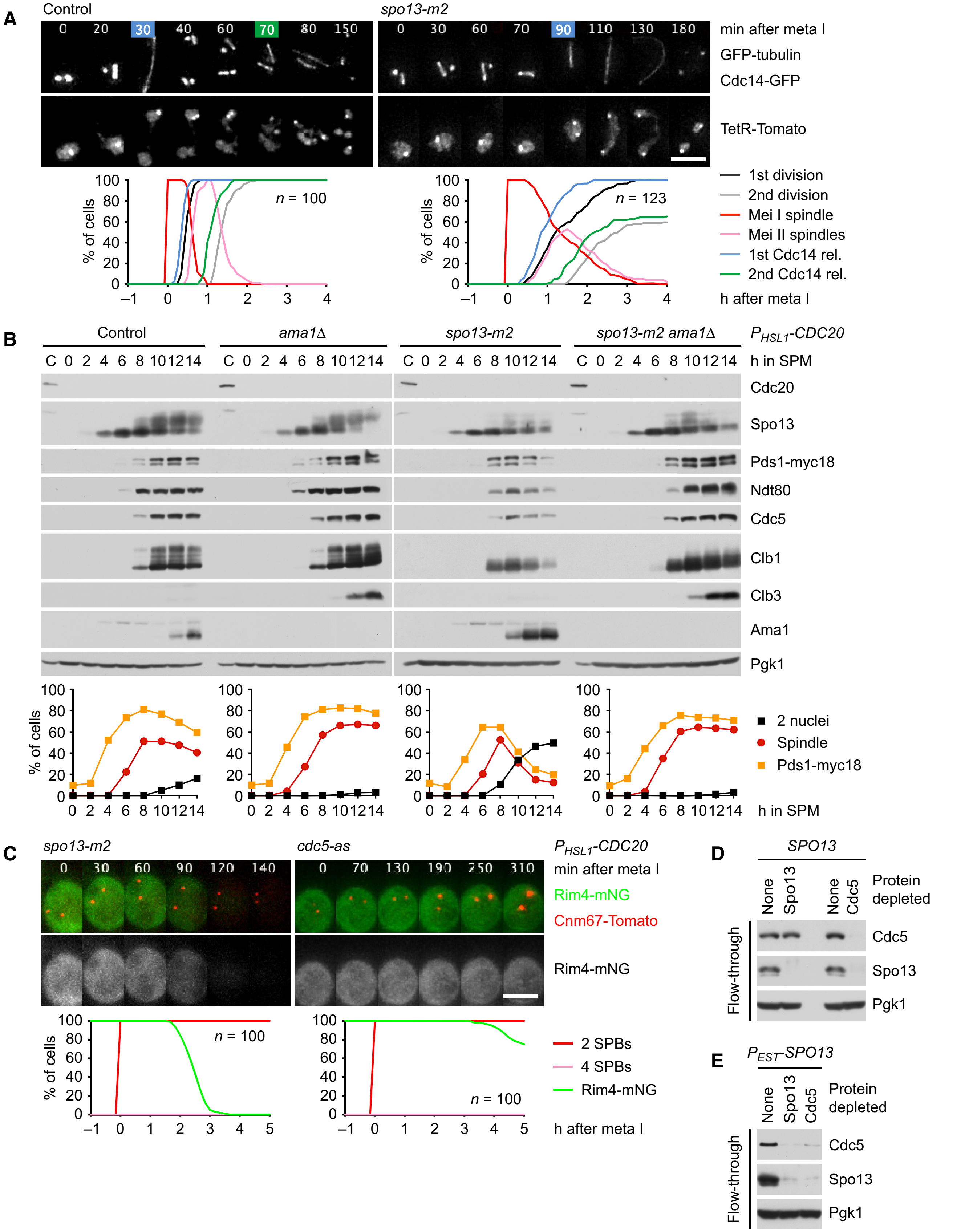
A
Control
spo13-m2
min after meta I
GFP-tubulin
Cdc14-GFP
TetR-Tomato
% of cells
n = 100
n = 123
1st division
2nd division
Mei I spindle
Mei II spindles
1st Cdc14 rel.
2nd Cdc14 rel.
h after meta I
B
Control
ama1Δ
spo13-m2
spo13-m2 ama1Δ
PHSL1-CDC20
h in SPM
Cdc20
Spo13
Pds1-myc18
Ndt80
Cdc5
Clb1
Clb3
Ama1
Pgk1
2 nuclei
Spindle
Pds1-myc18
C
spo13-m2
cdc5-as
PHSL1-CDC20
min after meta I
Rim4-mNG
Cnm67-Tomato
Rim4-mNG
n = 100
2 SPBs
4 SPBs
Rim4-mNG
h after meta I
D
SPO13
None
Spo13
None
Cdc5
Protein depleted
Flow-through
Cdc5
Spo13
Pgk1
E
PEST-SPO13
None
Spo13
Cdc5
Protein depleted
Flow-through
Cdc5
Spo13
Pgk1

Figure EV2.

Figure EV3. Regulation of Rim4 degradation by Hrr25.

A–C Imaging of Rim4-mNG and SPBs (Cnm67-Tomato) in metaphase I-arrested P_{HSL1}-*CDC20 ama1Δ* strains. Top, time-lapse series. Bottom, the presence of mNG quantified in cells synchronized *in silico* to SPB separation at metaphase I ($t = 0$). (A) Inhibition of Hrr25 activity prevents Rim4 degradation elicited by the *SPO13* deletion. Hrr25-as was inhibited with 1NM-PP1 at 7 h in SPM. (B) Inhibition of Hrr25 prevents Rim4 degradation induced by hyperactive Ime2-ΔC. Hrr25-as was inhibited with 1NM-PP1 at 7 h in SPM. (C) Rim4 is degraded with similar timing in *hrr25-ΔC* cells with active or inactive Ime2 ($P = 0.91$, Welch's *t*-test). Ime2-as was inhibited with 1Na-PP1 at 7 h in SPM.

D Binding of Rim4-mNG and Ime2 to Hrr25-myc9 immunoprecipitated with α-Myc antibodies from extracts of P_{HSL1}-*CDC20* cells.

Data information: Data are representative of two (A and B) or three (C) independent experiments. Scale bar, 4 μm.
Source data are available online for this figure.

 © 2023 The Authors

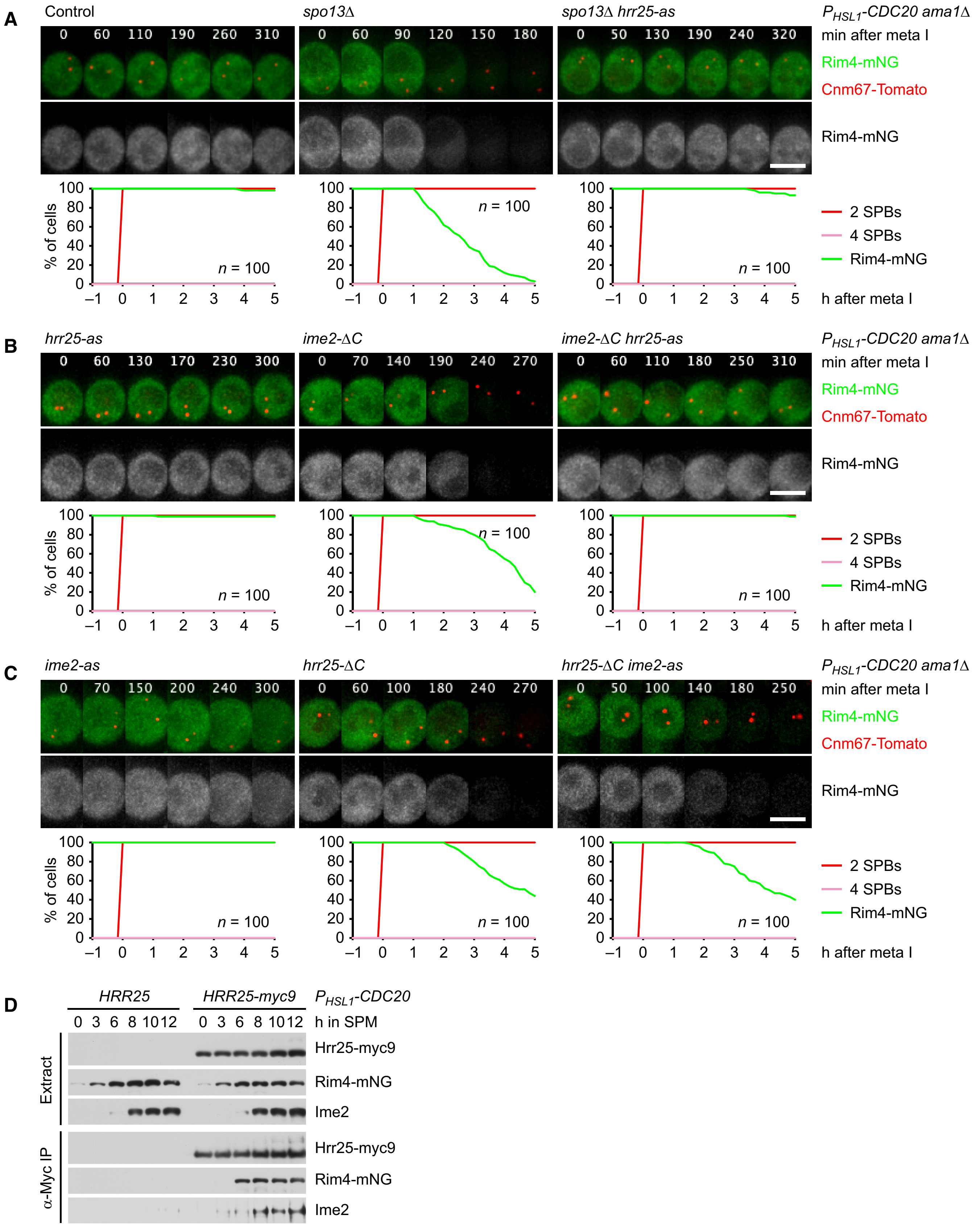
A
Control
spo13Δ
spo13Δ hrr25-as
P_{HSL1}-CDC20 ama1Δ
min after meta I
Rim4-mNG
Cnm67-Tomato
Rim4-mNG
% of cells
n = 100
2 SPBs
4 SPBs
Rim4-mNG
h after meta I
B
hrr25-as
ime2-ΔC
ime2-ΔC hrr25-as
C
ime2-as
hrr25-ΔC
hrr25-ΔC ime2-as
D
HRR25
HRR25-myc9
P_{HSL1}-CDC20
0 3 6 8 10 12
h in SPM
Extract
α-Myc IP
Hrr25-myc9
Rim4-mNG
Ime2

Figure EV3.

Figure EV4. Identification of proteins copurifying with Ama1.

A–C Binding of cyclins to the APC/C immunoprecipitated with α-Apc2 antibodies. (A) Clb1 and Clb3 but not the other Clbs copurify with the APC/C. (B) The degrons of Clb1 are not required for binding to the APC/C. Ha3-tagged Clb1 and Clb1-mDK were expressed from the *DMC1* promoter. (C) Deletion of *AMA1* reduces Clb1's interaction with the APC/C.

D Proteins interacting with Ama1 at metaphase I. Ama1-GFP and Esp1-GFP (negative control) were purified from P_{HSL1}-*CDC20* strains at 8 h in SPM, digested with trypsin and Lys-C, and subjected to LC–MS/MS. -$\log_{10}$-transformed *P*-values and mean $\log_2$-transformed label-free quantifications of copurifying proteins were obtained from MaxQuant and displayed as a volcano plot. Baits (green), selected proteins with $P < 0.05$ (red) or $P > 0.05$ (orange), and the eight subunits of the CCT chaperonin (brown) are labeled. CCT is known to encapsulate and fold APC/C activators (Camasses *et al*, 2003). Only a subset of APC/C subunits is marked. Note that C-terminally tagged Ama1 cannot bind the APC/C.

E Binding of Clb1, Clb3, and Cdk1 to Ama1-myc9 immunoprecipitated with α-Myc antibodies.

Data information: (D) is based on α-GFP purifications from three independent cultures per strain.
Source data are available online for this figure.

 © 2023 The Authors

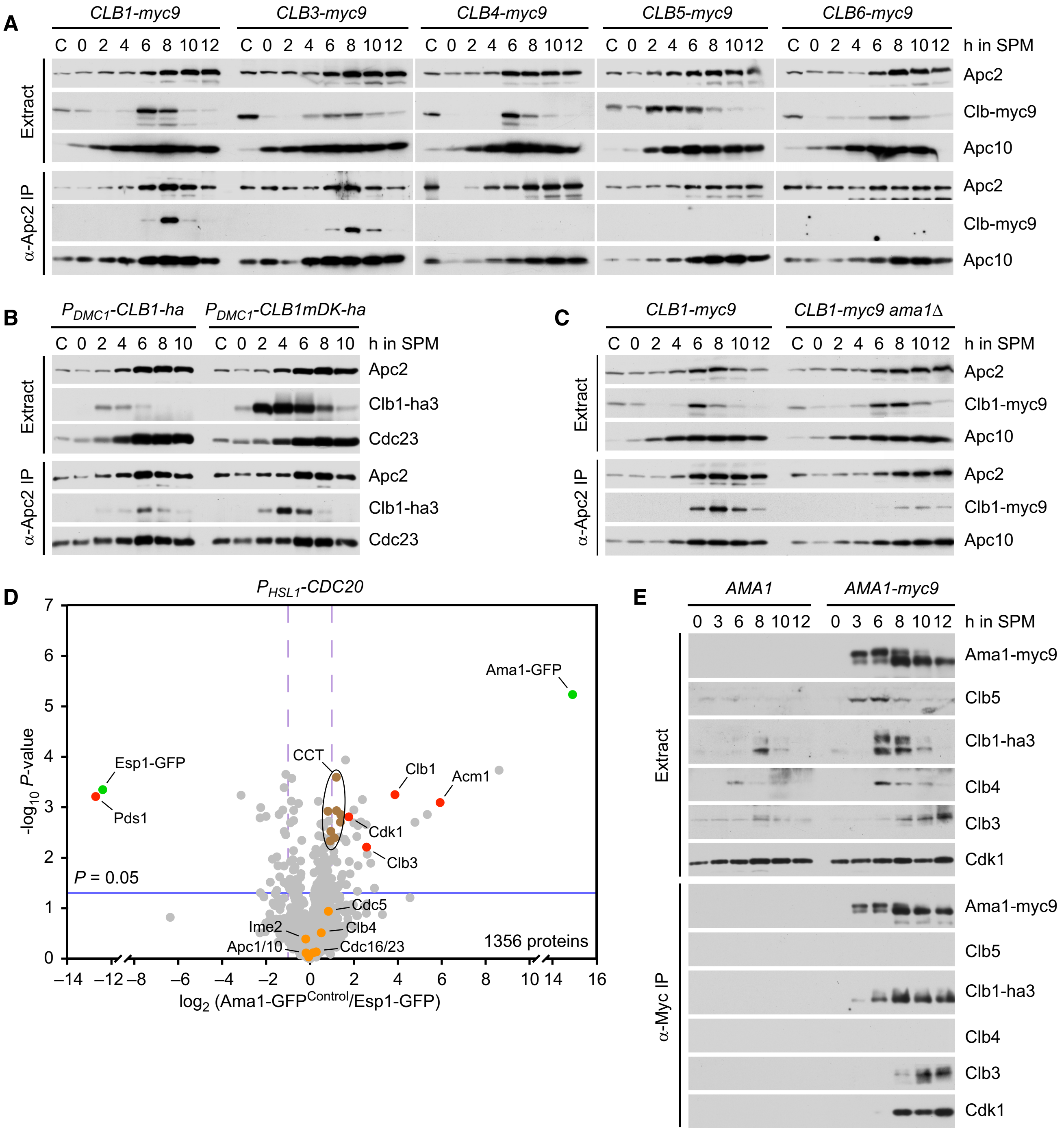
A
CLB1-myc9
CLB3-myc9
CLB4-myc9
CLB5-myc9
CLB6-myc9
C 0 2 4 6 8 10 12
h in SPM
Extract
α-Apc2 IP
Apc2
Clb-myc9
Apc10
B
PDMC1-CLB1-ha
PDMC1-CLB1mDK-ha
C 0 2 4 6 8 10
Clb1-ha3
Cdc23
C
CLB1-myc9
CLB1-myc9 ama1Δ
Clb1-myc9
D
PHSL1-CDC20
Ama1-GFP
Esp1-GFP
Pds1
CCT
Clb1
Acm1
Cdk1
Clb3
P = 0.05
Cdc5
Ime2
Clb4
Apc1/10
Cdc16/23
1356 proteins
-log10 P-value
log2 (Ama1-GFPControl/Esp1-GFP)
E
AMA1
AMA1-myc9
0 3 6 8 10 12
Ama1-myc9
Clb5
Clb1-ha3
Clb4
Clb3
Cdk1
α-Myc IP

Figure EV4.

Figure EV5. Analysis of Cdc5/Spo13-dependent phosphorylation of Clb1.

A Clb1-ha3 binds to Cdc15-myc15 in α-Myc immunoprecipitations from extracts of control and *spo13Δ* cells.

B Meiosis in *clb1-12A* and *clb1-12D* cells. Top, time lapse series from the imaging of spindles (GFP-tubulin), nucleolar release of Cdc14-GFP, and TetR-Tomato, which labels the nucleoplasm (diffuse signal) and the centromeres of one copy of chromosome V (dots). First (blue) and second (green) Cdc14 release are marked. Bottom, meiotic events quantified in cells synchronized *in silico* to spindle formation at metaphase I ($t = 0$). The duration of metaphase I is similar in *clb1-12A* and *clb1-12D* cells ($P = 0.33$).

C P_{HSL1}-*CDC20 clb1-12A* cells degrade Rim4 earlier than P_{HSL1}-*CDC20 clb1-6A* cells ($P < 0.0001$). Top, time-lapse series from the imaging of Rim4-mNG and SPBs (Cnm67-Tomato). Bottom, the presence of Rim4-mNG quantified in cells synchronized *in silico* to SPB separation at metaphase I ($t = 0$).

D Clb1 phosphorylation is not required for the activation of Spo13. Top, time-lapse series from the imaging of Rim4-mNG and SPBs (Cnm67-Tomato) in P_{HSL1}-*CDC20 ama1Δ* cells carrying *clb1-12A* or *clb1Δ*. Bottom, the presence of Rim4-mNG quantified in cells synchronized *in silico* to SPB separation at metaphase I ($t = 0$).

Data information: Data are representative of two (B and C) or three (D) independent experiments. Means were compared using Welch's *t*-test. Scale bar, 4 μm.
Source data are available online for this figure.

 © 2023 The Authors

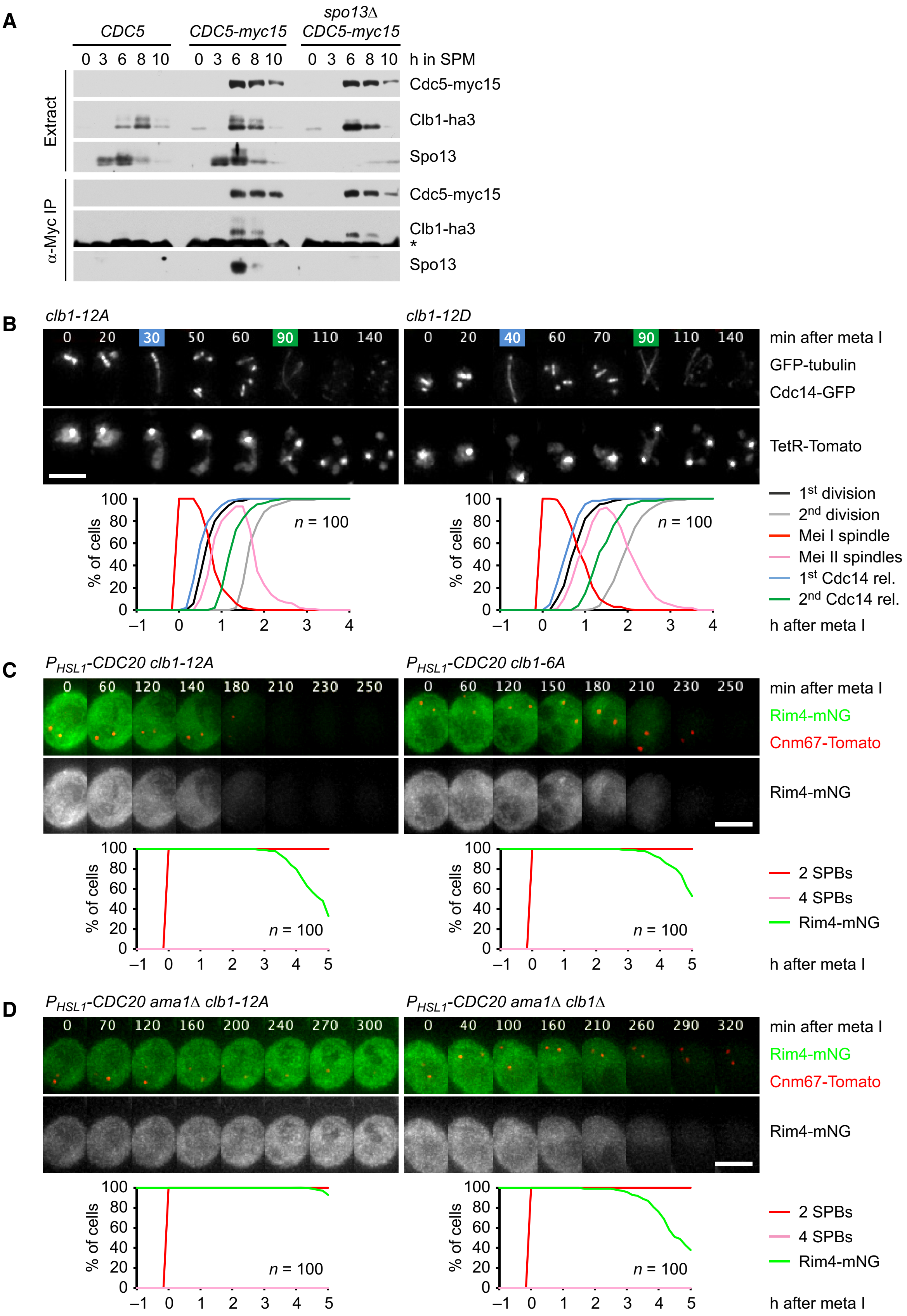
A
CDC5
CDC5-myc15
spo13Δ CDC5-myc15
0 3 6 8 10
h in SPM
Extract
α-Myc IP
Cdc5-myc15
Clb1-ha3
Spo13
*
B
clb1-12A
clb1-12D
min after meta I
GFP-tubulin
Cdc14-GFP
TetR-Tomato
% of cells
n = 100
1st division
2nd division
Mei I spindle
Mei II spindles
1st Cdc14 rel.
2nd Cdc14 rel.
h after meta I
C
PHSL1-CDC20 clb1-12A
PHSL1-CDC20 clb1-6A
Rim4-mNG
Cnm67-Tomato
2 SPBs
4 SPBs
D
PHSL1-CDC20 ama1Δ clb1-12A
PHSL1-CDC20 ama1Δ clb1Δ

Figure EV5.
