# Supplementary figures and images for: Spo13/MEIKIN ensures a Two‐Division meiosis by preventing the activation of APC/CAma1 at meiosis I (part 1 of 2)
Source: EMBO J. 2023 Sep 20;42(20):e114288. doi: 10.15252/embj.2023114288 (PMC10577557; doi:10.15252/embj.2023114288)

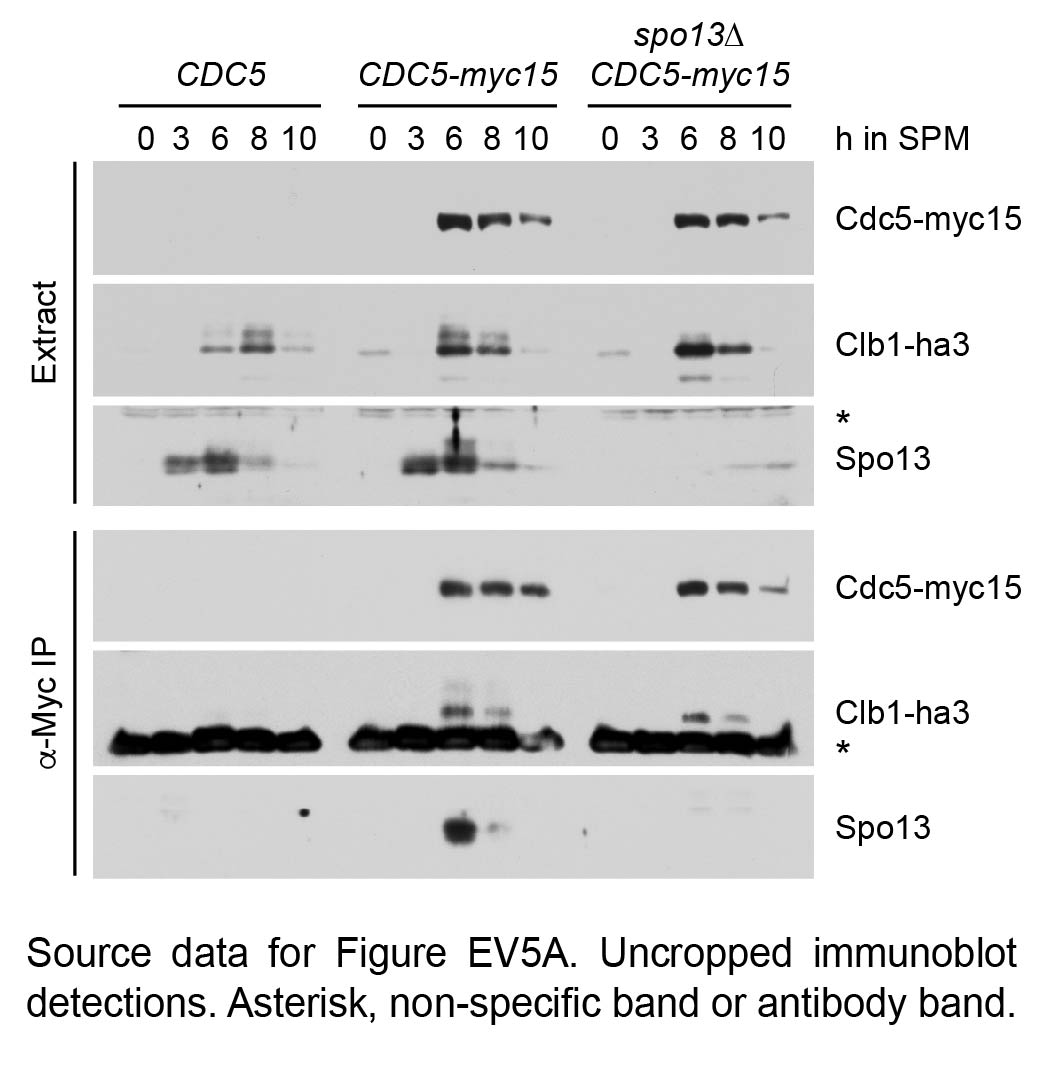

Supplement: Supplementary file 3 — Source Data for Expanded View and Appendix [file EMBJ-42-e114288-s014.zip › spo13_SourceDataForExpandedView_3/SourceDataForFigureEV5/SourceDataForFigureEV5A_Blots.jpg]

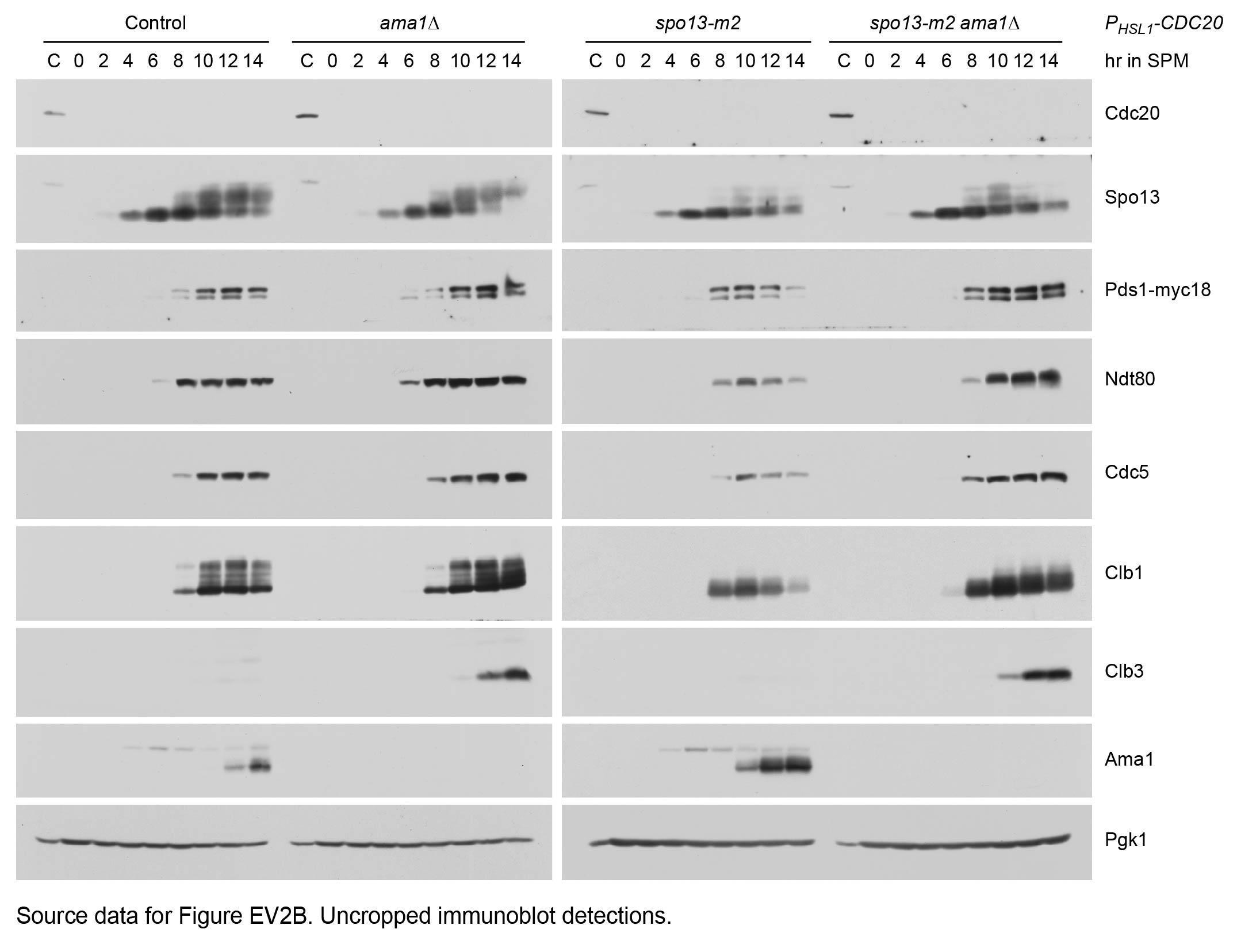

Supplement: Supplementary file 3 — Source Data for Expanded View and Appendix [file EMBJ-42-e114288-s014.zip › spo13_SourceDataForExpandedView_3/SourceDataForFigureEV2/SourceDataForFigureEV2B_Blots.jpg]

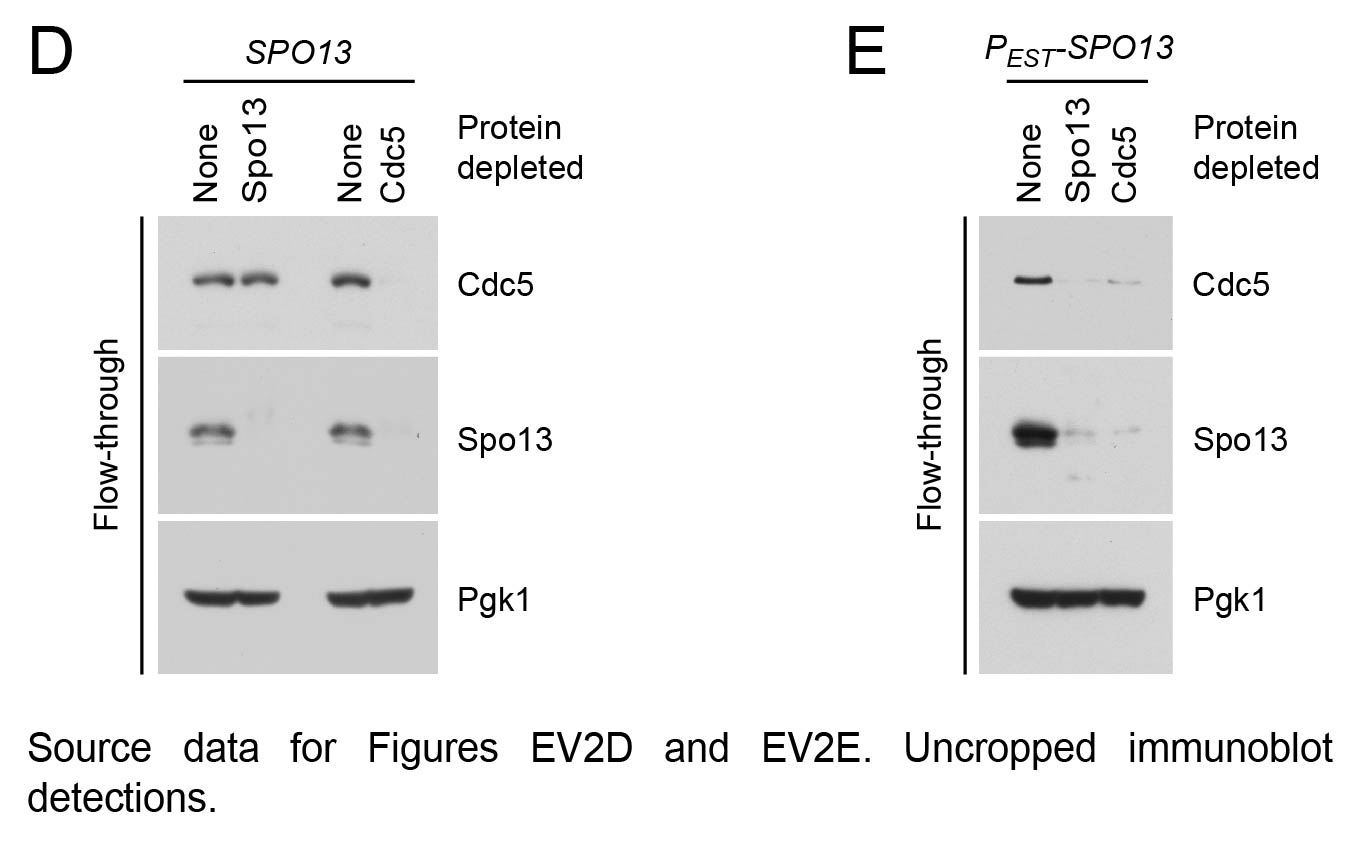

Supplement: Supplementary file 3 — Source Data for Expanded View and Appendix [file EMBJ-42-e114288-s014.zip › spo13_SourceDataForExpandedView_3/SourceDataForFigureEV2/SourceDataforFigureEV2DE_Blots.jpg]

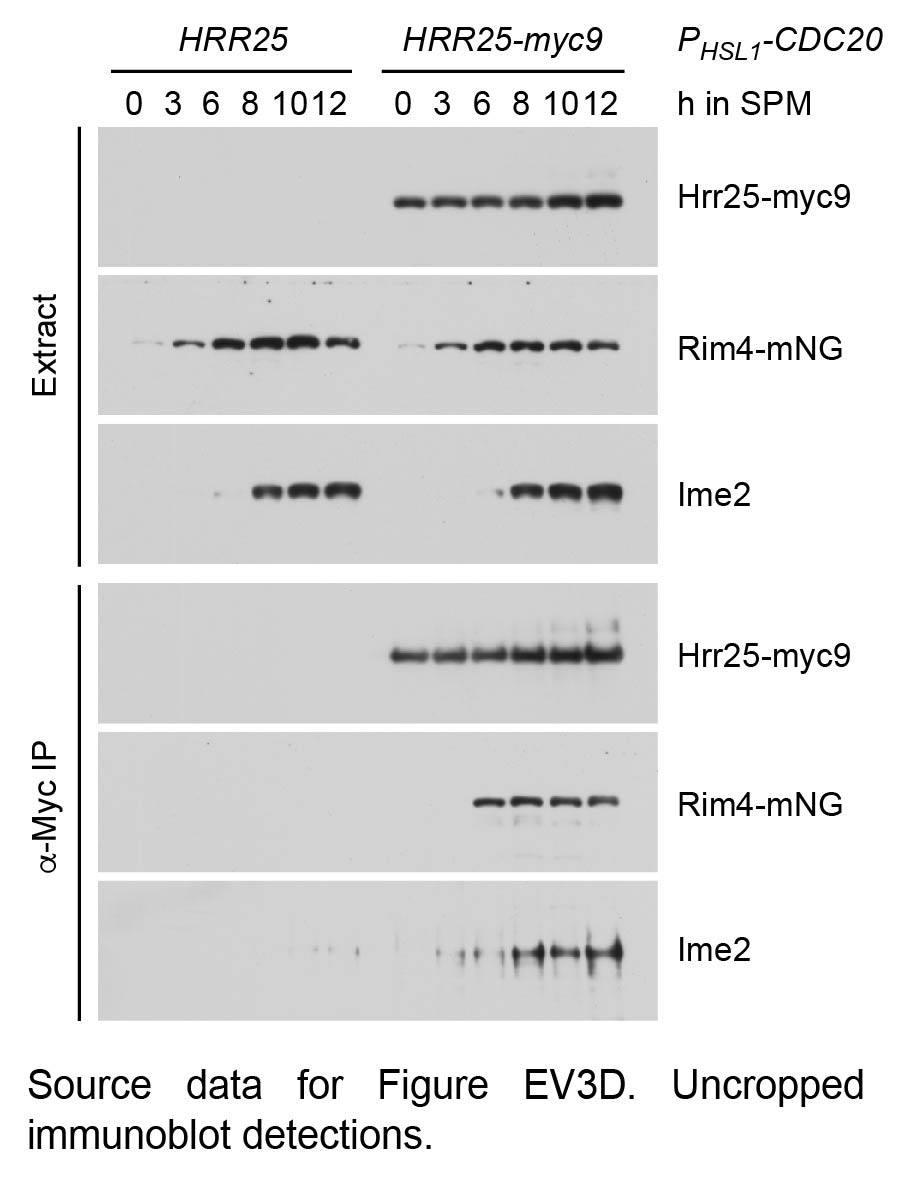

Supplement: Supplementary file 3 — Source Data for Expanded View and Appendix [file EMBJ-42-e114288-s014.zip › spo13_SourceDataForExpandedView_3/SourceDataForFigureEV3/SourceDataForFigureEV3D_Blots.jpg]

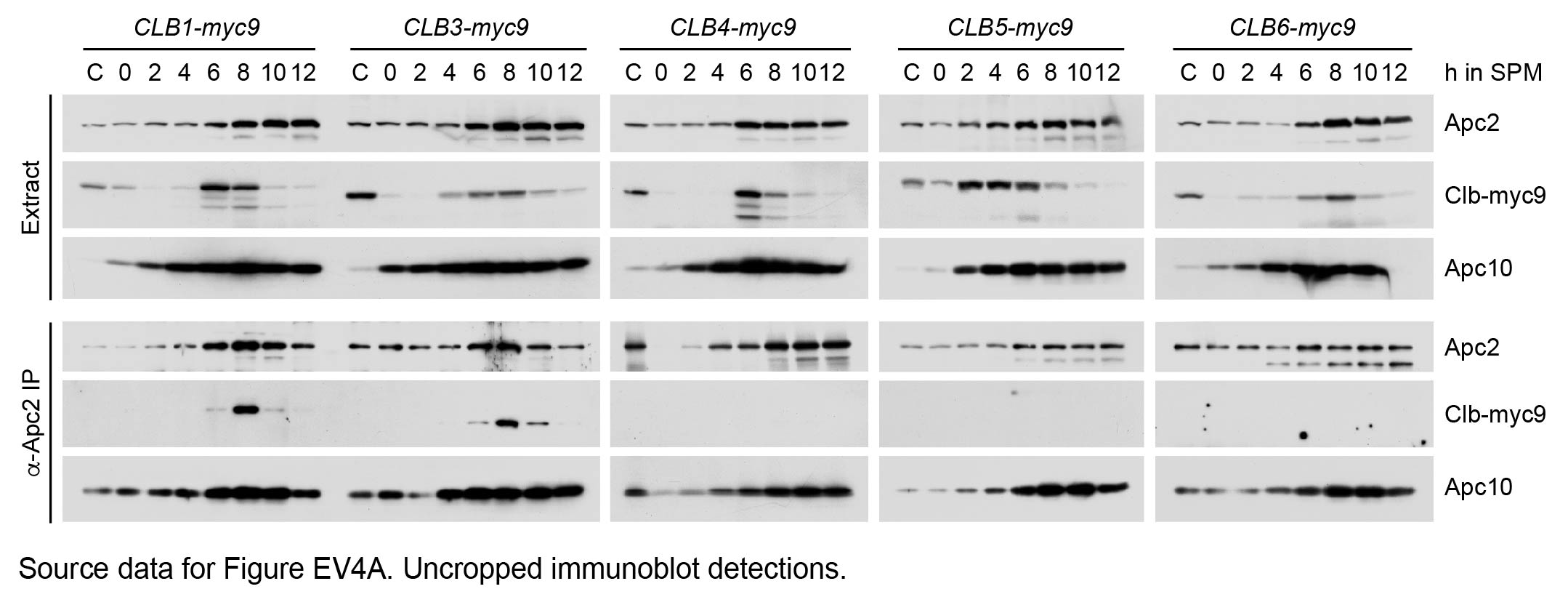

Supplement: Supplementary file 3 — Source Data for Expanded View and Appendix [file EMBJ-42-e114288-s014.zip › spo13_SourceDataForExpandedView_3/SourceDataForFigureEV4/SourceDataForFigureEV4A_Blots.jpg]

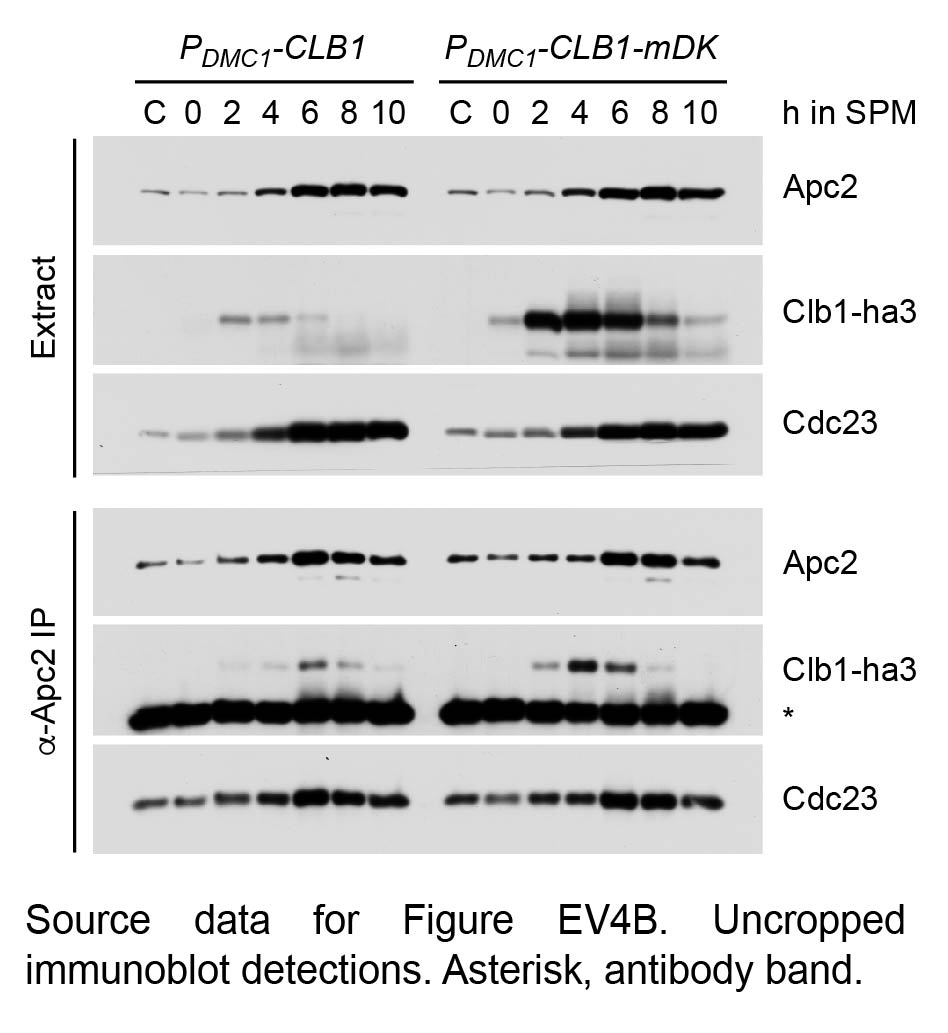

Supplement: Supplementary file 3 — Source Data for Expanded View and Appendix [file EMBJ-42-e114288-s014.zip › spo13_SourceDataForExpandedView_3/SourceDataForFigureEV4/SourceDataForFigureEV4B_Blots.jpg]

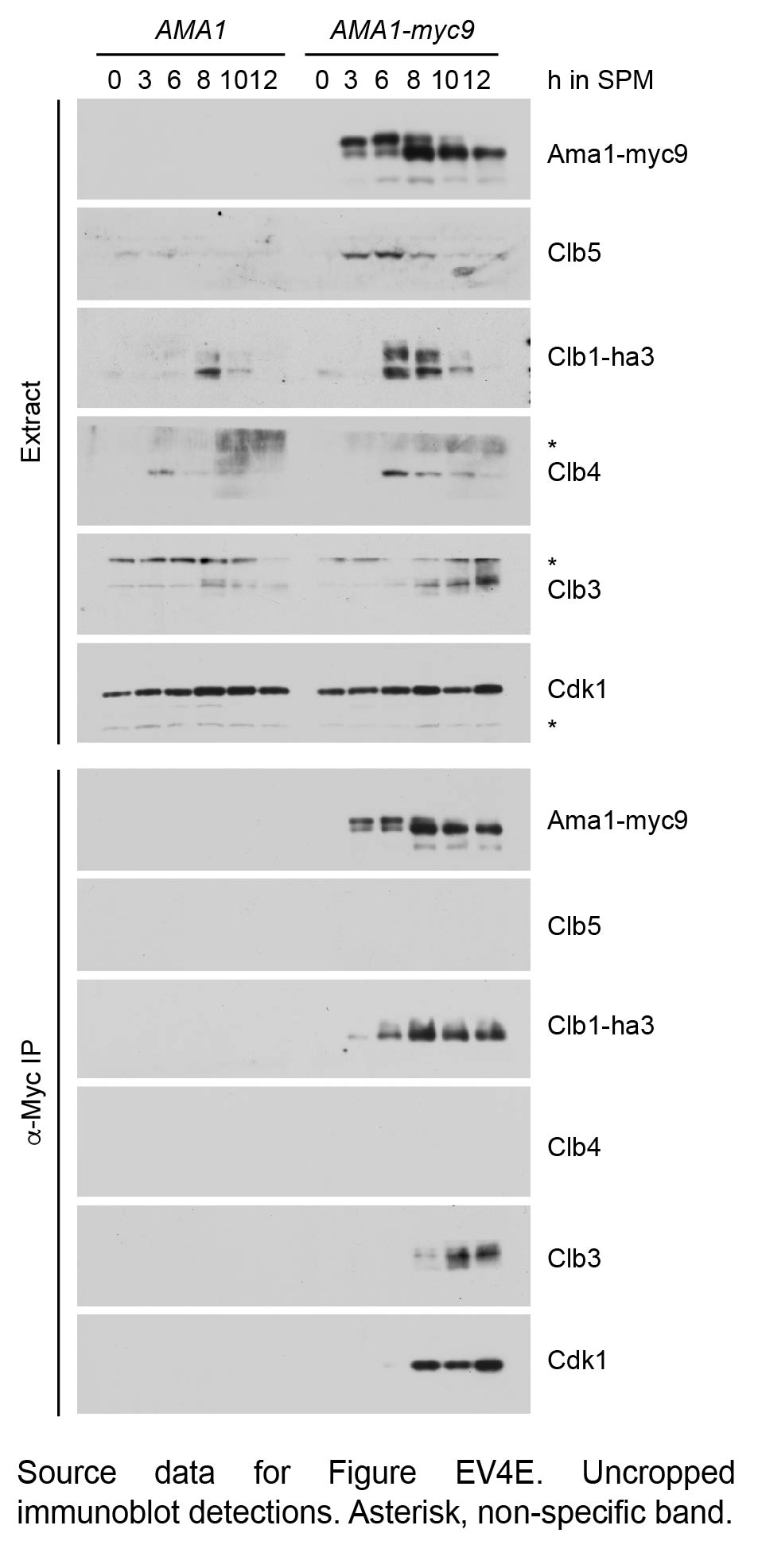

Supplement: Supplementary file 3 — Source Data for Expanded View and Appendix [file EMBJ-42-e114288-s014.zip › spo13_SourceDataForExpandedView_3/SourceDataForFigureEV4/SourceDataForFigureEV4E_Blots.jpg]

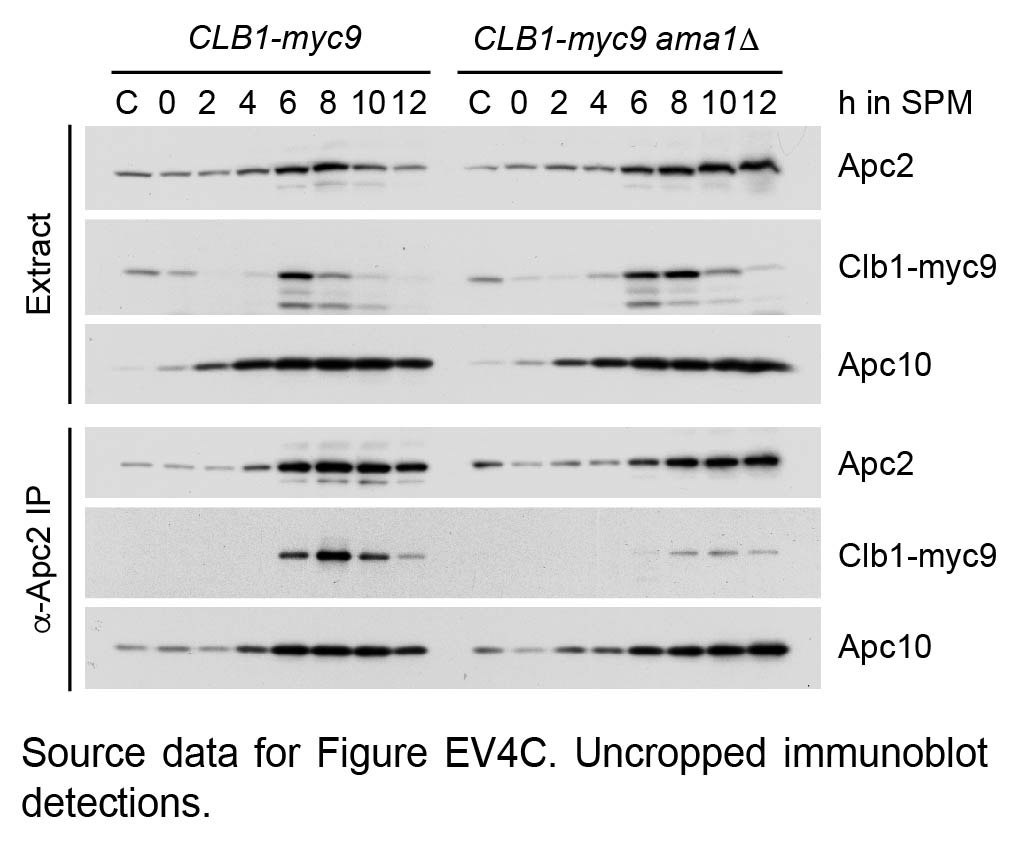

Supplement: Supplementary file 3 — Source Data for Expanded View and Appendix [file EMBJ-42-e114288-s014.zip › spo13_SourceDataForExpandedView_3/SourceDataForFigureEV4/SourceDataForFigureEV4C_Blots.jpg]

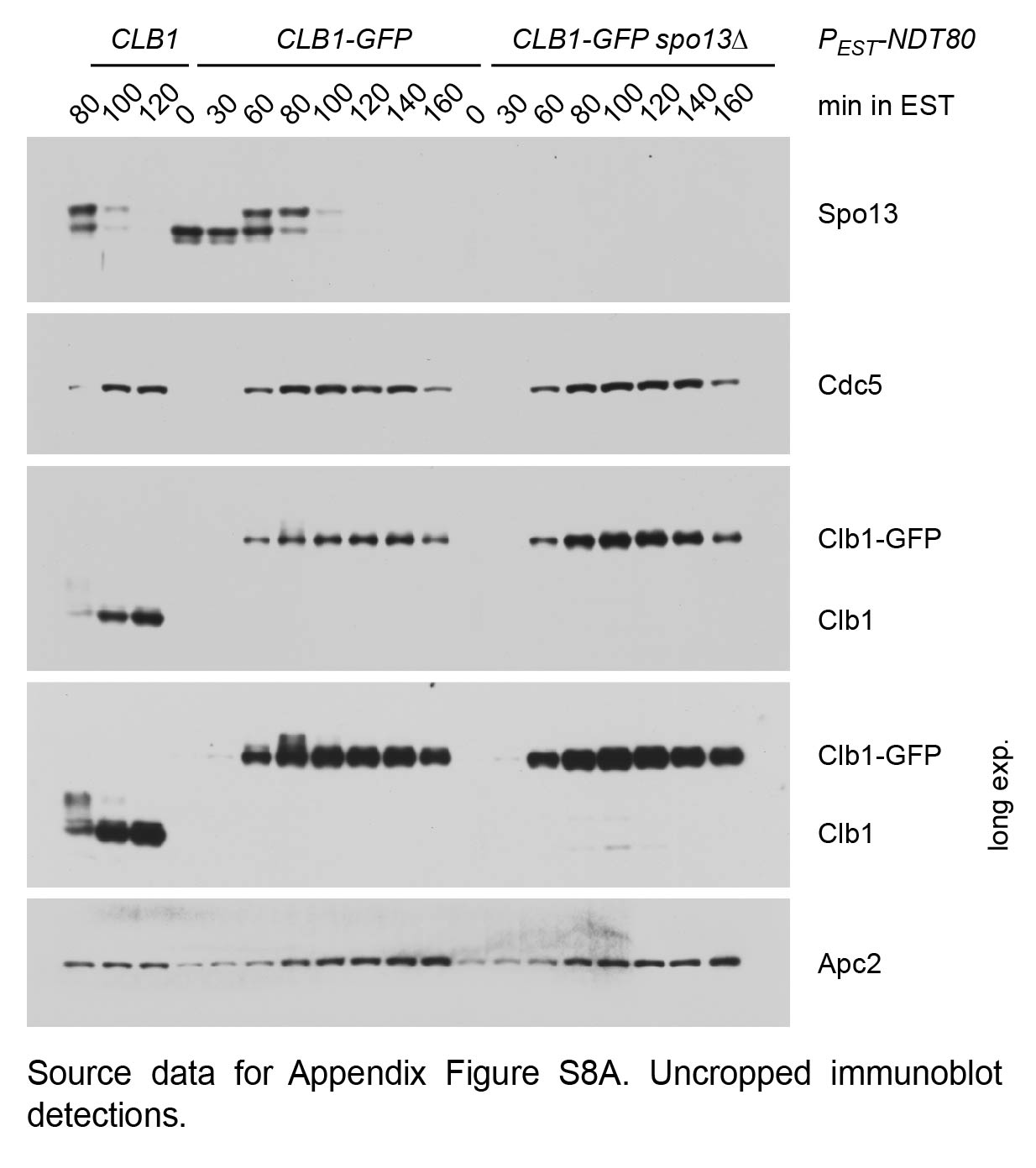

Supplement: Supplementary file 3 — Source Data for Expanded View and Appendix [file EMBJ-42-e114288-s014.zip › spo13_SourceDataForExpandedView_3/SourceDataForFigureS8/SourceDataForFigureS8A_Blots.jpg]

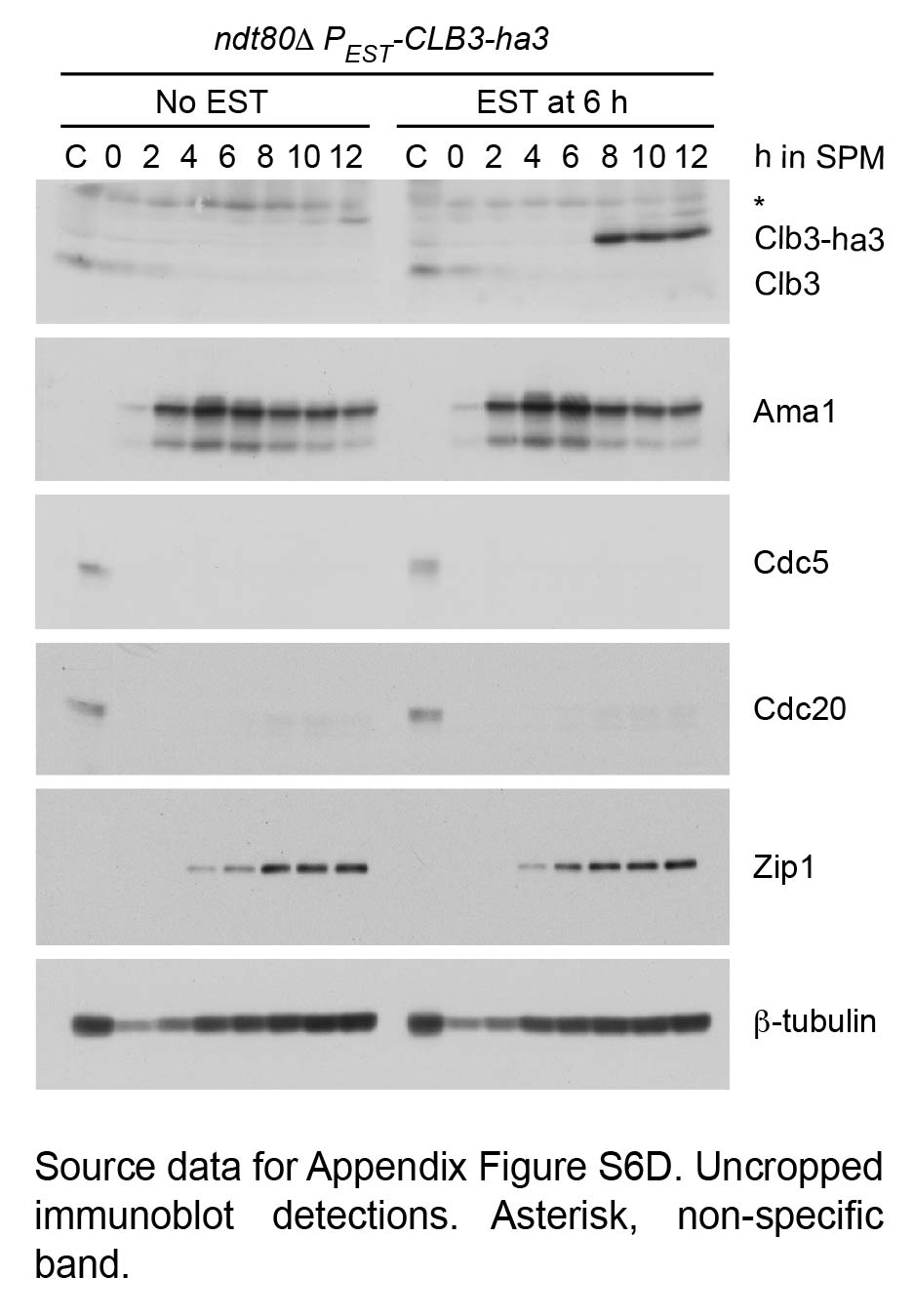

Supplement: Supplementary file 3 — Source Data for Expanded View and Appendix [file EMBJ-42-e114288-s014.zip › spo13_SourceDataForExpandedView_3/SourceDataForFigureS6/SourceDataForFigureS6D_Blots.jpg]

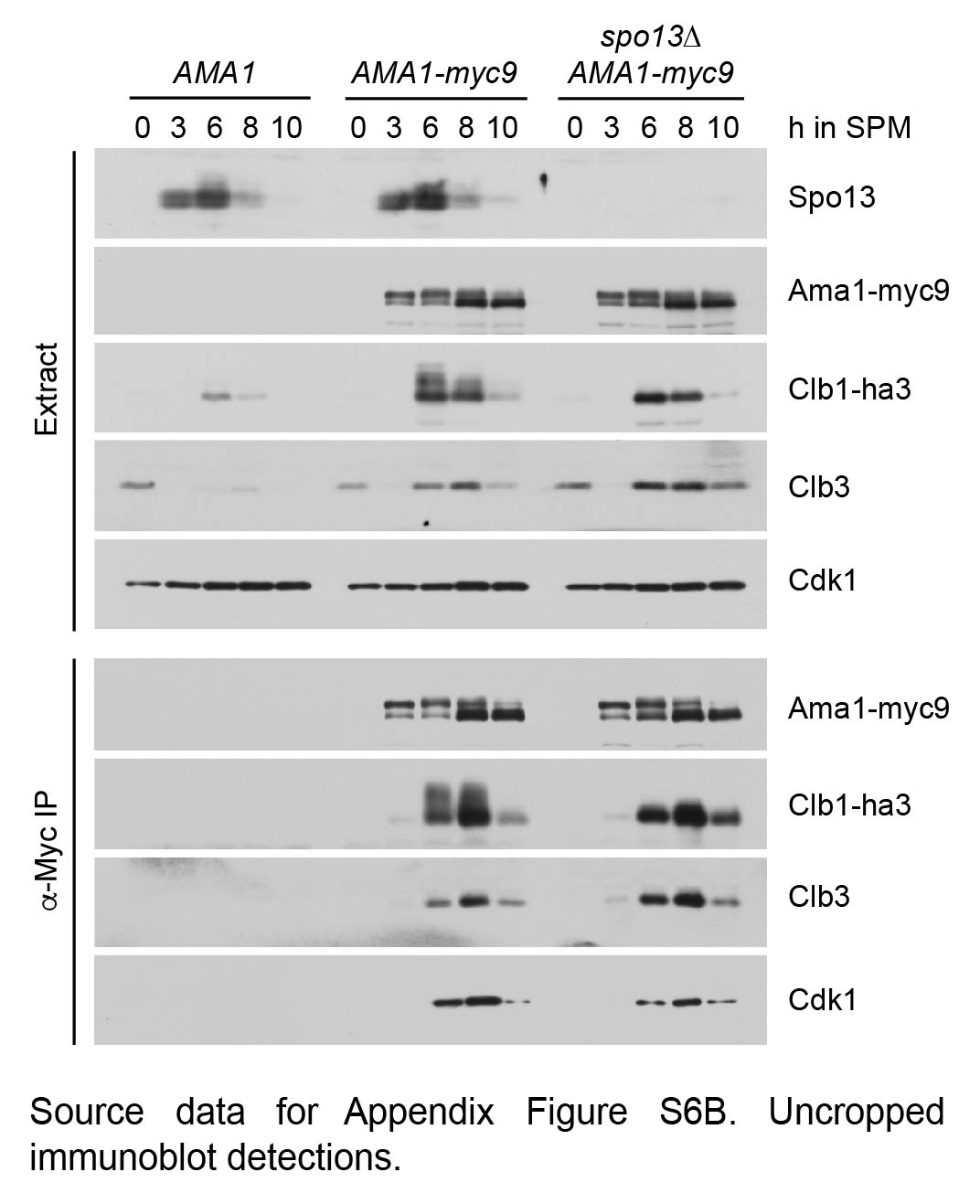

Supplement: Supplementary file 3 — Source Data for Expanded View and Appendix [file EMBJ-42-e114288-s014.zip › spo13_SourceDataForExpandedView_3/SourceDataForFigureS6/SourceDataForFigureS6B_Blots.jpg]

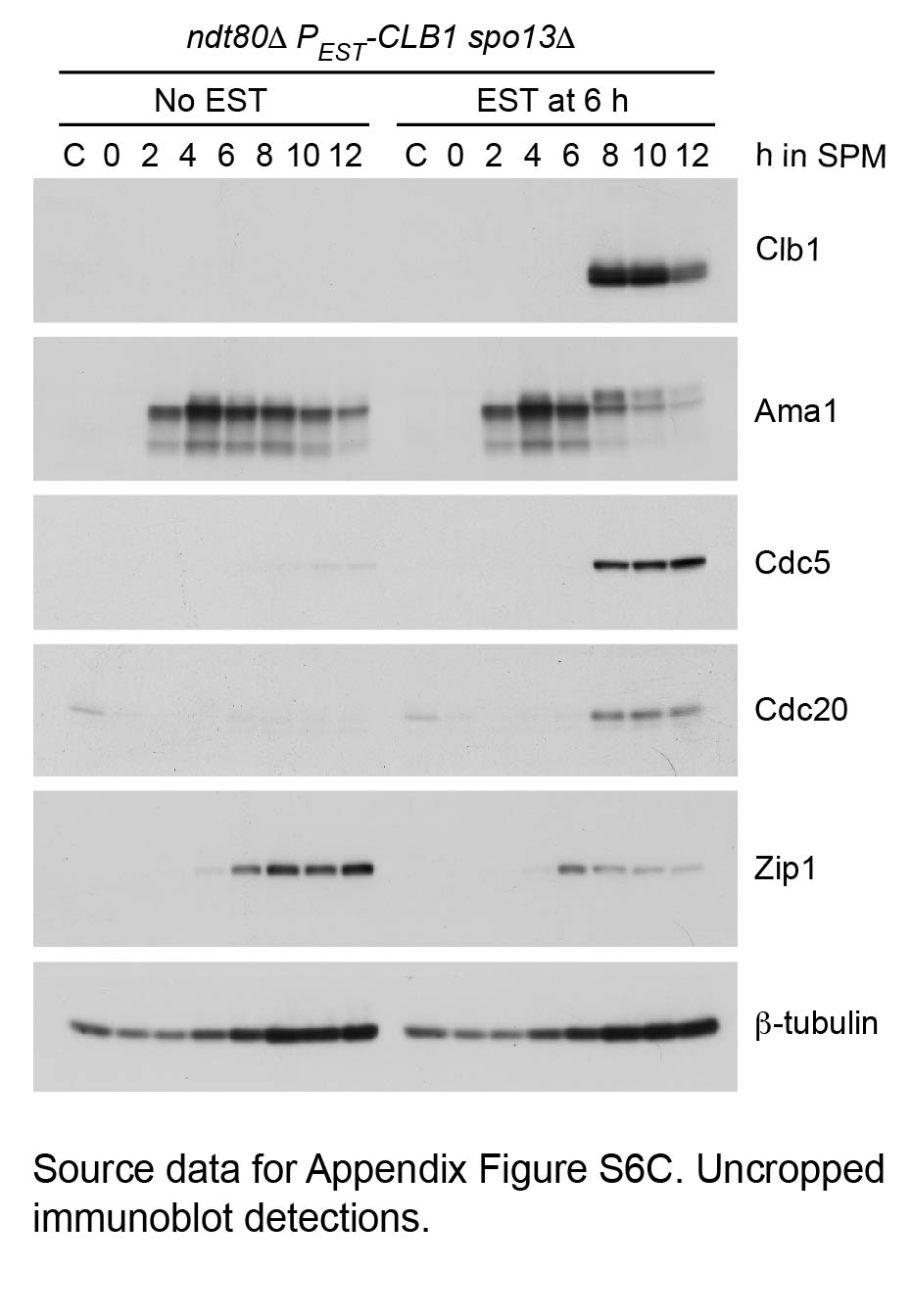

Supplement: Supplementary file 3 — Source Data for Expanded View and Appendix [file EMBJ-42-e114288-s014.zip › spo13_SourceDataForExpandedView_3/SourceDataForFigureS6/SourceDataForFigureS6C_Blots.jpg]

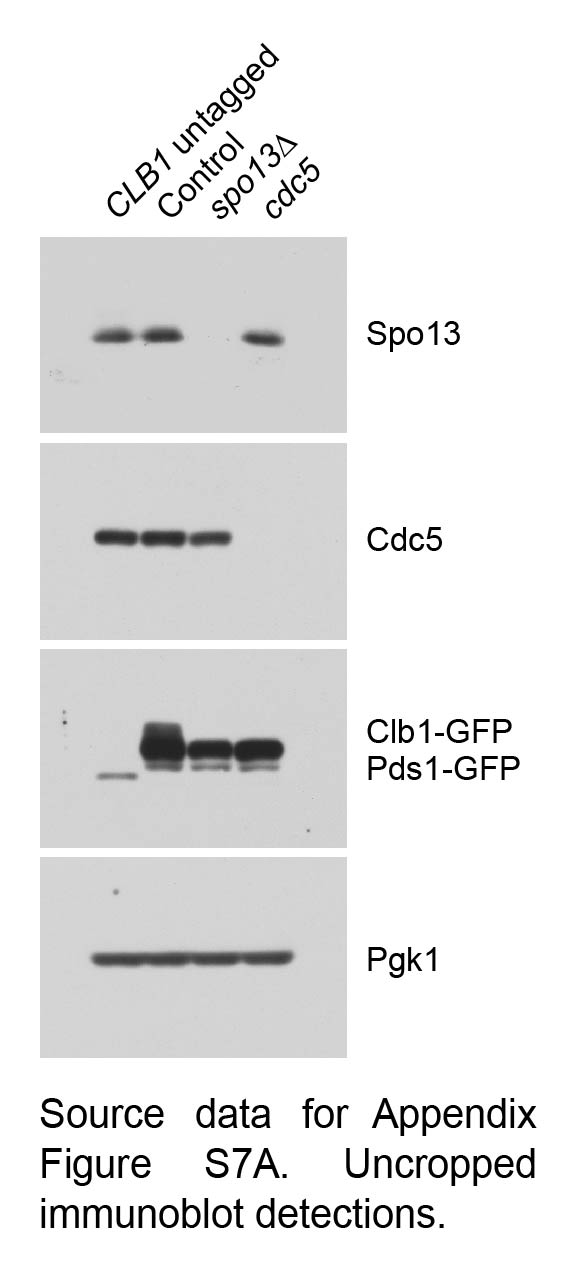

Supplement: Supplementary file 3 — Source Data for Expanded View and Appendix [file EMBJ-42-e114288-s014.zip › spo13_SourceDataForExpandedView_3/SourceDataForFigureS7/SourceDataForFigureS7A_Blots.jpg]

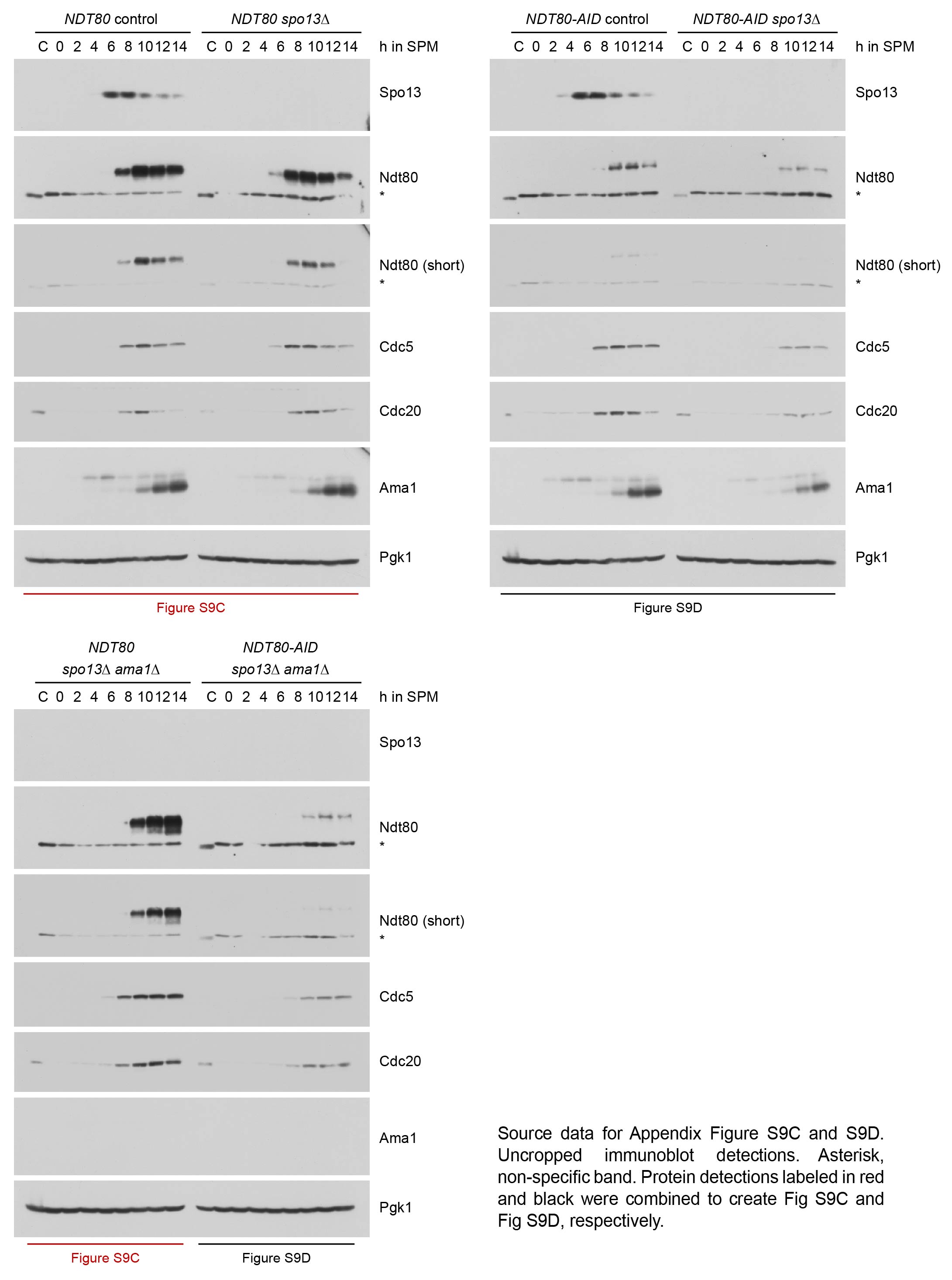

Supplement: Supplementary file 3 — Source Data for Expanded View and Appendix [file EMBJ-42-e114288-s014.zip › spo13_SourceDataForExpandedView_3/SourceDataForFigureS9/SourceDataForFigureS9CD_Blots.jpg]

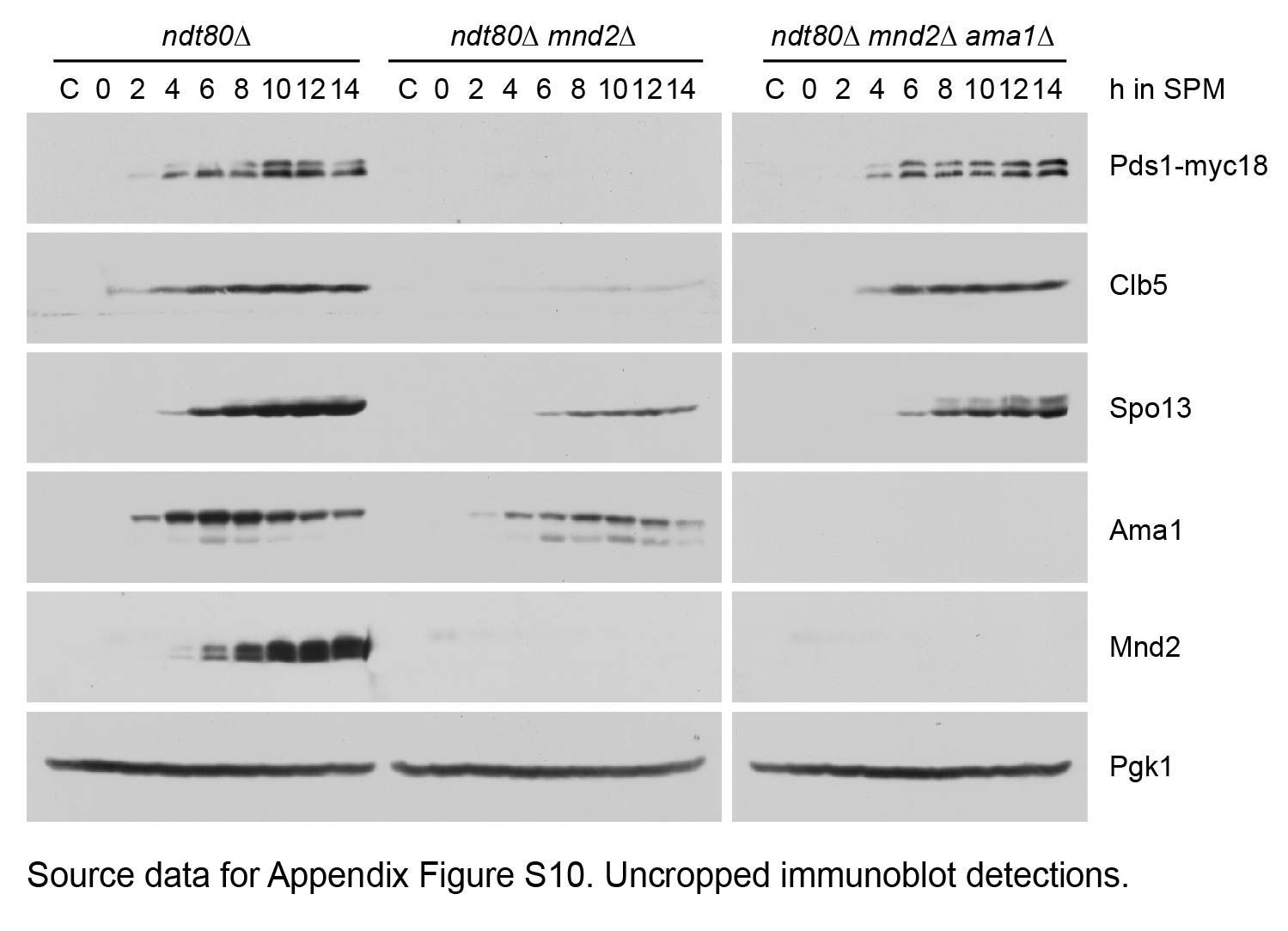

Supplement: Supplementary file 3 — Source Data for Expanded View and Appendix [file EMBJ-42-e114288-s014.zip › spo13_SourceDataForExpandedView_3/SourceDataForFigureS10/SourceDataForFigureS10_Blots.jpg]

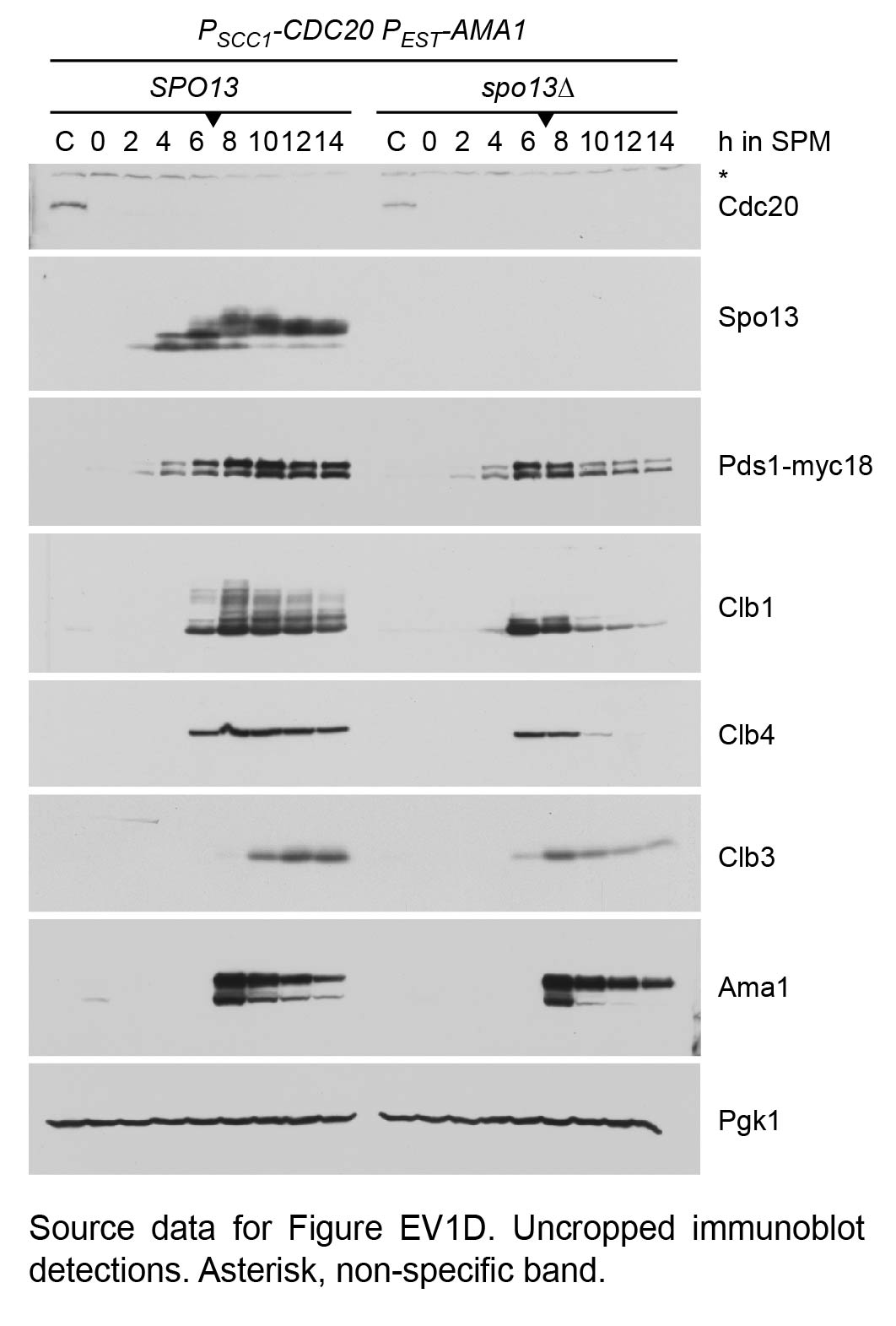

Supplement: Supplementary file 3 — Source Data for Expanded View and Appendix [file EMBJ-42-e114288-s014.zip › spo13_SourceDataForExpandedView_3/SourceDataForFigureEV1/SourceDataForFigureEV1D_Blots.jpg]

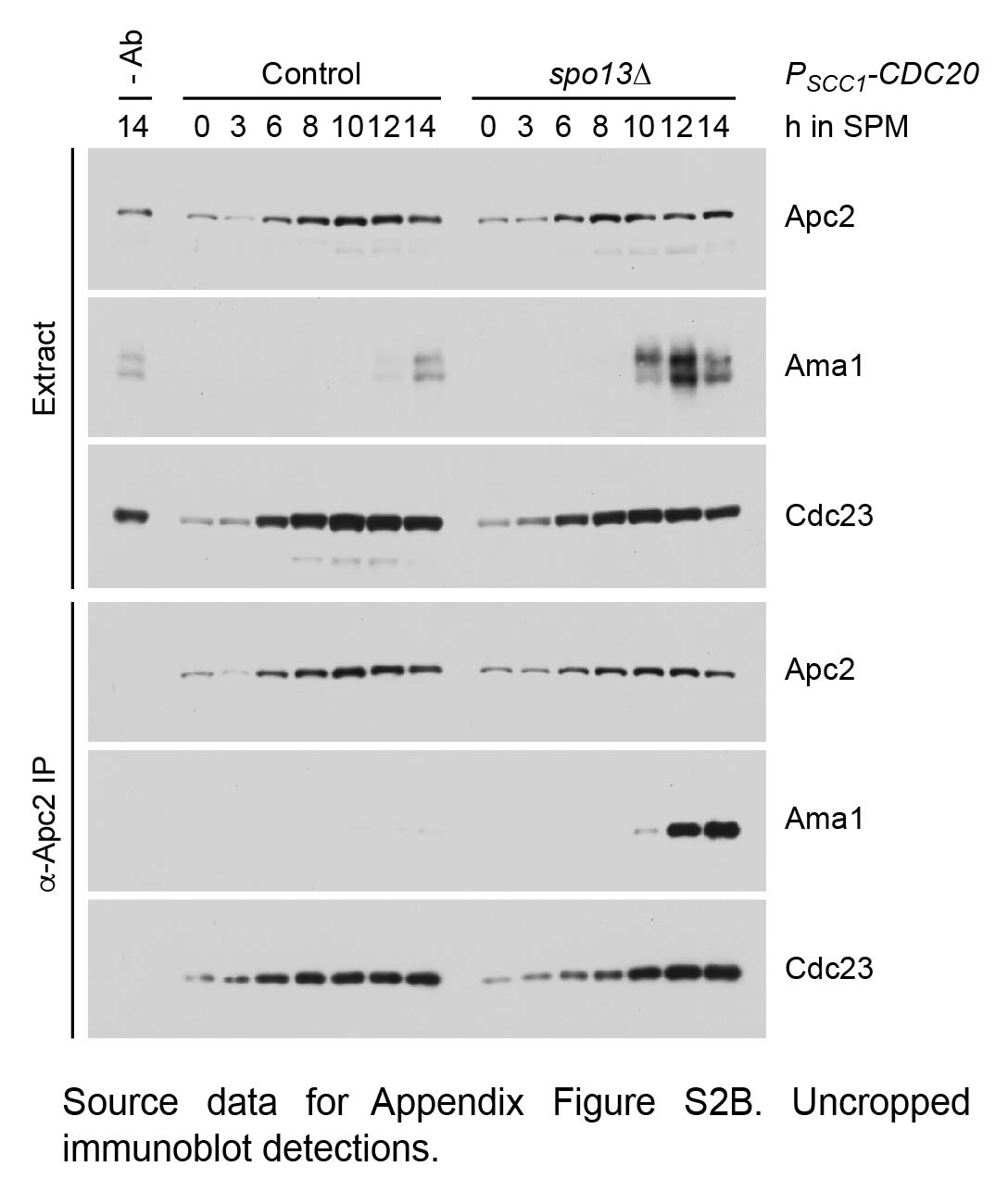

Supplement: Supplementary file 3 — Source Data for Expanded View and Appendix [file EMBJ-42-e114288-s014.zip › spo13_SourceDataForExpandedView_3/SourceDataForFigureS2/SourceDataForFigureS2B_Blots.jpg]

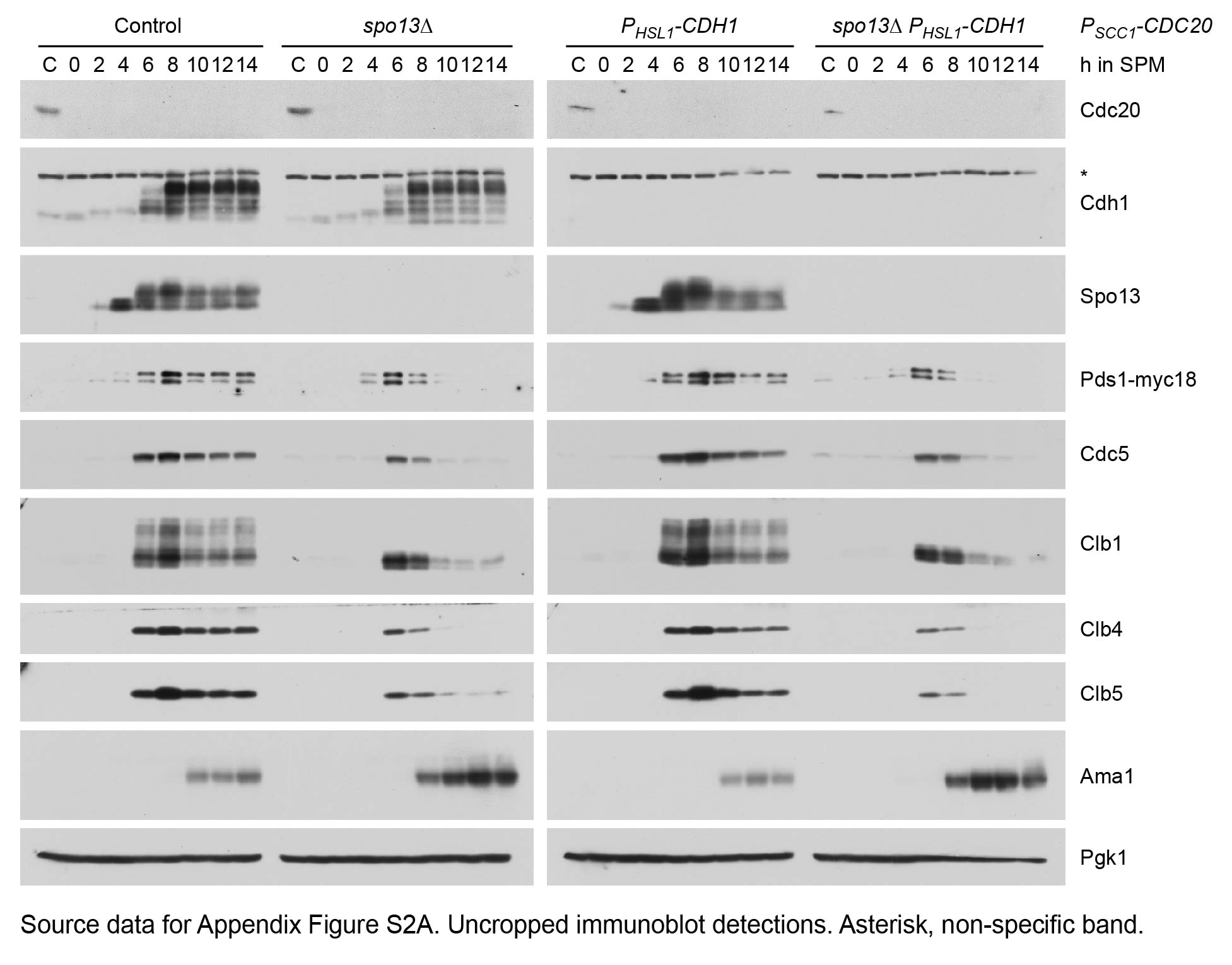

Supplement: Supplementary file 3 — Source Data for Expanded View and Appendix [file EMBJ-42-e114288-s014.zip › spo13_SourceDataForExpandedView_3/SourceDataForFigureS2/SourceDataForFigureS2A_Blots.jpg]

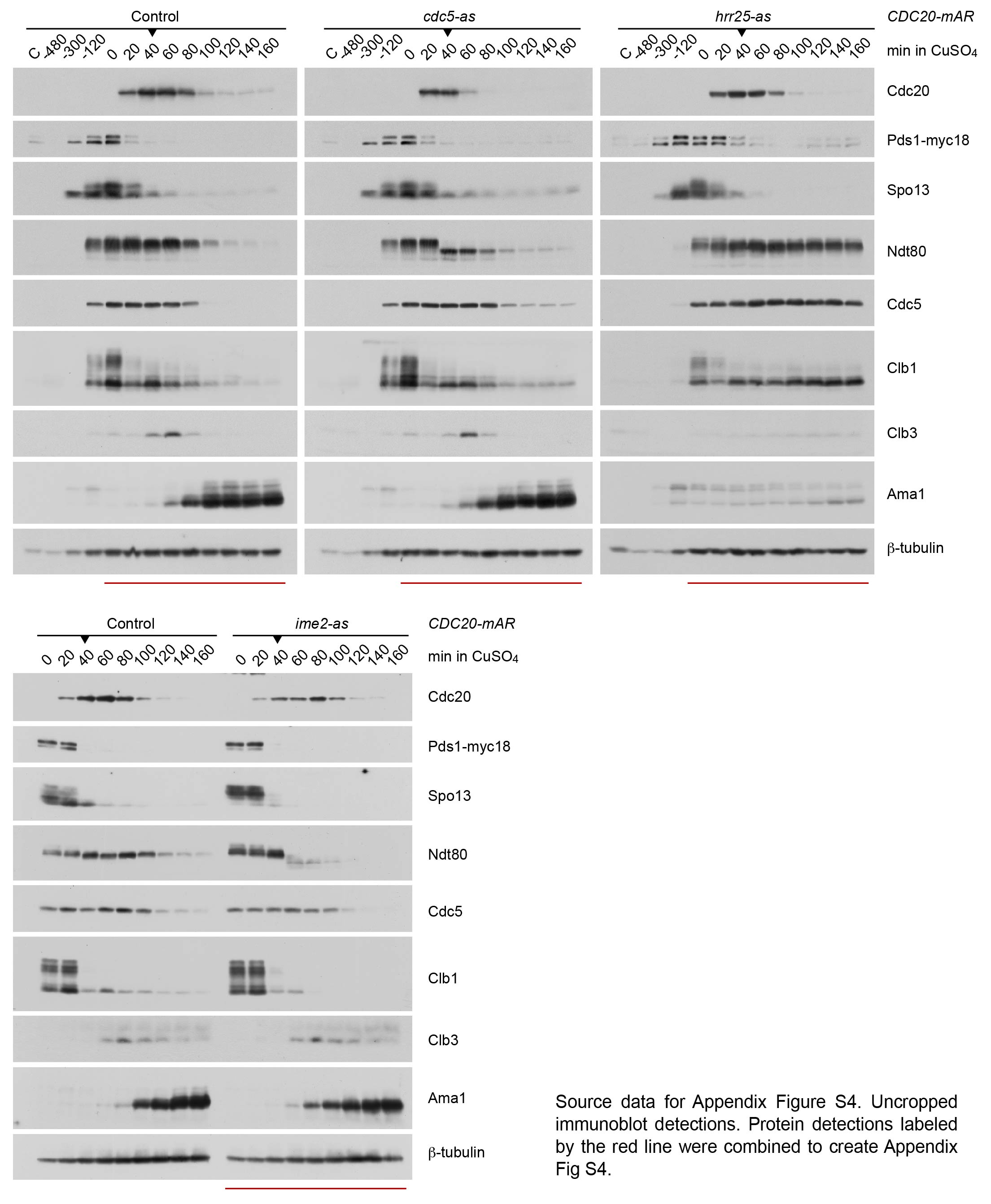

Supplement: Supplementary file 3 — Source Data for Expanded View and Appendix [file EMBJ-42-e114288-s014.zip › spo13_SourceDataForExpandedView_3/SourceDataForFigureS4/SourceDataForFigureS4_Blots.jpg]

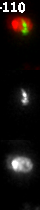

Supplement: Supplementary file 3 — Source Data for Expanded View and Appendix [file EMBJ-42-e114288-s014.zip › spo13_SourceDataForExpandedView_3/SourceDataForFigureEV5/SourceDataForFigureEV5B_Imaging/SourceDataForFigureEV5B_clb1-12A.tif]

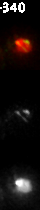

Supplement: Supplementary file 3 — Source Data for Expanded View and Appendix [file EMBJ-42-e114288-s014.zip › spo13_SourceDataForExpandedView_3/SourceDataForFigureEV5/SourceDataForFigureEV5B_Imaging/SourceDataForFigureEV5B_clb1-12D.tif]

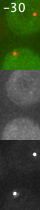

Supplement: Supplementary file 3 — Source Data for Expanded View and Appendix [file EMBJ-42-e114288-s014.zip › spo13_SourceDataForExpandedView_3/SourceDataForFigureEV5/SourceDataForFigureEV5C_Imaging/SourceDataForFigureEV5C_cdc20_clb1-12A.tif]

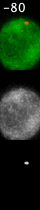

Supplement: Supplementary file 3 — Source Data for Expanded View and Appendix [file EMBJ-42-e114288-s014.zip › spo13_SourceDataForExpandedView_3/SourceDataForFigureEV5/SourceDataForFigureEV5C_Imaging/SourceDataForFigureEV5C_cdc20_clb1-6A.tif]

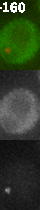

Supplement: Supplementary file 3 — Source Data for Expanded View and Appendix [file EMBJ-42-e114288-s014.zip › spo13_SourceDataForExpandedView_3/SourceDataForFigureEV5/SourceDataForFigureEV5D_Imaging/SourceDataForFigureEV5D_cdc20_ama1_clb1-12A.tif]

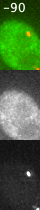

Supplement: Supplementary file 3 — Source Data for Expanded View and Appendix [file EMBJ-42-e114288-s014.zip › spo13_SourceDataForExpandedView_3/SourceDataForFigureEV5/SourceDataForFigureEV5D_Imaging/SourceDataForFigureEV5D_cdc20_ama1_clb1.tif]

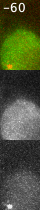

Supplement: Supplementary file 3 — Source Data for Expanded View and Appendix [file EMBJ-42-e114288-s014.zip › spo13_SourceDataForExpandedView_3/SourceDataForFigureEV2/SourceDataForFigureEV2C_Imaging/SourceDataForFigureEV2C_cdc20_spo13-m2.tif]

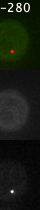

Supplement: Supplementary file 3 — Source Data for Expanded View and Appendix [file EMBJ-42-e114288-s014.zip › spo13_SourceDataForExpandedView_3/SourceDataForFigureEV2/SourceDataForFigureEV2C_Imaging/SourceDataForFigureEV2C_cdc20_cdc5-as.tif]

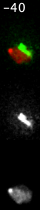

Supplement: Supplementary file 3 — Source Data for Expanded View and Appendix [file EMBJ-42-e114288-s014.zip › spo13_SourceDataForExpandedView_3/SourceDataForFigureEV2/SourceDataForFigureEV2A_Imaging/SourceDataForFigureEV2A_Control.tif]

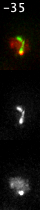

Supplement: Supplementary file 3 — Source Data for Expanded View and Appendix [file EMBJ-42-e114288-s014.zip › spo13_SourceDataForExpandedView_3/SourceDataForFigureEV2/SourceDataForFigureEV2A_Imaging/SourceDataForFigureEV2A_spo13-m2.tif]

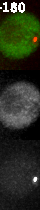

Supplement: Supplementary file 3 — Source Data for Expanded View and Appendix [file EMBJ-42-e114288-s014.zip › spo13_SourceDataForExpandedView_3/SourceDataForFigureEV3/SourceDataForFigureEV3C_Imaging/SourceDataForFigureEV3C_cdc20_ama1_hrr25dC.tif]

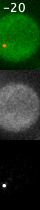

Supplement: Supplementary file 3 — Source Data for Expanded View and Appendix [file EMBJ-42-e114288-s014.zip › spo13_SourceDataForExpandedView_3/SourceDataForFigureEV3/SourceDataForFigureEV3C_Imaging/SourceDataForFigureEV3C_cdc20_ama1_ime2as.tif]

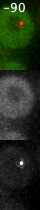

Supplement: Supplementary file 3 — Source Data for Expanded View and Appendix [file EMBJ-42-e114288-s014.zip › spo13_SourceDataForExpandedView_3/SourceDataForFigureEV3/SourceDataForFigureEV3C_Imaging/SourceDataForFigureEV3C_cdc20_ama1_hrr25dC_ime2as.tif]

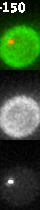

Supplement: Supplementary file 3 — Source Data for Expanded View and Appendix [file EMBJ-42-e114288-s014.zip › spo13_SourceDataForExpandedView_3/SourceDataForFigureEV3/SourceDataForFigureEV3B_Imaging/SourceDataForFigureEV3B_cdc20_ama1_ime2dC_hrr25as.tif]

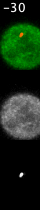

Supplement: Supplementary file 3 — Source Data for Expanded View and Appendix [file EMBJ-42-e114288-s014.zip › spo13_SourceDataForExpandedView_3/SourceDataForFigureEV3/SourceDataForFigureEV3B_Imaging/SourceDataForFigureEV3B_cdc20_ama1_ime2dC.tif]

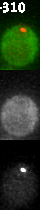

Supplement: Supplementary file 3 — Source Data for Expanded View and Appendix [file EMBJ-42-e114288-s014.zip › spo13_SourceDataForExpandedView_3/SourceDataForFigureEV3/SourceDataForFigureEV3B_Imaging/SourceDataForFigureEV3B_cdc20_ama1_hrr25as.tif]

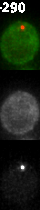

Supplement: Supplementary file 3 — Source Data for Expanded View and Appendix [file EMBJ-42-e114288-s014.zip › spo13_SourceDataForExpandedView_3/SourceDataForFigureEV3/SourceDataForFigureEV3A_Imaging/SourceDataForFigureEV3A_cdc20_ama1_spo13_hrr25as.tif]

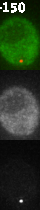

Supplement: Supplementary file 3 — Source Data for Expanded View and Appendix [file EMBJ-42-e114288-s014.zip › spo13_SourceDataForExpandedView_3/SourceDataForFigureEV3/SourceDataForFigureEV3A_Imaging/SourceDataForFigureEV3A_cdc20_ama1_spo13.tif]

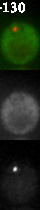

Supplement: Supplementary file 3 — Source Data for Expanded View and Appendix [file EMBJ-42-e114288-s014.zip › spo13_SourceDataForExpandedView_3/SourceDataForFigureEV3/SourceDataForFigureEV3A_Imaging/SourceDataForFigureEV3A_cdc20_ama1.tif]

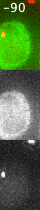

Supplement: Supplementary file 3 — Source Data for Expanded View and Appendix [file EMBJ-42-e114288-s014.zip › spo13_SourceDataForExpandedView_3/SourceDataForFigureS6/SourceDataForFigureS6E_Imaging/SourceDataForFigureS6E_cdc20_Ama1-6A.tif]

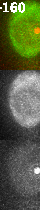

Supplement: Supplementary file 3 — Source Data for Expanded View and Appendix [file EMBJ-42-e114288-s014.zip › spo13_SourceDataForExpandedView_3/SourceDataForFigureS6/SourceDataForFigureS6E_Imaging/SourceDataForFigureS6E_cdc20_Ama1-6A_acm1.tif]

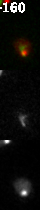

Supplement: Supplementary file 3 — Source Data for Expanded View and Appendix [file EMBJ-42-e114288-s014.zip › spo13_SourceDataForExpandedView_3/SourceDataForFigureS1/SourceDataForFigureS1C_Imaging/SourceDataForFigureS1C_mpc54_mpc70_spo13.tif]

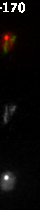

Supplement: Supplementary file 3 — Source Data for Expanded View and Appendix [file EMBJ-42-e114288-s014.zip › spo13_SourceDataForExpandedView_3/SourceDataForFigureS1/SourceDataForFigureS1C_Imaging/SourceDataForFigureS1C_mpc54_mpc70.tif]

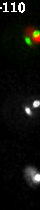

Supplement: Supplementary file 3 — Source Data for Expanded View and Appendix [file EMBJ-42-e114288-s014.zip › spo13_SourceDataForExpandedView_3/SourceDataForFigureS1/SourceDataForFigureS1B_Imaging/SourceDataForFigureS1B_mad2_spo13_rec8-18D.tif]

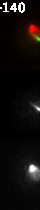

Supplement: Supplementary file 3 — Source Data for Expanded View and Appendix [file EMBJ-42-e114288-s014.zip › spo13_SourceDataForExpandedView_3/SourceDataForFigureS1/SourceDataForFigureS1B_Imaging/SourceDataForFigureS1B_mad2_spo13_REC8.tif]

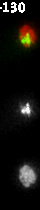

Supplement: Supplementary file 3 — Source Data for Expanded View and Appendix [file EMBJ-42-e114288-s014.zip › spo13_SourceDataForExpandedView_3/SourceDataForFigureS1/SourceDataForFigureS1A_Imaging/SourceDataForFigureS1A_cdc20_spo13.tif]

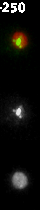

Supplement: Supplementary file 3 — Source Data for Expanded View and Appendix [file EMBJ-42-e114288-s014.zip › spo13_SourceDataForExpandedView_3/SourceDataForFigureS1/SourceDataForFigureS1A_Imaging/SourceDataForFigureS1A_cdc20.tif]

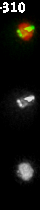

Supplement: Supplementary file 3 — Source Data for Expanded View and Appendix [file EMBJ-42-e114288-s014.zip › spo13_SourceDataForExpandedView_3/SourceDataForFigureS9/SourceDataForFigureS9B_Imaging/SourceDataForFigureS9B_NDT80aid.tif]

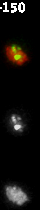

Supplement: Supplementary file 3 — Source Data for Expanded View and Appendix [file EMBJ-42-e114288-s014.zip › spo13_SourceDataForExpandedView_3/SourceDataForFigureS9/SourceDataForFigureS9B_Imaging/SourceDataForFigureS9B_NDT80aid_spo13_ama1.tif]

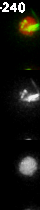

Supplement: Supplementary file 3 — Source Data for Expanded View and Appendix [file EMBJ-42-e114288-s014.zip › spo13_SourceDataForExpandedView_3/SourceDataForFigureS9/SourceDataForFigureS9B_Imaging/SourceDataForFigureS9B_NDT80aid_spo13.tif]

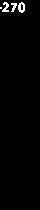

Supplement: Supplementary file 3 — Source Data for Expanded View and Appendix [file EMBJ-42-e114288-s014.zip › spo13_SourceDataForExpandedView_3/SourceDataForFigureS9/SourceDataForFigureS9A_Imaging/SourceDataForFigureS9A_control.tif]

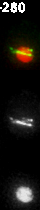

Supplement: Supplementary file 3 — Source Data for Expanded View and Appendix [file EMBJ-42-e114288-s014.zip › spo13_SourceDataForExpandedView_3/SourceDataForFigureS9/SourceDataForFigureS9A_Imaging/SourceDataForFigureS9A_spo13.tif]

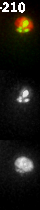

Supplement: Supplementary file 3 — Source Data for Expanded View and Appendix [file EMBJ-42-e114288-s014.zip › spo13_SourceDataForExpandedView_3/SourceDataForFigureS9/SourceDataForFigureS9A_Imaging/SourceDataForFigureS9A_spo13_ama1.tif]

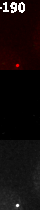

Supplement: Supplementary file 3 — Source Data for Expanded View and Appendix [file EMBJ-42-e114288-s014.zip › spo13_SourceDataForExpandedView_3/SourceDataForFigureEV1/SourceDataForFigureEV1B_Imaging/SourceDataForFigureEV1B_cdc20_ama1_gip1(1-15)-mNG_spo13.tif]

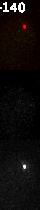

Supplement: Supplementary file 3 — Source Data for Expanded View and Appendix [file EMBJ-42-e114288-s014.zip › spo13_SourceDataForExpandedView_3/SourceDataForFigureEV1/SourceDataForFigureEV1B_Imaging/SourceDataForFigureEV1B_cdc20_ama1_ssp2(1-15)-mNG_spo13.tif]

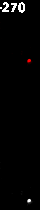

Supplement: Supplementary file 3 — Source Data for Expanded View and Appendix [file EMBJ-42-e114288-s014.zip › spo13_SourceDataForExpandedView_3/SourceDataForFigureEV1/SourceDataForFigureEV1B_Imaging/SourceDataForFigureEV1B_cdc20_ama1_gat4(1-15)-mNG_spo13.tif]

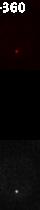

Supplement: Supplementary file 3 — Source Data for Expanded View and Appendix [file EMBJ-42-e114288-s014.zip › spo13_SourceDataForExpandedView_3/SourceDataForFigureEV1/SourceDataForFigureEV1B_Imaging/SourceDataForFigureEV1B_cdc20_ama1_sps4(1-15)-mNG_spo13.tif]

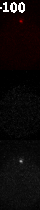

Supplement: Supplementary file 3 — Source Data for Expanded View and Appendix [file EMBJ-42-e114288-s014.zip › spo13_SourceDataForExpandedView_3/SourceDataForFigureEV1/SourceDataForFigureEV1B_Imaging/SourceDataForFigureEV1B_cdc20_ama1_ssp2(1-15)-mNG.tif]

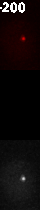

Supplement: Supplementary file 3 — Source Data for Expanded View and Appendix [file EMBJ-42-e114288-s014.zip › spo13_SourceDataForExpandedView_3/SourceDataForFigureEV1/SourceDataForFigureEV1B_Imaging/SourceDataForFigureEV1B_cdc20_ama1_sps4(1-15)-mNG.tif]

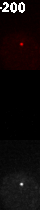

Supplement: Supplementary file 3 — Source Data for Expanded View and Appendix [file EMBJ-42-e114288-s014.zip › spo13_SourceDataForExpandedView_3/SourceDataForFigureEV1/SourceDataForFigureEV1B_Imaging/SourceDataForFigureEV1B_cdc20_ama1_gip1(1-15)-mNG.tif]

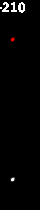

Supplement: Supplementary file 3 — Source Data for Expanded View and Appendix [file EMBJ-42-e114288-s014.zip › spo13_SourceDataForExpandedView_3/SourceDataForFigureEV1/SourceDataForFigureEV1B_Imaging/SourceDataForFigureEV1B_cdc20_ama1_gat4(1-15)-mNG.tif]

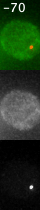

Supplement: Supplementary file 3 — Source Data for Expanded View and Appendix [file EMBJ-42-e114288-s014.zip › spo13_SourceDataForExpandedView_3/SourceDataForFigureEV1/SourceDataForFigureEV1C_Imaging/SourceDataForFigureEV1C_cdc20_ama1.tif]

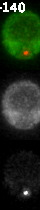

Supplement: Supplementary file 3 — Source Data for Expanded View and Appendix [file EMBJ-42-e114288-s014.zip › spo13_SourceDataForExpandedView_3/SourceDataForFigureEV1/SourceDataForFigureEV1C_Imaging/SourceDataForFigureEV1C_cdc20_ama1_spo13.tif]

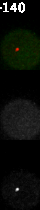

Supplement: Supplementary file 3 — Source Data for Expanded View and Appendix [file EMBJ-42-e114288-s014.zip › spo13_SourceDataForExpandedView_3/SourceDataForFigureEV1/SourceDataForFigureEV1A_Imaging/SourceDataForFigureEV1A_cdc20_ama1(1-30)-mNG.tif]

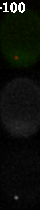

Supplement: Supplementary file 3 — Source Data for Expanded View and Appendix [file EMBJ-42-e114288-s014.zip › spo13_SourceDataForExpandedView_3/SourceDataForFigureEV1/SourceDataForFigureEV1A_Imaging/SourceDataForFigureEV1A_cdc20_ama1(1-30)-mNG_spo13.tif]

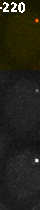

Supplement: Supplementary file 3 — Source Data for Expanded View and Appendix [file EMBJ-42-e114288-s014.zip › spo13_SourceDataForExpandedView_3/SourceDataForFigureS2/SourceDataForFigureS2C_Imaging/SourceDataForFigureS2C_spo13-mD.tif]

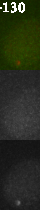

Supplement: Supplementary file 3 — Source Data for Expanded View and Appendix [file EMBJ-42-e114288-s014.zip › spo13_SourceDataForExpandedView_3/SourceDataForFigureS2/SourceDataForFigureS2C_Imaging/SourceDataForFigureS2C_Control.tif]

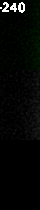

Supplement: Supplementary file 3 — Source Data for Expanded View and Appendix [file EMBJ-42-e114288-s014.zip › spo13_SourceDataForExpandedView_3/SourceDataForFigureS5/SourceDataForFigureS5C_Imaging/SourceDataForFigureS5C_clb3.tif]

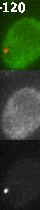

Supplement: Supplementary file 3 — Source Data for Expanded View and Appendix [file EMBJ-42-e114288-s014.zip › spo13_SourceDataForExpandedView_3/SourceDataForFigureS5/SourceDataForFigureS5C_Imaging/SourceDataForFigureS5C_clb1.tif]

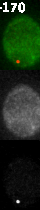

Supplement: Supplementary file 3 — Source Data for Expanded View and Appendix [file EMBJ-42-e114288-s014.zip › spo13_SourceDataForExpandedView_3/SourceDataForFigureS5/SourceDataForFigureS5C_Imaging/SourceDataForFigureS5C_clb1_clb3.tif]

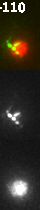

Supplement: Supplementary file 3 — Source Data for Expanded View and Appendix [file EMBJ-42-e114288-s014.zip › spo13_SourceDataForExpandedView_3/SourceDataForFigureS5/SourceDataForFigureS5B_Imaging/SourceDataForFigureS5B_clb1_meta1_long.tif]

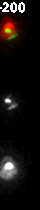

Supplement: Supplementary file 3 — Source Data for Expanded View and Appendix [file EMBJ-42-e114288-s014.zip › spo13_SourceDataForExpandedView_3/SourceDataForFigureS5/SourceDataForFigureS5B_Imaging/SourceDataForFigureS5B_clb1_meta1_short.tif]

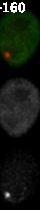

Supplement: Supplementary file 3 — Source Data for Expanded View and Appendix [file EMBJ-42-e114288-s014.zip › spo13_SourceDataForExpandedView_3/SourceDataForFigureS5/SourceDataForFigureS5D_Imaging/SourceDataForFigureS5C_cdk1as.tif]

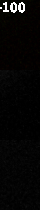

Supplement: Supplementary file 3 — Source Data for Expanded View and Appendix [file EMBJ-42-e114288-s014.zip › spo13_SourceDataForExpandedView_3/SourceDataForFigureS5/SourceDataForFigureS5D_Imaging/SourceDataForFigureS5C_spo13-10A.tif]

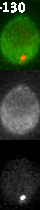

Supplement: Supplementary file 3 — Source Data for Expanded View and Appendix [file EMBJ-42-e114288-s014.zip › spo13_SourceDataForExpandedView_3/SourceDataForFigureS5/SourceDataForFigureS5D_Imaging/SourceDataForFigureS5C_control.tif]

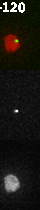

Supplement: Supplementary file 3 — Source Data for Expanded View and Appendix [file EMBJ-42-e114288-s014.zip › spo13_SourceDataForExpandedView_3/SourceDataForFigureS3/SourceDataForFigureS3B_Imaging/SourceDataForFigureS3B_Control.tif]

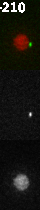

Supplement: Supplementary file 3 — Source Data for Expanded View and Appendix [file EMBJ-42-e114288-s014.zip › spo13_SourceDataForExpandedView_3/SourceDataForFigureS3/SourceDataForFigureS3B_Imaging/SourceDataForFigureS3B_hrr25-dC.tif]

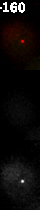

Supplement: Supplementary file 3 — Source Data for Expanded View and Appendix [file EMBJ-42-e114288-s014.zip › spo13_SourceDataForExpandedView_3/SourceDataForFigureS3/SourceDataForFigureS3C_Imaging/SourceDataForFigureS3C_cdc20_ama1(1-30)-mNG_hrr25-dC.tif]

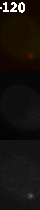

Supplement: Supplementary file 3 — Source Data for Expanded View and Appendix [file EMBJ-42-e114288-s014.zip › spo13_SourceDataForExpandedView_3/SourceDataForFigureS3/SourceDataForFigureS3C_Imaging/SourceDataForFigureS3C_cdc20_ama1(1-30)-mNG.tif]

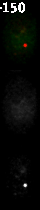

Supplement: Supplementary file 3 — Source Data for Expanded View and Appendix [file EMBJ-42-e114288-s014.zip › spo13_SourceDataForExpandedView_3/SourceDataForFigureS3/SourceDataForFigureS3A_Imaging/SourceDataForFigureS3A_cdc20_ama1(1-30)-mNG_ime2-dC.tif]

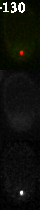

Supplement: Supplementary file 3 — Source Data for Expanded View and Appendix [file EMBJ-42-e114288-s014.zip › spo13_SourceDataForExpandedView_3/SourceDataForFigureS3/SourceDataForFigureS3A_Imaging/SourceDataForFigureS3A_cdc20_ama1(1-30)-mNG.tif]

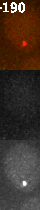

Supplement: Supplementary file 5 — Source Data for Figure 1 [file EMBJ-42-e114288-s009.zip › EMBOJ-2023-114288_SourceDataForFigure1/SourceDataForFigure1D_Imaging/SourceDataForFigure1D_mad2.tif]

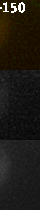

Supplement: Supplementary file 5 — Source Data for Figure 1 [file EMBJ-42-e114288-s009.zip › EMBOJ-2023-114288_SourceDataForFigure1/SourceDataForFigure1D_Imaging/SourceDataForFigure1D_mad2_spo13.tif]

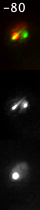

Supplement: Supplementary file 5 — Source Data for Figure 1 [file EMBJ-42-e114288-s009.zip › EMBOJ-2023-114288_SourceDataForFigure1/SourceDataForFigure1A_Imaging/SourceDataForFigure1A_Control.tif]

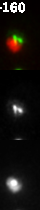

Supplement: Supplementary file 5 — Source Data for Figure 1 [file EMBJ-42-e114288-s009.zip › EMBOJ-2023-114288_SourceDataForFigure1/SourceDataForFigure1A_Imaging/SourceDataForFigure1A_spo13.tif]

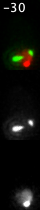

Supplement: Supplementary file 5 — Source Data for Figure 1 [file EMBJ-42-e114288-s009.zip › EMBOJ-2023-114288_SourceDataForFigure1/SourceDataForFigure1B_Imaging/SourceDataForFigure1B_mad2.tif]

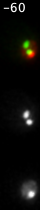

Supplement: Supplementary file 5 — Source Data for Figure 1 [file EMBJ-42-e114288-s009.zip › EMBOJ-2023-114288_SourceDataForFigure1/SourceDataForFigure1B_Imaging/SourceDataForFigure1B_mad2_spo13.tif]

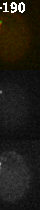

Supplement: Supplementary file 5 — Source Data for Figure 1 [file EMBJ-42-e114288-s009.zip › EMBOJ-2023-114288_SourceDataForFigure1/SourceDataForFigure1C_Imaging/SourceDataForFigure1C_spo13.tif]

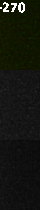

Supplement: Supplementary file 5 — Source Data for Figure 1 [file EMBJ-42-e114288-s009.zip › EMBOJ-2023-114288_SourceDataForFigure1/SourceDataForFigure1C_Imaging/SourceDataForFigure1C_Control.tif]

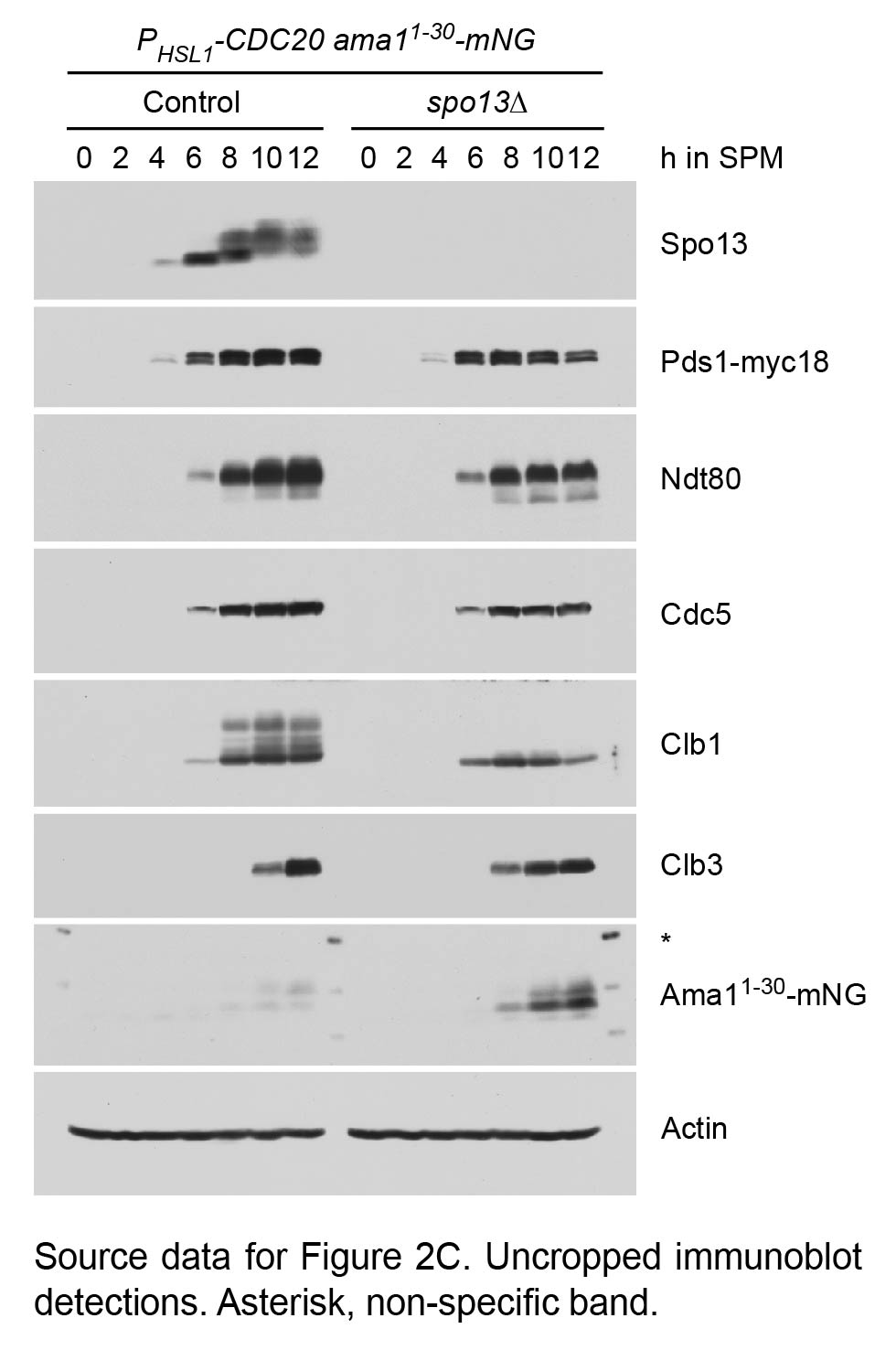

Supplement: Supplementary file 6 — Source Data for Figure 2 [file EMBJ-42-e114288-s008.zip › EMBOJ-2023-114288_SourceDataForFigure2/SourceDataForFigure2C_Blots.jpg]

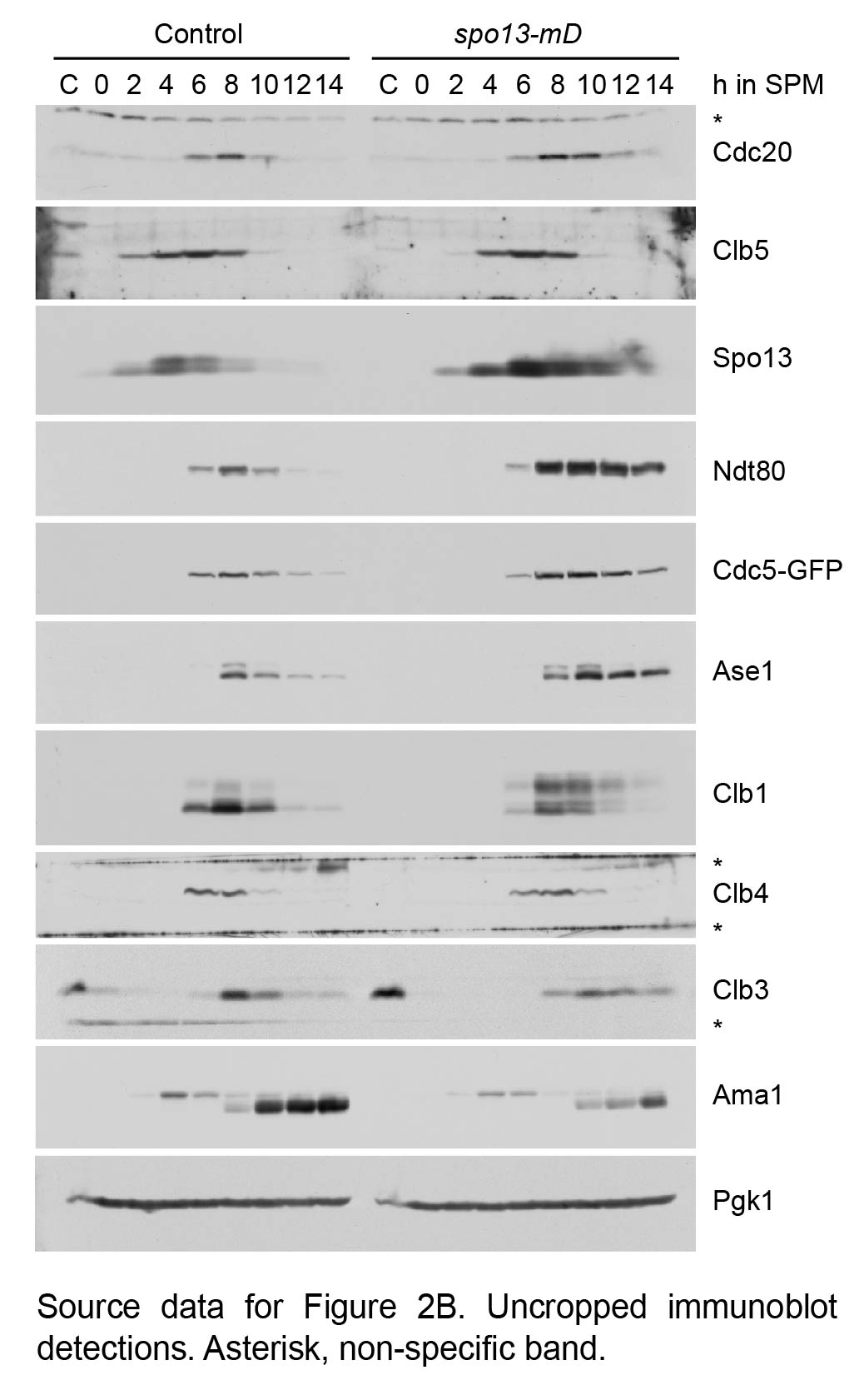

Supplement: Supplementary file 6 — Source Data for Figure 2 [file EMBJ-42-e114288-s008.zip › EMBOJ-2023-114288_SourceDataForFigure2/SourceDataForFigure2B_Blots.jpg]

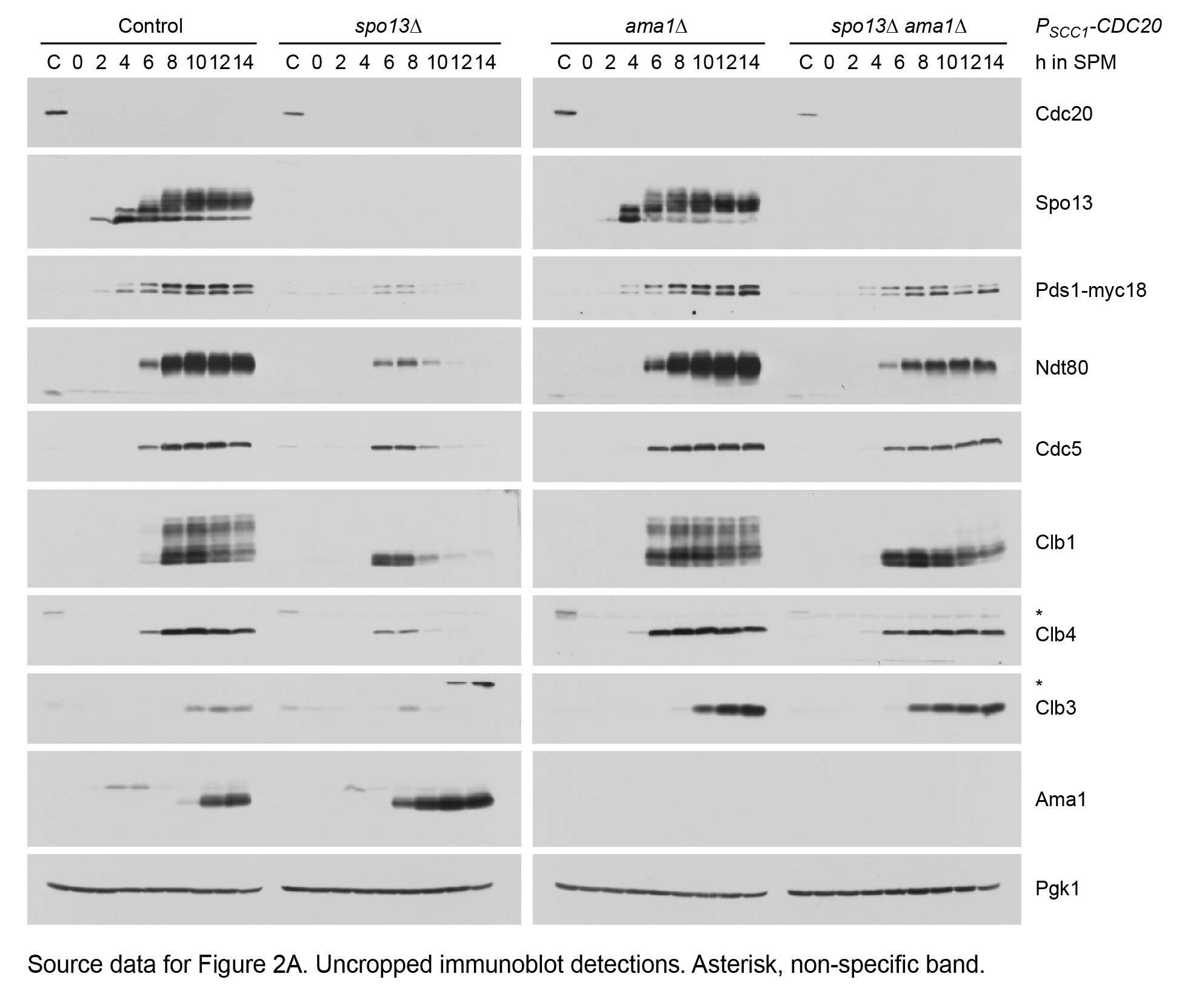

Supplement: Supplementary file 6 — Source Data for Figure 2 [file EMBJ-42-e114288-s008.zip › EMBOJ-2023-114288_SourceDataForFigure2/SourceDataForFigure2A_Blots.jpg]

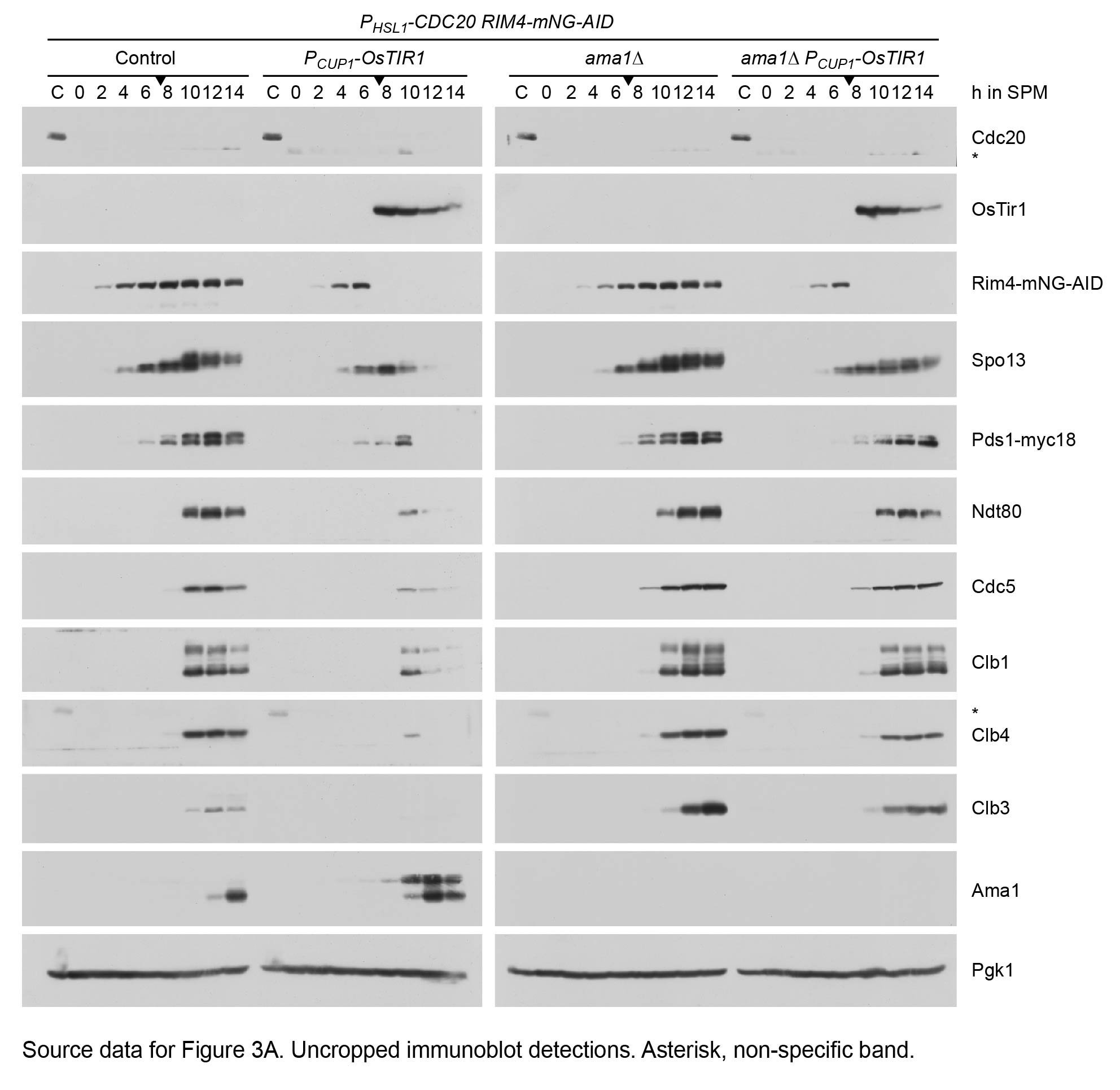

Supplement: Supplementary file 7 — Source Data for Figure 3 [file EMBJ-42-e114288-s007.zip › EMBOJ-2023-114288_SourceDataForFigure3/SourceDataForFigure3A_Blots.jpg]

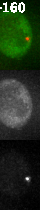

Supplement: Supplementary file 7 — Source Data for Figure 3 [file EMBJ-42-e114288-s007.zip › EMBOJ-2023-114288_SourceDataForFigure3/SourceDataForFigure3C_Imaging/SourceDataForFigure3C_cdc20_spo13.tif]

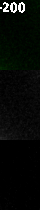

Supplement: Supplementary file 7 — Source Data for Figure 3 [file EMBJ-42-e114288-s007.zip › EMBOJ-2023-114288_SourceDataForFigure3/SourceDataForFigure3C_Imaging/SourceDataForFigure3C_cdc20.tif]

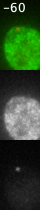

Supplement: Supplementary file 7 — Source Data for Figure 3 [file EMBJ-42-e114288-s007.zip › EMBOJ-2023-114288_SourceDataForFigure3/SourceDataForFigure3B_Imaging/SourceDataForFigure3B_spo13.tif]

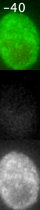

Supplement: Supplementary file 7 — Source Data for Figure 3 [file EMBJ-42-e114288-s007.zip › EMBOJ-2023-114288_SourceDataForFigure3/SourceDataForFigure3B_Imaging/SourceDataForFigure3B_spo13-mD.tif]

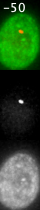

Supplement: Supplementary file 7 — Source Data for Figure 3 [file EMBJ-42-e114288-s007.zip › EMBOJ-2023-114288_SourceDataForFigure3/SourceDataForFigure3B_Imaging/SourceDataForFigure3B_Control.tif]

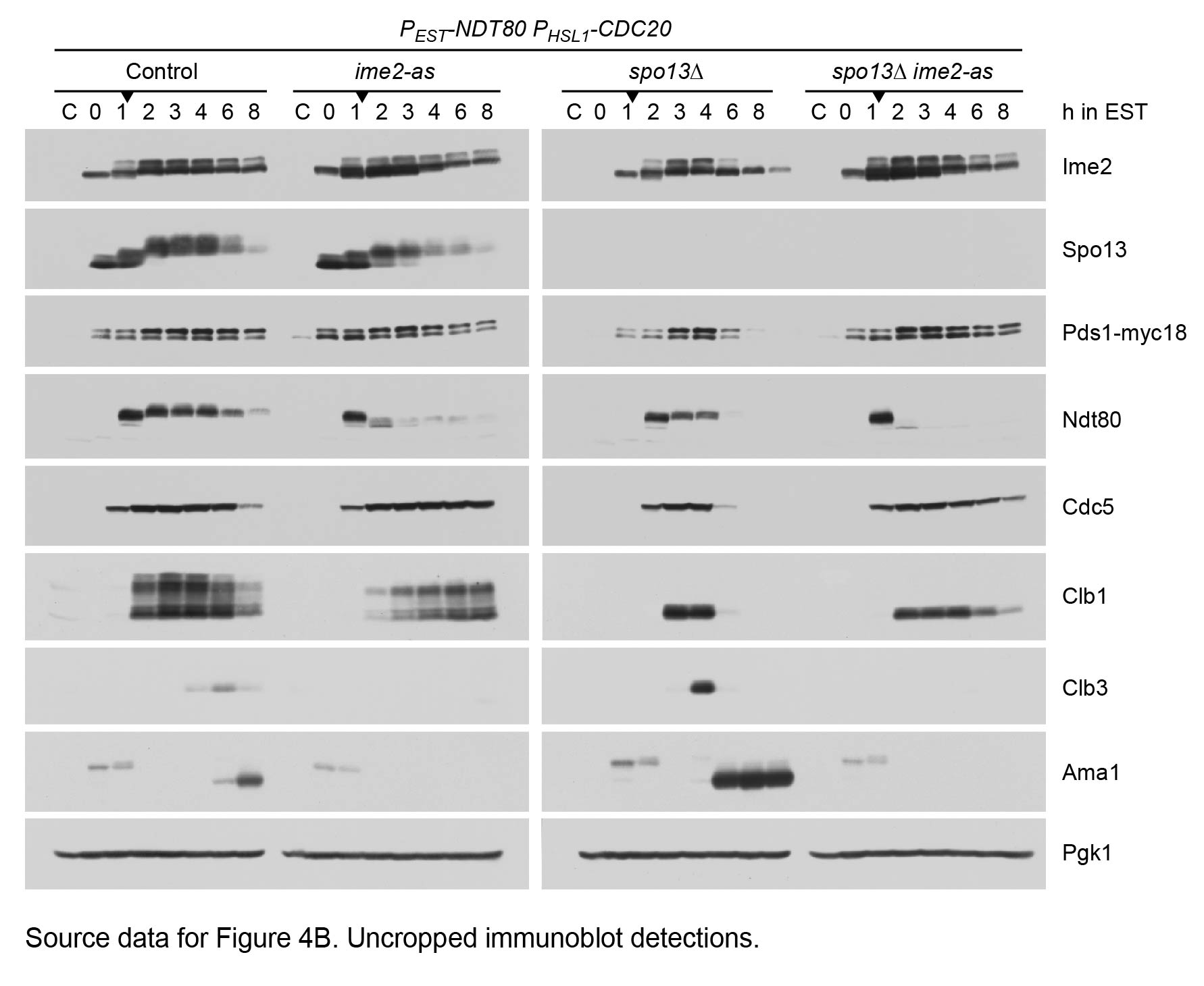

Supplement: Supplementary file 8 — Source Data for Figure 4 [file EMBJ-42-e114288-s006.zip › EMBOJ-2023-114288_SourceDataForFigure4/SourceDataForFigure4B_Blots.jpg]

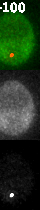

Supplement: Supplementary file 8 — Source Data for Figure 4 [file EMBJ-42-e114288-s006.zip › EMBOJ-2023-114288_SourceDataForFigure4/SourceDataForFigure4C_Imaging/SourceDataForFigure4C_cdc20_ama1_cdc5as.tif]

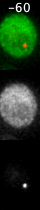

Supplement: Supplementary file 8 — Source Data for Figure 4 [file EMBJ-42-e114288-s006.zip › EMBOJ-2023-114288_SourceDataForFigure4/SourceDataForFigure4C_Imaging/SourceDataForFigure4C_cdc20_ama1_ime2-dC_cdc5as.tif]
