# Supplementary figures and images for: Spo13/MEIKIN ensures a Two‐Division meiosis by preventing the activation of APC/CAma1 at meiosis I (part 2 of 2)
Source: EMBO J. 2023 Sep 20;42(20):e114288. doi: 10.15252/embj.2023114288 (PMC10577557; doi:10.15252/embj.2023114288)

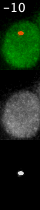

Supplement: Supplementary file 8 — Source Data for Figure 4 [file EMBJ-42-e114288-s006.zip › EMBOJ-2023-114288_SourceDataForFigure4/SourceDataForFigure4C_Imaging/SourceDataForFigure4C_cdc20_ama1_ime2-dC.tif]

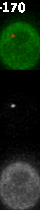

Supplement: Supplementary file 8 — Source Data for Figure 4 [file EMBJ-42-e114288-s006.zip › EMBOJ-2023-114288_SourceDataForFigure4/SourceDataForFigure4A_Imaging/SourceDataForFigure4A_cdc20_ama1_spo13.tif]

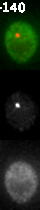

Supplement: Supplementary file 8 — Source Data for Figure 4 [file EMBJ-42-e114288-s006.zip › EMBOJ-2023-114288_SourceDataForFigure4/SourceDataForFigure4A_Imaging/SourceDataForFigure4A_cdc20_ama1_spo13_ime2as.tif]

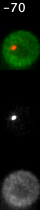

Supplement: Supplementary file 8 — Source Data for Figure 4 [file EMBJ-42-e114288-s006.zip › EMBOJ-2023-114288_SourceDataForFigure4/SourceDataForFigure4A_Imaging/SourceDataForFigure4A_cdc20_ama1_ime2as.tif]

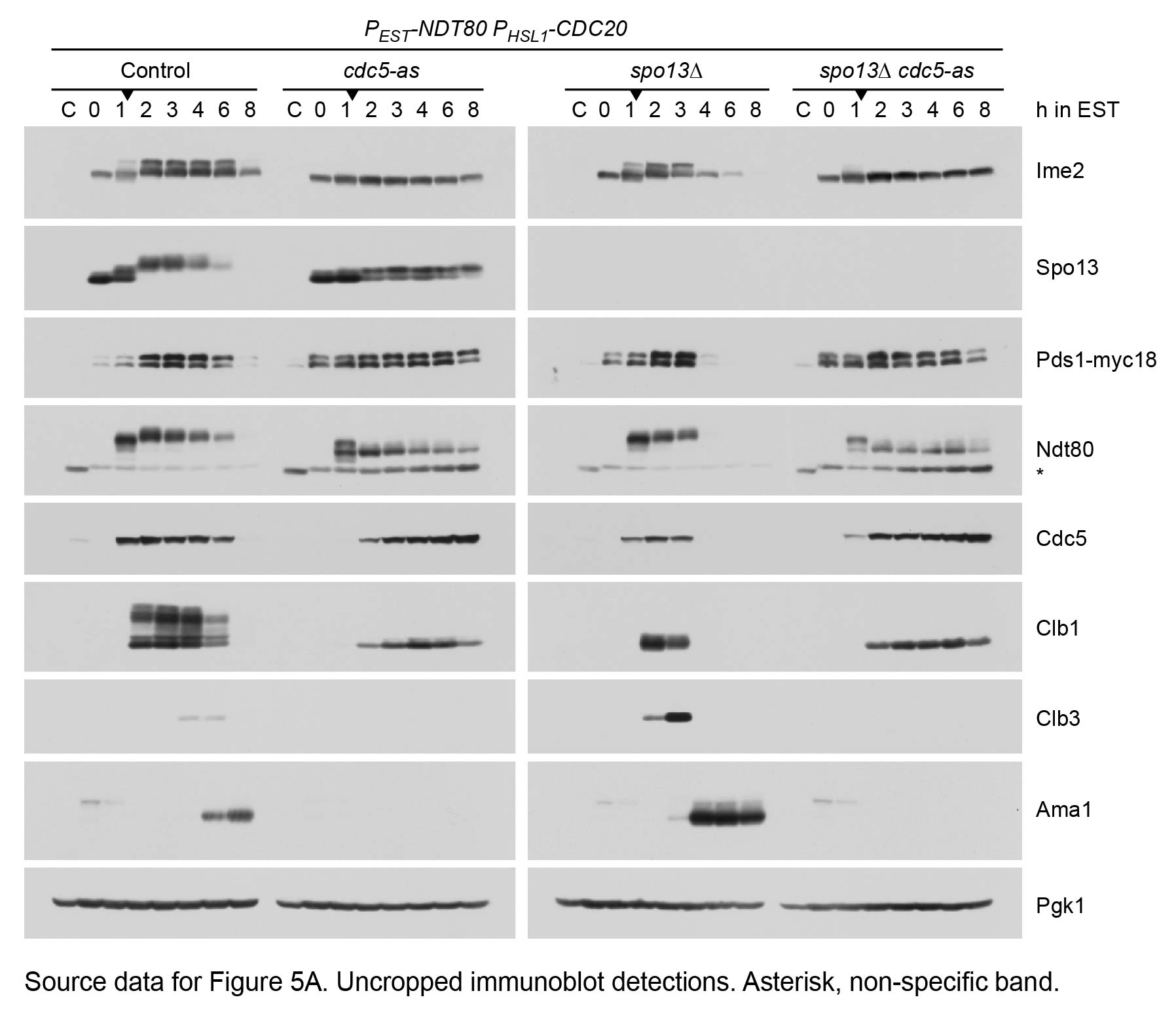

Supplement: Supplementary file 9 — Source Data for Figure 5 [file EMBJ-42-e114288-s004.zip › EMBOJ-2023-114288_SourceDataForFigure5/SourceDataForFigure5A_Blots.jpg]

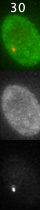

Supplement: Supplementary file 9 — Source Data for Figure 5 [file EMBJ-42-e114288-s004.zip › EMBOJ-2023-114288_SourceDataForFigure5/SourceDataForFigure5B_Imaging/SourceDataForFigure5B_ndt80_spo13_ESTp-CDC5_ime2as.tif]

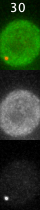

Supplement: Supplementary file 9 — Source Data for Figure 5 [file EMBJ-42-e114288-s004.zip › EMBOJ-2023-114288_SourceDataForFigure5/SourceDataForFigure5B_Imaging/SourceDataForFigure5B_ndt80_spo13.tif]

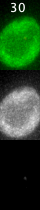

Supplement: Supplementary file 9 — Source Data for Figure 5 [file EMBJ-42-e114288-s004.zip › EMBOJ-2023-114288_SourceDataForFigure5/SourceDataForFigure5B_Imaging/SourceDataForFigure5B_ndt80_spo13_ESTp-CDC5.tif]

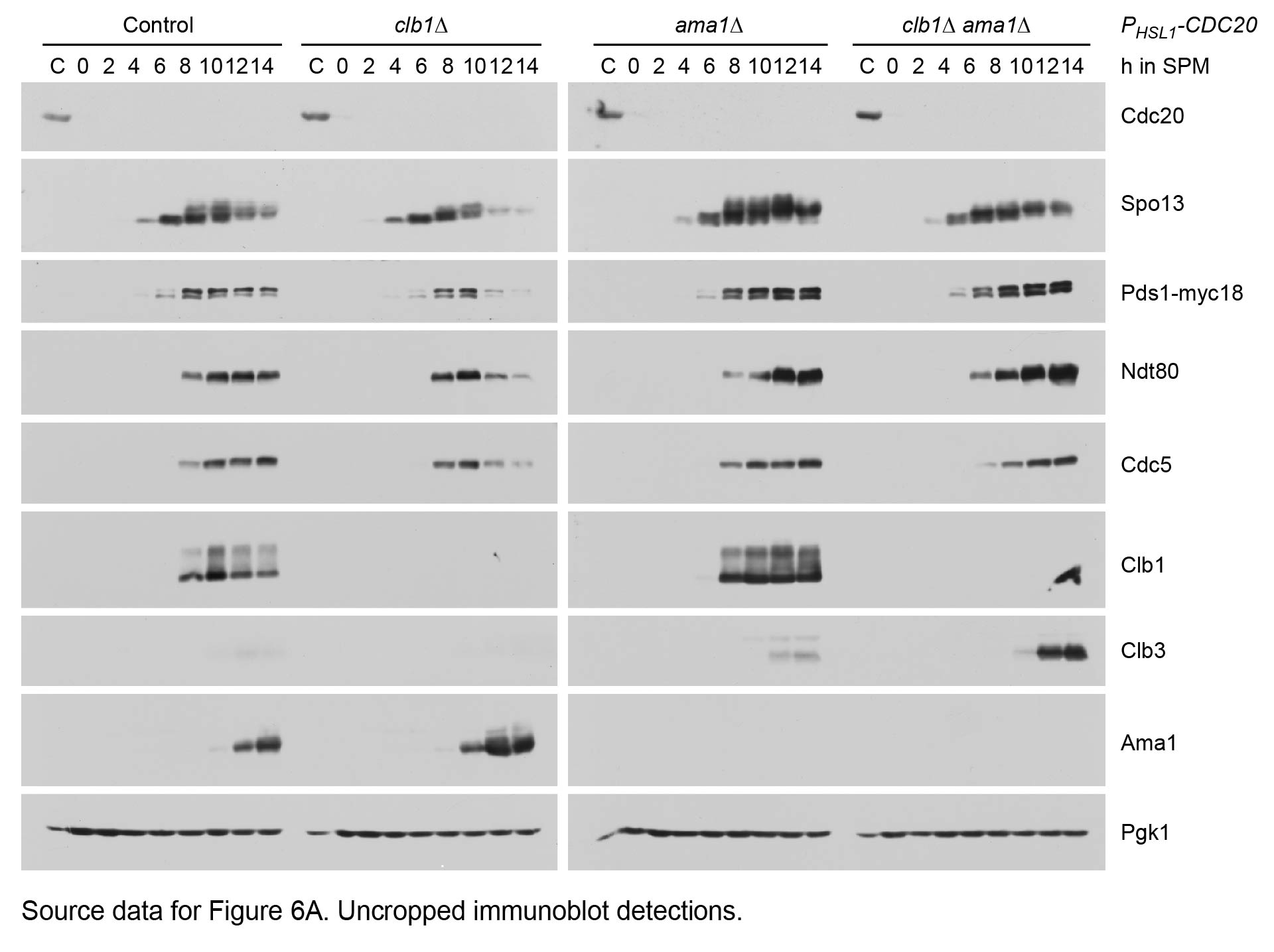

Supplement: Supplementary file 10 — Source Data for Figure 6 [file EMBJ-42-e114288-s013.zip › EMBOJ-2023-114288_SourceDataForFigure6/SourceDataForFigure6A_Blots.jpg]

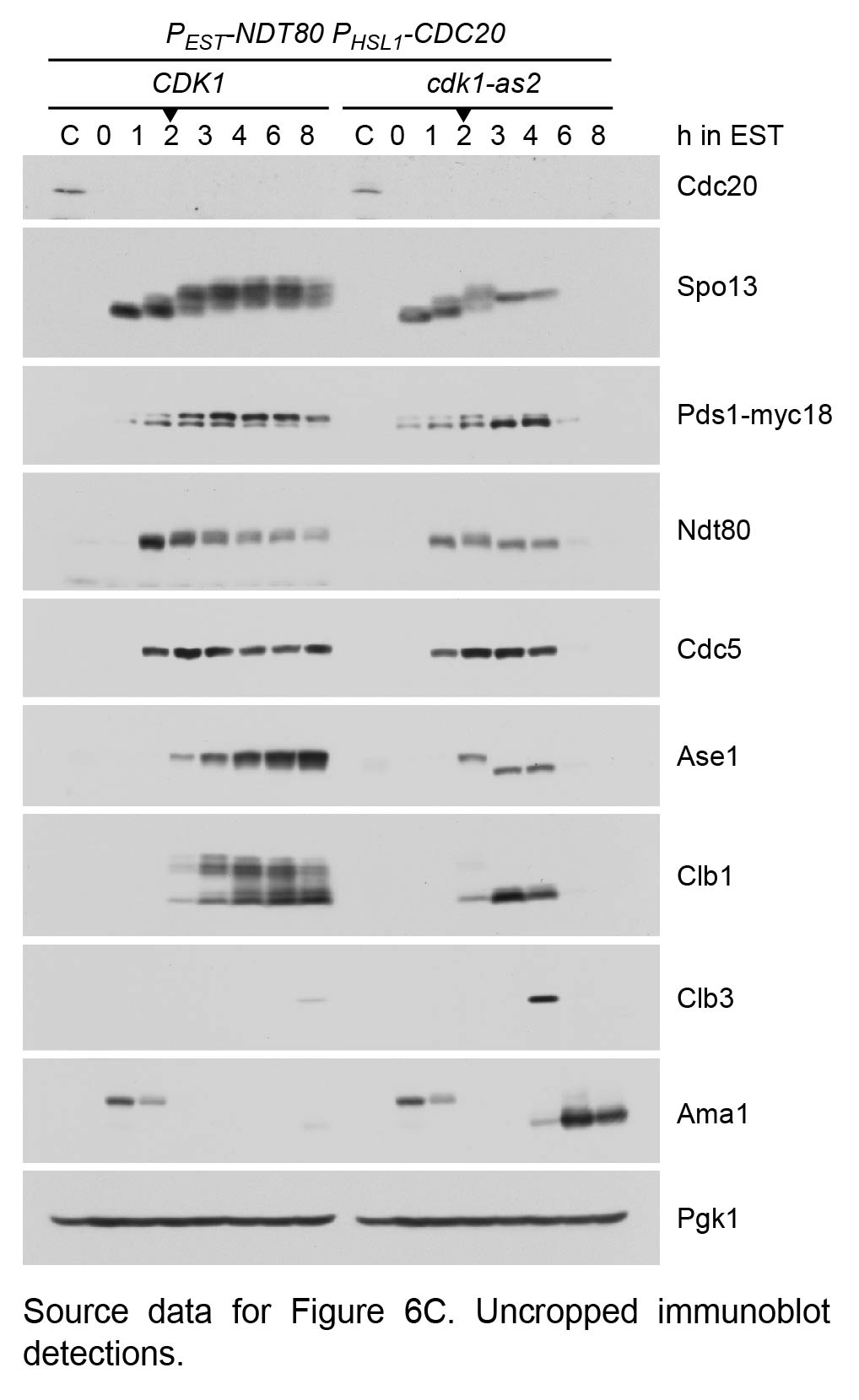

Supplement: Supplementary file 10 — Source Data for Figure 6 [file EMBJ-42-e114288-s013.zip › EMBOJ-2023-114288_SourceDataForFigure6/SourceDataForFigure6C_Blots.jpg]

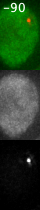

Supplement: Supplementary file 10 — Source Data for Figure 6 [file EMBJ-42-e114288-s013.zip › EMBOJ-2023-114288_SourceDataForFigure6/SourceDataForFigure6B_Imaging/SourceDataForFigure6B_cdc20_clb1.tif]

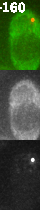

Supplement: Supplementary file 10 — Source Data for Figure 6 [file EMBJ-42-e114288-s013.zip › EMBOJ-2023-114288_SourceDataForFigure6/SourceDataForFigure6B_Imaging/SourceDataForFigure6B_cdc20_cdk1as2.tif]

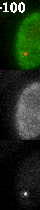

Supplement: Supplementary file 10 — Source Data for Figure 6 [file EMBJ-42-e114288-s013.zip › EMBOJ-2023-114288_SourceDataForFigure6/SourceDataForFigure6B_Imaging/SourceDataForFigure6B_cdc20_spo13-10A.tif]

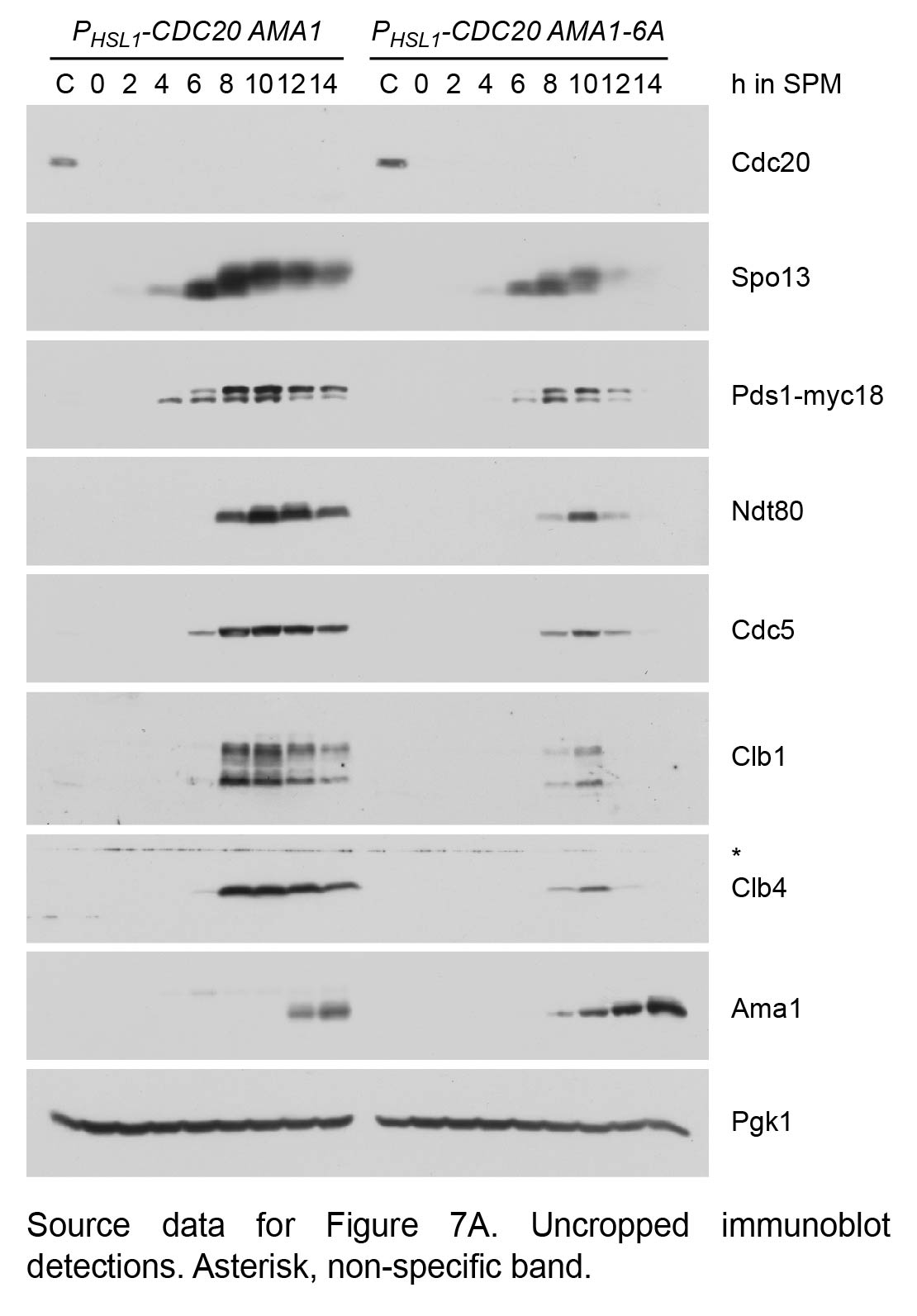

Supplement: Supplementary file 11 — Source Data for Figure 7 [file EMBJ-42-e114288-s002.zip › EMBOJ-2023-114288_SourceDataForFigure7/SourceDataForFigure7A_Blots.jpg]

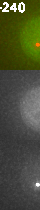

Supplement: Supplementary file 11 — Source Data for Figure 7 [file EMBJ-42-e114288-s002.zip › EMBOJ-2023-114288_SourceDataForFigure7/SourceDataForFigure7D_Imaging/SourceDataForFigure7D_cdc20_AMA1-6A_ime2as.tif]

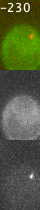

Supplement: Supplementary file 11 — Source Data for Figure 7 [file EMBJ-42-e114288-s002.zip › EMBOJ-2023-114288_SourceDataForFigure7/SourceDataForFigure7D_Imaging/SourceDataForFigure7D_cdc20_AMA1-6A_cdc5as.tif]

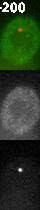

Supplement: Supplementary file 11 — Source Data for Figure 7 [file EMBJ-42-e114288-s002.zip › EMBOJ-2023-114288_SourceDataForFigure7/SourceDataForFigure7D_Imaging/SourceDataForFigure7D_cdc20_AMA1-6A_hrr25as.tif]

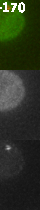

Supplement: Supplementary file 11 — Source Data for Figure 7 [file EMBJ-42-e114288-s002.zip › EMBOJ-2023-114288_SourceDataForFigure7/SourceDataForFigure7C_Imaging/SourceDataForFigure7B_cdc20_AMA1-6A_spo13mD.tif]

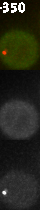

Supplement: Supplementary file 11 — Source Data for Figure 7 [file EMBJ-42-e114288-s002.zip › EMBOJ-2023-114288_SourceDataForFigure7/SourceDataForFigure7C_Imaging/SourceDataForFigure7B_cdc20_AMA1-6A_spo13mD_clb1mDK.tif]

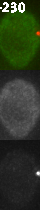

Supplement: Supplementary file 11 — Source Data for Figure 7 [file EMBJ-42-e114288-s002.zip › EMBOJ-2023-114288_SourceDataForFigure7/SourceDataForFigure7C_Imaging/SourceDataForFigure7B_cdc20_AMA1-6A_clb1mDK.tif]

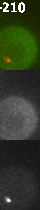

Supplement: Supplementary file 11 — Source Data for Figure 7 [file EMBJ-42-e114288-s002.zip › EMBOJ-2023-114288_SourceDataForFigure7/SourceDataForFigure7B_Imaging/SourceDataForFigure7B_cdc20_AMA1.tif]

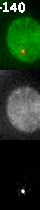

Supplement: Supplementary file 11 — Source Data for Figure 7 [file EMBJ-42-e114288-s002.zip › EMBOJ-2023-114288_SourceDataForFigure7/SourceDataForFigure7B_Imaging/SourceDataForFigure7B_cdc20_AMA1-6A.tif]

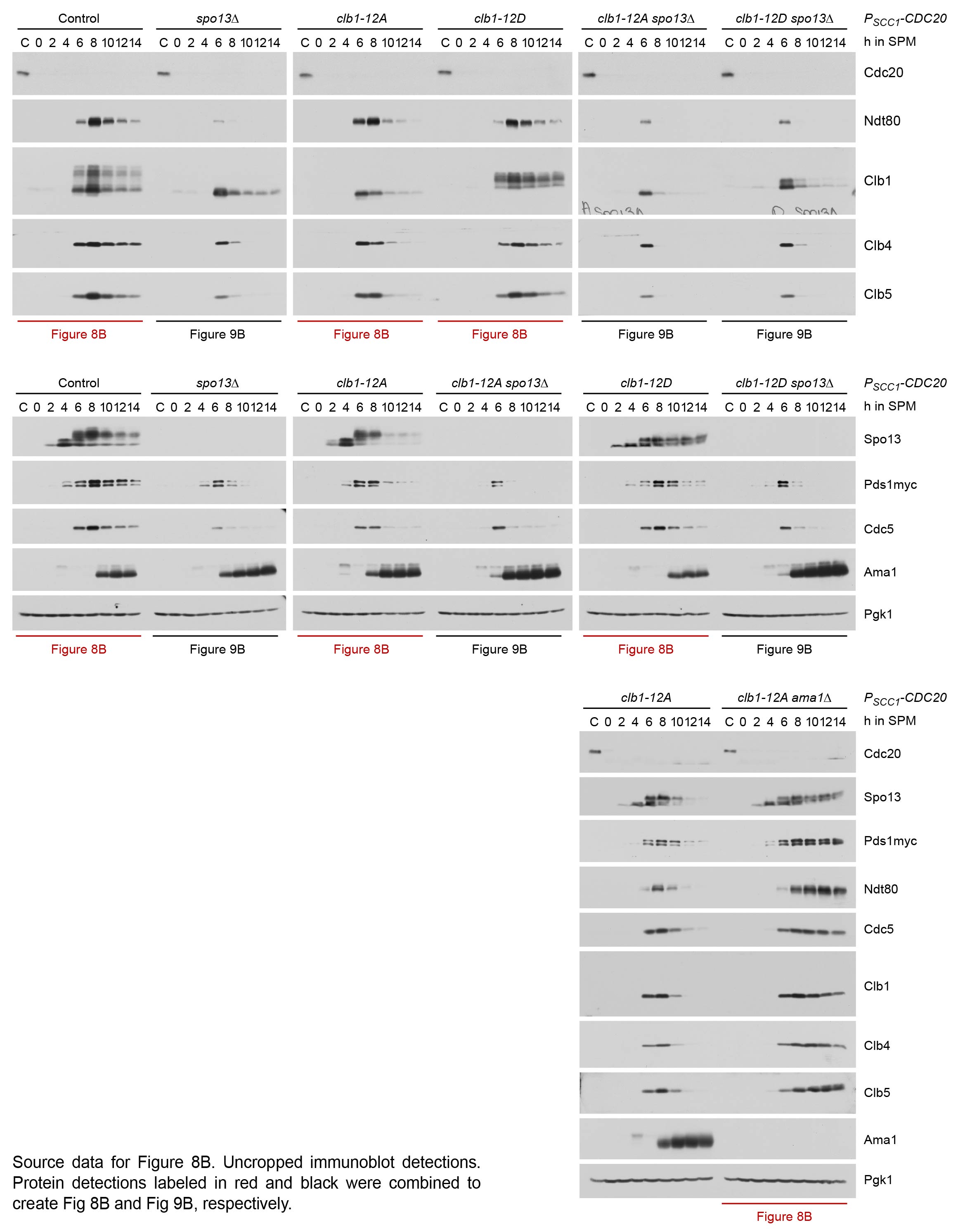

Supplement: Supplementary file 12 — Source Data for Figure 8 [file EMBJ-42-e114288-s001.zip › EMBOJ-2023-114288_SourceDataForFigure8/SourceDataForFigure8B_Blots.jpg]

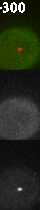

Supplement: Supplementary file 12 — Source Data for Figure 8 [file EMBJ-42-e114288-s001.zip › EMBOJ-2023-114288_SourceDataForFigure8/SourceDataForFigure8C_Imaging/SourceDataForFigure8C_cdc20.tif]

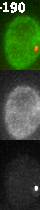

Supplement: Supplementary file 12 — Source Data for Figure 8 [file EMBJ-42-e114288-s001.zip › EMBOJ-2023-114288_SourceDataForFigure8/SourceDataForFigure8C_Imaging/SourceDataForFigure8C_cdc20_clb1-12D.tif]

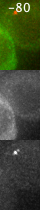

Supplement: Supplementary file 12 — Source Data for Figure 8 [file EMBJ-42-e114288-s001.zip › EMBOJ-2023-114288_SourceDataForFigure8/SourceDataForFigure8C_Imaging/SourceDataForFigure8C_cdc20_clb1-12A.tif]

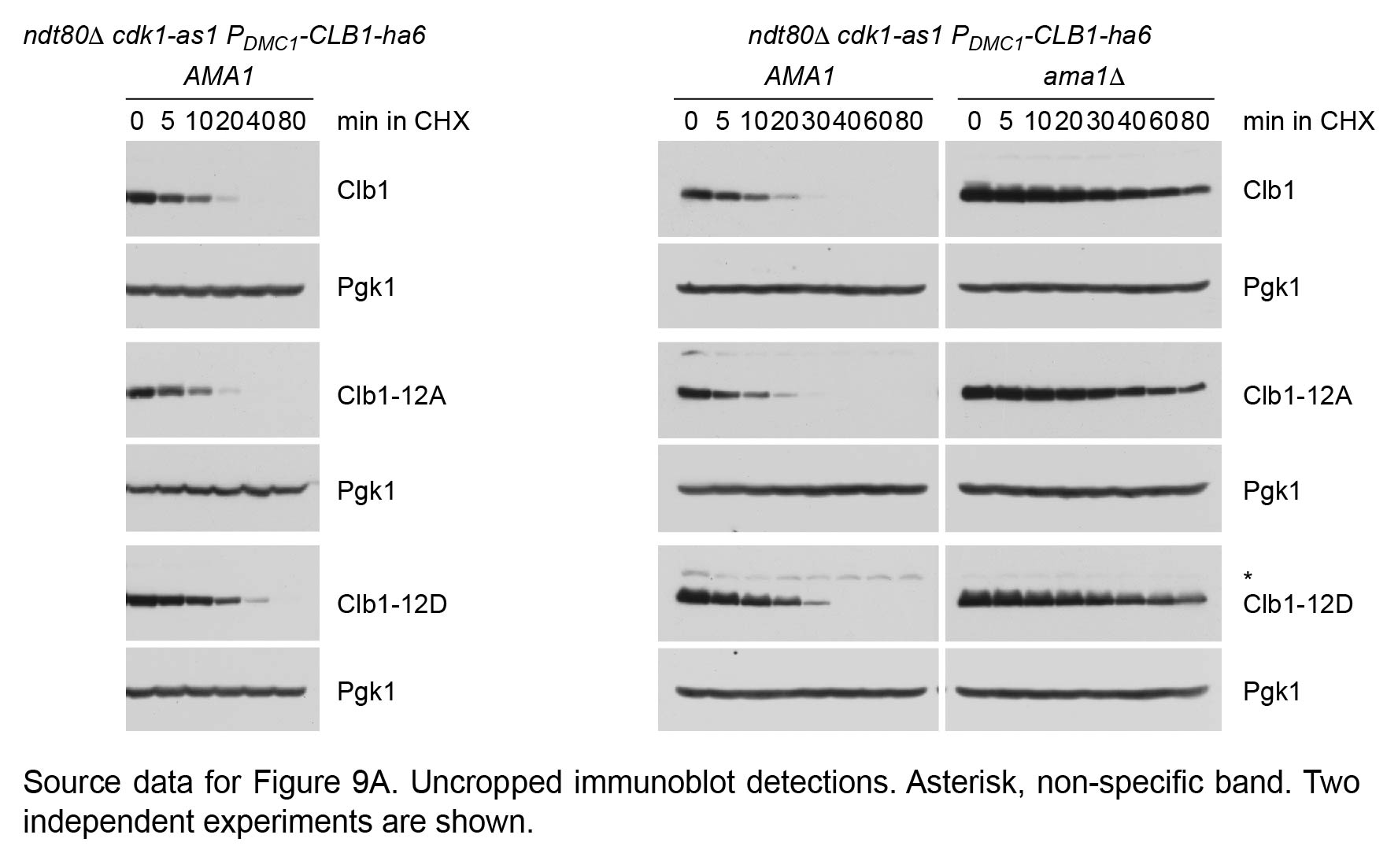

Supplement: Supplementary file 13 — Source Data for Figure 9 [file EMBJ-42-e114288-s003.zip › EMBOJ-2023-114288_SourceDataForFigure9/SourceDataForFigure9A_Blots.jpg]

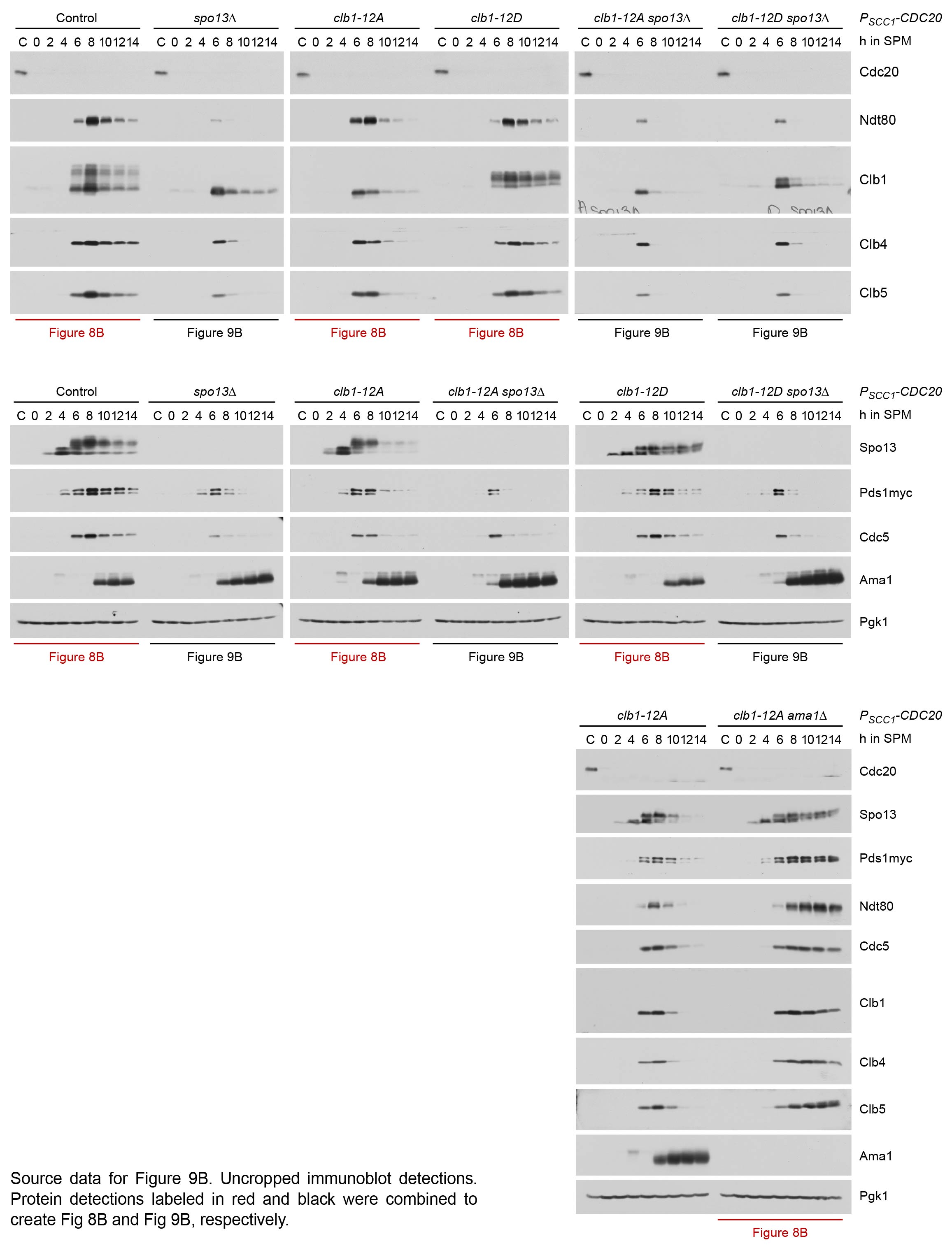

Supplement: Supplementary file 13 — Source Data for Figure 9 [file EMBJ-42-e114288-s003.zip › EMBOJ-2023-114288_SourceDataForFigure9/SourceDataForFigure9B_Blots.jpg]

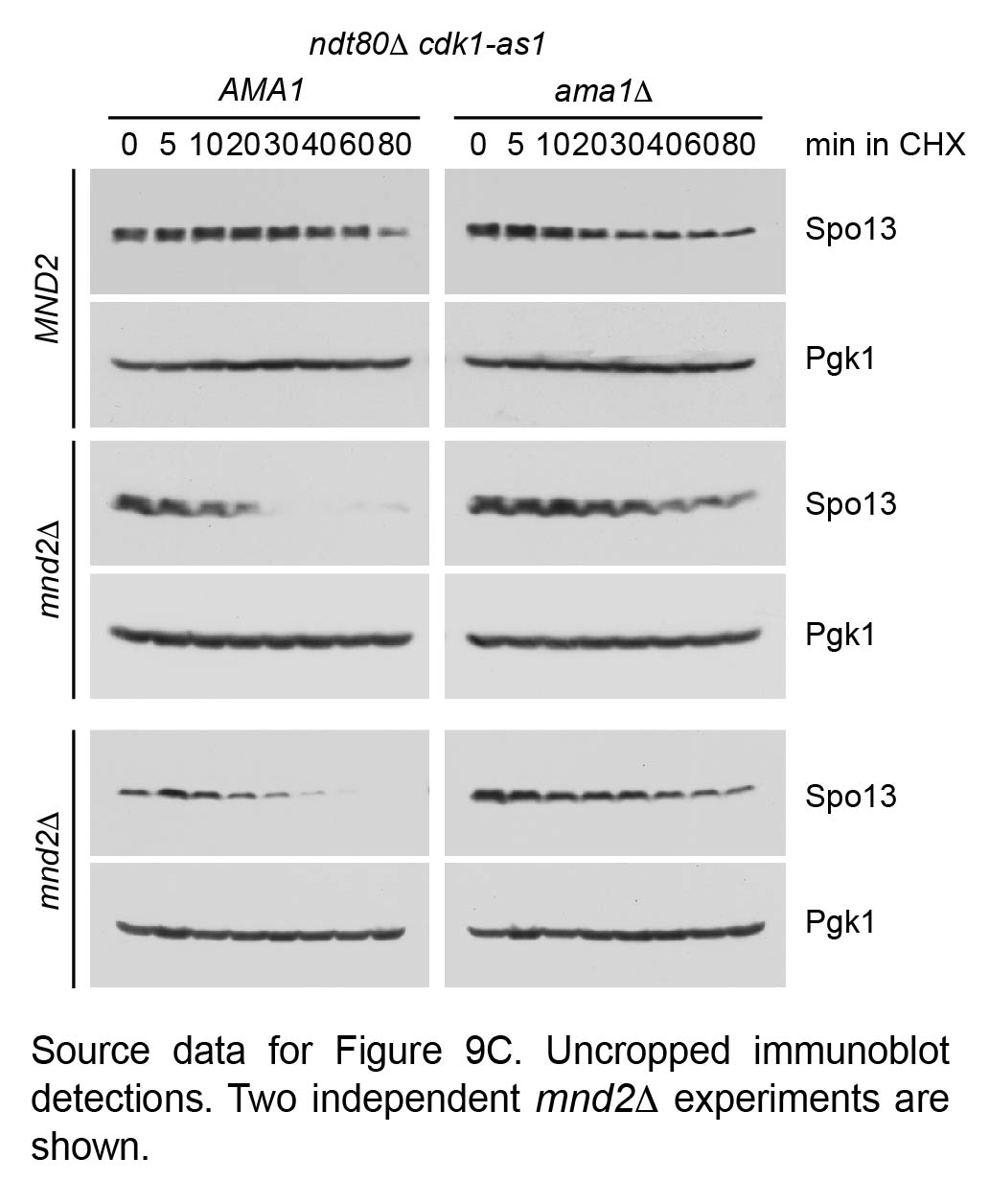

Supplement: Supplementary file 13 — Source Data for Figure 9 [file EMBJ-42-e114288-s003.zip › EMBOJ-2023-114288_SourceDataForFigure9/SourceDataForFigure9C_Blots.jpg]
