# Supplementary material for: Spo13/MEIKIN ensures a Two‐Division meiosis by preventing the activation of APC/CAma1 at meiosis I
Source: EMBO J. 2023 Sep 20;42(20):e114288. doi: 10.15252/embj.2023114288 (PMC10577557; doi:10.15252/embj.2023114288)
Supplement: Supplementary file 14 [file EMBJ-42-e114288-s012.pdf]

THE EMBO JOURNAL

Spo13/Meikin ensures a two-division meiosis by preventing the activation of APC/C-Ama1 at meiosis I

Wolfgang Zachariae, Julie Rojas, Tugce Oz, Katarzyna Jonak, Oleksii Lyzak, Vinal Massaad, and Olha Biriuk
DOI: 10.15252/embj.2023114288

Corresponding author(s): Wolfgang Zachariae (zachar@biochem.mpg.de)

Review Timeline:

Submission Date:	14th Apr 23
Editorial Decision:	22nd May 23
Revision Received:	16th Aug 23
Editorial Decision:	1st Sep 23
Revision Received:	3rd Sep 23
Accepted:	4th Sep 23

Editor: Hartmut Vodermaier

Transaction Report:

(Note: With the exception of the correction of typographical or spelling errors that could be a source of ambiguity, letters and reports are not edited. Depending on transfer agreements, referee reports obtained elsewhere may or may not be included in this compilation. Referee reports are anonymous unless the Referee chooses to sign their reports.)

1st Editorial Decision **22nd May 2023**

Dr. Wolfgang Zachariae
Max Planck Institute of Biochemistry
Laboratory of Chromosome Biology
Am Klopferspitz 18
Martinsried 82152
Germany

22nd May 2023

Re: EMBOJ-2023-114288
Spo13/Meikin ensures a two-division meiosis by preventing the activation of APC/C-Ama1 at meiosis I

Dear Wolfgang,

Thank you for submitting your study on how cells ensure meiosis exit only after the second meiotic division, to The EMBO Journal. It has now been assessed by three expert referees, whose comments are copied below for your information. As you will see, all referees consider this an important question and your results potentially interesting, Consequently, they are all supportive of publication, pending revision of a limited number of specific points noted in the three reports. I would therefore like to invite you to address these points and resubmit a revised manuscript, taking into account also the detailed guidelines below and on our website.

I should remind you that it is our policy to allow only a single round of (major) revision, making it important to carefully respond to all points raised at this stage; therefore, please do not hesitate to contact me in case you would like to discuss any of the issues raised by the reviewers. Also, should revision require more time than our default three-months revision period, we would be open to offering an extension, during which our 'scooping protection' (meaning that competing work appearing elsewhere in the meantime will not affect our considerations of your study) would of course remain valid.

Thank you again for the opportunity to consider this work for The EMBO Journal, and I look forward to your revision!

With kind regards,

Hartmut

Hartmut Vodermaier, PhD
Senior Editor, The EMBO Journal
h.vodermaier@embojournal.org

*** PLEASE NOTE: All revised manuscript are subject to initial checks for completeness and adherence to our formatting guidelines. Revisions may be returned to the authors and delayed in their editorial re-evaluation if they fail to comply to the following requirements (see also our Guide to Authors for further information):

1) Every manuscript requires a Data Availability section (even if only stating that no deposited datasets are included). Primary datasets or computer code produced in the current study have to be deposited in appropriate public repositories prior to resubmission, and reviewer access details provided in case that public access is not yet allowed. Further information: embopress.org/page/journal/14602075/authorguide#dataavailability

2) Each figure legend must specify
- size of the scale bars that are mandatory for all micrograph panels
- the statistical test used to generate error bars and P-values
- the type error bars (e.g., S.E.M., S.D.)
- the number (n) and nature (biological or technical replicate) of independent experiments underlying each data point
- Figures may not include error bars for experiments with n<3; scatter plots showing individual data points should be used instead.

3) Revised manuscript text (including main tables, and figure legends for main and EV figures) has to be submitted as editable text file (e.g., .docx format). We encourage highlighting of changes (e.g., via text color) for the referees' reference.

4) Each main and each Expanded View (EV) figure should be uploaded as individual production-quality files (preferably in .eps, .tif, .jpg formats). For suggestions on figure preparation/layout, please refer to our Figure Preparation Guidelines:
http://bit.ly/EMBOPressFigurePreparationGuideline

5) Point-by-point response letters should include the original referee comments in full together with your detailed responses to them (and to specific editor requests if applicable), and also be uploaded as editable (e.g., .docx) text files.

6) Please complete our Author Checklist, and make sure that information entered into the checklist is also reflected in the manuscript; the checklist will be available to readers as part of the Review Process File. A download link is found at the top of our Guide to Authors: embopress.org/page/journal/14602075/authorguide

7) All authors listed as (co-)corresponding need to deposit, in their respective author profiles in our submission system, a unique ORCiD identifier linked to their name. Please see our Guide to Authors for detailed instructions.

8) Please note that supplementary information at EMBO Press has been superseded by the 'Expanded View' for inclusion of additional figures, tables, movies or datasets; with up to five EV Figures being typeset and directly accessible in the HTML version of the article. For details and guidance, please refer to:
embopress.org/page/journal/14602075/authorguide#expandedview

9) Digital image enhancement is acceptable practice, as long as it accurately represents the original data and conforms to community standards. If a figure has been subjected to significant electronic manipulation, this must be clearly noted in the figure legend and/or the 'Materials and Methods' section. The editors reserve the right to request original versions of figures and the original images that were used to assemble the figure. Finally, we generally encourage uploading of numerical as well as gel/blot image source data; for details see: embopress.org/page/journal/14602075/authorguide#sourcedata

At EMBO Press, we ask authors to provide source data for the main manuscript figures. Our source data coordinator will contact you to discuss which figure panels we would need source data for and will also provide you with helpful tips on how to upload and organize the files.

Further information is available in our Guide For Authors:
https://www.embopress.org/page/journal/14602075/authorguide

In the interest of ensuring the conceptual advance provided by the work, we recommend submitting a revision within 3 months (20th Aug 2023). Please discuss the revision progress ahead of this time with the editor if you require more time to complete the revisions. Use the link below to submit your revision:

Link Not Available

Referee #1:

The work by Rojas et al addresses important open questions about the regulation of meiosis and it provides important answers that start to outline a coherent picture about the central regulatory mechanisms that enable the cell to synchronize the two consecutive chromosome separations, in MI and MII. I found the paper a fantastic piece of work that addresses in a very dense but scientifically stringent manner the question, how the cell cycle machinery in meiosis is tuned in such a way that it enables faithful progression from Meiosis I to Meiosis II without exiting the cell cycle. In essence, the work demonstrates that Spo13, in combination with Cdc5 functions as a specific inhibitor of meiosis I processes to timely regulate the activity of the Apc/C Cdc20 and Ama1. A particular role was found for the Spo13-Cdc5 complex to prevent premature activation of various processes while the divisions are still ongoing, in particular by the inhibition of degradation of Rim4 to prevent translation of Rim4 bound transcripts. This paper is a masterpiece of clean experimental design. It combines elegant genetic manipulations with systematic exploration of every aspect of the process by using tricks to expose individual regulatory system and by this to identify how the different branches are connected. With this it also nicely complements previous work by the same group that already demonstrated another important aspect of meiosis regulation, namely the prevention of spore formation at the transition from Meiosis I to meiosis II. I recommend this paper enthusiastically for publication in EMBO J without further experimentation.
Minor points:
Page 5:
I anyway struggled a bit with this paragraph that refers to Ama1 function, in particular the sentence "Three properties of Ama1 might make this a challenging task: First, while Ama1 accumulates at metaphase II of an unperturbed meiosis, it nevertheless appears when metaphase I is prolonged (Oelschlaegel et al., 2005)." Could this be explained a bit better? In context I it could be helpful if the Spo13-Cdc5 interaction would also be more thoroughly introduced.
General:
I was wondering whether the authors could summarize their findings by extending their wiring diagrams from Figure 9D from their previous paper (Oz et al., 2022)? A paper of this density is hard to comprehend, the wiring diagrams could - maybe be

combined with schematic outline the cell structures (spindles, nuclei) in the different phases of Mi-MII? - help the reader to put the pieces together.
Table S1 or Methods: please provide the name and original reference of the parental SK1 strain that is used for this study.

Referee #2:

In the submitted manuscript, Rojas et al investigate the function of Spo13 in yeast meiosis. Loss of Spo13 causes a single meiotic division, but only when metaphase I is long. Under conditions of SAC-inhibited Cdc20, Spo13 prevents Ama-APC/C activation. Spo13 itself is degraded by Cdc20-APC/C at anaphase I and degradation of Spo13 is required for Ama1 accumulation in MII and degradation of Ama1 substrates. Together with Cdc5, Spo13 prevents Ime2 from mediating the degradation of the translational repressor Rim4. This kinase cascade additionally involves Hrr25. Cdk1/Clb1 promotes both the stabilization and activity of Spo13, while it restrains activity and synthesis of Ama1. Cdc5/Spo13 in turn phosphorylate Clb1, a prerequisite for Ama-APC/C inhibition. Notably, the Ama1-APC/C inhibitory protein Mnd2 allows Spo13 accumulation in MI by protecting it from Ama1-APC/C mediated degradation.
This ms addresses a highly an interesting and relevant question, i.e., why do cells exit meiosis after second rather than after the first meiotic division. As one has to expect from the Zachariae lab, the presented data are of highest quality, despite their enormous complexity. WB analyses in combination with fluorescent microscopy studies are applied to investigate the consequences of the different manipulations on meiotic cell cycle progression. All conclusions are in strict accordance with the obtained results and confirmed as well as extended by additional control experiments. This reviewer highly recommends publication of this excellent paper, once the authors have addressed the following points:
- Fig. S2A: the depletion of Cdc20 results in a metaphase arrest with stable Cdc5. Cdc5 is destabilized in the absence of SPO13 and the authors state that this does not happen when CDH1 is depleted. However, in S2A, Cdc5 is also destabilized to some extent in the absence of both CDC20 and CDH1. This seems to correlate with a partial decrease in Spo13 levels, which however does not correspond to an increase in Ama1 protein. Can the authors explain this behavior?

- One key mechanism revealed in this study is that free Cdc5 activates Ime2, whereas Spo13-bound Cdc5 inhibits it. Although the cell-based evidence is convincing, it would be very nice to see if this can also be recapitulated in Ime2 in vitro kinase assays in the presence of free Cdc5 / Spo13-bound Cdc5. This could then also help to get a better understanding how this surprising difference in substrate regulation by Cdc5 is established on the molecular level (e.g. which sites are phosphorylated in both cases).

- Fig. 7A: the slower-migrating, phosphorylated species of Ama1 (wild-type) is detectable 2h before Clb1 (or Clb4) becomes evidently expressed. Furthermore, Ama1 seems to get dephosphorylated when the Clb1 levels reach a maximum (at t=8h). How can this be explained? Are there other possible kinases phosphorylating the six S/TP sites?

- Page 15: The authors write "Ama6-A"

- Fig. 8B: the authors identify by MS several sites on Clb1 potentially phosphorylated by Cdc5, and all of them are serine residues. The authors could further analyze if the sites fit the canonical Polo-consensus motif or if there are other obvious similarities between the sites that allow their phosphorylation by Cdc5.

- Fig. 8B: the authors observe that "the clb1-12D mutant persists in the presence of Ama1, similar to phosphorylated wild-type Clb1". However, I find it hard to tell if the dephosphorylated wild-type form is less stable than the phosphorylated wild-type form. Indeed, to me both phosphorylated/dephosphorylated wild-type form seem to get degraded with more or less similar efficiencies. Can this be quantified?

- Fig. 8: the finding that phosphorylated Clb1 inhibits APC/C(Ama1) is very interesting. If possible, it would be very nice to recapitulate this finding (and the dependency on Cdc5-Spo13) in an in vitro ubiquitination assay, to show that these are indeed direct effects. If this experiment is too demanding, I recommend adding some ideas about how direct this effect is in the discussion.

- Page 29: in their discussion about the conserved role for Spo13, the authors state that the Cdh1-9A mutant is the only known variant showing a similar phenotype than the Spo13 deletion. Although not shown by generating knock-out organisms, the authors should discuss the role of Emi2 (Ref. e.g., Ohe et al 2007) and B-type cyclins (Ref. e.g., Hochegger et al., 2001) for the MI-MII transition of vertebrate female oocytes.

Referee #3:

The manuscript by Rojas et al addresses a question that has plagued the meiosis field for many years: why spo13 mutants undergo a single meiotic division with a mixture of meiosis I and meiosis II events. In answering this question, the authors also

reveal important regulation of meiotic exit. The authors show that Spo13 has very important roles in restraining meiosis II and meiotic exit events from occurring in meiosis I. In a series of cell cycle experiments monitoring expression of several cell cycle regulators, they show that Spo13 bound to polo kinase Cdc5 has important roles in regulating Cdk-Clb1, Ime2, and Ama1 activity. Through these roles, Spo13 prevents premature degradation of the translational repressor Rim4 and the activation of APC/C-Ama1 which targets proteins for degradation at the end of meiosis II. Their results further explain how Rim4 degradation is activated once Spo13 is degraded through a Cdc5-Ime2-Hrr25 cascade.

This is a very important paper for the field of meiotic regulation, with mechanisms that likely have conserved mechanisms. The experiments are carefully and rationally designed and well-executed. In addition, the authors were incredibly thorough. There are just a couple of points that need addressed.

1) The authors mention the timings of a normal metaphase I, a metaphase I in spo13, and in spo13 mad2. Can the authors mention the standard deviation and number of cells analyzed for the average?
2) On page 7, can the authors mention the two bands in the Ama1 western blot and whether they are thought to be modulated forms.
3) I hate to propose any new experiments given the number of experiments in this very thorough manuscript, but the authors mention that the spo13 undergoes one division due to the activity of Ama1. It would be very helpful to show that the spo13 ama1 cells now undergo two divisions (without the Cdc20-meiotic null).
4) On page 8, it should be mentioned that Berchowitz et al uncovered Ama1 as a transcript bound to Rim4.
5) Can the authors mention or discuss how Ama1-6 is present to target substrates prior to Rim4 release of mRNAs on page 15?
6) The Clb1-12D appears to have modifications. Can the authors speculate on what is causing the upshift?

Point-by-point response to reviewer's comments

We thank all reviewers for their insightful comments, which we feel have substantially improved the manuscript. Please find below our point-by-point response in blue text. Major changes in the manuscript are in red text.

Referee #1:
The work by Rojas et al addresses important open questions about the regulation of meiosis and it provides important answers that start to outline a coherent picture about the central regulatory mechanisms that enable the cell to synchronize the two consecutive chromosome separations, in MI and MII. I found the paper a fantastic piece of work that addresses in a very dense but scientifically stringent manner the question, how the cell cycle machinery in meiosis is tuned in such a way that it enables faithful progression from Meiosis I to Meiosis II without exiting the cell cycle. In essence, the work demonstrates that Spo13, in combination with Cdc5 functions as a specific inhibitor of meiosis I processes to timely regulate the activity of the Apc/C Cdc20 and Ama1. A particular role was found for the Spo13-Cdc5 complex to prevent premature activation of various processes while the divisions are still ongoing, in particular by the inhibition of degradation of Rim4 to prevent translation of Rim4 bound transcripts. This paper is a masterpiece of clean experimental design. It combines elegant genetic manipulations with systematic exploration of every aspect of the process by using tricks to expose individual regulatory system and by this to identify how the different branches are connected. With this it also nicely complements previous work by the same group that already demonstrated another important aspect of meiosis regulation, namely the prevention of spore formation at the transition from Meiosis I to meiosis II. I recommend this paper enthusiastically for publication in EMBO J without further experimentation.
We thank the reviewer for his/her positive and very supportive comments on our study.

Minor points:
Page 5:
I anyway struggled a bit with this paragraph that refers to Ama1 function, in particular the sentence "Three properties of Ama1 might make this a challenging task: First, while Ama1 accumulates at metaphase II of an unperturbed meiosis, it nevertheless appears when metaphase I is prolonged (Oelschlaegel et al., 2005)." Could this be explained a bit better? In context I it could be helpful if the Spo13-Cdc5 interaction would also be more thoroughly introduced.
We have revised the sentences on Ama1 accumulation and Spo13's binding to Cdc5 in the introduction. Furthermore, we have added more details on the Cdc5-Spo13 interaction at the beginning of the section on "Ime2 is inhibited by Cdc5-Spo13 and activated by free Cdc5."

General:
I was wondering whether the authors could summarize their findings by extending their wiring diagrams from Figure 9D from their previous paper (Oz et al., 2022)? A paper of this density is hard to comprehend, the wiring diagrams could - maybe be combined with schematic outline the cell structures (spindles, nuclei) in the different phases of Mi-MII? - help the reader to put the pieces together.
We have tried to combine the wiring diagrams of meiotic plaque (MP) assembly (Oz et al., 2022) and meiotic exit but found this to be impractical. While the two diagrams show common features, such as having two "arms", they differ in important aspects. For instance, MP assembly employs a repressor (Spc72) and an activator (Cdk1-Clb1) to confine MP assembly to metaphase II. By contrast, Ama1 is regulated by two repressors, one for synthesis (Rim4) and another one for activity (Cdk1-Clb1-phospho). Activation of APC/C-Ama1 is delayed to meiosis II by the time required for degradation of Rim4 and accumulation of Ama1 to high levels. Furthermore, the crosstalk between the two arms is quite different. Schemes integrating all of these aspects tend to get very convoluted. Showing the diagrams side-by-side is, indeed, revealing but might be more suitable for a review article.
We take the comment on the density of our paper to mean that there is room for improvements in clarity. We agree and have revised several paragraphs: In Results, we revised the sections on "Cdk1-Clb1 inhibits the function of Ama1" and "Mnd2 protects Spo13." In the Discussion, we revised the sections on "The *spo13* mutant fails to repress meiosis II-specific processes" and "Integration of APC/C-Ama1 with the Cdk1-APC/C-Cdc20 oscillator."

Table S1 or Methods: please provide the name and original reference of the parental SK1 strain that is used for this study.
All strains have been constructed in the SK1 background carrying commonly used auxotrophic markers. Its full genotype is given at the end of Appendix Table S1. We have added the reference for the SK1 background (Kane & Roth, 1974).

Referee #2:

In the submitted manuscript, Rojas et al investigate the function of Spo13 in yeast meiosis. Loss of Spo13 causes a single meiotic division, but only when metaphase I is long. Under conditions of SAC-inhibited Cdc20, Spo13 prevents Ama-APC/C activation. Spo13 itself is degraded by Cdc20-APC/C at anaphase I and degradation of Spo13 is required for Ama1 accumulation in MII and degradation of Ama1 substrates. Together with Cdc5, Spo13 prevents Ime2 from mediating the degradation of the translational repressor Rim4. This kinase cascade additionally involves Hrr25. Cdk1/Clb1 promotes both the stabilization and activity of Spo13, while it restrains activity and synthesis of Ama1. Cdc5/Spo13 in turn phosphorylate Clb1, a prerequisite for Ama-APC/C inhibition. Notably, the Ama1-APC/C inhibitory protein Mnd2 allows Spo13 accumulation in MI by protecting it from Ama1-APC/C mediated degradation. This ms addresses a highly an interesting and relevant question, i.e., why do cells exit meiosis after second rather than after the first meiotic division. As one has to expect from the Zachariae lab, the presented data are of highest quality, despite their enormous complexity. WB analyses in combination with fluorescent microscopy studies are applied to investigate the consequences of the different manipulations on meiotic cell cycle progression. All conclusions are in strict accordance with the obtained results and confirmed as well as extended by additional control experiments. This reviewer highly recommends publication of this excellent paper, once the authors have addressed the following points:

We thank the reviewer for his/her positive and very generous comments on our manuscript.

- Fig. S2A: the depletion of Cdc20 results in a metaphase arrest with stable Cdc5. Cdc5 is destabilized in the absence of SPO13 and the authors state that this does not happen when CDH1 is depleted. However, in S2A, Cdc5 is also destabilized to some extent in the absence of both CDC20 and CDH1. This seems to correlate with a partial decrease in Spo13 levels, which however does not correspond to an increase in Ama1 protein. Can the authors explain this behavior?

The reduction in Cdc5 levels in *cdc20 cdh1* cells occurs very late, at 12-14 h in SPM, and is therefore unlikely to be relevant to the regulation of metaphase I. On the other hand, the Cdc5 signals in the *cdc20 spo13Δ cdh1* strain (right) indicate a blotting issue. Thus, we have redetected Cdc5 and Spo13, and signal intensities are now consistent with those of the other proteins. Importantly, the purpose of this experiment was to test whether depletion of Cdh1 affects the degradation of APC/C substrates elicited by the *SPO13* deletion. The experiment shows that depletion of Cdh1 has no effect. Thus, it is Ama1 that prematurely activates APC/C in *cdc20 spo13Δ* cells (Fig 2A).

- One key mechanism revealed in this study is that free Cdc5 activates Ime2, whereas Spo13-bound Cdc5 inhibits it. Although the cell-based evidence is convincing, it would be very nice to see if this can also be recapitulated in Ime2 in vitro kinase assays in the presence of free Cdc5 / Spo13-bound Cdc5. This could then also help to get a better understanding how this surprising difference in substrate regulation by Cdc5 is established on the molecular level (e.g. which sites are phosphorylated in both cases).

While convinced by our cell-based evidence, the reviewer suggests that we identify the phosphorylation sites of free Cdc5 and Cdc5-Spo13 on Ime2, make and characterize the corresponding mutants, and then reconstitute in vitro the activation and inhibition of Ime2 by free Cdc5 and Cdc5-Spo13, respectively. Such experiments are certainly nice but go well beyond our already dense story. We note that we have previously reached a similar conclusion on free Cdc5 activating Ime2 by reconstituting the meiosis II-specific process of MP assembly at prophase, at metaphase I, and in mitotic cells (Oz et al., 2022).

- Fig. 7A: the slower-migrating, phosphorylated species of Ama1 (wild-type) is detectable 2h before Clb1 (or Clb4) becomes evidently expressed. Furthermore, Ama1 seems to get dephosphorylated when the Clb1 levels reach a maximum (at t=8h). How can this be explained? Are there other possible kinases phosphorylating the six S/TP sites?

The slow-migrating Ama1 band at prophase responds to mutations of the S/T-P motifs and is therefore a convenient indicator of the presence of Ama1-6A. However, it neither responds to inhibition of Cdk1 nor does it correlate with Ama1 activity. Please note that Ama1 is active at prophase (Oelschlaegel et al., 2005; Okaz et al., 2012). Instead, a Cdk1- and Clb1-dependent modification appears "on top" of this band. This modification correlates with inhibition of Ama1, as shown in previous work (Okaz et al., 2012) and New Appendix Fig S6C and S6D.

We have repeated the analysis of protein levels in Fig 7A with strains isogenic to those used for imaging of Rim4 in Fig 7B-D. Furthermore, we exposed Ama1 similarly to other Ama1 detections in *cdc20* strains. Under these conditions, the Ama1 signal at prophase is very weak. To avoid confusion, we have deleted the sentence referring to the mobility shift. We do not exclude that Ama1 is phosphorylated by kinases in addition to Cdk1-Clb1 but have no evidence for such kinases. On the other hand, this and previous work provide strong evidence that Cdk1-Clb1 inhibits Ama1 activity: (i) Inhibition of Cdk1-as activates Ama1 in metaphase I-arrested cells that have accumulated Ama1 (Oelschlaegel et al., 2005). (ii) Expression of Clb1 in *ndt80Δ* cells inhibits Ama1-mediated proteolysis in a Cdk1-dependent manner. This is accompanied by a Cdk1- and Clb1-dependent modification of Ama1 (new Appendix Fig S6C and S6D; Okaz et al., 2012). (iii) Cdk1-Clb1 binds to Ama1 (Fig EV4D and EV4E). (iv) APC/C selectively binds the unmodified form of Ama1 (Appendix Fig S2B). (v) Mutation of S/T-P motifs around Ama1's C-box creates a hyperactive activator (Fig 7). In analogy to Cdh1 and Cdc20, the simplest model is that Cdk1-Clb1 phosphorylates the S/T-P motifs around the C-box, thereby preventing Ama1's binding to the APC/C.

Independently of the mechanistic details, our regulatory network rests on three aspects of the relationship between APC/C-Ama1 and Cdk1-Clb1: (i) Clb1 is a substrate of Ama1-dependent proteolysis. We have previously shown that Clb1 is degraded in a manner dependent on APC/C core subunits, the presence of Ama1, and the integrity of Clb1's degrons (Okaz et al., 2012). (ii) Cdk1-Clb1 inhibits Ama1-dependent proteolysis (Fig 6, new Appendix Fig S6C, and Okaz et al., 2012). (iii) Clb1's phosphorylation by Cdc5-Spo13 reduces its Ama1-dependent degradation rate (Fig 8B, Fig 9A). We feel that we have comprehensively addressed these issues.

- Page 15: The authors write "Ama6-A"
We thank the reviewer for pointing out this typo, which has been corrected.

- Fig. 8B: the authors identify by MS several sites on Clb1 potentially phosphorylated by Cdc5, and all of them are serine residues. The authors could further analyze if the sites fit the canonical Polo-consensus motif or if there are other obvious similarities between the sites that allow their phosphorylation by Cdc5.
Our data suggest that Clb1 is phosphorylated by the Cdc5-Spo13 kinase but not by free Cdc5, even though the latter binds to Clb1. Phosphorylation of Clb1 depends on Spo13 (Fig 2A, Appendix Figs S7 and S8), Spo13's ability to bind Cdc5 (Fig EV2B), the presence of Cdc5 (Appendix Fig S7), and the catalytic activity of Cdc5 (Fig 5A). We have added immunoblot analysis of the samples used for phospho-MS (new Appendix Figs S7A and S8A), showing a Spo13-dependent phosphorylation shift of Clb1-GFP.
We have analyzed the phosphorylation sites of Clb1 and compared them to the phosphorylation consensus of Cdc5/PLK1. The preference of Cdc5/PLK1 for D/E/N at position Ser/Thr-2 and a hydrophobic residue at position Ser/Thr+1 is not obvious in Clb1 phosphorylation sites. However, having only 12 sites from a single substrate, we are reluctant to make broader claims. We feel that more substrates need to be identified to determine the phosphorylation consensus of Cdc5-Spo13 and compare it to that of Cdc5/PLK1. This argument also applies to the question of whether Cdc5-Spo13 generally prefers serine to threonine.

- Fig. 8B: the authors observe that "the clb1-12D mutant persists in the presence of Ama1, similar to phosphorylated wild-type Clb1". However, I find it hard to tell if the dephosphorylated wild-type form is less stable than the phosphorylated wild-type form. Indeed, to me both phosphorylated/dephosphorylated wild-type form seem to get degraded with more or less similar efficiencies. Can this be quantified?
This sentence does not refer to the fractions of phosphorylated and unphosphorylated Clb1 in control cells but was meant to compare Clb1-12D (which carries phospho-mimetic aspartates but cannot be phosphorylated) to wild-type Clb1 (which is phosphorylated). We do not know the exact fraction of phosphorylated versus unphosphorylated Clb1 in living control cells. Our data imply, however, that even unphosphorylated Clb1 is stabilized in *cdc20* control cells, like any other APC/C substrate, because APC/C-Ama1 is inhibited. Therefore, the stability of phosphorylated and unphosphorylated Clb1 should be similar, as is seen in Fig 8B. To avoid confusion, we have changed the text to: "phospho-mimetic Clb1-12D persists in the presence of Ama1, similar to wild-type Clb1."

- Fig. 8: the finding that phosphorylated Clb1 inhibits APC/C(Ama1) is very interesting. If possible, it would be very nice to recapitulate this finding (and the dependency on Cdc5-Spo13) in an in vitro ubiquitination assay, to show that these are indeed direct effects. If this experiment is too demanding, I recommend adding some ideas about how direct this effect is in the discussion.
We agree that it would be nice to recapitulate the effect of Cdc5-Spo13 on Clb1's Ama1-dependent proteolysis in an ubiquitinylation assay. We feel, however, that such an analysis goes beyond our already dense story. Our data predict that Clb1 phosphorylation has a relatively small, 2.5-fold effect on Clb1 ubiquitinylation. Recapitulation such a small difference is not without challenges. We have focused on the question of how a small difference in Clb1's degradation rate (Fig 9A) is translated into a large physiological effect – to stay at metaphase I or to prematurely exit from meiosis (Fig 8B). Clearly, more work is required to address the mechanistic questions raised by our study.
We see little reason to suspect that phosphorylation affects Clb1's stability by a mechanism other than reducing the rate of ubiquitinylation by APC/C-Ama1. First, there is precedent for phosphorylation reducing the degradation of APC/C substrates (Pds1, Cdc6, KIF1C; see Discussion). In these cases, it has been concluded that phosphorylation reduces ubiquitinylation. A new twist in our study is that phosphorylation not merely stabilizes Clb1 but allows it to inhibit Ama1. Second, Clb1 is phosphorylated in the region relevant to ubiquitinylation, namely the N-terminal, unstructured extension where degrons and ubiquitinylation sites are located. We now reference AlphaFold (Varadi et al., 2022) for predicting that the N-terminal extension is indeed unstructured. Third, one might ask whether Clb1 phosphorylation has an effect on proteasomal degradation. All Clb1 variants a rapidly degraded in *cdc20 spo13Δ* cells, in which APC/C-Ama1 is highly active (Fig 9B). We do not see differences attributable to proteasomal degradation. Fourth, N-terminal regions of cyclins have been implicated in nuclear transport and/or recruitment to subcellular structures. Clb1 localizes to the nucleus (Buonomo et al., 2003) and carries a potential NLS, which is, however, located downstream of the "last" phosphorylation site. We do not exclude that phosphorylation affects Clb1's localization but note that *clb1-12A* and *clb1-12D* mutants progress normally through meiosis (Fig EV5B), whereas the *CLB1* deletion has severe defects (Appendix Fig S5A, B). Thus, Clb1-12A and Clb12D support all Cdk1-functions required for a normal meiosis (e.g., spindle assembly and activation of APC/C-Cdc20). Clb1

phosphorylation becomes important when Ama1 activity has to be restrained at metaphase I, that is, upon SAC activation. We have shown that meiotic cells transiently activate the SAC at each division (Mengoli et al., 2021). We have also shown that Ama1 localizes to the nucleus at metaphase I (Oelschlaegel et al., 2005). In *cdc20* cells, Clb1-12A is degraded via Ama1, while Clb1-12D inhibits Ama1 (Fig 8B), suggesting that these Clb1-mutants also localize to the nucleus.

- Page 29: in their discussion about the conserved role for Spo13, the authors state that the Cdh1-9A mutant is the only known variant showing a similar phenotype than the Spo13 deletion. Although not shown by generating knock-out organisms, the authors should discuss the role of Emi2 (Ref. e.g., Ohe et al 2007) and B-type cyclins (Ref. e.g., Hochegger et al., 2001) for the MI-MII transition of vertebrate female oocytes.
We discuss mammalian spermatogenesis for two reasons: (i) mammalian genomes encode only two APC/C activators, namely Cdc20 and Cdh1 (also called Fzr1), raising the question of whether our findings on inhibition of Ama1 by Spo13 are relevant to mammals. (ii) similar to yeast, spermatocytes undergo two divisions, which are not interrupted by developmental arrests. Remarkably, spermatocytes from *Meikin* knock-out mice (Kim et al., 2015) or *Cdh1-9A* knock-in mice (Tanno et al., 2020) exit from meiosis after the first division, which resembles the yeast *spo13Δ* and *AMA6-6A* mutant, respectively. The similarity between the *Meikin* knock-out and the *Cdh1-9A* knock-in phenotype has been noted by Tanno et al. (2020). The implication is that Spo13/MEIKIN prevents premature exit from meiosis by inhibiting Ama1 or Cdh1. Indeed, in sequence and regulation, Ama1 is more related to Cdh1 than to Cdc20. We have discussed the features common to and different between Ama1 and Cdh1. Emi2 (or cytostatic factor) is an inhibitor of APC/C-Cdc20, which arrests vertebrate oocytes at metaphase II. The paper of Hochegger et al. (2001) addresses the role of cyclin B translation at entry into meiosis II in *Xenopus* oocytes. While Emi2 and cyclin B translation play crucial roles in the entry into meiosis II, our work focuses on the inhibition of exit from meiosis (a meiosis II-specific process) at meiosis I to achieve a two-division meiosis.

Referee #3:
The manuscript by Rojas et al addresses a question that has plagued the meiosis field for many years: why spo13 mutants undergo a single meiotic division with a mixture of meiosis I and meiosis II events. In answering this question, the authors also reveal important regulation of meiotic exit. The authors show that Spo13 has very important roles in restraining meiosis II and meiotic exit events from occurring in meiosis I. In a series of cell cycle experiments monitoring expression of several cell cycle regulators, they show that Spo13 bound to polo kinase Cdc5 has important roles in regulating Cdk-Clb1, Ime2, and Ama1 activity. Through these roles, Spo13 prevents premature degradation of the translational repressor Rim4 and the activation of APC/C-Ama1 which targets proteins for degradation at the end of meiosis II. Their results further explain how Rim4 degradation is activated once Spo13 is degraded through a Cdc5-Ime2-Hrr25 cascade.
This is a very important paper for the field of meiotic regulation, with mechanisms that likely have conserved mechanisms. The experiments are carefully and rationally designed and well-executed. In addition, the authors were incredibly thorough. There are just a couple of points that need addressed.
We thank the reviewer for his/her positive comments on our work and are happy to hear about its importance for the field of meiotic regulation.

1) The authors mention the timings of a normal metaphase I, a metaphase I in spo13, and in spo13 mad2. Can the authors mention the standard deviation and number of cells analyzed for the average?
Mean +/- SD of the duration of metaphase I is given in the legend to Fig 1A (control and *spo13Δ*) and Fig 1B (*mad2Δ* and *mad2Δ spo13Δ*). Comparisons of the group means using Welch's *t*-test are also provided in the legends. All graphs for imaging experiments show the number of cells counted. For graphs quantifying progression of meiosis, at least 100 cells were scored per timepoint, as stated in Material & Methods.

2) On page 7, can the authors mention the two bands in the Ama1 western blot and whether they are thought to be modulated forms.
Our data suggest that Ama1, like Cdh1 and Cdc20, is phosphorylated at S/T-P sites around the C-box, which inhibits its binding to the APC/C. We have previously shown that Ama1 is modified and inhibited in a manner dependent on Clb1 and Cdk1 activity (Okaz et al., 2012). See also new Appendix Fig S6C and S6D. We now mention the observation that APC/C selectively binds the unmodified form of Ama1 (Appendix Fig S2B) at the end of the section on "Cdk1-Clb1 inhibits the function of Ama1 as an APC/C activator".
3) I hate to propose any new experiments given the number of experiments in this very thorough manuscript, but the authors mention that the spo13 undergoes one division due to the activity of Ama1. It would be very helpful to show that the spo13 ama1 cells now undergo two divisions (without the Cdc20-meiotic null).
spo13Δ ama1Δ double mutants undergo only one division. As we show (Figs 1 and 2) and elaborate in the Discussion, the single-division meiosis of the *spo13Δ* mutant results from two defects: (i) prolongation of metaphase I due to inhibition of Cdc20 by the SAC and (ii) the inability to restrain Ama1 synthesis and activation during an extended metaphase I. While the *AMA1* deletion cannot alleviate the former defect, it creates a new problem: we

have previously shown that deletion of *AMA1* results in stabilization and accumulation of Ndt80 (Okaz et al., 2012). This, together with a prolonged metaphase I, leads to elevated Cdc20 levels in the *spo13Δ ama1Δ* double mutant. Thus, Cdc20 might override the SAC and then prevent entry into meiosis II. To test this, we have created an *NDT80* allele producing low levels of protein. As shown in New Appendix Fig S9, this allele restores a second division in 70% of *spo13Δ ama1Δ* double mutant cells and in 50% of *spo13Δ* single mutant cells.

4) On page 8, it should be mentioned that Berchowitz et al uncovered Ama1 as a transcript bound to Rim4.
We have revised the text accordingly: "Translation of *CLB3* and *SPS4* is repressed by the RNA-binding protein Rim4, which is degraded at entry into metaphase II. Furthermore, these mRNAs and those encoding Ama1 were found among mRNAs copurifying with Rim4 (Berchowitz et al., 2013; Carpenter et al., 2018; Jin et al., 2015)."

5) Can the authors mention or discuss how Ama1-6 is present to target substrates prior to Rim4 release of mRNAs on page 15?
We agree that Ama1 levels are expected to be consistent with the regulation of Ama1 translation by Rim4. We have therefore repeated the analysis of protein levels (new Fig 7A) using strains, which are isogenic to those used for imaging of Rim4-mNG in Fig 7B-D. Thus, all strains in Fig 7 now carry *HSL1p-CDC20*. Furthermore, we have exposed the Ama1 immunoblot similarly to other Ama1 detections in *cdc20* strains and have quantified the Ama1 signal. Since Ama1-6A is a more potent activator than Ama1, it causes degradation of Spo13 and Clb1 in *cdc20* cells (Fig 7A). As a result, Rim4 is degraded, and *AMA1-6A* mRNA is translated earlier in *cdc20 AMA1-6A* cells than in *cdc20 AMA1* cells (Fig 7A and 7B). Since Spo13 function depends on Cdk1-Clb1, degradation of either Spo13 or Clb1 is sufficient to elicit Rim4 degradation (Fig 7C). Finally, Rim4 degradation in *cdc20 AMA1-6A* cells requires signaling through the Cdc5-Ime2-Hrr25 kinase cascade (Fig 7D). Thus, Ama1-6A unleashes the positive feedback loops that couple an increase in Ama1 activity to an increase in Ama1 translation and *vice versa*.

6) The Clb1-12D appears to have modifications. Can the authors speculate on what is causing the upshift?
We suspect that Clb1-12D is phosphorylated on a few sites. A large group of kinases, called acidophilic kinases, prefers negatively charged or already phosphorylated substrates. Indeed, Cdc5/PLK1 is considered an acidophilic kinase. Thus, Cdc5-Spo13 might bind phospho-mimetic Clb1-12D more strongly, leading to phosphorylation of sites less frequently or not at all phosphorylated in wild-type Clb1. We note that no modification is detectable on Clb1-12A.

Additional experiments for the revised manuscript:
1) We have replaced Fig EV4D (Ama1-GFP from control cells versus Esp1-GFP) with a revised version. The original volcano plot was based on an older version of the yeast proteome data base and did not consider the sequences of GFP-tagged bait proteins. The new plot shows the same result as the original version but has somewhat better statistics. The text in Materials & Methods has been revised.

2) We have added a new volcano plot of Ama1-GFP from *spo13Δ* cells versus Esp1-GFP (new Appendix Fig S6A). This purification was performed together with the Ama1-GFP purification from control cells in Fig EV4D. Furthermore, we have added detection of Cdk1 to the immunoprecipitation of Ama1-myc9 from control and *spo13Δ* cells in Appendix Fig S6B (previously Fig EV4F). Together, these data show that Cdk1-Clb1 can bind to Ama1 in *spo13Δ* cells, arguing against the possibility that Ama1 is more active in *spo13Δ* cells because it cannot bind its inhibitor Cdk1-Clb1. Indeed, when expressed in *ndt80Δ* cells, Clb1 is capable of inhibiting Ama1 in the absence of Spo13 (new Appendix Fig S6C).

3) Since Clb3 is translated together with Ama1 and binds to Ama1, Cdk1-Clb3 seems ideally suited to assist Cdk1-Clb1 is restraining Ama1-dependent proteolysis. However, deletion of *CLB3* has no effect on Rim4 degradation, even in cells lacking *CLB1* (new Appendix Fig S5D). Furthermore, expression of Clb3 in prophase-arrested *ndt80Δ* cells does not affect the activity of Ama1 (new Appendix Fig S6D). Thus, while attractive at first glance, we find no evidence for the idea that Clb3's translational control and binding to Ama1 play a role in restraining Ama1-dependent proteolysis.

4) Appendix Figure S1A has been replaced by a better version comparing *cdc20* with *cdc20 spo13Δ* cells. The *SPO13* deletion causes Cdc20-depleted cells to undergo a division.

Dr. Wolfgang Zachariae
Max Planck Institute of Biochemistry
Laboratory of Chromosome Biology
Am Klopferspitz 18
Martinsried 82152
Germany

1st Sep 2023

Re: EMBOJ-2023-114288R
Spo13/Meikin ensures a two-division meiosis by preventing the activation of APC/C-Ama1 at meiosis I

Dear Wolfgang,

Thank you for submitting your revised manuscript to The EMBO Journal. One of the original referees has once more looked into it and assessed your responses (see below), and was fully satisfied with your revisions. I am therefore happy to say that we are ready to accept the study for publication, following incorporation of the following few editorial points:

- Pre-acceptance checks by our data editors have raised a few queries with the data descriptors (missing scale bars) in the figure legends, which you will find as comments in the attached edited/commented Word document with activated "Track changes" option. I would appreciate if you incorporated the requested final text modifications and answered the Figure legend queries directly in this version (and modified figures where necessary), uploading the edited main text document upon resubmission with changes/additions still highlighted via the "Track changes" option, to facilitate our final checking.

- Please reduce the number of keywords to five, ideally favoring general terms over.

- Please correct the reference list, making sure that for references with multiple authors, the first up to 10 (rather than 20) authors are listed, followed by 'et al.' after that (please refer to our Guide to Authors for additional information on EMBO J reference format).

- As we are switching from a free-text author contribution statement towards a more formal statement based on Contributor Role Taxonomy (CRediT) terms, please remove the present Author Contribution section and instead specify each author's contribution(s) directly in the Author Information page of our submission system during upload of the final manuscript. See https://casrai.org/credit/ for more information.

- Please rename the Conflict of Interest section into "Disclosure and Competing Interests Statement", in accordance with our update Guide to Authors (https://www.embopress.org/competing-interests(

- Please remove the Appendix Figure Legends from the main manuscript text, they should only be present in the Appendix PDF itself.

- Finally, in the Data Availability section, please remove the reviewer tokens now, and ensure that all listed datasets will be publicly released at this point.

I am therefore returning the manuscript to you for a final round of minor revision, to allow you to make these adjustments and upload all modified files. Once we will have received them, we should be ready to swiftly proceed with formal acceptance and production of the manuscript.

With kind regards,

Hartmut

Hartmut Vodermaier, PhD
Senior Editor, The EMBO Journal
h.vodermaier@embojournal.org

*** PLEASE NOTE: All revised manuscript are subject to initial checks for completeness and adherence to our formatting guidelines. Revisions may be returned to the authors and delayed in their editorial re-evaluation if they fail to comply to the following requirements (see also our Guide to Authors for further information):

1) Every manuscript requires a Data Availability section (even if only stating that no deposited datasets are included). Primary datasets or computer code produced in the current study have to be deposited in appropriate public repositories prior to resubmission, and reviewer access details provided in case that public access is not yet allowed. Further information: embopress.org/page/journal/14602075/authorguide#dataavailability

2) Each figure legend must specify
- size of the scale bars that are mandatory for all micrograph panels
- the statistical test used to generate error bars and P-values
- the type error bars (e.g., S.E.M., S.D.)
- the number (n) and nature (biological or technical replicate) of independent experiments underlying each data point
- Figures may not include error bars for experiments with n<3; scatter plots showing individual data points should be used instead.

3) Revised manuscript text (including main tables, and figure legends for main and EV figures) has to be submitted as editable text file (e.g., .docx format). We encourage highlighting of changes (e.g., via text color) for the referees' reference.

4) Each main and each Expanded View (EV) figure should be uploaded as individual production-quality files (preferably in .eps, .tif, .jpg formats). For suggestions on figure preparation/layout, please refer to our Figure Preparation Guidelines: http://bit.ly/EMBOPressFigurePreparationGuideline

5) Point-by-point response letters should include the original referee comments in full together with your detailed responses to them (and to specific editor requests if applicable), and also be uploaded as editable (e.g., .docx) text files.

6) Please complete our Author Checklist, and make sure that information entered into the checklist is also reflected in the manuscript; the checklist will be available to readers as part of the Review Process File. A download link is found at the top of our Guide to Authors: embopress.org/page/journal/14602075/authorguide

7) All authors listed as (co-)corresponding need to deposit, in their respective author profiles in our submission system, a unique ORCiD identifier linked to their name. Please see our Guide to Authors for detailed instructions.

8) Please note that supplementary information at EMBO Press has been superseded by the 'Expanded View' for inclusion of additional figures, tables, movies or datasets; with up to five EV Figures being typeset and directly accessible in the HTML version of the article. For details and guidance, please refer to: embopress.org/page/journal/14602075/authorguide#expandedview

9) Digital image enhancement is acceptable practice, as long as it accurately represents the original data and conforms to community standards. If a figure has been subjected to significant electronic manipulation, this must be clearly noted in the figure legend and/or the 'Materials and Methods' section. The editors reserve the right to request original versions of figures and the original images that were used to assemble the figure. Finally, we generally encourage uploading of numerical as well as gel/blot image source data; for details see: embopress.org/page/journal/14602075/authorguide#sourcedata

At EMBO Press, we ask authors to provide source data for the main manuscript figures. Our source data coordinator will contact you to discuss which figure panels we would need source data for and will also provide you with helpful tips on how to upload and organize the files.

Further information is available in our Guide For Authors: https://www.embopress.org/page/journal/14602075/authorguide

In the interest of ensuring the conceptual advance provided by the work, we recommend submitting a revision within 3 months (30th Nov 2023). Please discuss the revision progress ahead of this time with the editor if you require more time to complete the revisions. Use the link below to submit your revision:

Link Not Available

Referee #3:

The authors have added experimental data or changes to wording to address all of the concerns. There are no further concerns. This is a strong manuscript that will be of strong interest to the community.

Response to reviewer's comments

We thank the reviewer for appreciating our new experiments and the changes to the manuscript text. Following the advice of the editor we have made the following, additional changes to the manuscript text:

- Added Scale Bars to all microscope images and indicated their size in the figure legends.
- Reduced to five the number of Key Words.
- Removed the free-text Author Contribution Statement from the manuscript text.
- Renamed the Disclosure and Competing Interests Statement.
- Removed the reviewer tokens from the Data Availability section and activated public access.
- Marked in Red the changes above in the manuscript text.
- Limited to 10 the maximum number of author names in the reference list.
- Removed the Appendix Figure Legends from the manuscript text.
- Removed a number of typos from the Source Data. Please only use the newly submitted Source Data.

Dr. Wolfgang Zachariae
Max Planck Institute of Biochemistry
Laboratory of Chromosome Biology
Am Klopferspitz 18
Martinsried 82152
Germany

4th Sep 2023

Re: EMBOJ-2023-114288R1
Spo13/Meikin ensures a two-division meiosis by preventing the activation of APC/C-Ama1 at meiosis I

Dear Dr. Zachariae,

Thank you for submitting your final revised manuscript for our consideration. I am pleased to inform you that we have now accepted it for publication in The EMBO Journal.

Your article will be processed for publication in The EMBO Journal by EMBO Press and Wiley, who will contact you with further information regarding production/publication procedures and license requirements. You will also be provided with page proofs after copy-editing and typesetting of main manuscript and expanded view figure files.

Should you be planning a Press Release on your article, please get in contact with embojournal@wiley.com as early as possible, in order to coordinate publication and release dates.

Thank you again for this contribution to The EMBO Journal and congratulations on a successful publication! Please consider us again in the future for your most exciting work.

Yours sincerely,

Hartmut Vodermaier

Hartmut Vodermaier, PhD
Senior Editor, The EMBO Journal
h.vodermaier@embojournal.org

Please note that it is EMBO Journal policy for the transcript of the editorial process (containing referee reports and your response letter) to be published as an online supplement to each paper. If you do NOT want this, you will need to inform the Editorial Office via email immediately. More information is available here:
https://www.embopress.org/page/journal/14602075/authorguide#transparentprocess

EMBO PRESS

YOU MUST COMPLETE ALL CELLS WITH A PINK BACKGROUND ➔

PLEASE NOTE THAT THIS CHECKLIST WILL BE PUBLISHED ALONGSIDE YOUR PAPER

Corresponding Author Name: Wolfgang Zachariae
Journal Submitted to: EMBO Journal
Manuscript Number: EMBOJ-2023-114288

Reporting Checklist For Life Sciences Articles (Rev. June 2017)

This checklist is used to ensure good reporting standards and to improve the reproducibility of published results. These guidelines are consistent with the Principles and Guidelines for Reporting Preclinical Research issued by the NIH in 2014. Please follow the journal's authorship guidelines in preparing your manuscript.

A- Figures
1. Data
The data shown in figures should satisfy the following conditions:

- the data were obtained and processed according to the field's best practice and are presented to reflect the results of the experiments in an accurate and unbiased manner.
- figure panels include only data points, measurements or observations that can be compared to each other in a scientifically meaningful way.
- graphs include clearly labeled error bars for independent experiments and sample sizes. Unless justified, error bars should not be shown for technical replicates.
- if n< 5, the individual data points from each experiment should be plotted and any statistical test employed should be justified
- Source Data should be included to report the data underlying graphs. Please follow the guidelines set out in the author ship guidelines on Data Presentation.

2. Captions

Each figure caption should contain the following information, for each panel where they are relevant:

- a specification of the experimental system investigated (eg cell line, species name).
- the assay(s) and method(s) used to carry out the reported observations and measurements
- an explicit mention of the biological and chemical entity(ies) that are being measured.
- an explicit mention of the biological and chemical entity(ies) that are altered/varied/perturbed in a controlled manner.

- the exact sample size (n) for each experimental group/condition, given as a number, not a range;
- a description of the sample collection allowing the reader to understand whether the samples represent technical or biological replicates (including how many animals, litters, cultures, etc.).
- a statement of how many times the experiment shown was independently replicated in the laboratory.
- definitions of statistical methods and measures:
  - common tests, such as t-test (please specify whether paired vs. unpaired), simple χ2 tests, Wilcoxon and Mann-Whitney tests, can be unambiguously identified by name only, but more complex techniques should be described in the methods section;
  - are tests one-sided or two-sided?
  - are there adjustments for multiple comparisons?
  - exact statistical test results, e.g., P values = x but not P values < x;
  - definition of 'center values' as median or average;
  - definition of error bars as s.d. or s.e.m.

Any descriptions too long for the figure legend should be included in the methods section and/or with the source data.

In the pink boxes below, please ensure that the answers to the following questions are reported in the manuscript itself. Every question should be answered. If the question is not relevant to your research, please write NA (non applicable). We encourage you to include a specific subsection in the methods section for statistics, reagents, animal models and human subjects.

USEFUL LINKS FOR COMPLETING THIS FORM

http://www.antibodypedia.com	Antibodypedia
http://1degreebio.org	1DegreeBio
http://www.equator-network.org/reporting-guidelines/improving-bioscience-research-reporti	ARRIVE Guidelines
http://grants.nih.gov/grants/olaw/olaw.htm	NIH Guidelines in animal use
http://www.mrc.ac.uk/Ourresearch/Ethicsresearchguidance/Useofanimals/index.htm	MRC Guidelines on animal use
http://ClinicalTrials.gov	Clinical Trial registration
http://www.consort-statement.org	CONSORT Flow Diagram
http://www.consort-statement.org/checklists/view/32-consort/66-title	CONSORT Check List
http://www.equator-network.org/reporting-guidelines/reporting-recommendations-for-tumo	REMARK Reporting Guidelines (marker prognostic studies)
http://datadryad.org	Dryad
http://figshare.com	Figshare
http://www.ncbi.nlm.nih.gov/gap	dbGAP
http://www.ebi.ac.uk/ega	EGA
http://biomodels.net/	Biomodels Database
http://biomodels.net/miriam/	MIRIAM Guidelines
http://jjj.biochem.sun.ac.za	JWS Online
https://osp.od.nih.gov/biosafety-biosecurity-and-emerging-biotechnology/	Biosecurity Documents from NIH
http://www.selectagents.gov/	List of Select Agents

B- Statistics and general methods

Please fill out these boxes ➔ (Do not worry if you cannot see all your text once you press return)

1.a. How was the sample size chosen to ensure adequate power to detect a pre-specified effect size?	We did not pre-specify an effect size.
1.b. For animal studies, include a statement about sample size estimate even if no statistical methods were used.	No animals were used; this study uses budding yeast.
2. Describe inclusion/exclusion criteria if samples or animals were excluded from the analysis. Were the criteria pre-established?	No samples were excluded from analysis.
3. Were any steps taken to minimize the effects of subjective bias when allocating animals/samples to treatment (e.g. randomization procedure)? If yes, please describe.	Randomization procedures were not applied.
For animal studies, include a statement about randomization even if no randomization was used.	No animals were used; this study uses budding yeast.
4.a. Were any steps taken to minimize the effects of subjective bias during group allocation or/and when assessing results (e.g. blinding of the investigator)? If yes please describe.	Blinding was not performed.
4.b. For animal studies, include a statement about blinding even if no blinding was done	No animals were used; this study uses budding yeast.
5. For every figure, are statistical tests justified as appropriate?	Yes, to the best of our knowledge.
Do the data meet the assumptions of the tests (e.g., normal distribution)? Describe any methods used to assess it.	Distributions tend to be symmteric or slightly skewed. Thus, we decided, a priori, to use Welch's t-test to compare group means and provide 95% confidence intervals for the difference between group means. With a balanced design and samples sizes of n = 100 per group, Welch's t-test is robust to moderate deviations from normality.

Is there an estimate of variation within each group of data?	An estimate of variation (SD) is provided, either in the figures, the figure legends, or in the text.
Is the variance similar between the groups that are being statistically compared?	We did not assume the variance to be similar between gropus. We used the t-test with correction for unequal variance (Welch's t-test) to compare group means.

C- Reagents

6. To show that antibodies were profiled for use in the system under study (assay and species), provide a citation, catalog number and/or clone number, supplementary information or reference to an antibody validation profile. e.g., Antibodypedia (see link list at top right), 1DegreeBio (see link list at top right).	Citations and/or supplier and catalog numbers for antibodies are provided.
7. Identify the source of cell lines and report if they were recently authenticated (e.g., by STR profiling) and tested for mycoplasma contamination.	A table with the full genotypes of all Saccharomyces cerevisiae strains used in this study is provided as Appendix Table S1.

* for all hyperlinks, please see the table at the top right of the document

D- Animal Models

8. Report species, strain, gender, age of animals and genetic modification status where applicable. Please detail housing and husbandry conditions and the source of animals.	NA
9. For experiments involving live vertebrates, include a statement of compliance with ethical regulations and identify the committee(s) approving the experiments.	NA
10. We recommend consulting the ARRIVE guidelines (see link list at top right) (PLoS Biol. 8(6), e1000412, 2010) to ensure that other relevant aspects of animal studies are adequately reported. See author guidelines, under 'Reporting Guidelines'. See also: NIH (see link list at top right) and MRC (see link list at top right) recommendations. Please confirm compliance.	NA

E- Human Subjects

11. Identify the committee(s) approving the study protocol.	NA
12. Include a statement confirming that informed consent was obtained from all subjects and that the experiments conformed to the principles set out in the WMA Declaration of Helsinki and the Department of Health and Human Services Belmont Report.	NA
13. For publication of patient photos, include a statement confirming that consent to publish was obtained.	NA
14. Report any restrictions on the availability (and/or on the use) of human data or samples.	NA
15. Report the clinical trial registration number (at ClinicalTrials.gov or equivalent), where applicable.	NA
16. For phase II and III randomized controlled trials, please refer to the CONSORT flow diagram (see link list at top right) and submit the CONSORT checklist (see link list at top right) with your submission. See author guidelines, under 'Reporting Guidelines'. Please confirm you have submitted this list.	NA
17. For tumor marker prognostic studies, we recommend that you follow the REMARK reporting guidelines (see link list at top right). See author guidelines, under 'Reporting Guidelines'. Please confirm you have followed these guidelines.	NA

F- Data Accessibility

18: Provide a "Data Availability" section at the end of the Materials & Methods, listing the accession codes for data generated in this study and deposited in a public database (e.g. RNA-Seq data: Gene Expression Omnibus GSE39462, Proteomics data: PRIDE PXD000208 etc.) Please refer to our author guidelines for 'Data Deposition'.<br><br>Data deposition in a public repository is mandatory for:<br>a. Protein, DNA and RNA sequences<br>b. Macromolecular structures<br>c. Crystallographic data for small molecules<br>d. Functional genomics data<br>e. Proteomics and molecular interactions	Under "Data Availability", we state that raw mass spectrometry data are available at the PRIDE datbase. The identifier is provided..
19. Deposition is strongly recommended for any datasets that are central and integral to the study; please consider the journal's data policy. If no structured public repository exists for a given data type, we encourage the provision of datasets in the manuscript as a Supplementary Document (see author guidelines under 'Expanded View' or in unstructured repositories such as Dryad (see link list at top right) or Figshare (see link list at top right).	NA
20. Access to human clinical and genomic datasets should be provided with as few restrictions as possible while respecting ethical obligations to the patients and relevant medical and legal issues. If practically possible and compatible with the individual consent agreement used in the study, such data should be deposited in one of the major public access-controlled repositories such as dbGAP (see link list at top right) or EGA (see link list at top right).	NA
21. Computational models that are central and integral to a study should be shared without restrictions and provided in a machine-readable form. The relevant accession numbers or links should be provided. When possible, standardized format (SBML, CellML) should be used instead of scripts (e.g. MATLAB). Authors are strongly encouraged to follow the MIRIAM guidelines (see link list at top right) and deposit their model in a public database such as Biomodels (see link list at top right) or JWS Online (see link list at top right). If computer source code is provided with the paper, it should be deposited in a public repository or included in supplementary information.	NA

G- Dual use research of concern

22. Could your study fall under dual use research restrictions? Please check biosecurity documents (see link list at top right) and list of select agents and toxins (APHIS/CDC) (see link list at top right). According to our biosecurity guidelines, provide a statement only if it could.	No.
